# Photoinduced Atom Transfer Radical Addition/Cyclization Reaction between Alkynes or Alkenes with Unsaturated α-Halogenated Carbonyls

**DOI:** 10.3390/molecules26226781

**Published:** 2021-11-10

**Authors:** Kazuki Matsuo, Tadashi Yoshitake, Eiji Yamaguchi, Akichika Itoh

**Affiliations:** Department of Pharmacy, Gifu Pharmaceutical University, 1-25-4 Daigaku-Nishi, Gifu 501-1196, Japan; 136031@gifu-pu.ac.jp (K.M.); 165093@gifu-pu.ac.jp (T.Y.)

**Keywords:** halogen-bonding, ATRA reaction, ATRC reaction, radical reaction, photoreaction

## Abstract

We have developed a photochemical ATRA/ATRC reaction that is mediated by halogen bonding interactions. This reaction is caused by the reaction of malonic acid ester derivatives containing bromine or iodine with unsaturated compounds such as alkenes and alkynes in the presence of diisopropylethylamine under visible light irradiation. As a result of various control experiments, it was found that the formation of complexes between amines and halogens by halogen-bonding interaction occurs in the reaction system, followed by the cleavage of the carbon–halogen bonds by visible light, resulting in the formation of carbon radicals. In this reaction, a variety of substrates can be used, and the products, cyclopentenes and cyclopentanes, were obtained by intermolecular addition and intramolecular cyclization.

## 1. Introduction

Cyclic compounds are ubiquitous in natural products and artificially synthesized functional molecules. They also have wide applications in various areas of organic chemistry, such as medicinal and materials chemistry [1,2,3]. Consequently, the efficient preparation of cyclic systems continues to be an important area of modern organic chemistry. The formation of such cyclic systems by carbon–carbon bond formation has increasingly been achieved by the use of free radical cyclization protocols [4].

Atom transfer radical addition (ATRA) was pioneered by Kharasch approximately 70 years ago, and the ATRA reaction of halogen compounds to alkenes is a powerful and atom-economical way to form carbon–carbon and carbon–halogen bonds simultaneously [5,6,7]. An intramolecular version of this reaction, known as atom transfer radical cyclization (ATRC), has been extensively exploited and refined into a versatile and powerful tool for organic synthesis, and provides a convenient route to the construction of cyclic frameworks [8,9]. It has long been known that a wide range of metal complexes, such as copper [10], iron [11], ruthenium [12], palladium [13], and nickel [14], can catalyze such ATRC reactions under thermal conditions. However, the radical generation process generally requires a high reaction temperature and is difficult, due to certain competitive side reactions that generate reactive intermediates.

**Figure 1:**
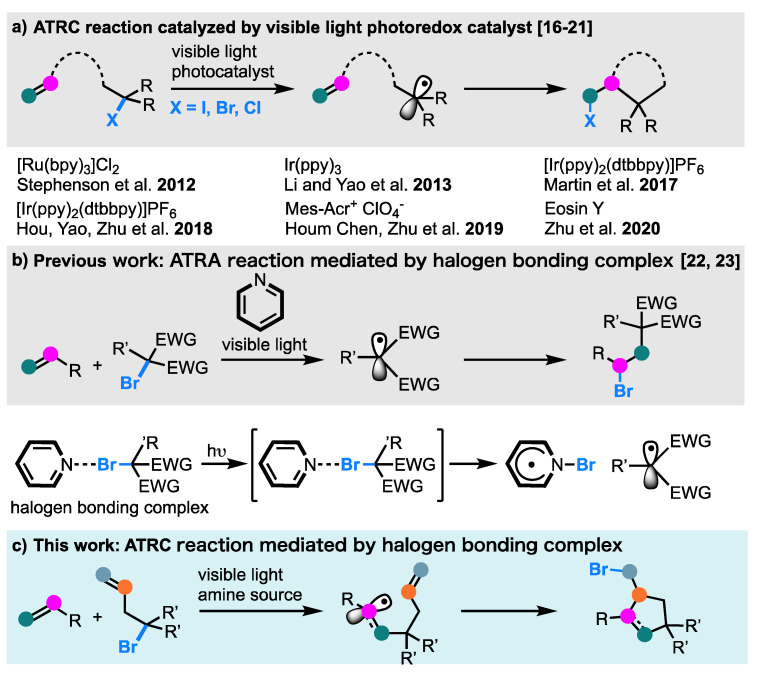
The atom-transfer radical cyclization (ATRC) protocol. (**a**) The developed photochemical ATRC reaction. (**b**) Our previous halogen-bonding complex mediated photochemical ATRA reaction. (**c**) Halogen-bonding complex mediated photochemical ATRA/ATRC reaction.

Recently, metal-based photoredox catalysis and organo-photocatalysis that are driven by visible light have further expanded the potential of the ATRC technology (Figure 1a) [15]. In 2012, Stephenson et al. developed an intramolecular ATRA reaction of unsaturated hydrocarbons with bromomalonates as side chains, which is promoted by a visible light photoredox catalysis [16]. Subsequently, visible-light-responsive photocatalysts, based on Ir and Ru mediating the ATRC reaction, have been developed [17,18,19]. Moreover, non-metallic photocatalysts such as organo-photocatalyst were also developed. For example, in 2019, Zhu et al. described a visible-light-driven chlorotrifluoromethylative and chlorotrichloromethylative cyclization approach by using organo-photocatalysts; a series of chlorotrifluoromethylated and chlorotrichloromethylated pyrrolidines, piperidines, and cyclopentanes were obtained in moderate to good yields [20]. Additionally, a visible-light-mediated intermolecular radical cyclization approach to access the heterocycle was developed by using Eosin Y as the catalyst for the hydrogen atom transfer [21]. However, methods for the synthesis of cyclic systems through an intermolecular ATRC process have not been extensively developed. Therefore, the development of a novel and efficient strategy for the intermolecular ATRC reactions between alkyl halides with alkynes would be extremely valuable.

Recently, we have reported that the in situ generated halogen-bonding complex of an alkyl halide with pyridines enabled the ATRA reactions of olefins (Figure 1b) [22]. The carbohalogenation of olefins with carbon tetrabromide generated a variety of products in moderate to good yields when irradiated at 450 nm. In addition, we reported further applications for this reaction, which apply them to a bromomalonate ester instead of a carbon tetrabromide [23].

Therefore, inspired by the methodologies developed, we started to challenge the advantages of the intramolecular ATRA/ATRC process that 2-halogenmalonate-containing, unsaturated functional groups could react with alkynes or alkenes, leading to the construction of substituted cyclopentenes or cyclopentanes (Figure 1c).

## 2. Results and Discussion

Based on the previous report for a halogen-bonding-initiated ATRA reaction, this study was initiated by examining the intramolecular ATRA/ATRC reaction between ethynylbenzene (**1a**) and dimethyl 2-allyl-2-bromomalonate (**2a**), which were chosen as reaction partners using the halogen-bonding approach (Table 1, see Tables S1–S5 for full detail for optimization study). First, the wavelength of the light was evaluated by using 5.0 mol% of 4-Ph-pyridine as the catalyst. Various wavelengths of the light were explored, showing that 420 nm was the most effective and gave **3a** in 50% isolated yield (Entries 1–4) [24]. When the reaction was carried out without a catalyst, it was found that the reaction proceeded under UV light (Table S5). Therefore, it is suggested that the yield of the reaction, when irradiated by 380 nm LED, was increased by the background reaction without halogen-bonding interactions. Next, we examined the impact of solvent concentration on the outcome of the reaction (Entries 3 and 5–7). The dramatic effect of doubling solvent volume is also worth noting (cf. Entries 3 and 5) [25]. Since the lone pair of 4-Ph-pyridine reacts with the σ-hole of the halogen in the formation of the halogen-bonding complex, we hypothesized that the electron-donating alkyl amines would show a stronger interaction and increased reactivity. As expected, the addition of 1.0 equiv of DIPEA (*N*,*N*-diisopropylethylamine) improved the isolated yield to 71% (Entry 8). Surprisingly, we found that 4-Ph-pyridine as a catalyst was not necessary (Entry 9). Amines are known to be good halogen-bonding acceptors, and it is assumed that the use of an electron-rich alkylamine such as DIPEA would allow the reaction to proceed without the use of pyridine, as previously reported, to yield the corresponding product **3a** [22,23]. Finally, the loading of compound **2a** was increased to 3.0 equiv, which resulted in the highest reaction yield of 79% (isolated yield) (Entries 10 and 11).

**Figure 2:**
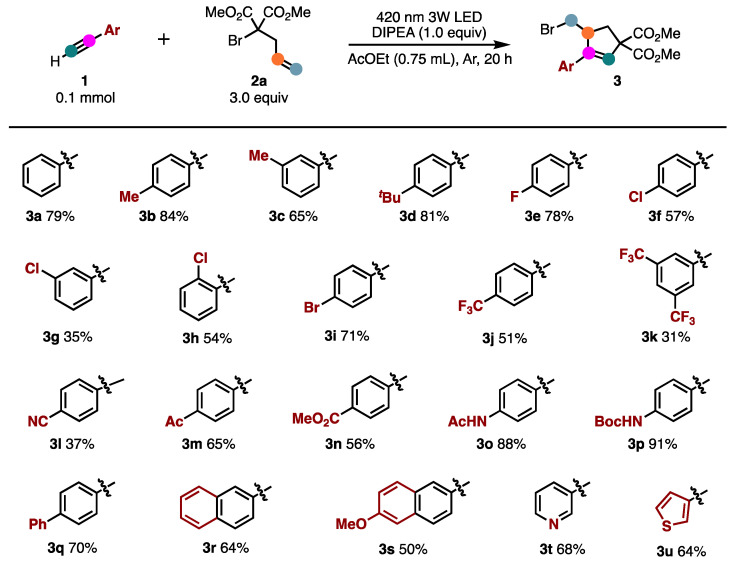
Scope of alkynes. Isolated yields.

The scope of the photoinduced ATRA/ATRC reaction was further explored by varying the alkyne **1** to optimize the reaction conditions (Figure 2). Various functional groups on the benzene ring were examined, and most were tolerated under the optimized conditions. With alkyl substitution on benzene, these compounds reacted effectively to yield the desired products (**3b**–**3d**) in 65–84% yields. Halogen atoms such as fluoro, chloro, and bromo on the aromatic ring were not affected under the present reaction conditions to afford the corresponding products (**3e**–**3i**) in moderate to good yields, thus providing ample opportunity for further elaboration by the transition-metal-catalyzed cross-coupling reactions. The substrates bearing electron-withdrawing (CF_3_, CN, Ac, CO_2_Me, NHAc and NHBoc) groups on the benzene ring were also investigated and smoothly converted to products (**3j**–**3p**) in moderate to good yields. In addition, alkynes bearing 4-phenyl, 2-naphthyl, 3-pyridyl, and 3-thyenyl could also react smoothly with **2a** to afford the expected products (**3q**–**3t**) in good yields.

**Figure 3:**
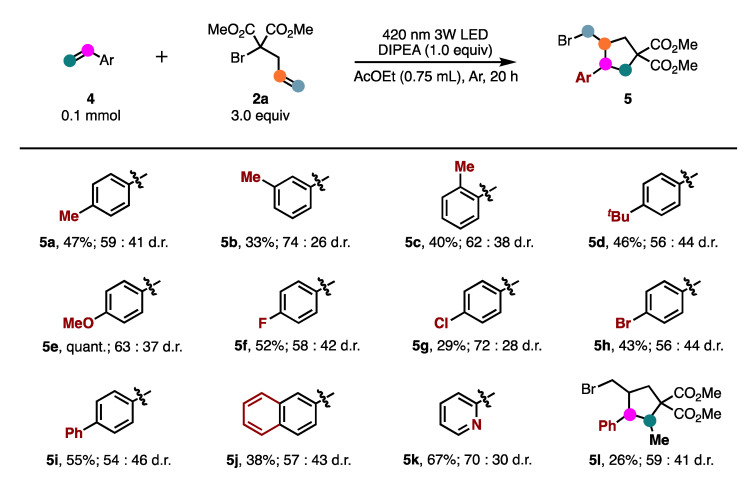
Scope of alkenes. Isolated yields.

Next, various aromatic alkenes were subjected to this optimized ATRC reaction (Figure 3). Similar to the alkynes, the styrenes were tolerated under these reaction conditions and gave the corresponding product in moderate to good yields. With alkyl substitution on the benzene ring, these substrates reacted efficiently to obtain the desired products (**5a**–**5d**) in moderate yields. Interestingly, the use of 4-MeO-substituted styrene (**4e**) yielded the desired product quantitatively. Halogen atoms such as fluoro, chloro, and bromo on the aromatic ring were not affected under the present reaction conditions to afford the corresponding products **5f**–**5h** in moderate yields. In addition, compounds with 4-phenyl, 2-naphthyl, and 2-pyridyl could also react smoothly with **2a** to afford the expected products **5i**–**5k** in good yields. The use of an internal alkene such as β-methylstyrene (**4l**) resulted in low yield.

**Figure 4:**
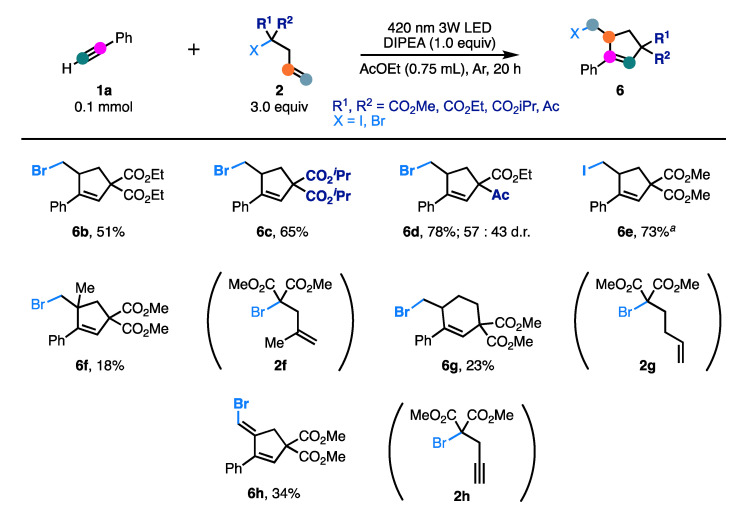
Scope of unsaturated α-halogenocarbonyls. Isolated yields. *^a^* Without *N*,*N*-diisopropylethylamine (DIPEA).

Additionally, we turned our attention to exploring various unsaturated α-halogenocarbonyl compounds (**2a**) under standard conditions (Figure 4). Diethyl 2-allyl-2-bromomalonate (**2b**) and Diisopropyl 2-allyl-2-bromomalonate (**2c**) were also employed and resulted in the production of **6b** and **6c** in moderate yields. The reaction could be applied to other activated organobromides, as exemplified by the construction of **6d** in good yield. Pleasingly, with dimethyl 2-allyl-2-iodomalonate (**2e**) acting as the carbohalogenation reagent, iodinated product **6e** was successfully obtained in moderate yield. When dimethyl 2-(2-methylallyl)-bromomalonate **2f** was employed instead of **2a**, the cyclopentene ring with a quaternary carbon center **6f** was successfully synthesized in a low yield. Additionally, the homoallyl-substituted counterpart **2g** led to the corresponding product **6g** in low yield. Finally, the reaction was investigated using a malonic acid ester derivative with a propargyl group as substrate **2h**. The generated vinyl radicals underwent intramolecular addition reaction to alkynes to give the corresponding vinyl bromides **6h** in low yield. The ^1^H-^1^H NOESY spectrum identified the geometry of the product **6h** as an *E*-isomer.

**Figure 5:**
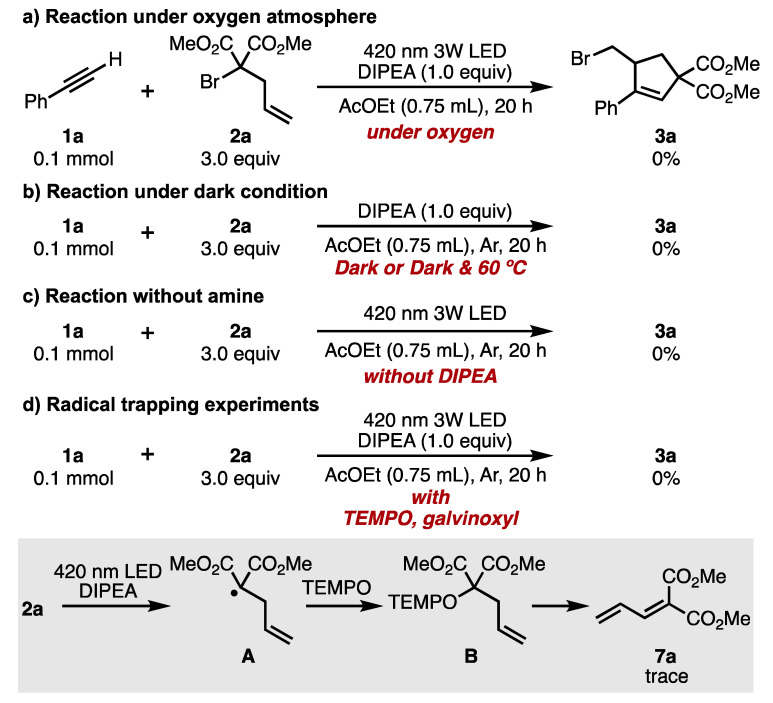
Control experiments. (**a**) Reaction under oxygen atmosphere. (**b**) Reaction under dark condition. (**c**) Reaction without amine. (**d**) Radical trapping experiments.

Further mechanistic investigations were also conducted (Figure 5). The reaction between **1a** and **2a** under the optimized conditions and oxygen atmosphere resulted in no reaction (Figure 5a). The result indicated that the photoexcited active species was quenched by a triplet oxygen molecule [25]. Furthermore, the reaction was also investigated under dark conditions or when heated to 60 °C under dark conditions, revealing that no ATRA/ATRC product was obtained (Figure 5b). These results suggested that light is essential for this reaction. When the reaction was examined in the absence of DIPEA, no desired product was obtained, indicating that DIPEA acted as a halogen-bonding donor (Figure 5c). Moreover, the radical trapping experiments using TEMPO and galvinoxyl as a radical trapping agent did not proceed desired reaction under the optimized conditions. In the reaction employing TEMPO, the radical trapping product **B** was not given, but the diene product **7a** was obtained instead (Figure 5d) [26]. Based on a previous report, we assume that the malonate radical **A**, which is formed from **2a**, is trapped by TEMPO to form the intermediate **B**. With the assistance of another molecule of TEMPO, intermediate **B** undergoes elimination to form product **7a** [27,28]. These results indicate that the present ATRA/ATRC reaction might proceed via a radical intermediate.

**Figure 6:**
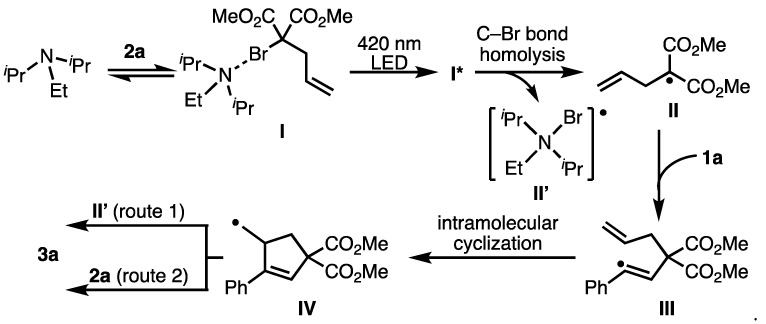
Possible reaction mechanism.

Based on the previously reported results and control experiments, the possible reaction pathway for the generation of the malonate radical is shown in Figure 6. The reaction was initiated by the photoexcitation of the halogen-bonding complex **I**, which is formed by the reaction of **2a** with DIPEA to produce the excited state I*. The photoexcitation of **I’** led to the C-Br bond homolysis, generating the C-centered malonate radical **II** and the radical intermediate **II’**. Next, the resulting C-centered radical **II** reacted with the alkynes in the ATRA manner to generate the radical intermediate **III**. The radical intermediate **III** participates in an intramolecular radical cyclization to generate the radical intermediate **IV**. Subsequently, the radical species **IV** reacted with **II’** to provide the corresponding ATRC product (route 1). Another possibility is a radical chain mechanism, where the carbon radical **IV** reacts with dimethyl 2-allyl-2-bromomalonate **2a** to give the ATRC product and regenerate **II** (route 2).

## 3. Materials and Methods

### 3.1. General Information

Unless otherwise noted, all reactants or reagents, including dry solvents, were obtained from commercial suppliers and used as received. Analytical thin-layer chromatography (TLC) was carried out by using 0.25 mm commercial silica gel plates from Merck (Darmstadt, Germany) (silica gel 60 F_254_). Flash column chromatography was performed with Kanto (Tokyo, Japan) silica gel 60 N (Spherical, Neutral, 40–50 nm). Visualization of the developed chromatogram was performed by a UV lamp (254 nm) and vanillin or basic potassium permanganate stain. NMR spectra were recorded on a JEOL ECA 500 spectrometer (500 MHz for ^1^H NMR and 125 MHz for ^13^C NMR), and were internally referenced to residual protio solvent signals or TMS (note: CDCl_3_ referenced at δ 7.26 and 77.0 ppm, respectively, TMS referenced at δ 0 and 0 ppm respectively). Data for ^1^H NMR are reported as follows: chemical shift (δ ppm), multiplicity (s = singlet, d = doublet, t = triplet, q = quartet, m = multiplet, br = broad, dd = doublet of doublets, ddd = doublet of doublet of doublets, td = triplet of doublets), coupling constant (Hz), and integration. Data for ^13^C NMR are reported in terms of chemical shifts (δ ppm). IR spectra were recorded on a Perkin-Elmer (Boston, MA, USA) Spectrum 100 FTIR spectrometer and are reported in terms of frequency of absorption (cm^–1^). High-resolution mass spectra (HRMS) were obtained on a JEOL JMS-T100TD and are reported as *m*/*z* (M + H^+^, relative intensity). Melting points were measured on a Yanaco (Kyoto, Japan) micro melting point apparatus without correlation.

### 3.2. General Procedure for ATRA/ATRC Reaction

A Pyrex^®^ test tube from Corning (Tokyo, Japan) (12.5 cm × 1.6 cm) containing a mixture of alkyne **1** or alkene **4** (1.0 equiv, 0.1 mmol), α-halogencarbonyl **2** (3.0 equiv, 0.3 mmol) and *N*,*N*-diisopropylethylamine (1.0 equiv, 0.1 mmol) in ethyl acetate (0.75 mL) was degassed via FPT cycling three times and backfilled with Ar. The tube was placed ca. 0.5 cm from 3 W 420 nm LED. The resulting solution was stirred at ambient temperature for 20 h. The residue was concentrated in vacuo. The resulting mixture was purified by flash column chromatography on silica gel to yield product **3**, **5** or **6**.

## 4. Conclusions

In summary, we have demonstrated that an in situ formed halogen-bonding complex between alkynes or alkenes and DIPEA has the potential to induce ATRC reactions under visible-light irradiation. Importantly, this transformation provides a new pathway for the formation of two Csp^3^−Csp^2^ bonds and one Csp^3^−X bond in one step, highlighting the step-economics of this protocol. This method is highly efficient, and a wide range of functional groups were well-tolerated under mild reaction conditions. Moreover, the substrate activation through the halogen bonding is a novel activation method that can be used for challenging synthetic routes, since halogenbonding acceptors such as tertiary amines are relatively inexpensive and readily available. Further efforts are focused on extending this new methodology to other classes of compounds, including aryl halides with amines or phenols.

## Figures and Tables

**Table 1:**
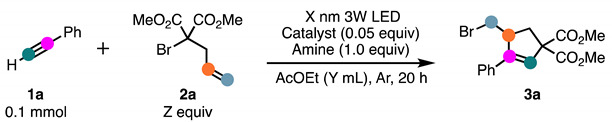
Optimization of reaction conditions.

Entry	Catalyst	Amine	X (nm)	Y (mL)	Z (equiv)	Yield (%) *^a^*
1	4-Ph-pyridine		380	1.0	2.5	53
2	4-Ph-pyridine		400	1.0	2.5	32
3	4-Ph-pyridine		420	1.0	2.5	57 (50)
4	4-Ph-pyridine		450	1.0	2.5	0
5	4-Ph-pyridine		420	2.0	2.5	0
6	4-Ph-pyridine		420	0.75	2.5	60 (62)
7	4-Ph-pyridine		420	0.25	2.5	47
8	4-Ph-pyridine	DIPEA	420	0.75	2.5	79 (71)
9		DIPEA	420	0.75	2.5	89 (75)
10		DIPEA	420	0.75	2.0	69
11		DIPEA	420	0.75	3.0	88 (79)

*^a^* ^1^H NMR yields. Numbers in parentheses are isolated yields.

## Data Availability

Data are contained within the article or Supplementary Materials.

## Supplementary Materials

The following are available online, Table S1: Optimization of catalyst; Table S2: Optimization of Amines; Table S3: Screening of wavelength; Table S4: Screening of solvent; Table S5: Screening of wavelength without amine; Table S6: Attempted alkynes for ATRA/ATRC reaction; Table S7: Attempted alkenes for ATRA/ATRC reaction. Charts of NMR spectra (^1^H, ^13^C) and characterization data for all compounds, are available online.

## Sample Availability

Samples of the compounds are available from the authors.

## References

[1] Vince R., Hua M. (1990). Synthesis and anti-HIV Activity of Carbocyclic 2,3-didehydro-2,3-dideoxy 2,6-disubstituted Purine Nucleosides. J. Med. Chem..

[2] Bisacchi G.S., Chao S.T., Bachard C., Daris J.P., Innaimo S., Jacobs G.A., Kocy O., Lapointe P., Martel A., Merchant Z. (1997). BMS-200475, A Novel Carbocyclic 2-deoxyguanosine analog with Potent and Selective Anti-hepatitis B Virus Qctivity in vitro. Biol. Med. Chem. Lett..

[3] Meijere A., Kozhushkov S.I., Schill H. (2006). Three-Membered-Ring-Based Molecular Architectures. Chem. Rev..

[4] Bowman W.R., Bridge C.F., Brookes P. (2000). Synthesis of Heterocycles by Radical Cyclisation. J. Chem. Soc. Perkin Trans..

[5] Kamigaito M., Ando T., Sawamoto M. (2001). Metal-Catalyzed Living Radical Polymerization. Chem. Rev..

[6] Kharasch M.S., Jensen E.V., Urry W.H. (1945). Addition of Carbon Tetrachloride and Chloroform to Olefines. Science.

[7] Kharasch M.S., Skell P.S., Fisher P. (1948). Reactions of Atoms and Free Radicals in Solution. XII. The Addition of Bromo Esters to Olefins. J. Am. Chem. Soc..

[8] Clark A.J. (2002). Atom Transfer Radical Cyclisation Reactions Mediated by Copper Complexes. Chem. Soc. Rev..

[9] Pintauer T., Matyjaszewski K. (2008). Atom Transfer Radical Addition and Polymerization Reactions Catalyzed by ppm Amounts of Copper Complexes. Chem. Soc. Rev..

[10] Nagashima H., Wakamatsu H., Itoh K., Tomo Y., Tsuji J. (1983). New Regio and Stereoselective Preparation of Trichlorinated γ-Butyrolactones by Copper Catalyzed Cyclization of Allyl Trichloroacetates. Tetrahedron Lett..

[11] Lee G.M., Parvez M., Weinreb S.M. (1988). Intramolecular Metal Catalyzed Kharasch Cyclizations of Olefinic α-Halo Esters and Acids. Tetrahedron.

[12] Quayle P., Fengas D., Richards S. (2003). Atom Transfer Radical Cyclisations Mediated by the Grubbs Ruthenium Metathesis Catalyst. Synlett.

[13] Monks B.M., Cook S.P. (2013). Palladium-catalyzed Intramolecular Iodine-transfer Reactions in the Presence of β-Hydrogen Atoms. Angew. Chem. Int. Ed..

[14] Boivin J., Yousfi M., Zard S.Z. (1994). A Versatile Radical Based Synthesis of γ–Lactams using Nickel Powder/Acetic acid. Tetrahedron Lett..

[15] Bag D., Kour H., Sawant S.D. (2021). Photo-induced 1,2-carbohalofunctionalization of C-C Multiple Bonds via ATRA Pathway. Org. Biomol. Chem..

[16] Wallentin C.J., Nguyen J.D., Finkbeiner P., Stephenson C.R.J. (2012). Visible Light-Mediated Atom Transfer Radical Addition via Oxidative and Reductive Quenching of Photocatalysts. J. Am. Chem. Soc..

[17] Shen Y., Cornella J., Julia-Hernandez F., Martin R. (2017). Visible-Light-Promoted Atom Transfer Radical Cyclization of Unactivated Alkyl Iodides. ACS Catal..

[18] Cheng J., Cheng Y., Xie J., Zhu C. (2017). Photoredox Divergent 1,2-Difunctionalization of Alkenes with *gem*-Dibromides. Org. Lett..

[19] Zhao Q., Xu G., Xu J., Wang Z., Xu P. (2019). A lutidine-promoted Photoredox Catalytic Atom-transfer Radical Cyclization Reaction for the Synthesis of 4-Bromo-3,3-dialkyl-octahydro-indol-2-ones. Chem. Commun..

[20] Hou H., Tang D., Li H., Xu Y., Yan C., Shi Y., Chen X., Zhu S. (2019). Visible-Light-Driven Chlorotrifluoromethylative and Chlorotrichloromethylative Cyclizations of Enynes. J. Org. Chem..

[21] Hou H., Xu Y., Yang H., Chen X., Yan C., Shi Y., Zhu S. (2020). Visible-Light Mediated Hydrosilylative and Hydrophosphorylative Cyclizations of Enynes and Dienes. Org. Lett..

[22] Matsuo K., Yamaguchi E., Itoh A. (2020). In Situ-Generated Halogen-Bonding Complex Enables Atom Transfer Radical Addition (ATRA) Reactions of Olefins. J. Org. Chem..

[23] Matsuo K., Kondo T., Yamaguchi E., Itoh A. (2021). Photoinduced Atom Transfer Radical Addition Reaction of Olefins with α-Bromo Carbonyls. Chem. Pharm. Bull.

[B24-molecules-26-06781] 24.The reaction temperature rose to about 30 °C after 20 h of LED light irradiation.

[B25-molecules-26-06781] 25.At low concentrations, hydrogen radicals were abstracted from the amine or solvent before the intermolecular addition (ATRA) reaction proceeded, and the hydrogenated form of the malonate ester was preferentially formed. (Table 1, entry 5. 88% hydrogenated malonate was recovered.)

[26] Wilkinson F., Abdel-Shafi A.A. (1997). Mechanism of Quenching of Triplet States by Oxygen: Biphenyl Derivatives in Acetonitrile. J. Phys. Chem. A.

[27] Sylla M., Joseph D., Chevallier E., Camara C., Dumas F. (2006). A Simple and Direct Access to Ethylidene Malonates. Synthesis.

[28] Gui Q., Hu L., Chen X., Liu J., Tan Z. (2015). Stereoselective Synthesis of Vinylphosphonates and Phosphine Oxides via Silver-catalyzed Phosphorylation of Styrenes. Chem. Commun..

