# Supplementary material for: Photoinduced Atom Transfer Radical Addition/Cyclization Reaction between Alkynes or Alkenes with Unsaturated α-Halogenated Carbonyls
Source: Molecules. 2021 Nov 10;26(22):6781. doi: 10.3390/molecules26226781 (PMC8618827; doi:10.3390/molecules26226781)

Supporting Information

Photoinduced Atom Transfer Radical Addition/Cyclization Reaction Between Alkynes or Alkenes with Unsaturated α-Halogenated Carbonyls

Kazuki Matsuo, Tadashi Yoshitake, Eiij Yamaguchi, Akichika Itoh
Laboratory of pharmaceutical synthetic chemistry, Gifu Pharmaceutical University
1-25-4, Daigaku-nishi, Gifu 501-1196, Japan
yamaguchi@gifu-pu.ac.jp; itoha@gifu-pu.ac.jp

Table of contents

1. **Optimization of reaction conditions** S1
   Table S1. Optimization of catalyst
   Table S2. Optimization of Amines
   Table S3. Screening of wavelength
   Table S4. Screening of solvent
   Table S5. Screening of wavelength without amine
2. **Substrate scope** S6
   Table S6 Attempted alkynes for ATRA/ATRC reaction
   Table S7 Attempted alkenes for ATRA/ATRC reaction
3. **Radical trapping experiment** S7
4. **Synthesis of cyclic compounds 3** S8
5. **Synthesis of cyclic compounds 5** S19
6. **Synthesis of cyclic compounds 6** S26
7. **References** S29
8. **^{1}H NMR, ^{13}C NMR, ^{19}F and DEPT-135 NMR Spectra of 3** S30
9. **^{1}H NMR, ^{13}C NMR, ^{19}F and DEPT-135 NMR Spectra of 5** S57
10. **^{1}H NMR, ^{13}C NMR, ^{19}F and DEPT-135 NMR Spectra of 6** S70
11. **^{1}H-^{1}H NOESY spectrum of 6h** S80
12. **^{1}H NMR and ^{13}C NMR spectra of 7** S81

1. Optimization of reaction conditions

Table S1. Optimization of catalyst

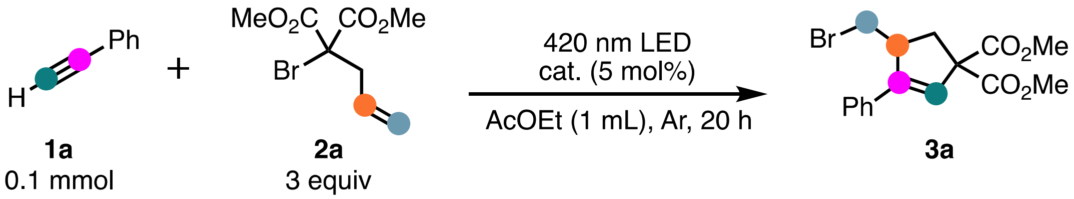

entry	cat	**3a** (%)
1	4-CN-pyridine	33
2	4-NH_2-pyridine	no reaction
3	4-Ac-pyridine	18
4	3-CN-pyridine	17
5	2-CN-pyridine	no reaction
6	2,3-diCN-pyridine	no reaction
7	DMAP	no reaction
8	2-F-pyridine	no reaction
9	2-Cl-pyridine	no reaction
10	2-Br-pyridine	no reaction
11	3-Br-pyridine	20
12	2-CHO-pyridine	no reaction
13	4-Et-pyridine	45
14	2-Et-pyridine	no reaction
15	2-NH_2CH_2-pyridine	no reaction
16	2-NH_2-6-MeO-pyridine	no reaction

[a] ^{1}H NMR yields.

Table S2. Optimization of Amines

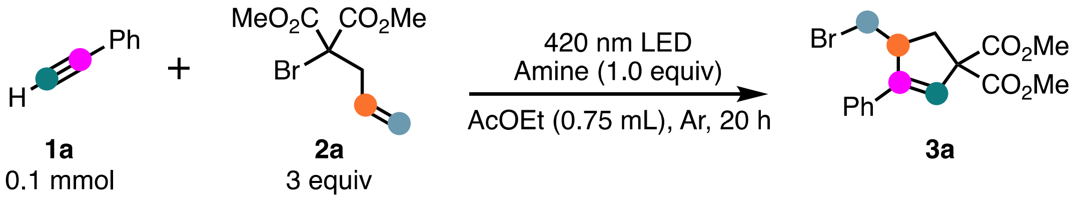

entry	amines	**3** (%)
1	benzyl amine	11
2	pyrrolidine	56
3	piperidine	35
4	diethylamine	51
5	butylamine	no reaction
6	DIPEA	88

[a] ^{1}H NMR yields.

Table S3. Screening of wavelength

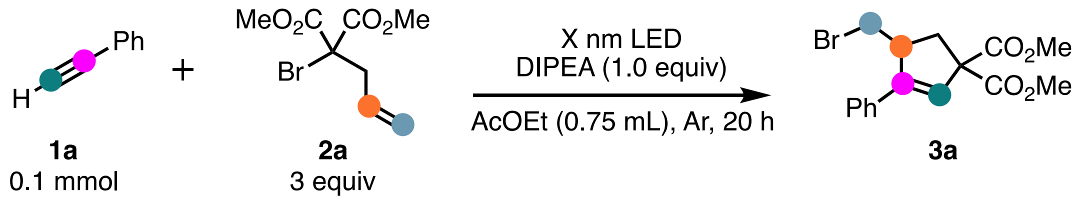

entry	X (nm)	**3** (%)
1	370	68
2	380	70
3	400	78
4	420	88
5	450	81
6	470	70
7	500	trace

[a] ^{1}H NMR yields.

Table S4. Screening of solvent

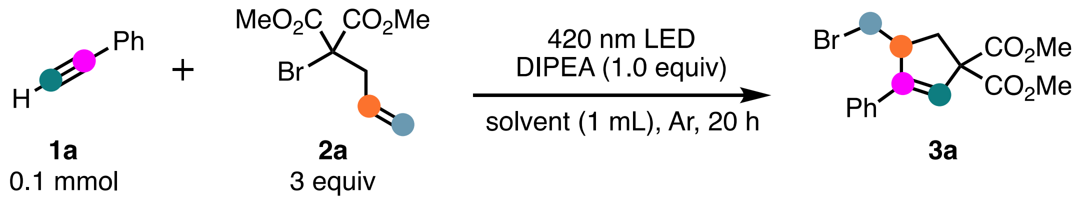

entry	solvent	**3a** (%)
1	IPA	40
2	tBuOH	17
3	THF	8
4	DMF	29
5	DMA	48
6	DMSO	39
7	water	46
8	hexane	0
9	toluene	0
10	EtOAc	50
11	$CHCl_3$	1
12	DCM	6
13	MeOH	2
14	Et_2O	23
15	MeCN	0
16	Acetone	34

[a] ^{1}H NMR yields.

Table S5. Screening of wavelength without amine

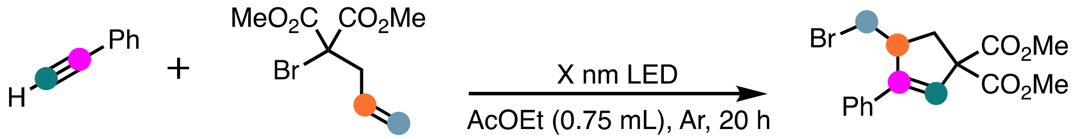

1a 0.1 mmol + **2a** 3 equiv → **3a**

entry	X (nm)	**3** (%)
1	380	33
2	400	trace
3	420	no reaction
4	450	no reaction
5	470	no reaction
6	500	no reaction

[a] ^{1}H NMR yields.

2. Substrate scope

Table S6. Attempted alkynes for ATRA/ATRC reaction

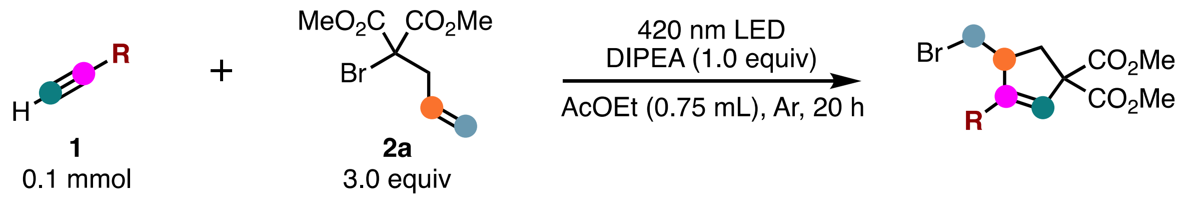

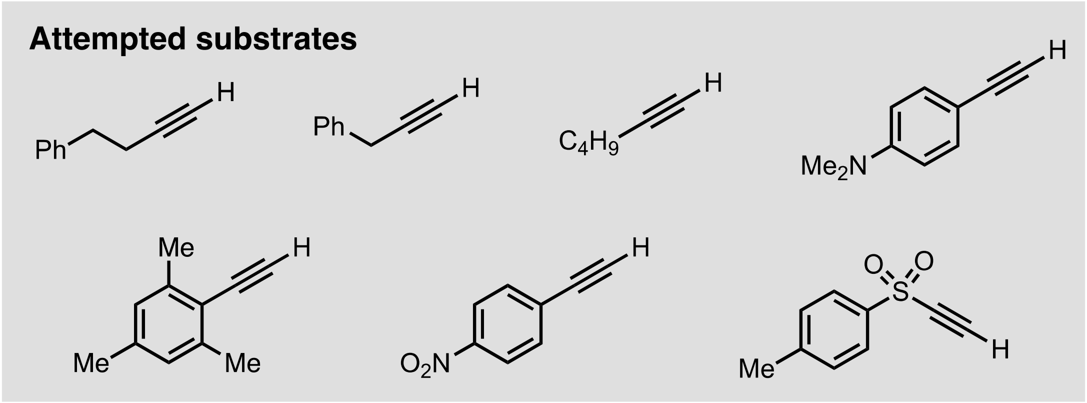

* The reaction using these substrate did not proceed.

Table S7. Attempted alkenes for ATRA/ATRC reaction

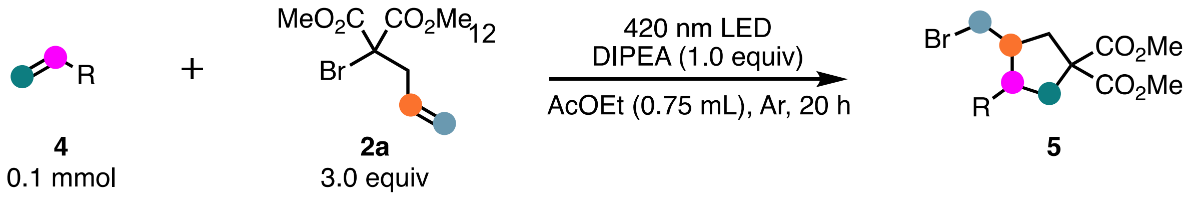

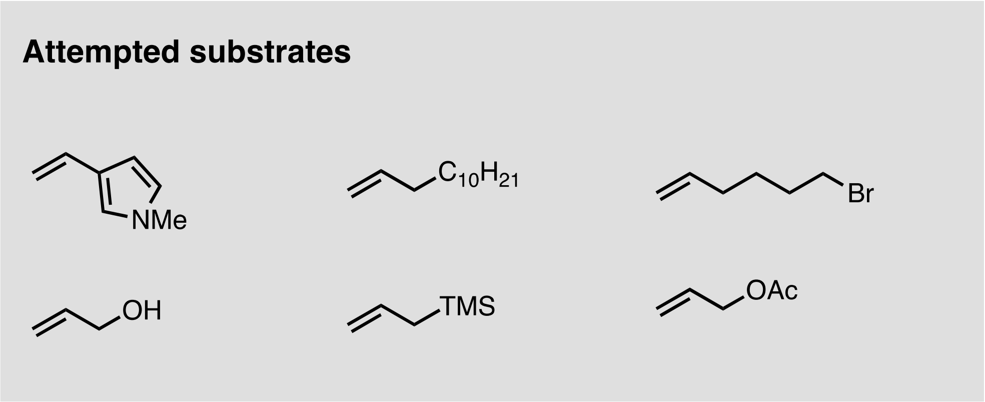

* The reaction using these substrate did not proceed.

3. Radical trapping experiment

Another radical scavenging experiment was performed. When BHT (1.0 equiv) was added as a radical scavenger, the yield of the desired product (**3a**) was slightly reduced and a small amount of product (**7b**) with trapped malonic acid radical was obtained.

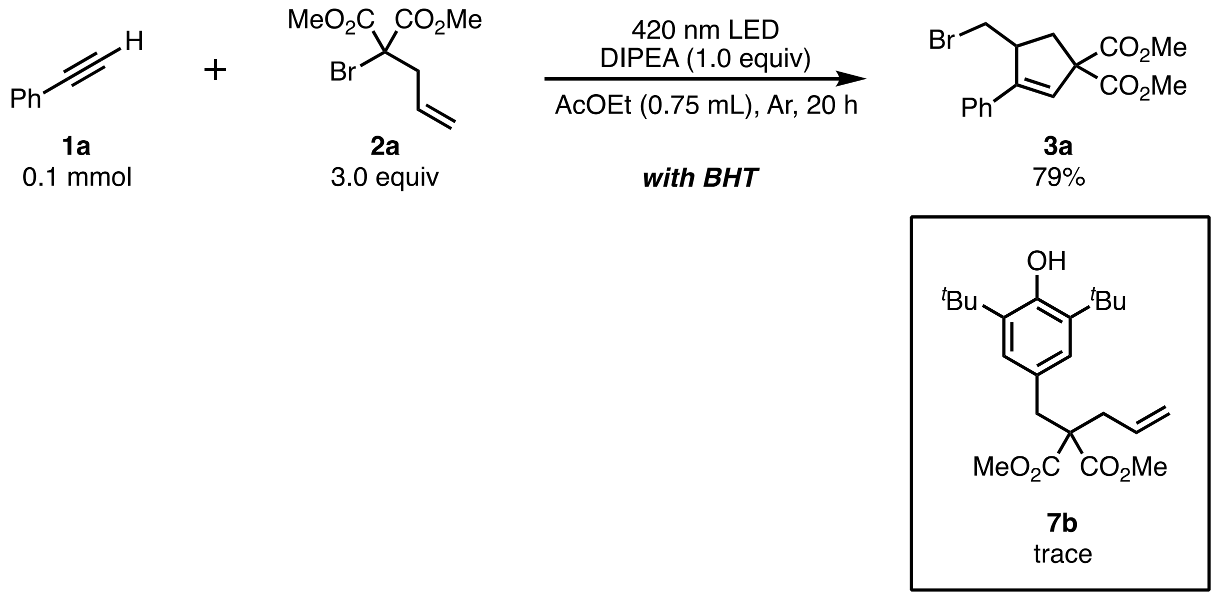

4. Synthesis of cyclic compound 3

Dimethyl 4-(bromomethyl)-3-phenylcyclopent-2-ene-1,1-dicarboxylate (**3a**)

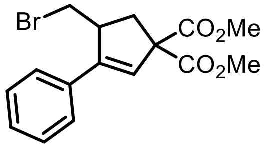

79 %, 28.0 mg (Colorless oil)

TLC (SiO_2) R_f = 0.40 (*n*-hexane : EtOAc = 4 : 1)

^{1}H NMR (500 MHz, $CDCl_3$) δ 7.42-7.32 (m, 5H), 6.19 (s, 1H), 3.80-3.75 (m, 7H), 3.59 (dd, *J* = 10.3, 2.9 Hz, 1H), 3.31 (dd, *J* = 10.3, 9.2 Hz, 1H), 2.93 (dd, *J* = 13.8, 8.0 Hz, 1H), 2.63 (dd, *J* = 13.8, 4.0 Hz, 1H).

^{13}C {^{1}H} NMR (125 MHz, $CDCl_3$) δ 171.26, 171.20, 147.8, 133.7, 128.8, 128.5, 126.6, 125.8, 65.1, 53.0, 47.4, 36.7, 36.4.

FTIR (ATR) 2954, 2924, 2851, 1734, 1599, 1576, 1496, 1447, 1434, 1247, 1205, 1141, 1080, 1047, 1013, 947, 849, 766, 697 cm^{-1}.

HRMS m/z (DART) calcd for $C_{16}H_{18}BrO_4^+$ $(M+H)^+$, 353.0383; found, 353.0390.

Dimethyl 4-(bromomethyl)-3-(*p*-tolyl)cyclopent-2-ene-1,1-dicarboxylate (**3b**)[1]

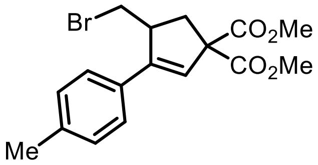

84 %, 31.0 mg (Colorless oil)

TLC (SiO_2) R_f = 0.40 (*n*-hexane : EtOAc = 4 : 1)

^{1}H NMR (500 MHz, $CDCl_3$) δ 7.30 (d, *J* = 8.0 Hz, 2H), 7.17 (d, *J* = 8.0 Hz, 2H), 6.14 (d, *J* = 1.1 Hz, 1H), 3.80-3.74 (m ,7H), 3.59 (dd, *J* = 10.3, 2.9 Hz, 1H), 3.30 (t, *J* = 9.7 Hz, 1H), 2.91 (dd, *J* = 13.8, 8.0 Hz, 1H), 2.64 (dd, *J* = 13.8, 4.0 Hz, 1H), 2.36 (s, 3H).

^{13}C {^{1}H} NMR (125 MHz, $CDCl_3$) δ 171.4, 171.3, 147.7, 138.5, 130.8, 129.4, 126.5, 124.8, 65.0, 52.98, 52.95, 47.4, 36.7, 36.6, 21.2.

Dimethyl 4-(bromomethyl)-3-(*m*-tolyl)cyclopent-2-ene-1,1-dicarboxylate (**3c**)

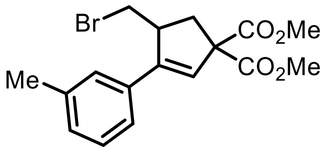

65 %, 23.8 mg (Colorless oil)

TLC (SiO$_2$) R_f = 0.40 (*n*-hexane : EtOAc = 4 : 1)

^{1}H NMR (500 MHz, CDCl$_3$) δ 7.27-7.22 (m, 2H), 7.19 (d, J = 7.5 Hz, 1H), 7.13 (d, J = 7.5 Hz, 1H), 6.16 (d, J = 1.2 Hz, 1H), 3.80-3.74 (m ,7H), 3.60 (dd, J = 10.3, 2.9 Hz, 1H), 3.30 (t, J = 9.7 Hz, 1H), 2.93 (dd, J = 13.8, 8.6 Hz, 1H), 2.63 (dd, J = 13.8, 4.0 Hz, 1H), 2.26 (s, 3H).

^{13}C {^{1}H} NMR (125 MHz, CDCl$_3$) δ 171.3, 171.2, 147.9, 138.4, 133.7, 129.3, 128.6, 127.2, 125.6, 123.6, 65.0, 52.98, 52.95, 47.4, 36.7, 36.6, 21.4.

FTIR (ATR) 2954, 1735, 1435, 1251, 1141, 1081, 846, 788, 701 cm^{-1}.

HRMS m/z (DART) calcd for $C_{17}H_{20}BrO_4^+$ (M+H)$^+$, 367.0540 ; found, 367.0542.

Dimethyl 4-(bromomethyl)-3-(4-(*tert*-butyl)phenyl)cyclopent-2-ene-1,1-dicarboxylate (**3d**)

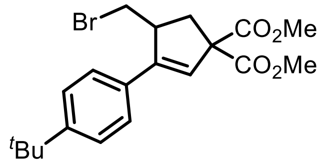

81 %, 33.1 mg (Colorless oil)

TLC (SiO$_2$) R_f = 0.40 (*n*-hexane : EtOAc = 4 : 1)

^{1}H NMR (500 MHz, CDCl$_3$) δ 7.39-7.33 (m, 4H), 6.15 (d, J = 1.1 Hz, 1H), 3.83-3.73 (m, 7H), 3.62 (dd, J = 10.3, 2.9 Hz, 1H), 3.30 (t, J = 9.7 Hz, 1H), 2.91 (dd, J = 13.8, 8.0 Hz, 1H), 2.63 (dd, J = 13.8, 4.0 Hz, 1H), 1.32 (s, 9H).

^{13}C {^{1}H} NMR (125 MHz, CDCl$_3$) δ 171.4, 171.3, 151.7, 147.5, 130.8, 126.2, 125.7, 124.9, 65.0, 52.96, 52.92, 47.4, 36.71, 36.66, 34.6, 31.2.

FTIR (ATR) 3484, 2957, 2869, 1734, 1608, 1513, 1434, 1407, 1365, 1250, 1205, 1142, 1080, 1022, 948, 838, 744, 671 cm^{-1}.

HRMS m/z (DART) calcd for $C_{20}H_{26}BrO_4^+$ (M+H)$^+$, 409.1009; found, 409.1000.

Dimethyl 4-(bromomethyl)-3-(4-fluorophenyl)cyclopent-2-ene-1,1-dicarboxylate (**3e**)

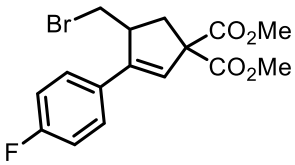

78 %, 28.8 mg (White solid)

TLC (SiO_2) R_f = 0.40 (*n*-hexane : EtOAc = 4 : 1)

^{1}H NMR (500 MHz, $CDCl_3$) δ 7.39-7.37 (m, 2H), 7.08-7.04 (m, 2H), 6.13 (s, 1H), 3.80-3.75 (m ,7H), 3.54 (d, *J* = 9.2 Hz, 1H), 3.30 (dd, *J* = 9.7, 9.2 Hz, 1H), 2.92 (dd, *J* = 14.3, 8.6 Hz, 1H), 2.63 (dd, *J* = 14.3, 3.4 Hz, 1H).

^{13}C {^{1}H} NMR (125 MHz, $CDCl_3$) δ 171.2, 171.1, 162.7 (d, $J_{C\text{–}F}$ = 249.5 Hz), 146.7, 129.9, 128.3 (d, $J_{C\text{–}F}$ = 7.2 Hz), 125.7, 115.7 (d, $J_{C\text{–}F}$ = 21.6 Hz), 65.0, 53.0, 47.4, 36.7, 36.2.

^{19}F NMR (475 MHz, $CDCl_3$) δ -112.5.

FTIR (ATR) 3002, 2954, 2923, 2850, 2031, 1732, 1602, 1509, 1434, 1339, 1298, 1236, 1206, 1161, 1141, 1080, 1047, 1013, 947, 834, 756, 719, 667 cm^{-1}.

HRMS m/z (DART) calcd for $C_{16}H_{17}BrFO_4^+$ $(M+H)^+$, 371.0289; found, 371.0276.

m.p. 89.7-91.0 °C.

Dimethyl 4-(bromomethyl)-3-(4-chlorophenyl)cyclopent-2-ene-1,1-dicarboxylate (**3f**)

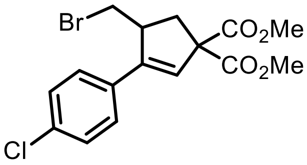

57 %, 22.1 mg (White solid)

TLC (SiO_2) R_f = 0.30 (*n*-hexane : EtOAc = 4 : 1)

^{1}H NMR (500 MHz, $CDCl_3$) δ 7.39-7.34 (m, 4H), 6.18 (d, *J* = 1.7 Hz, 1H), 3.80-3.74 (m, 7H), 3.53 (dd, *J* = 10.3, 2.9 Hz, 1H), 3.30 (dd, *J* = 10.3, 9.2 Hz, 1H), 2.90 (dd, *J* = 14.3, 8.6 Hz, 1H), 2.63 (dd, *J* = 14.3, 4.0 Hz, 1H).

^{13}C {^{1}H} NMR (125 MHz, $CDCl_3$) δ 171.1, 171.0, 146.7, 134.3, 132.2, 128.9, 127.9, 126.4, 65.0, 53.0, 47.3, 36.6, 36.0.

FTIR (ATR) 3631, 3597, 3579, 3023, 2955, 2923, 2852, 1979, 1890, 1731, 1595, 1492, 1434, 1403, 1339, 1297, 1236, 1216, 1141, 1093, 1080, 1046, 1013, 973, 946, 929, 908, 894, 857, 826 cm^{-1}.

HRMS m/z (DART) calcd for $C_{16}H_{17}BrClO_4^+$ $(M+H)^+$, 386.9993; found, 387.0008.
m.p. 74.0-75.3 ºC.

Dimethyl 4-(bromomethyl)-3-(3-chlorophenyl)cyclopent-2-ene-1,1-dicarboxylate (**3g**)

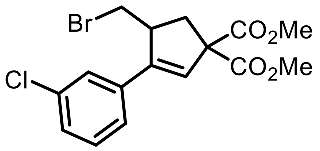

35 %, 13.7 mg (Colorless oil)
TLC (SiO_2) R_f = 0.40 (*n*-hexane : EtOAc = 4 : 1)
^{1}H NMR (500 MHz, $CDCl_3$) δ 7.39 (s ,1H), 7.30-7.26 (m, 3H), 6.20 (d, J = 1.7 Hz, 1H), 3.80-3.74 (m, 7H), 3.54 (dd, J = 10.3, 2.9 Hz, 1H), 3.31 (dd, J = 10.3, 9.2 Hz, 1H), 2.92 (dd, J = 14.3, 8.6 Hz, 1H), 2.63 (dd, J = 14.3, 4.6 Hz, 1H).
^{13}C {^{1}H} NMR (125 MHz, $CDCl_3$) δ 171.0, 170.9, 146.6, 135.7, 134.7, 130.0, 128.5, 127.3, 126.7, 124.7, 65.1, 53.1, 47.2, 36.6, 36.0yo3.
FTIR (ATR) 2954, 2923, 2852, 1734, 1594, 1564, 1434, 1248, 1206, 1143, 1079, 1048, 1019, 948, 849, 788, 734, 695, 671 cm^{-1}.
HRMS m/z (DART) calcd for $C_{16}H_{17}BrClO_4^+$ $(M+H)^+$, 386.9993; found, 386.9985.

Dimethyl 4-(bromomethyl)-3-(2-chlorophenyl)cyclopent-2-ene-1,1-dicarboxylate (**3h**)

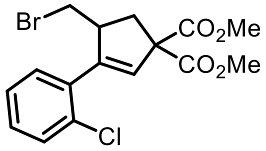

54 %, 20.9 mg (Colorless oil)
TLC (SiO_2) R_f = 0.30 (*n*-hexane : EtOAc = 4 : 1)
^{1}H NMR (500 MHz, $CDCl_3$) δ 7.39-7.25 (m, 4H), 6.07 (s, 1H), 4.02-3.99 (m, 1H), 3.81-3.76 (m, 6H), 3.40 (dd, J = 10.3, 2.9 Hz, 1H), 3.27 (dd, J = 9.7, 9.2 Hz, 1H), 2.98 (dd, J = 13.8, 8.0 Hz, 1H), 2.47 (dd, J = 13.8, 6.3 Hz, 1H).
^{13}C {^{1}H} NMR (125 MHz, $CDCl_3$) δ 171.1, 170.8, 147.1, 133.7, 132.3, 131.0, 130.4, 129.9, 129.4, 126.8, 65.0, 53.0, 52.9, 48.1, 36.7, 36.3.
FTIR (ATR) 3020, 2874, 2401, 1732, 1436, 1215, 1141, 929, 852, 750, 669 cm^{-1}.
HRMS m/z (DART) calcd for $C_{16}H_{17}BrClO_4^+$ $(M+H)^+$, 386.9993; found, 386.9988.

Dimethyl 4-(bromomethyl)-3-(4-bromophenyl)cyclopent-2-ene-1,1-dicarboxylate (**3i**)[1]

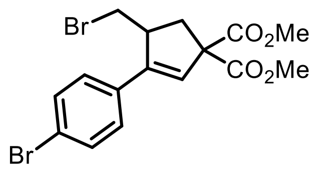

71 %, 30.7 mg (Colorless oil)

TLC (SiO_2) R_f = 0.40 (*n*-hexane : EtOAc = 4 : 1)

^{1}H NMR (500 MHz, $CDCl_3$) δ 7.49 (d, J = 8.0 Hz, 2H), 7.27 (d, J = 8.0 Hz, 2H), 6.19 (s, 1H), 3.80-3.73 (m, 7H), 3.53 (dd, J = 9.7, 2.9 Hz, 1H), 3.30 (t, J = 9.7 Hz, 1H), 2.92 (dd, J = 14.3, 8.6 Hz, 1H), 2.62 (dd, J = 14.3, 4.0 Hz, 1H).

^{13}C {^{1}H} NMR (125 MHz, $CDCl_3$) δ 171.02, 170.99, 146.7, 132.7, 131.9, 128.2, 126.6, 122.5, 65.1, 53.0, 47.2, 36.6, 36.0.

Dimethyl 4-(bromomethyl)-3-(4-(trifluoromethyl)phenyl)cyclopent-2-ene-1,1-dicarboxylate (**3j**)

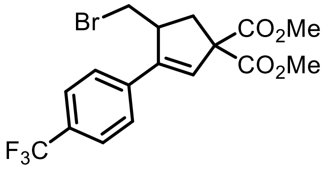

51 %, 21.3 mg (White solid)

TLC (SiO_2): R_f = 0.30 (*n*-hexane : EtOAc = 4 : 1)

^{1}H NMR (500 MHz, $CDCl_3$) δ 7.62 (d, J = 8.0 Hz, 2H), 7.51 (d, J = 8.0 Hz, 2H), 6.28 (s, 1H), 3.85-3.74 (m ,7H), 3.53 (dd, J = 10.3, 2.3 Hz, 1H), 3.32 (dd, J = 9.7, 9.2 Hz, 1H), 2.97 (dd, J = 14.3, 8.6 Hz, 1H), 2.64 (dd, J = 14.3, 4.0 Hz, 1H).

^{13}C {^{1}H} NMR (125 MHz, $CDCl_3$) δ 170.89, 170.85, 146.6, 137.4, 130.4 (q, J_{C-F} = 31.8 Hz), 128.2, 126.9, 125.7 (q, J_{C-F} = 3.9 Hz), 123.9 (q, J_{C-F} = 272.6 Hz), 65.1, 53.1, 47.2, 36.6, 35.8.

^{19}F NMR (475 MHz, $CDCl_3$) δ -62.6.

FTIR (ATR) 2956, 2923, 2851, 1735, 1617, 1575, 1435, 1412, 1326, 1250, 1207, 1167, 1125, 1082, 1067, 1016, 948, 839, 684 cm^{-1}.

HRMS m/z (DART) calcd for $C_{17}H_{17}BrF_3O_4^+$ $(M+H)^+$, 421.0257; found, 421.0269.

m.p. 82.0-84.0 ^{o}C.

Dimethyl 4-(bromomethyl)-3-(3,5-bis(trifluoromethyl)phenyl)cyclopent-2-ene-1,1-dicarboxy-late (**3k**)

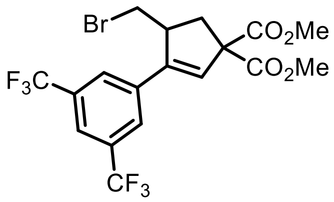

31 %, 15.4 mg (Colorless oil)

TLC (SiO_2): R_f = 0.40 (*n*-hexane : EtOAc = 4 : 1)

^{1}H NMR (500 MHz, $CDCl_3$) δ 7.83 (s, 1H), 7.81 (s, 2H), 6.34 (d, J = 1.7 Hz, 1H), 3.86-3.77 (m, 7H), 3.50 (dd, J = 10.3, 2.9 Hz, 1H), 3.35 (dd, J = 10.3, 9.5 Hz, 1H), 2.98 (dd, J = 14.3, 8.6 Hz, 1H), 2.66 (dd, J = 14.3, 5.2 Hz, 1H).

^{13}C {^{1}H} NMR (125 MHz, $CDCl_3$) δ 170.6, 145.4, 136.2, 132.2 (q, $J_{C–F}$ = 31.8 Hz), 130.0, 126.6, 123.1 (q, $J_{C–F}$ = 272.6 Hz), 122.1 (m), 65.2, 53.2, 47.0, 36.5, 35.3.

^{19}F NMR (475 MHz, $CDCl_3$) δ -62.8.

FTIR (ATR) 2930, 2255, 2020, 1734, 1436, 1384, 1280, 1184, 1143, 1088, 904, 846, 805, 726, 683 cm^{-1}.

HRMS m/z (DART) calcd for $C_{18}H_{16}BrF_6O_4^+$ $(M+H)^+$, 489.0131; found, 489.0148.

Dimethyl 4-(bromomethyl)-3-(4-cyanophenyl)cyclopent-2-ene-1,1-dicarboxylate (**3l**)

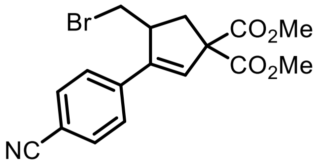

34 %, 13.0 mg (Colorless oil)

TLC (SiO_2): R_f = 0.10 (*n*-hexane : EtOAc = 4 : 1)

^{1}H NMR (500 MHz, $CDCl_3$) δ 7.67 (d, J = 8.0 Hz, 2H), 7.50 (d, J = 8.0 Hz, 2H), 6.31 (d, J = 1.7 Hz, 1H), 3.81-3.76 (m, 7H), 3.51 (dd, J = 10.3, 2.9 Hz, 1H), 3.32 (dd, J = 10.9, 8.6 Hz, 1H), 2.95 (dd, J = 14.3, 8.6 Hz, 1H), 2.64 (dd, J = 14.3, 4.6 Hz, 1H).

^{13}C {^{1}H} NMR (125 MHz, $CDCl_3$) δ 170.7, 146.3, 138.4, 132.6, 129.4, 127.2, 118.5, 112.0, 65.2, 53.2, 47.0, 36.5, 35.6.

FTIR (ATR) 2956, 2229, 1734, 1607, 1435, 1368, 1248, 1144, 1081, 838 cm^{-1}.

HRMS m/z (DART) calcd for $C_{17}H_{17}BrNO_4^+$ $(M+H)^+$, 378.0335; found, 378.0321.

Dimethyl 3-(4-acetylphenyl)-4-(bromomethyl)-cyclopent-2-ene-1,1-dicarboxylate (**3m**)

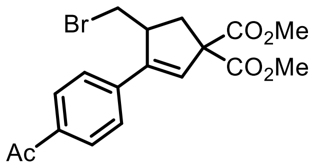

65 %, 25.5 mg (White solid)

TLC (SiO_2): R_f = 0.10 (*n*-hexane : EtOAc = 4 : 1)

^{1}H NMR (500 MHz, $CDCl_3$) δ 7.96 (d, *J* = 8.6 Hz, 2H), 7.50 (d, *J* = 8.6 Hz, 2H), 6.30 (d, *J* = 1.2 Hz, 1H), 3.81-3.74 (m, 7H), 3.54 (dd, *J* = 10.3, 2.9 Hz, 1H), 3.33 (dd, *J* = 10.3, 9.2 Hz, 1H), 2.95 (dd, *J* = 14.3, 8.6 Hz, 1H), 2.63 (dd, *J* = 14.3, 4.6 Hz, 1H), 2.61 (s, 3H).

^{13}C {^{1}H} NMR (125 MHz, $CDCl_3$) δ 197.4, 170.9, 170.8, 146.9, 138.4, 136.8, 128.8, 128.4, 126.7, 65.2, 53.1, 47.2, 36.6, 36.0, 26.6.

FTIR (ATR) 3003, 2955, 2923, 2852, 1733, 1682, 1604, 1561, 1434, 1407, 1359, 1258, 1206, 1143, 1074, 1048, 1014, 958, 834, 720, 668 cm^{-1}.

HRMS m/z (DART) calcd for $C_{18}H_{20}BrO_5^+$ $(M+H)^+$, 395.0489; found, 395.0507.

m.p. 116.0-118.7 ºC.

Dimethyl 4-(bromomethyl)-3-(4-(methoxycarbonyl)phenyl)cyclopent-2-ene-1,1-dicarboxylate (**3n**)

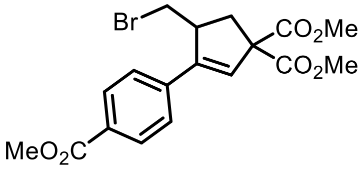

56 %, 23.2 mg (Colorless oil)

TLC (SiO_2) R_f = 0.20 (*n*-hexane : EtOAc = 4 : 1)

^{1}H NMR (500 MHz, $CDCl_3$) δ 8.03 (d, *J* = 8.0 Hz, 2H), 7.47 (d, *J* = 8.0 Hz, 2H), 6.30 (d, *J* = 1.7 Hz, 1H), 3.93 (s ,3H), 3.85-3.73 (m, 7H), 3.55 (dd, *J* = 10.3, 2.9 Hz, 1H), 3.32 (dd, *J* = 10.3, 9.2 Hz, 1H), 2.94 (dd, *J* = 14.3, 8.6 Hz, 1H), 2.64 (dd, *J* = 14.3, 4.6 Hz, 1H).

^{13}C {^{1}H} NMR (125 MHz, $CDCl_3$) δ 170.91, 170.89, 166.5, 147.0, 138.2, 130.0, 129.9, 128.0, 126.5, 65.2, 53.1, 52.2, 47.2, 36.6, 36.0.

FTIR (ATR) 3001, 2954, 2848, 1935, 1725, 1609, 1567, 1434, 1409, 1279, 1253, 1206, 1191, 1142, 1110, 1080, 1048, 1017, 966, 948, 847, 827, 774, 707, 666 cm^{-1}.

HRMS m/z (DART) calcd for $C_{18}H_{20}BrO_6^+$ $(M+H)^+$, 411.0438; found, 411.0455.

Dimethyl 3-(4-(acetamide)phenyl)-4-(bromomethyl)-cyclopent-2-ene-1,1-dicarboxylate (**3o**)

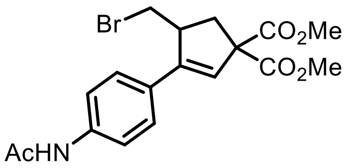

88 %, 36.0 mg (Colorless oil)

TLC (SiO_2) R_f = 0.20 (*n*-hexane : EtOAc = 1 : 1)

^{1}H NMR (500 MHz, $CDCl_3$) δ 7.74 (brs, 1H), 7.53 (d, *J* = 8.6 Hz, 2H), 7.33 (d, *J* = 8.6 Hz, 2H), 6.12 (d, *J* = 1.2 Hz, 1H), 3.80-3.73 (m, 7H), 3.55 (dd, *J* = 10.3, 2.9 Hz, 1H), 3.30 (dd, *J* = 9.7, 9.2 Hz, 1H), 2.89 (dd, *J* = 14.3, 8.6 Hz, 1H), 2.65 (dd, *J* = 14.3, 4.6 Hz, 1H), 2.19 (s, 3H).

^{13}C {^{1}H} NMR (125 MHz, $CDCl_3$) δ 171.3, 168.5, 147.1, 138.2, 129.4, 127.2, 124.8, 119.8, 65.1, 53.0, 47.3, 36.6, 36.4, 36.4, 24.5.

FTIR (ATR) 3308, 3182, 3108, 3014, 2956, 2848, 2433, 2028, 1729, 1673, 1595, 1520, 1434, 1408, 1371, 1318, 1296, 1253, 1217, 1142, 1080, 1045, 1010, 965, 947, 833, 753, 667 cm^{-1}.

HRMS m/z (DART) calcd for $C_{18}H_{21}BrNO_5^+$ $(M+H)^+$, 410.0598 ; found, 410.0578.

Dimethyl 3-(4-(N-*tert*-butoxycarbonyl)aminophenyl)-4-(bromomethyl)-cyclopent-2-ene-1,1-dicarboxylate (**3p**)

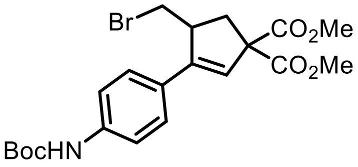

91 %, 42.8 mg (White solid)

TLC (SiO_2) R_f = 0.20 (*n*-hexane : EtOAc = 4 : 1)

^{1}H NMR (500 MHz, $CDCl_3$) δ 7.38-7.32 (m, 4H), 6.57 (brs, 1H), 6.11 (d, *J* = 1.7 Hz, 1H), 3.79-3.73 (m, 7H), 3.57 (dd, *J* = 10.3, 2.9 Hz, 1H), 3.30 (t, *J* = 9.7 Hz, 1H), 2.88 (dd, *J* = 14.3, 8.6 Hz, 1H), 2.64 (dd, *J* = 13.8, 4.0 Hz, 1H), 1.52 (s, 9H).

^{13}C {^{1}H} NMR (125 MHz, $CDCl_3$) δ 171.4, 171.3, 152.5, 147.1, 138.6, 128.3, 127.3, 124.4, 118.4, 80.8, 65.0, 53.0, 47.4, 36.6, 36.5, 28.3.

FTIR (ATR) 3436, 3020, 2957, 2929, 2401, 1729, 1611, 1586, 1520, 1502, 1435, 1410, 1317, 1215, 1156, 1082, 1053, 1028, 929, 903, 834, 749, 668 cm^{-1}.

HRMS m/z (DART): calcd for $C_{21}H_{27}BrNO_6^+$ $(M+H)^+$, 468.1017 ; found, 468.1001.

m.p. 52.7-55.7 ^oC.

Dimethyl-3-[(1,1'-biphenyl)-4-yl]4-(bromomethyl)-cyclopent-2-ene-1,1-dicarboxylate (**3q**)

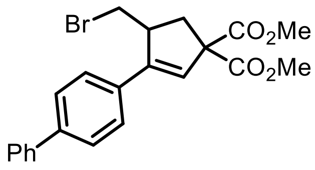

70 %, 30.0 mg (White solid)

TLC (SiO_2) R_f = 0.40 (*n*-hexane : EtOAc = 4 : 1)

^{1}H NMR (500 MHz, $CDCl_3$) δ 7.60-7.59 (m, 4H), 7.49-7.43 (m, 4H), 7.36 (t, J = 13.8 Hz, 1H), 6.24 (s, 1H), 3.81-3.75 (m, 7H), 3.63 (d, J = 9.2 Hz, 1H), 3.35 (dd, J = 9.7, 9.2 Hz, 1H), 2.94 (dd, J = 13.8, 8.6 Hz, 1H), 2.66 (dd, J = 14.3, 4.0 Hz, 1H).

^{13}C {^{1}H} NMR (125 MHz, $CDCl_3$): δ 171.25, 171.17, 147.3, 141.3, 140.3, 132.6, 128.8, 127.5, 127.4, 126.97, 126.95, 125.8, 65.1, 53.0, 47.4, 36.7, 36.4.

FTIR (ATR): 3030, 2953, 2924, 2853, 1732, 1599, 1580, 1510, 1488, 1433, 1405, 1339, 1300, 1247, 1205, 1141, 1080, 1046, 1007, 970, 836, 767, 731, 698, 670 cm^{-1}.

HRMS m/z (DART): calcd for $C_{22}H_{22}BrO_4^+$ $(M+H)^+$, 429.0696; found, 429.0714.

m.p. 205.3-210.0 ºC.

Dimethyl 4-(bromomethyl)-3-(naphthalen-2-yl)cyclopent-2-ene-1,1-dicarboxylate (**3r**)

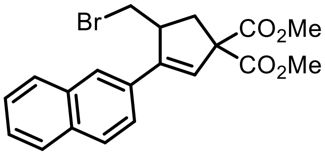

64 %, 25.9 mg (White solid)

TLC (SiO_2) R_f = 0.20 (*n*-hexane : EtOAc = 4 : 1)

^{1}H NMR (500 MHz, $CDCl_3$) δ 7.85-7.80 (m, 4H), 7.57 (dd, J = 6.9, 1.7 Hz, 1H), 7.50-7.48 (m, 2H), 6.32 (d, J = 1.2 Hz, 1H), 3.94-3.88 (m, 1H), 3.82 (s, 3H), 3.76 (s, 3H), 3.66 (dd, J = 10.3, 2.9 Hz, 1H), 3.38 (t, J = 9.7 Hz, 1H), 2.96 (dd, J = 13.8, 8.0 Hz, 1H), 2.70 (dd, J = 14.3, 4.0 Hz, 1H).

^{13}C {^{1}H} NMR (125 MHz, $CDCl_3$) δ 171.3, 171.2, 147.7, 133.25, 133.19, 131.1, 128.5, 128.2, 127.7, 126.6, 126.5, 126.3, 125.6, 124.5, 65.2, 53.05, 53.01, 47.5, 36.7, 36.5.

FTIR (ATR) 3650, 3021, 2954, 2925, 2852, 2345, 1731, 1626, 1597, 1504, 1434, 1356, 1249, 1216, 1140, 1078, 1046, 1020, 948, 930, 897, 845, 819, 751, 684, 688 cm^{-1}.

HRMS m/z (DART) calcd for $C_{20}H_{20}BrO_4^+$ $(M+H)^+$, 403.0539; found, 403.0535.

m.p. 92.3-94.3 ºC.

Dimethyl 4-(bromomethyl)-3-((6-methoxy)naphthalen-2-yl)cyclopent-2-ene-1,1-dicarboxy-late (**3s**)

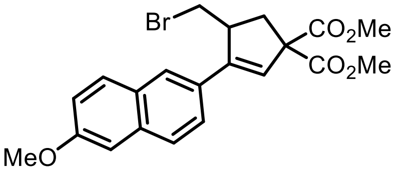

50 %, 21.5 mg (White solid)

TLC (SiO_2) R_f = 0.10 (*n*-hexane : EtOAc = 4 : 1)

^{1}H NMR (500 MHz, $CDCl_3$) δ 7.74-7.71 (m, 3H), 7.53 (dd, *J* = 6.9, 1.7 Hz, 1H), 7.16 (dd, *J* = 6.3, 2.3 Hz, 1H), 7.12 (d, *J* = 2.3 Hz, 1H), 6.27 (d, *J* = 1.7 Hz, 1H), 3.93 (s, 3H), 3.93-3.87 (m, 1H), 3.82 (s ,3H), 3.76 (s ,3H), 3.66 (dd, *J* = 10.3, 3.4 Hz, 1H), 3.37 (dd, *J* = 10.3, 9.7 Hz, 1H), 2.96 (dd, *J* = 14.3, 8.6 Hz, 1H), 2.70 (dd, *J* = 14.3, 4.0 Hz, 1H).

^{13}C {^{1}H} NMR (125 MHz, $CDCl_3$) δ 171.4, 171.3, 158.2, 147.7, 134.4, 129.7, 128.9, 128.6, 127.3, 125.4, 125.2, 125.0, 119.3, 105.7, 65.1, 55.3, 53.02, 52.97, 47.5, 36.7, 36.6.

FTIR (ATR) 3003, 2954, 2849, 1918, 1732, 1629, 1601, 1506, 1485, 1434, 1391, 1346, 1235, 1201, 1165, 1140, 1124, 1079, 1032, 947, 895, 848, 809, 756, 721, 699, 653 cm^{-1}.

HRMS m/z (DART) calcd for $C_{21}H_{22}BrIO_5^+$ $(M+H)^+$, 433.0645; found, 433.0667.

m.p. 139.7-141.7 ºC.

Dimethyl 4-(bromomethyl)-3-(pyridine-3-yl)cyclopent-2-ene-1,1-dicarboxylate (**3t**)

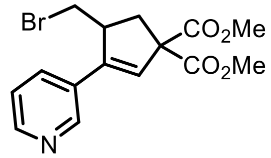

68 %, 24.1 mg (Colorless oil)

TLC (SiO_2) R_f = 0.20 (*n*-hexane : EtOAc = 4 : 1)

^{1}H NMR (500 MHz, $CDCl_3$) δ 8.67 (s, 1H), 8.57 (s, 1H), 7.71 (d, *J* = 8.0 Hz, 1H), 7.32 (brs, 1H), 6.27 (d, *J* = 1.8 Hz, 1H), 3.81-3.74 (m, 7H), 3.54 (dd, *J* = 10.3, 2.9 Hz, 1H), 3.34 (dd, *J* = 10.3, 8.0 Hz, 1H), 2.94 (dd, *J* = 14.3, 8.6 Hz, 1H), 2.65 (dd, *J* = 14.3, 4.6 Hz, 1H).

^{13}C {^{1}H} NMR (125 MHz, $CDCl_3$) δ 170.8, 149.5, 147.7, 144.8, 133.9, 129.7, 127.9, 123.6, 65.2, 53.1, 47.1, 36.5, 35.7.

FTIR (ATR) 3004, 2954, 2921, 2850, 1958, 1730, 1587, 1568, 1475, 1434, 1415, 1248, 1142, 1083, 1047, 1025, 1013, 969, 946, 852, 809, 756, 711, 666 cm^{-1}.

HRMS m/z (DART) calcd for $C_{15}H_{17}BrNO_4^+$ $(M+H)^+$, 354.0335; found, 354.0331.

Dimethyl 4-(bromomethyl)-3-(thiophen-3-yl)cyclopent-2-ene-1,1-dicarboxylate (**3u**)[1]

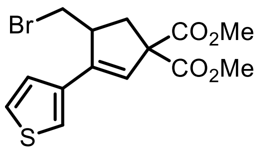

64 %, 22.8 mg (Colorless oil)

TLC (SiO_2) R_f = 0.40 (*n*-hexane : EtOAc = 4 : 1)

^{1}H NMR (500 MHz, $CDCl_3$) δ 7.32 (dd, *J* = 2.9, 2.3 Hz, 1H), 7.26 (s, 1H), 7.22-7.21 (m, 1H), 6.12 (d, *J* = 1.2 Hz, 1H), 3.79 (s ,3H), 3.75 (s ,3H), 3.68-3.64 (m ,2H), 3.36 (t, *J* = 9.7 Hz, 1H), 2.84 (dd, *J* = 14.3, 9.2 Hz, 1H), 2.68 (dd, *J* = 14.3, 3.4 Hz, 1H).

^{13}C {^{1}H} NMR (125 MHz, $CDCl_3$) δ 171.3, 171.2, 142.6, 135.4, 126.4, 126.2, 124.8, 122.4, 65.0, 53.01, 52.98, 48.5, 36.7, 36.5.

5. Synthesis of cyclic compounds 5

Dimethyl 4-(bromomethyl)-3-(*p*-tolyl)-1,1-cyclopentanedicarboxylate (**5a**)

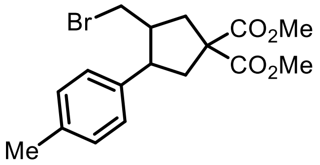

47 %, 17.3 mg, 59 : 41 d.r. (Colorless oil)

TLC (SiO_2) R_f = 0.40 (*n*-hexane : EtOAc = 4 : 1)

^{1}H NMR (500 MHz, $CDCl_3$) δ 7.13-7.06 (m, 4H), 3.80-3.77 (m, 6H), 3.49-3.39 (m, 1H), 3.21 (dd, J = 10.4, 7.5 Hz, 0.41H), 2.95-2.86 (m, 1.50H), 2.78-2.70 (m, 2.49H), 2.58 (dd, J = 13.8, 10.9 Hz, 0.56H), 2.40-2.32 (m, 4.43H), 2.24 (dd, J = 13.8, 10.9 Hz, 0.41H).

^{13}C {^{1}H} NMR (125 MHz, $CDCl_3$) δ 172.86, 172.71, 172.66, 172.58, 137.5, 136.7, 136.5, 136.1, 129.4, 129.2, 128.0, 127.3, 58.6, 57.6, 53.09, 52.96, 52.93, 52.92, 49.0, 48.4, 46.7, 45.6, 42.4, 38.93, 38.87, 38.1, 35.6, 35.5, 21.01, 20.97.

FTIR (ATR) 2953, 1905, 1730, 1516, 1434, 1350, 1252, 1197, 1164, 1095, 1067, 1027, 1005, 936, 865, 844, 812, 759, 720, 699, 674 cm^{-1}.

HRMS m/z (DART) calcd for $C_{17}H_{22}BrO_4^+$ (M+H)$^+$, 369.0696; found, 369.0698.

Dimethyl 4-(bromomethyl)-3-(*m*-tolyl)-1,1-cyclopentanedicarboxylate (**5b**)

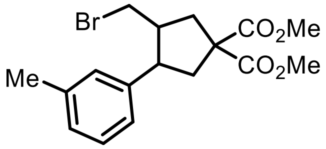

33 %, 12.0 mg, 74 : 26 d.r. (Colorless oil)

TLC (SiO_2) R_f = 0.40 (*n*-hexane : EtOAc = 4 : 1)

^{1}H NMR (500 MHz, $CDCl_3$) δ 7.23-7.19 (m, 1H), 7.06-6.99 (m, 3H), 3.79-3.77 (m, 6 H), 3.47-3.40 (m, 1H), 3.22 (dd, J = 10.3, 7.5 Hz, 0.74H), 2.95-2.85 (m, 1H), 2.81-2.71 (m, 2H), 2.63-2.59 (m, 0.22H), 2.45-2.18 (m, 6H).

^{13}C {^{1}H} NMR (125 MHz, $CDCl_3$) δ 172.85, 172.75, 172.65, 172.59, 140.5, 139.1, 138.4, 138.1, 128.9, 128.6, 128.4, 128.2, 127.8, 127.6, 125.1, 124.5, 58.5, 57.6, 52.9 (4C), 49.4, 48.4, 47.1, 45.5, 42.4, 38.91, 38.85, 37.8, 35.6, 35.5, 21.44, 21.36.

FTIR (ATR) 2953, 2924, 2853, 1733, 1607, 1589, 1491, 1435, 1259, 1200, 1163, 1096, 937, 866, 786, 704 cm^{-1}.

HRMS m/z (DART) calcd for $C_{17}H_{22}BrO_4^+$ (M+H)$^+$, 369.0696; found, 369.0698.

Dimethyl 4-(bromomethyl)-3-(*o*-tolyl)-1,1-cyclopentanedicarboxylate (**5c**)

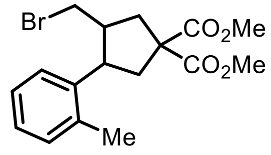

40 %, 14.7 mg, 62 : 38 d.r. (Colorless oil)

TLC (SiO_2) R_f = 0.40 (*n*-hexane : EtOAc = 4 : 1)

^{1}H NMR (500 MHz, $CDCl_3$) δ 7.24-7.11 (m, 4H), 3.80-3.76 (m, 6H), 3.56-3.53 (m, 0.60H), 3.45-3.42 (m, 0.34H), 3.25-3.21 (m, 0.63H), 2.91-2.58 (m, 5H), 2.37-2.32 (m, 3.60H), 2.24-2.14 (m, 0.61H).

^{13}C {^{1}H} NMR (125 MHz, $CDCl_3$) δ 172.8, 172.7, 172.6, 172.5, 138.8, 136.8, 136.7, 136.5, 130.7 (2C), 130.5, 126.8, 126.6, 126.5, 126.0, 125.4, 58.2, 53.0, 52.9, 47.4, 44.5, 43.6, 42.6, 42.4, 39.1, 38.8, 37.6, 36.4, 35.5, 19.9, 19.8.

FTIR (ATR) 3021, 2953, 2924, 2850, 1918, 1730, 1604, 1492, 1458, 1434, 1380, 1257, 1198, 1170, 1122, 1095, 1048, 1031, 1004, 938, 865, 946, 820, 903, 758, 727, 705, 676 cm^{-1}.

HRMS m/z (DART) calcd for $C_{17}H_{22}BrO_4^+$ $(M+H)^+$, 369.0696; found, 369.0705.

Dimethyl 4-(bromomethyl)-3-(4-(*tert*-butyl)phenyl)-1,1-cyclopentanedicarboxylate (**5d**)

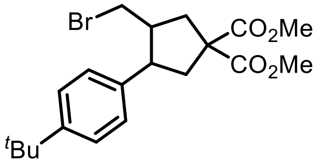

46 %, 18.9 mg, 56 : 44 d.r. (Colorless oil)

TLC (SiO_2): R_f = 0.40 (*n*-hexane : EtOAc = 4 : 1)

^{1}H NMR (500 MHz, $CDCl_3$) δ 7.34-7.32 (m, 2H), 7.16 (d, *J* = 8.0 Hz, 1.1H), 7.11 (d, *J* = 8.6 Hz, 0.9H), 3.79-3.73 (m, 6H), 3.46-3.41 (m, 1H), 3.22 (dd, *J* =10.5, 7.5 Hz, 0.41H), 2.97-2.68 (m, 4H), 2.61 (dd, *J* = 13.8, 10.9 Hz, 0.51H), 2.41-2.36 (m, 1.45H), 2.24 (dd, *J* = 13.8, 10.9 Hz, 0.43H), 1.30 (s, 9H).

^{13}C {^{1}H} NMR (125 MHz, $CDCl_3$) δ 172.9, 172.72, 172.67, 172.6, 149.9, 149.7, 137.4, 136.1, 127.8, 127.1, 125.6, 125.4, 58.6, 57.6, 53.0, 52.9, 52.8, 48.9, 48.4, 46.7, 45.6, 42.3, 39.0, 38.8, 38.0, 35.7, 35.6, 34.4, 31.3. * *Carbon signal of tBu was overlapped with each diastereomers.*

FTIR (ATR) 2955, 2905, 2869, 1910, 1731, 1511, 1458, 1434, 1394, 1363, 1254, 1199, 1165, 1096, 1067, 1023, 937, 866, 832, 802, 761, 745, 703, 680 cm^{-1}.

HRMS m/z (DART) calcd for $C_{20}H_{28}BrO_4^+$ $(M+H)^+$, 411.1165; found, 411.1168.

Dimethyl 4-(bromomethyl)-3-(4-methoxyphenyl)-1,1-cyclopentanedicarboxylate (**5e**)

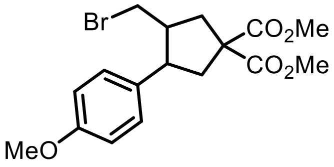

Quant, 38.5 mg, 63 : 37 d.r. (Colorless oil)

TLC (SiO_2) R_f = 0.20 (*n*-hexane : EtOAc = 4 : 1)

^{1}H NMR (500 MHz, $CDCl_3$) *δ* 7.17-7.10 (m, 2H), 6.87-6.84 (m, 2H), 3.83-3.75 (m, 9H), 3.45-3.40 (m,1H), 3.21 (dd, *J* = 10.3, 7.5 Hz, 0.36H), 2.94-2.66 (m, 4H), 2.57 (dd, *J* = 13.8, 10.3 Hz, 0.59H), 2.38-2.33 (m, 1.32H), 2.23 (dd, *J* = 13.8, 10.9 Hz, 0.37H).

^{13}C {^{1}H} NMR (125 MHz, $CDCl_3$) *δ* 172.9, 172.70, 172.65, 172.59, 158.6, 158.4, 132.5, 131.2, 129.1, 128.4, 114.1, 113.9, 58.6, 57.5, 55.2, 53.00, 52.97, 52.93, 52.91, 48.6, 48.5, 46.3, 45.7, 42.4, 38.8, 38.2, 35.6, 35.4.

FTIR (ATR) 3000, 2954, 2927, 2840, 1731, 1612, 1583, 1515, 1435, 1351, 1249, 1199, 1180, 1167, 1096, 166, 1035, 938, 866, 832, 809, 757 cm^{-1}.

HRMS m/z (DART) calcd for $C_{17}H_{22}BrO_5^+$ $(M+H)^+$, 385.0645; found, 385.0652.

Dimethyl 4-(bromomethyl)-3-(4-fluorophenyl)-1,1-cyclopentanedicarboxylate (**5f**)

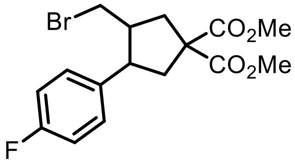

52 %, 19.5 mg, 58 : 42 d.r. (Colorless oil)

TLC (SiO_2) R_f = 0.40 (*n*-hexane : EtOAc = 4 : 1)

^{1}H NMR (500 MHz, $CDCl_3$) *δ* 7.22-7.15 (m, 2H), 7.03-6.97 (m, 2H), 3.81-3.73 (m, 6H), 3.47 (ddd, *J* = 10.3, 8.0, 7.5 Hz, 0.58H), 3.40 (dd, *J* = 10.3, 3.4 Hz, 0.42H), 3.20 (dd, *J* = 10.3, 6.9 Hz, 0.42H), 2.96-2.868 (m, 4H), 2.57 (dd, *J* = 13.8, 10.3 Hz, 0.60H), 2.38-2.23 (m, 1.83H).

^{13}C {^{1}H} NMR (125 MHz, $CDCl_3$) *δ* 172.8, 172.6, 172.50, 172.47, 161.8 (d, J_{C-F} = 245.6 Hz), 161.7 (d, J_{C-F} = 245.6HZ), 136.3, 135.0, 129.6 (d, J_{C-F} = 7.7 Hz), 128.9 (d, J_{C-F} = 6.7 Hz), 115.6 (d, J_{C-F} = 22.2 Hz), 115.4 (d, J_{C-F} = 22.2 Hz), 58.5, 57.5, 53.1, 53.0, 52.9, 52.8, 48.52, 48.46, 46.3, 45.6, 42.3, 38.8, 38.2, 35.2, 34.8. * One carbon signal was overlapped.

^{19}F NMR (475 MHz, $CDCl_3$): *δ* -115.7.

FTIR (ATR): 2955, 2851, 1731, 1606, 1512, 1435, 1261, 1226, 1200, 1162, 1094, 1067, 1027, 1016, 939, 866, 837, 817, 762 cm^{-1}.

HRMS m/z (DART): calcd for $C_{16}H_{19}BrFO_4^+$ $(M+H)^+$, 373.0445; found, 373.0445.

Dimethyl 4-(bromomethyl)-3-(4-chlorophenyl)-1,1-cyclopentanedicarboxylate (**5g**)

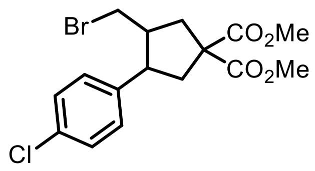

29 %, 11.3 mg, 72 : 28 d.r. (Colorless oil)

TLC (SiO_2) R_f = 0.40 (*n*-hexane : EtOAc = 4 : 1)

^{1}H NMR (500 MHz, $CDCl_3$) δ 7.30-7.28 (m, 2H), 7.19-7.13 (m, 2H), 3.82-3.73 (m, 6H), 3.46 (ddd, *J* = 10.3, 8.0, 7.5 Hz, 0.72H), 3.40 (dd, *J* = 10.3, 2.9 Hz, 0.26H), 3.20 (dd, *J* = 10.3, 6.9 Hz, 0.27H), 2.96-2.53 (m, 5H), 2.38-2.33 (m, 1.32H), 2.26 (dd, *J* = 14.3, 10.9 Hz, 0.28H).

^{13}C {^{1}H} NMR (125 MHz, $CDCl_3$) δ 172.8, 172.6, 172.5, 172.4, 139.2, 137.9, 132.8, 132.7, 129.5, 128.92, 128.86, 128.7, 58.6, 57.6, 53.08, 53.05, 53.02, 52.97, 48.6, 48.4, 46.4, 45.6, 42.2, 38.8, 38.1, 35.1, 34.7. * One carbon signal was overlapped.

FTIR (ATR) 2954, 2850, 1731, 1597, 1494, 1435, 1414, 1347, 1266, 1200, 1168, 1093, 1066, 1027, 1014, 937, 866, 830, 801, 762, 719 cm^{-1}.

HRMS m/z (DART) calcd for $C_{16}H_{19}BrClO_4^+$ $(M+H)^+$, 389.0150; found, 389.0158.

Dimethyl 4-(bromomethyl)-3-(4-bromophenyl)-1,1-cyclopentanedicarboxylate (**5h**)

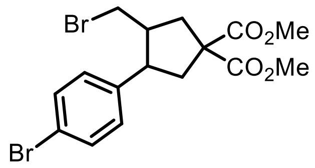

43 %, 18.6 mg, 56 : 44 d.r. (Colorless oil)

TLC (SiO_2) R_f = 0.40 (*n*-hexane : EtOAc = 4 : 1)

^{1}H NMR (500 MHz, $CDCl_3$) δ 7.45-7.44 (m, 2H), 7.14-7.07 (m, 2H), 3.81-3.73 (m, 6H), 3.43 (ddd, J = 10.3, 8.0, 7.5 Hz, 0.56H), 3.38 (dd, J = 10.3, 2.9 Hz, 0.42H), 3.20 (dd, *J* = 10.3, 6.9 Hz, 0.44H), 2.96-2.67 (m, 4H), 2.55 (dd, *J* = 13.8, 10.3 Hz, 0.63H), 2.38-2.33 (m, 1.41H), 2.25 (dd, *J* = 10.3, 3.4 Hz, 0.47H).

^{13}C {^{1}H} NMR (125 MHz, $CDCl_3$) δ 172.7, 172.5, 172.43, 172.39, 139.7, 138.4, 131.9, 131.6, 129.9, 129.2, 120.81, 120.76, 58.5, 57.6, 53.1, 53.04, 53.00, 52.96, 48.7, 48.3, 46.5, 45.5, 42.1, 38.8, 38.0, 35.1, 34.6. * One carbon signal was overlapped.

FTIR (ATR) 2952, 2924, 2849, 1905, 1730, 1591, 1490, 1434, 1410, 1347, 1259, 1200, 1166, 1096, 1073, 1027, 1010, 936, 865, 825, 759, 717, 676 cm^{-1}.

HRMS m/z (DART) calcd for $C_{16}H_{19}Br_2O_4^+$ $(M+H)^+$, 432.9645; found, 432.9661.

Dimethyl-3-([1,1'-biphenyl]-4-yl)-4-(bromomethyl)-1,1-cyclopentanedicarboxylate (**5i**)

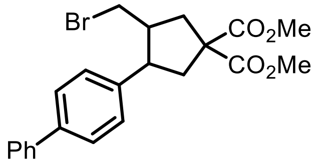

55 %, 23.5 mg, 54 : 46 d.r. (Colorless oil)

TLC (SiO_2) R_f = 0.20 (*n*-hexane : EtOAc = 4 : 1)

^{1}H NMR (500 MHz, $CDCl_3$) *δ* 7.59-7.54 (m, 4H), 7.45-7.42 (m, 2H), 7.36--7.27 (m, 3H), 3.83-3.74 (m, 6H), 3.53 (ddd, J = 10.3, 8.0, 7.5 Hz, 0.58H), 3.46 (dd, *J* = 10.3, 7.5 Hz, 0.42H), 3.26 (dd, *J* = 10.3, 6.9 Hz, 0.44H), 3.02-2.96 (m, 1.58H), 2.86-2.65 (m, 3.10H), 2.49-2.39 (m, 1.34H), 2.28 (dd, *J* = 13.8, 10.3 Hz, 0.44H).

^{13}C {^{1}H} NMR (125 MHz, $CDCl_3$) *δ* 172.8, 172.7, 172.61, 172.56, 140.7, 140.6, 140.0, 139.8, 139.7, 138.4, 128.8, 128.6, 127.9, 127.5, 127.3, 127.25, 127.19, 127.0, 126.9, 58.6, 57.6, 53.05, 53.02, 52.98, 52.95, 49.0, 48.4, 46.8, 45.7, 42.3, 38.94, 38.88, 38.1, 35.5, 35.3. * One carbon signal was overlapped.

FTIR (ATR) 3029, 2953, 2925, 2852, 1732, 1600, 1522, 1488, 1435, 1263, 1200, 1167, 1097, 1008, 937, 839, 766, 736, 699 cm^{-1}.

HRMS m/z (DART) calcd for $C_{22}H_{24}BrO_4^+$ $(M+H)^+$, 431.0852; found, 431.0874.

Dimethyl-4-(bromomethyl)-3-(naphthalen-2-yl)-1,1-cyclopentanedicarboxylate (**5j**)

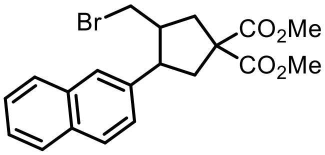

38 %, 15.4 mg, 57 : 43 d.r. (Colorless oil)

TLC (SiO_2) R_f = 0.50 (*n*-hexane : EtOAc = 4 : 1)

^{1}H NMR (500 MHz, $CDCl_3$) *δ* 7.83-7.79 (m, 3H), 7.68-7.63 (m, 1H), 7.49-7.45 (m, 2H), 7.39-7.31 (m, 1H), 3.84-3.74 (m, 6H), 3.68-3.63 (m, 0.58H), 3.44 (dd, J = 10.3, 3.4 Hz, 0.41H), 3.25 (dd, *J* = 10.3, 6.9 Hz, 0.42H), 3.14-3.08 (m, 0.47H), 2.96-2.44 (m, 5.39H), 2.31 (dd, *J* = 13.8, 10.9 Hz, 0.43H).

^{13}C {^{1}H} NMR (125 MHz, $CDCl_3$) *δ* 172.9, 172.7, 172.62, 172.60, 138.0, 136.7, 133.5, 133.3, 132.6, 132.3, 128.6, 128.2, 127.7, 127.62, 127.58, 126.6, 126.5, 126.4, 126.30, 126.28, 125.8, 125.7, 125.2, 58.6, 57.7, 53.07, 53.05, 53.01, 52.97, 49.5, 48.3, 47.2, 45.5, 42.3, 39.0, 38.9, 37.8, 35.5, 35.4. * One carbon signal was overlapped.

FTIR (ATR) 3054, 2953, 4731, 1633, 1601, 1508, 1435, 1377, 1260, 1227, 1200, 1166, 1127, 1097, 1069, 1027, 959, 892, 858, 820, 751, 703 cm^{-1}.

HRMS m/z (DART) calcd for $C_{20}H_{22}BrO_4^+$ $(M+H)^+$, 405.0696; found, 405.0704.

Dimethyl-4-(bromomethyl)-3-(pyridine-2-yl)-1,1-cyclopentanedicarboxylate (**5k**)

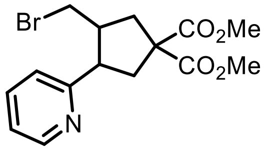

67 %, 24.0 mg, 70 : 30 d.r. (Colorless oil)

TLC (SiO_2) R_f = 0.20 (*n*-hexane : EtOAc = 4 : 1)

^{1}H NMR (500 MHz, $CDCl_3$) δ 8.56-8.55 (m, 1H), 7.64-7.60 (m, 1H), 7.21-7.14 (m, 2H), 3.82-3.76 (m, 6H), 3.48 (dd, *J* = 10.3, 3.4 Hz, 0.72H), 3.30 (dd, J = 10.3, 6.3 Hz, 0.71H), 3.18-3.12 (m, 0.73H), 3.04-2.53 (m, 4.23H), 2.25-2.05 (m, 1.59H).

^{13}C {^{1}H} NMR (125 MHz, $CDCl_3$) δ major diastereomer 172.8, 172.4, 160.1, 149.7, 136.5, 123.2, 122.0, 58.0, 53.85, 52.89, 50.9, 46.9, 41.1, 38.6, 36.0.

FTIR (ATR) 3458, 3004, 2955, 2894, 2844, 1730, 1591, 1571, 1435, 1254, 1202, 1166, 964, 864, 753, 729 cm^{-1}.

HRMS m/z (DART) calcd for $C_{15}H_{19}BrNO_4^+$ $(M+H)^+$, 356.0492; found, 356.0494.

Dimethyl 4-(bromomethyl)-2-methyl-3-phenyl-1,1-cyclopentanedicarboxylate (**5l**)

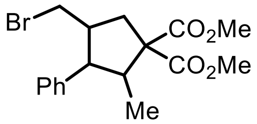

26 %, 9.6 mg, 59 : 41 d.r. (Colorless oil)

TLC (SiO_2) R_f = 0.60 (*n*-hexane : EtOAc = 4 : 1)

^{1}H NMR (500 MHz, $CDCl_3$)

Major δ 7.34-7.31 (m, 2H), 7.26-7.23 (m, 1H), 7.16 (d, *J* = 7.5 Hz, 2H), 3.80 (s, 3H), 3.77 (s, 3H), 3.18 (dd, *J* = 12.6, 7.5 Hz, 1H), 3.11 (dd, *J* = 12.6, 6.9 Hz, 1H), 2.96-2.85 (m, 2H), 2.58-2.49 (m, 2H), 2.09-2.04 (m, 1H), 0.98 (d, *J* = 6.3 Hz, 3H).

Minor δ 7.34-7.31 (m, 2H), 7.26-7.23 (m, 1H), 7.20 (d, *J* = 7.5 Hz, 2H), 3.79 (s, 3H), 3.76 (s, 3H), 3.36 (dd, *J* = 10.3, 3.4 Hz, 1H), 3.30 (dd, *J* = 10.3, 8.6 Hz, 1H), 2.96-2.85 (m, 4H), 2.43-2.37 (m, 1H), 0.86 (d, *J* = 6.9 Hz, 3H).

^{13}C {^{1}H} NMR (125 MHz, $CDCl_3$)

Major δ 172.5, 171.9, 138.0, 128.7, 128.6, 127.0, 62.0, 54.0, 52.7, 52.3, 47.6, 43.5, 40.0, 37.7, 30.9.

Minor δ 172.8, 172.1, 140.1, 128.8, 127.9, 127.2, 61.6, 56.6, 52.7, 52.3, 47.9, 43.0, 38.7, 35.9, 29.7.

FTIR (ATR) 2954, 1729, 1602, 1497, 1455, 1435, 1380, 1261, 1206, 1157, 1157, 1071, 1013, 803, 778, 749, 702 cm^{-1}.

HRMS m/z (DART) calcd for $C_{17}H_{22}BrO_4^+$ $(M+H)^+$, 369.0696; found, 369.0709.

6. Synthesis of cyclic compounds 6

Diethyl 4-(bromomethyl)-3-phenylcyclopent-2-ene-1,1-dicarboxylate (**6b**)[1]

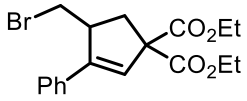

51 %, 19.4 mg (Colorless oil)

TLC (SiO$_2$) R$_f$ = 0.40 (*n*-hexane : EtOAc = 4 : 1)

^{1}H NMR (500 MHz, CDCl$_3$) *δ* 7.42-7.30 (m, 5H), 6.20 (d, *J* = 1.2 Hz, 1H), 4.27-4.19 (m ,4H), 3.80-3.72 (m, 1H), 3.58 (d, *J* = 10.3 Hz, 1H), 3.31 (dd, *J* = 10.3, 9.7 Hz, 1H), 2.89 (dd, *J* = 13.8, 8.0 Hz, 1H), 2.63 (dd, *J* = 13.8, 4.0 Hz, 1H), 1.32-1.25 (m, 6H).

^{13}C {^{1}H} NMR (125 MHz, CDCl$_3$) *δ* 170.84, 170.77, 147.6, 133.8, 128.7, 128.5, 126.6, 126.0, 65.3, 61.8, 47.5, 36.5, 14.1, 14.0.

Di(iso-propyl)-4-(bromomethyl)-3-phenylcyclopent-2-ene-1,1-dicarboxylate (**6c**)

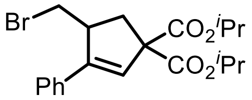

65 %, 26.6 mg (Colorless oil)

TLC (SiO$_2$) R$_f$ = 0.40 (*n*-hexane : EtOAc = 4 : 1)

^{1}H NMR (500 MHz, CDCl$_3$) δ 7.42-7.29 (m, 5H), 6.18 (d, *J* = 1.8 Hz, 1H), 5.11-5.02 (m ,2H), 3.77-3.75 (m, 1H), 3.57 (dd, *J* = 10.3, 3.4 Hz, 1H), 3.32 (dd, *J* = 10.3, 9.7 Hz, 1H), 2.85 (dd, *J* = 13.8, 8.6 Hz, 1H), 2.63 (dd, *J* = 13.8, 3.4 Hz, 1H), 1.29-1.21 (m, 12H).

^{13}C {^{1}H} NMR (125 MHz, CDCl$_3$) δ 170.4, 170.3, 147.5, 133.9, 128.7, 128.4, 126.6, 126.2, 69.2, 65.4, 47.6, 36.5, 36.3, 47.6, 36.5, 36.3, 21.6, 21.5.

FTIR (ATR) 3059, 2981, 2935, 1724, 1600, 1576, 1497, 1467, 1448, 1387, 1375, 1348, 1298, 1248, 1204, 1182, 1145, 1103, 1076, 1040, 1002, 962, 913, 855, 828, 765, 696, 673 cm^{-1}.

HRMS m/z (DART) calcd for $C_{20}H_{26}BrO_4^+$ $(M+H)^+$, 409.1009; found, 409.1007.

Ethyl 1-acetyl-4-(bromomethyl)-3-phenylcyclopent-2-enecarboxylate (**6d**)[1]

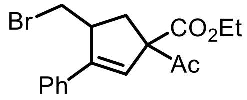

78 %, 27.3 mg, 57 : 43 d.r. (Colorless oil)

TLC (SiO$_2$) R$_f$ = 0.20 (*n*-hexane : EtOAc = 4 : 1)

^{1}H NMR (500 MHz, CDCl$_3$) δ 7.41-7.32 (m, 5H), 6.23-6.22 (m, 1H), 4.20-4.19 (m, 2H), 3.78-3.72 (m, 1H), 3.60-3.54 (m, 1H), 3.31-3.23 (m, 1H), 2.92 (dd, *J* = 14.3, 8.6 Hz, 0.44H), 2.80 (dd, *J* =

14.3, 8.6 Hz, 0.54H), 2.61 (dd, J = 14.3, 4.6 Hz, 0.53H), 2.53 (dd, J = 14.3, 4.6 Hz, 0.43H), 2.31 (s, 1.30H), 2.27 (s, 1.58H), 1.32 (t, J = 13.8 Hz, 1.71H), 1.26 (t, J = 13.8 Hz, 1.29H).

^{13}C {^{1}H} NMR (125 MHz, $CDCl_3$) δ 203.5, 202.8, 171.2, 171.1, 148.1, 148.0, 133.9, 133.8, 128.7, 128.5, 126.51, 126.48, 126.0, 125.8, 72.1 (2C), 61.9 (2C), 47.3, 47.1, 36.5 (2C), 35.1, 35.0, 27.0, 26.5, 14.1, 14.0. * Two carbon signals were overlapped.

Dimethyl 4-(iodomethyl)-3-phenylcyclopent-2-ene-1,1-dicarboxylate (**6e**)

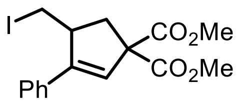

73 %, 29.1 mg. (Colorless oil)

TLC (SiO_2) R_f = 0.40 (*n*-hexane : EtOAc = 4 : 1)

^{1}H NMR (500 MHz, $CDCl_3$) δ 7.41-7.32 (m, 5H), 6.23 (d, J = 1.2 Hz, 1H), 3.80 (s ,3H), 3.74 (s ,3H), 3.70-3.65 (m, 1H), 3.42 (dd, J = 9.5, 2.9 Hz, 1H), 3.10 (t, J = 9.5 Hz, 1H), 2.94 (dd, J = 14.3, 8.0 Hz, 1H), 2.51 (dd, J = 14.3, 4.6 Hz, 1H).

^{13}C {^{1}H} NMR (125 MHz, $CDCl_3$) δ 171.13, 171.10, 148.8, 133.8, 128.7, 128.5, 126.6, 125.3, 64.8, 52.99, 52.97, 47.4, 38.5, 11.3.

FTIR (ATR) 3026, 2953, 2923, 2850, 1733, 1599, 1576, 1495, 1446, 1444, 1247, 1209, 1176, 1134, 1076, 1042, 1007, 966, 947, 922, 850, 765, 697 cm^{-1}.

HRMS m/z (DART) calcd for $C_{16}H_{18}IO_4^+$ $(M+H)^+$, 401.0244; found, 401.0236.

Dimethyl 4-(bromomethyl)-4-methyl-3-phenylcyclopent-2-ene-1,1-dicarboxylate (**6f**)

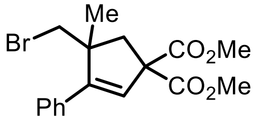

18 %, 6.2 mg. (Colorless oil)

TLC (SiO_2) R_f = 0.40 (*n*-hexane : EtOAc = 4 : 1)

^{1}H NMR (500 MHz, $CDCl_3$) δ 7.37-7.29 (m, 5H), 5.90 (s, 1H), 3.79 (s, 3H), 3.78 (s, 3H), 3.45 (s, 2H), 2.88 (d, J = 13.8 Hz, 1H), 2.53 (d, J = 13.8 Hz, 1H), 1.41 (s, 3H).

^{13}C {^{1}H} NMR (125 MHz, $CDCl_3$) δ 171.4, 171.3, 152.2, 135.0, 128.2, 128.0, 127.3, 63.6, 53.0, 52.9, 52.3, 44.0, 43.1, 25.6. * One carbon signal was overlapped.

FTIR (ATR) 2955, 2926, 2852, 1734, 1494, 1434, 1376, 1288, 1243, 1146, 1107, 1072, 1032, 960, 848, 766, 700 cm^{-1}.

HRMS m/z (DART) calcd for $C_{17}H_{20}BrO_4^+$ $(M+H)^+$, 367.0539; found, 367.0535.

Dimethyl 4-(bromomethyl)-3-phenylcyclohex-2-ene-1,1-dicarboxylate (**6g**)

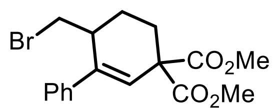

23 %, 8.4 mg. (Colorless oil)

TLC (SiO_2) R_f = 0.30 (*n*-hexane : EtOAc = 4 : 1)

^{1}H NMR (500 MHz, $CDCl_3$) δ 7.36-7.30 (m, 5H), 6.18 (d, *J* = 1.2 Hz, 1H), 3.80 (s, 3H), 3.72 (s, 3H), 3.34 (dd, *J* = 10.3, 2.9 Hz, 1H), 3.19 (dd, *J* = 10.3, 9.7 Hz, 1H), 3.17-3.10 (m, 1H), 2.32-2.27 (m, 1H), 2.16-2.10 (m, 2H), 2.06-2.00 (m, 1H).

^{13}C {^{1}H} NMR (125 MHz, $CDCl_3$) δ 171.1, 170.7, 142.1, 139.8, 128.6, 128.0, 126.6, 124.7, 55.5, 53.0, 52.9, 38.0, 35.5, 24.9, 23.2.

FTIR (ATR) 2954, 2926, 2853, 1734, 1600, 1576, 1493, 1446, 1434, 1362, 1264, 1236, 1218, 1172, 1146, 1079, 1062, 1015, 967, 917, 865, 762, 700, 676 cm-1.

HRMS m/z (DART) calcd for $C_{17}H_{20}BrO_4^+$ $(M+H)^+$, 367.0539; found, 367.0533.

(E)-Dimethyl 4-(bromomethylene)-3-phenylcyclopent-2-ene-1,1-dicarboxylate (**6h**)

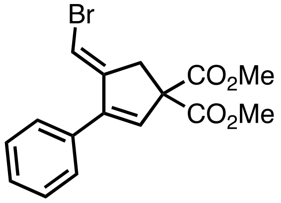

34 %, 11.9 mg, E / Z = 95 : 5 (Colorless oil)

TLC (SiO_2) R_f = 0.30 (*n*-hexane : EtOAc = 4 : 1)

^{1}H NMR (500 MHz, $CDCl_3$) δ 7.39-7.33 (m, 5H), 6.29 (s, 1H), 6.25 (t, *J* = 2.3 Hz, 1H), 3.79 (s ,6H), 3.39 (d, *J* = 2.3 Hz, 1H).

^{13}C {^{1}H} NMR (125 MHz, $CDCl_3$) δ 170.3, 147.2, 147.0, 133.2, 133.1, 128.63, 128.57, 128.1, 101.5, 63.1, 53.2, 39.4.

FTIR (ATR) 3084, 2954, 2847, 1736, 1672, 1622, 1494, 1435, 1259, 1198, 1170, 1119, 1064, 956, 845, 802, 759, 700 cm^{-1}.

HRMS m/z (DART) calcd for $C_{16}H_{16}BrO_4^+$ $(M+H)^+$, 351.0226; found, 351.0214.

Dimethyl 2-Allylidene Malonate (**7a**)[2]

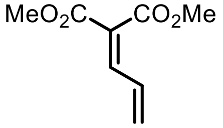

Trace (colorless oil)

TLC (SiO_2) R_f = 0.50 (*n*-hexane : EtOAc = 4 : 1)

^{1}H NMR (500 MHz, CDCl$_3$) δ 7.36 (d, J = 11.5 Hz, 1H), 6.80-6.73 (m, 1H), 5.80 (d, J = 17.2 Hz, 1H), 5.69 (d, J = 10.9 Hz, 1H), 3.86 (s, 3H), 3.81 (s, 3H).
^{13}C {^{1}H} NMR (125 MHz, CDCl$_3$) δ 165.4, 164.8, 145.2, 131.7, 130.0, 125.8, 52.5, 52.4.

Dimethyl 2-allyl-2-(3,5-di-*tert*-butyl-4-hydroxybenzyl)malonate (**7b**)

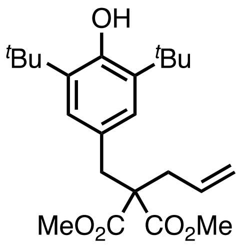

Trace (Colorless oil)
TLC (SiO$_2$) R_f = 0.50 (*n*-hexane : EtOAc = 4 : 1)
^{1}H NMR (500 MHz, CDCl$_3$) δ 6.87 (s, 2H), 5.79-5.70 (m, 1H), 5.17-5.13 (m, 2H), 5.10 (s, 1H), 3.73 (s, 6H), 3.16 (s, 2H), 2.54 (d, J = 7.5 Hz, 2H), 1.40 (s, 18H).
^{13}C {^{1}H} NMR (125 MHz, CDCl$_3$) δ 171.4, 152.7, 135.5, 132.9, 126.5, 126.2, 119.1, 59.1, 52.3, 38.0, 36.3, 34.2, 30.3, 29.7.
FTIR (ATR) 3645, 2956, 2925, 1737, 1437, 1365, 1210 cm-1.
HRMS m/z (DART) calcd for $C_{23}H_{35}O_5^+$ (M+H)$^+$, 391.2479; found, 391.2489.

7. Reference

[1] G. Yuzhen, Z. Pengbo, J. Zhe, T. Guo, Z. Yufen, *ACS Catal.* **2017**, *7*, 186–190.
[2] M. Sylla, D. Joseph, E. Chevallier, C. Camara, F. Dumas, *Synthesis* **2006**, *6*, 1045–1049.

8. ^{1}H NMR, ^{13}C NMR, ^{19}F and DEPT-135 NMR Spectra of 3

- ^{1}H and ^{13}C NMR Spectra of 3a

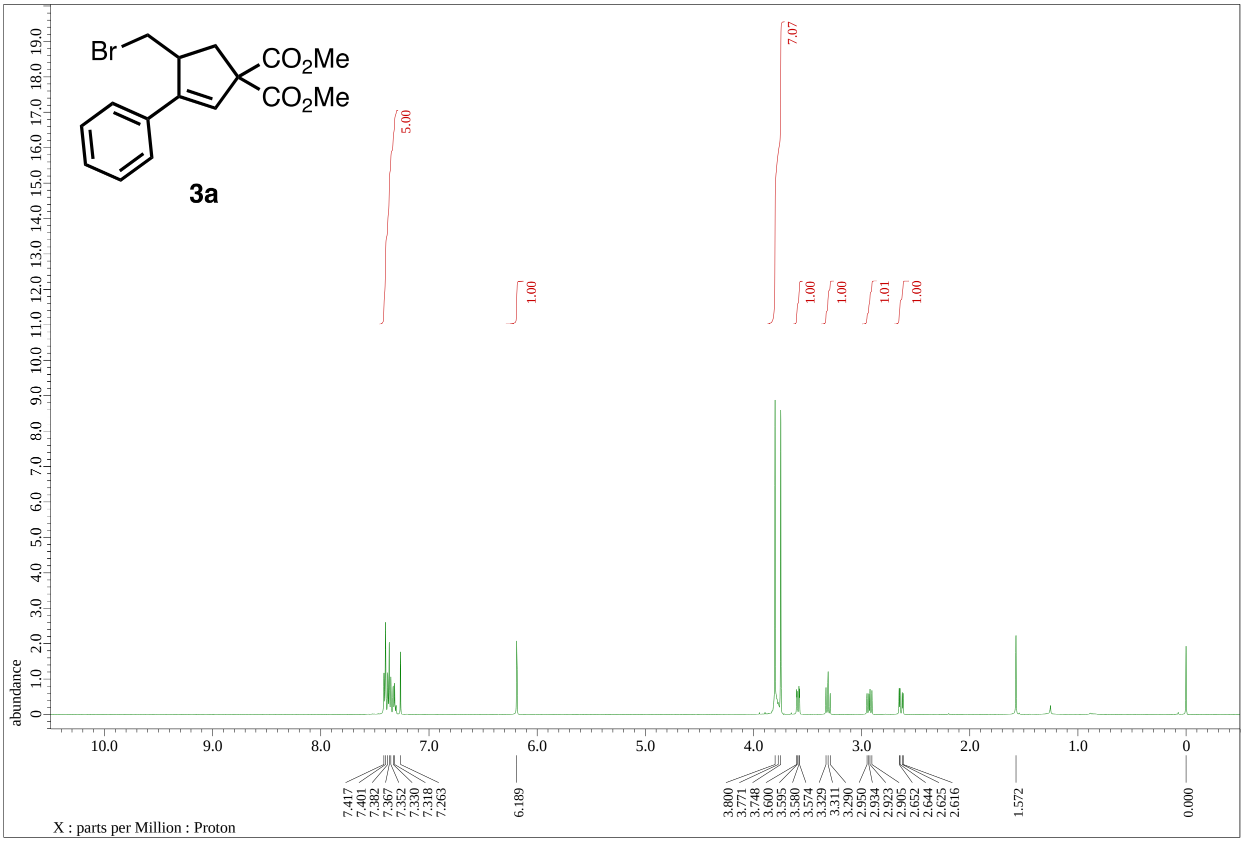

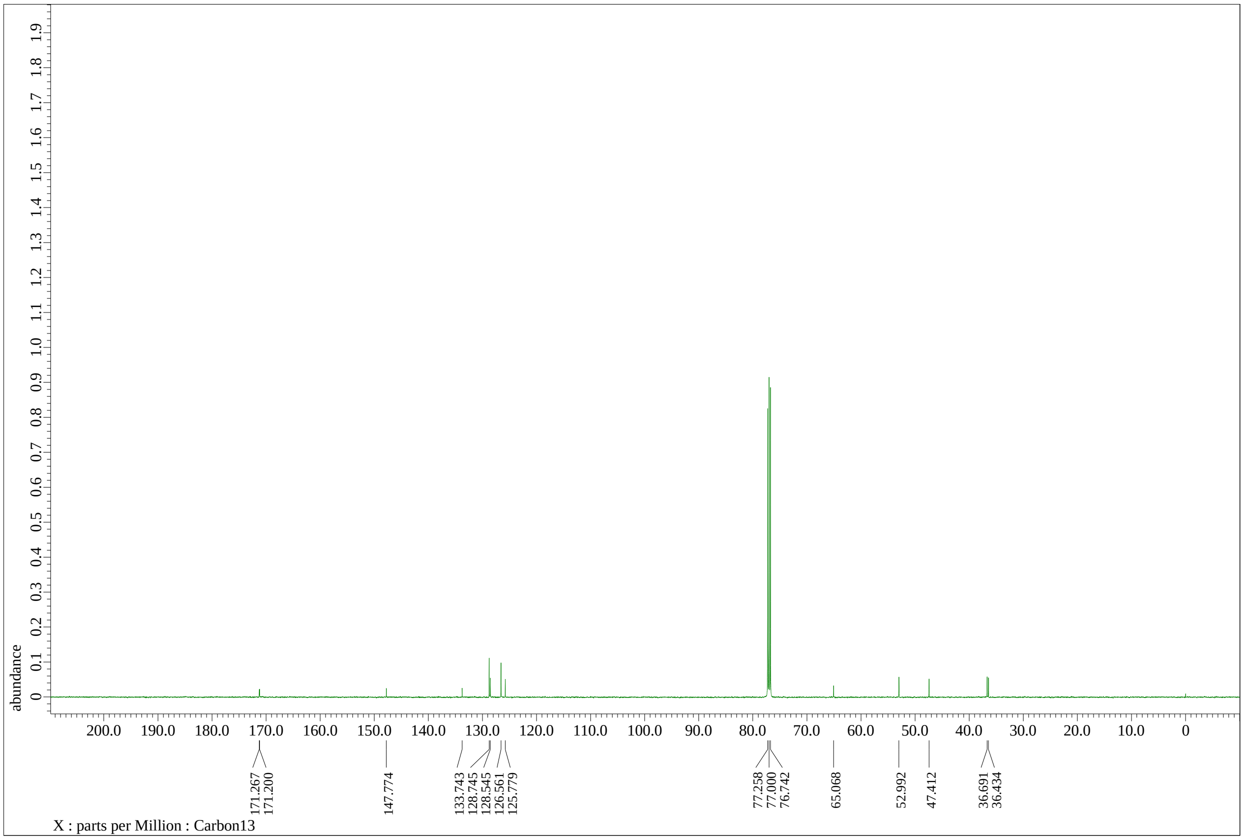

- ^{1}H and ^{13}C NMR Spectra of 3b

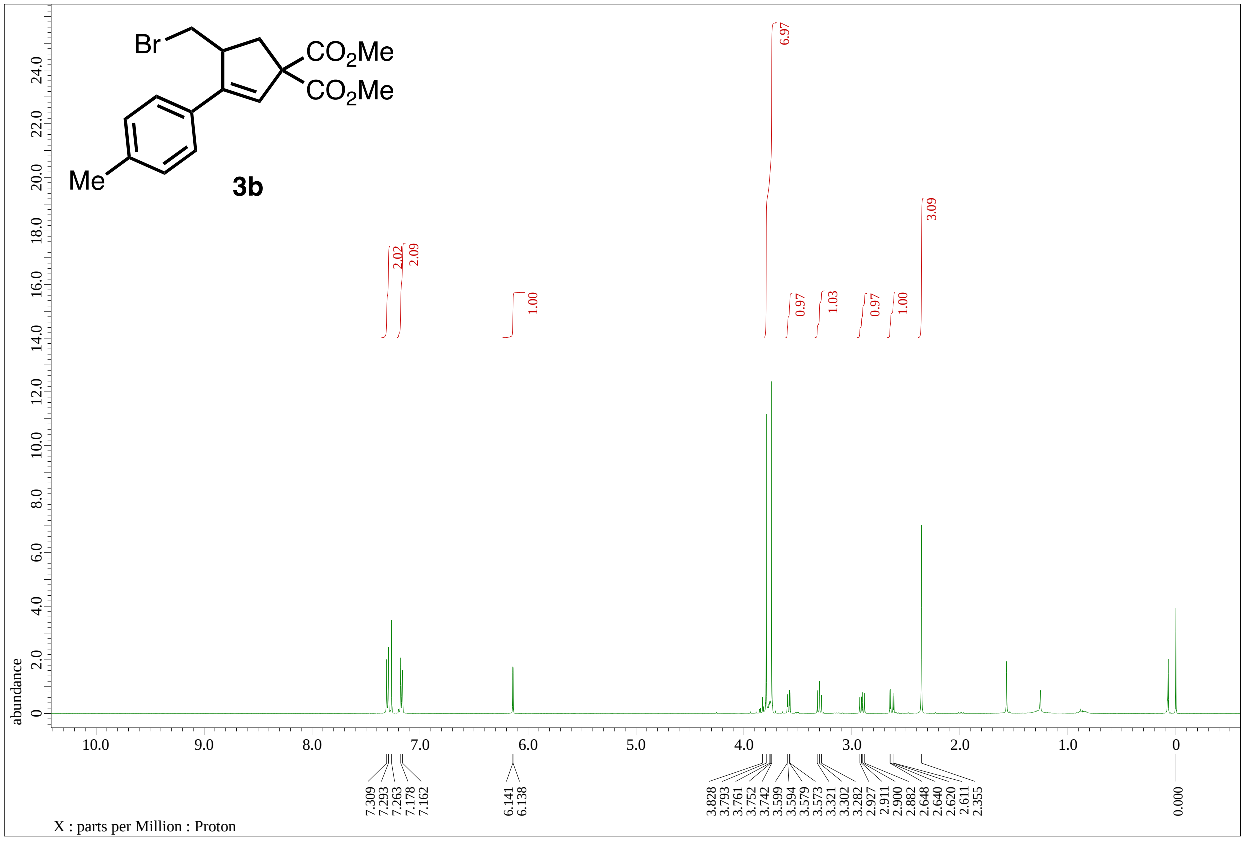

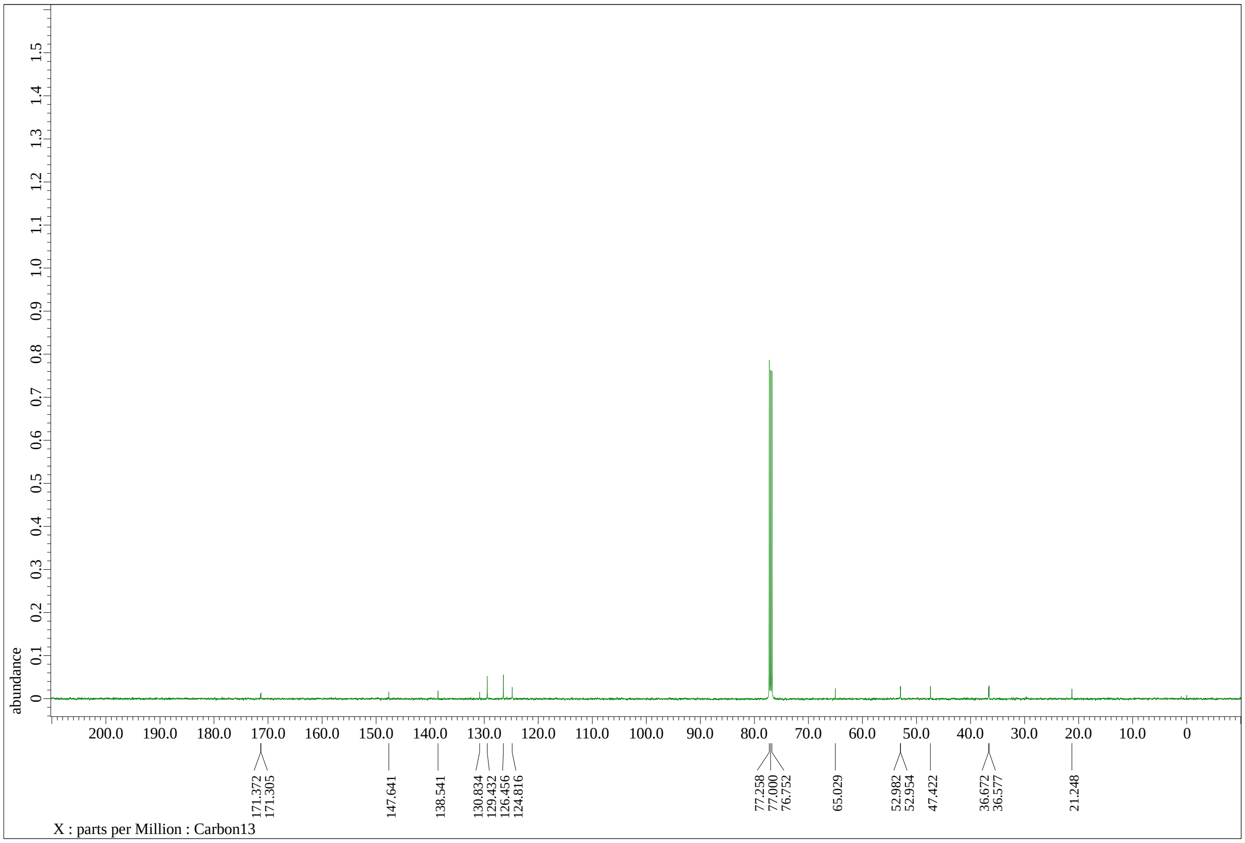

- ^{1}H and ^{13}C NMR Spectra of 3c

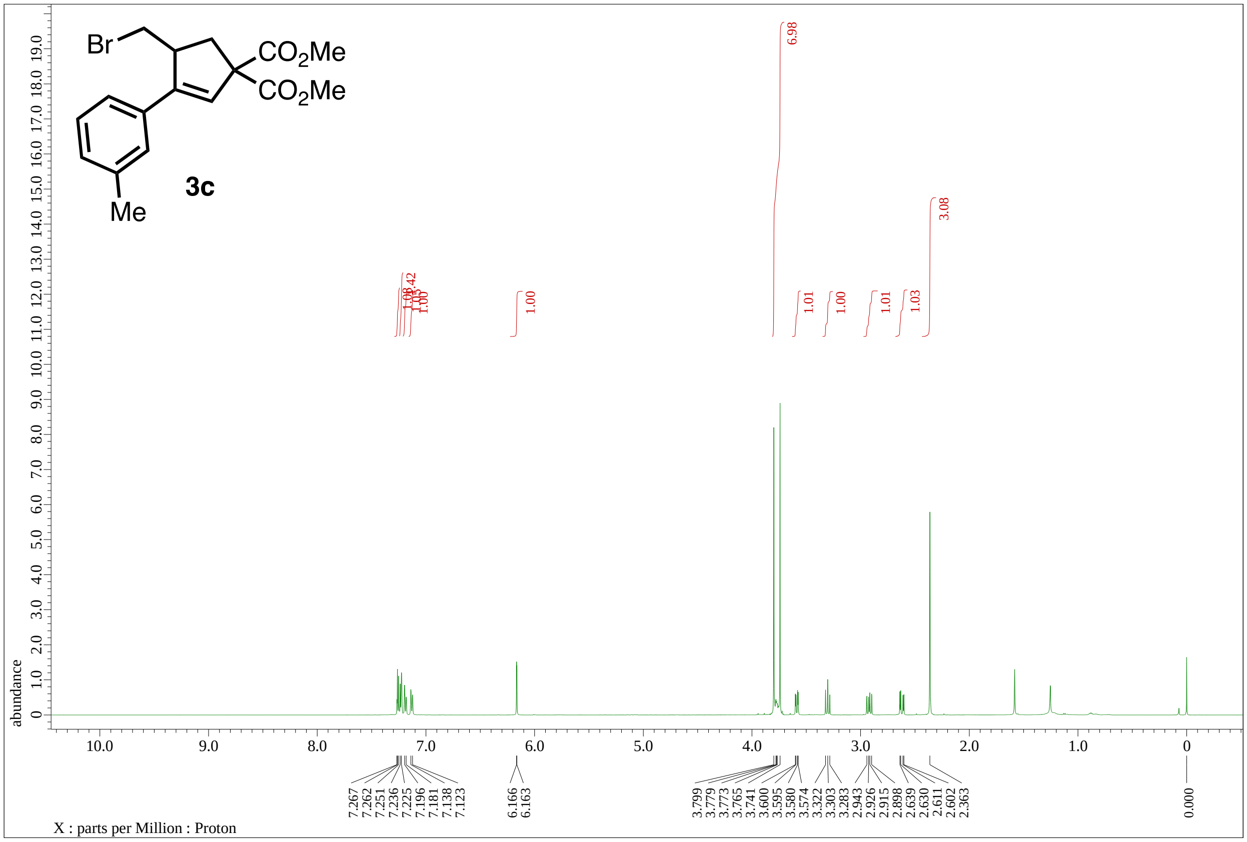

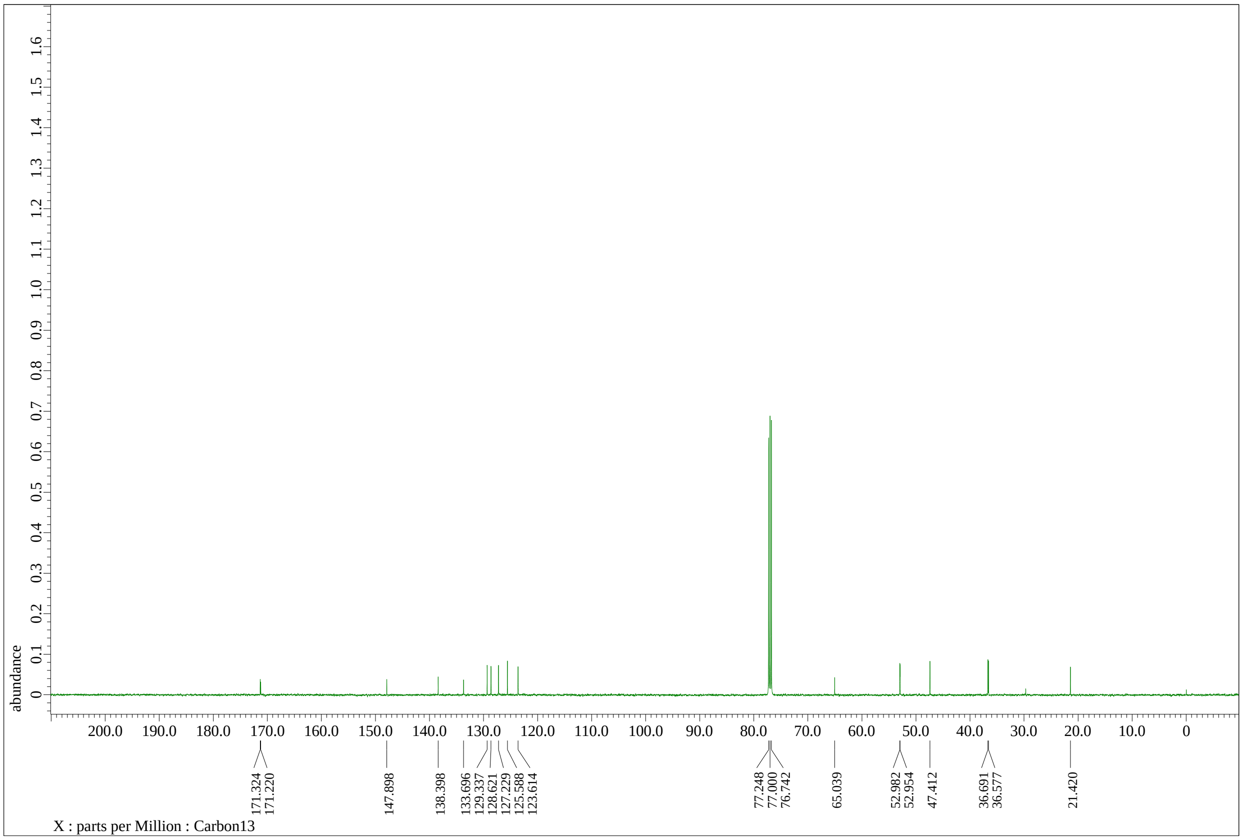

- ^{1}H and ^{13}C NMR Spectra of 3d

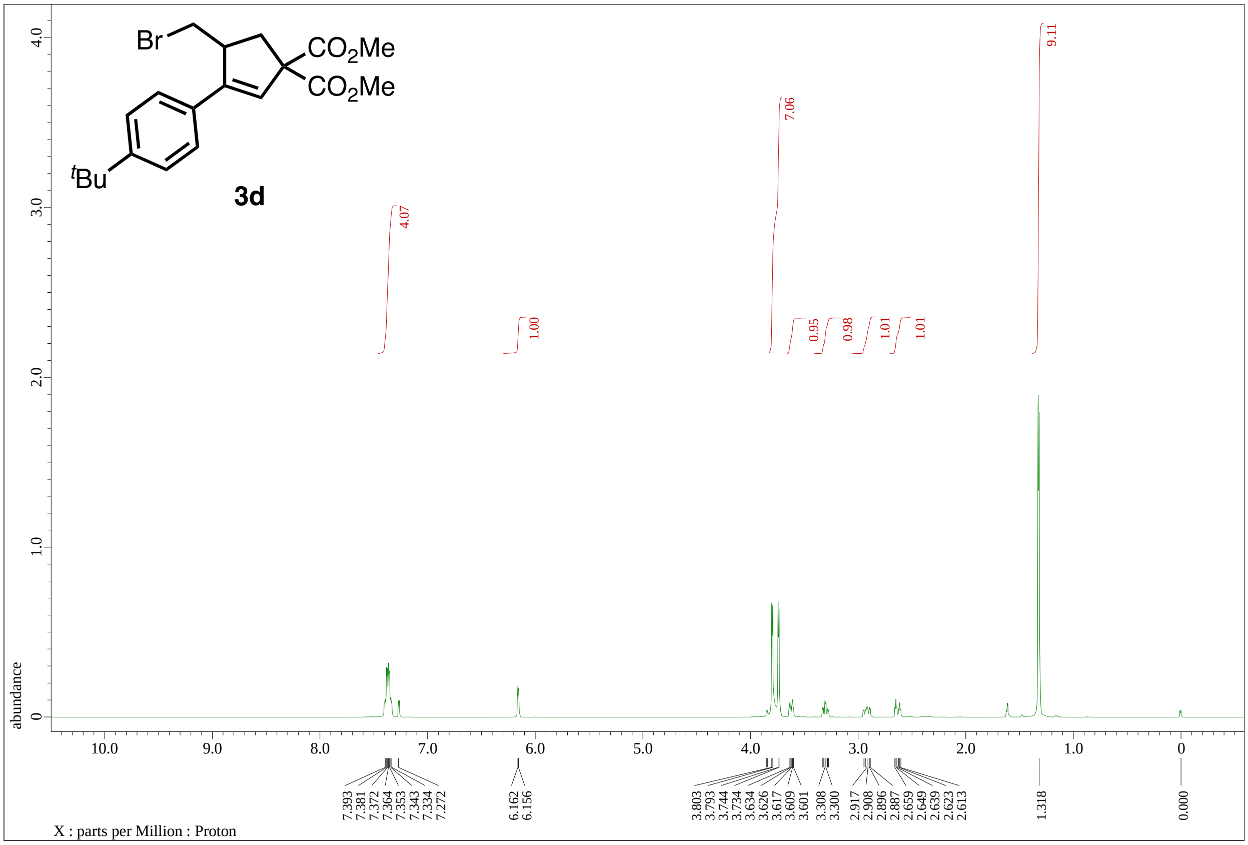

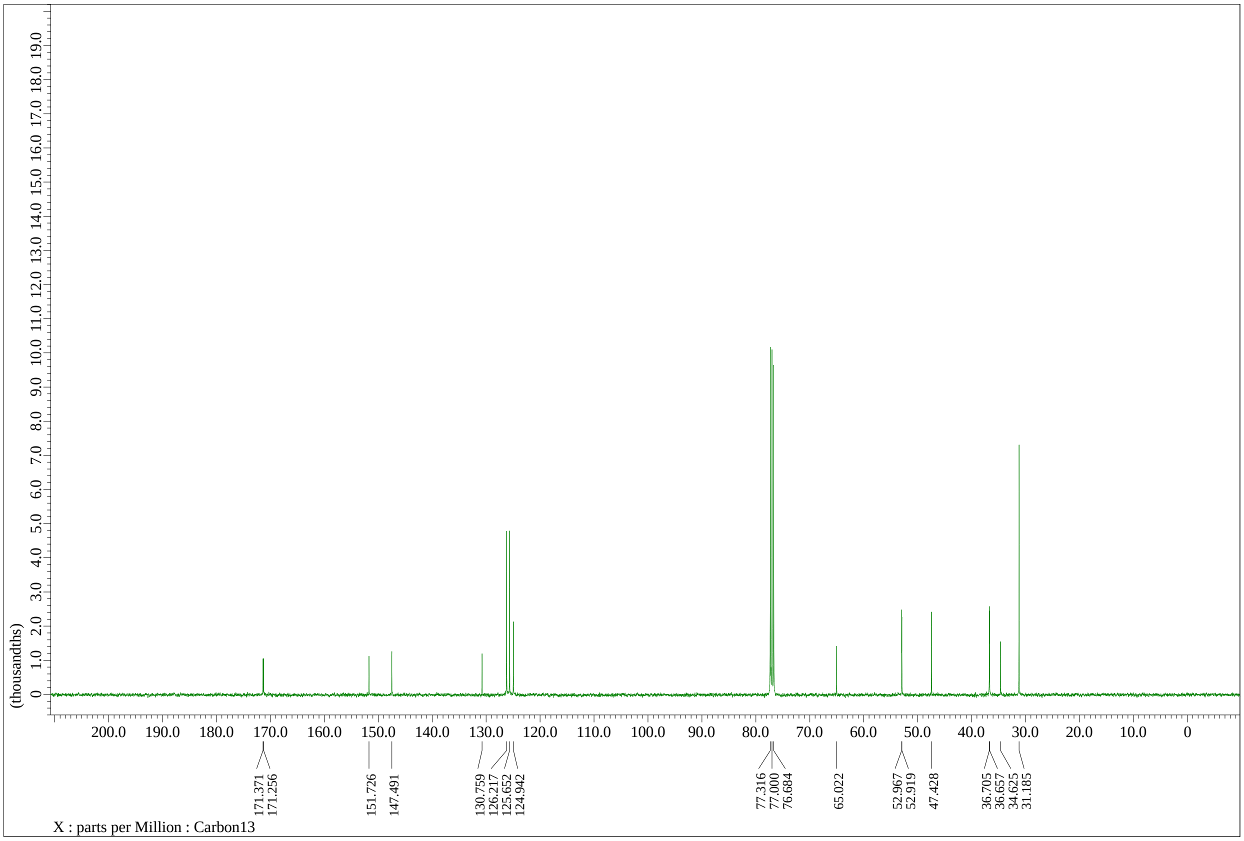

- ^{1}H, ^{13}C and ^{19}F NMR Spectra of 3e

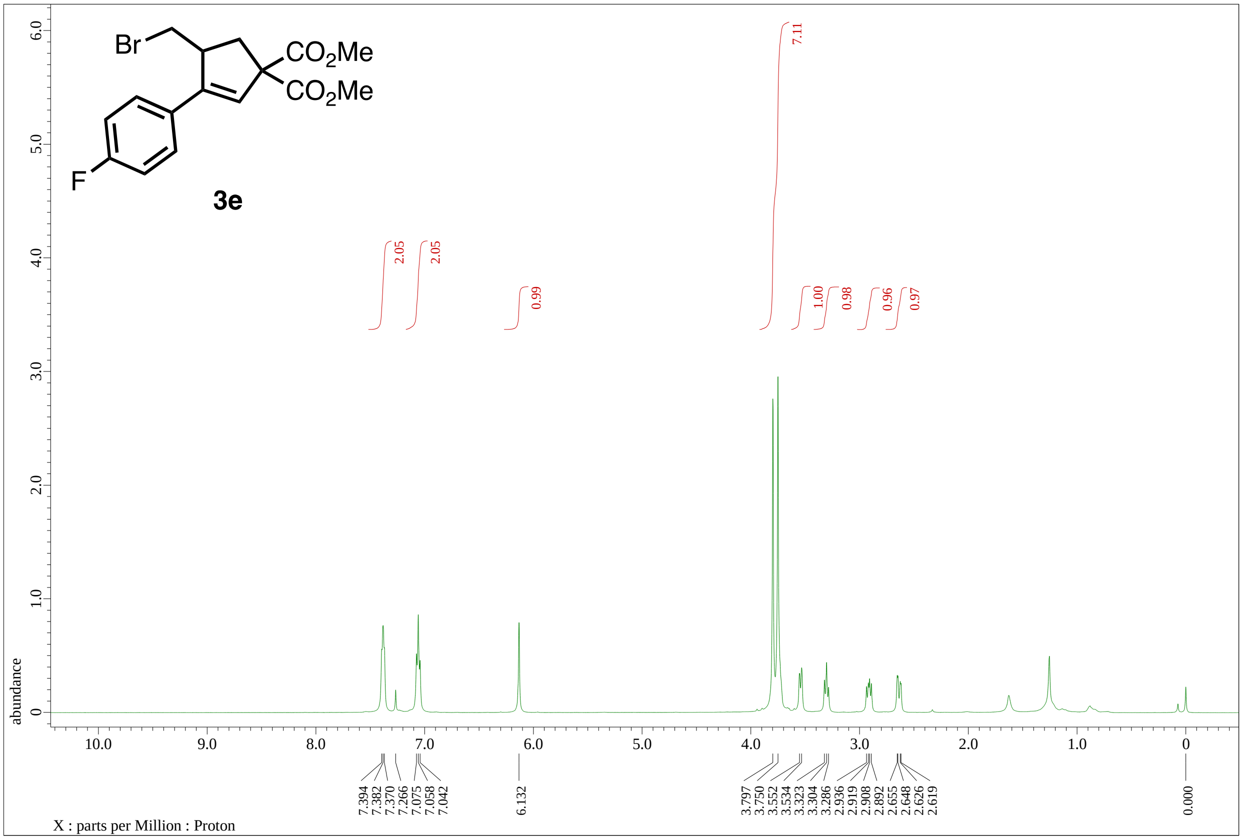

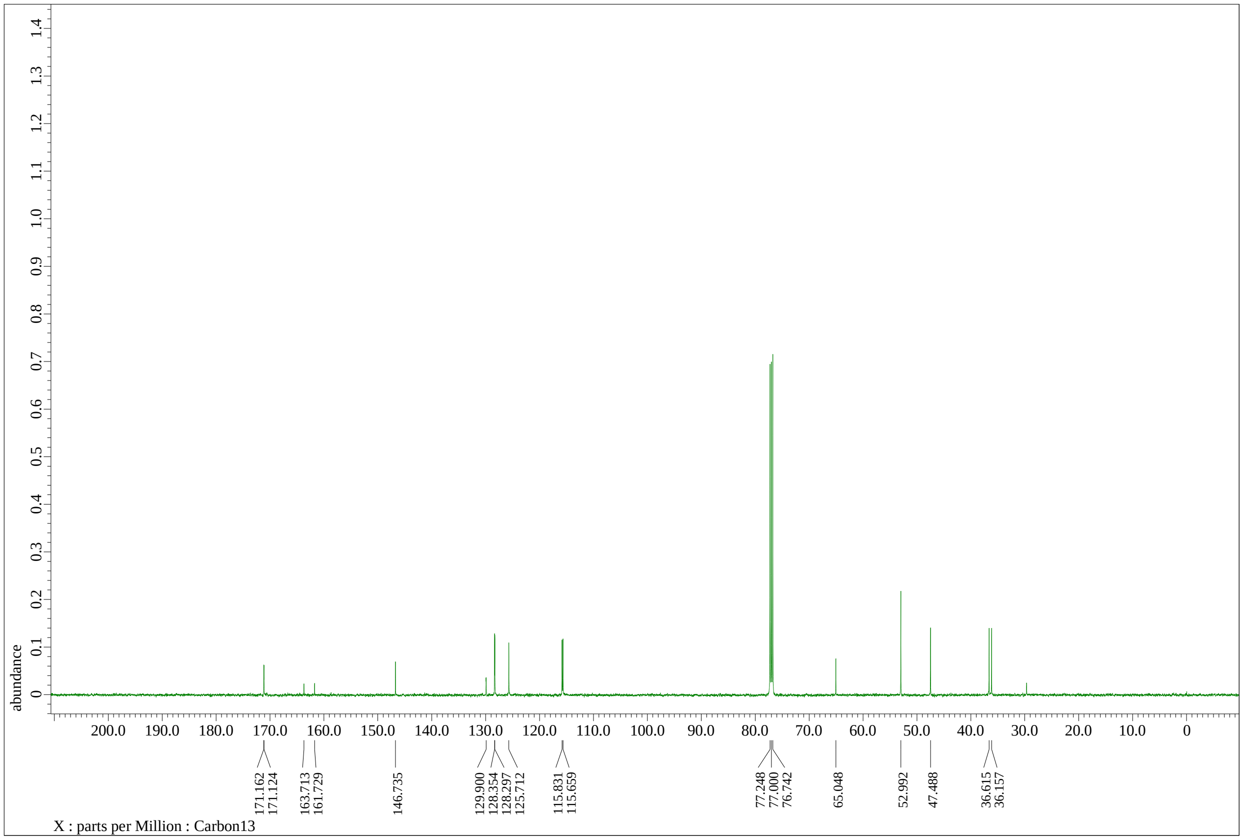

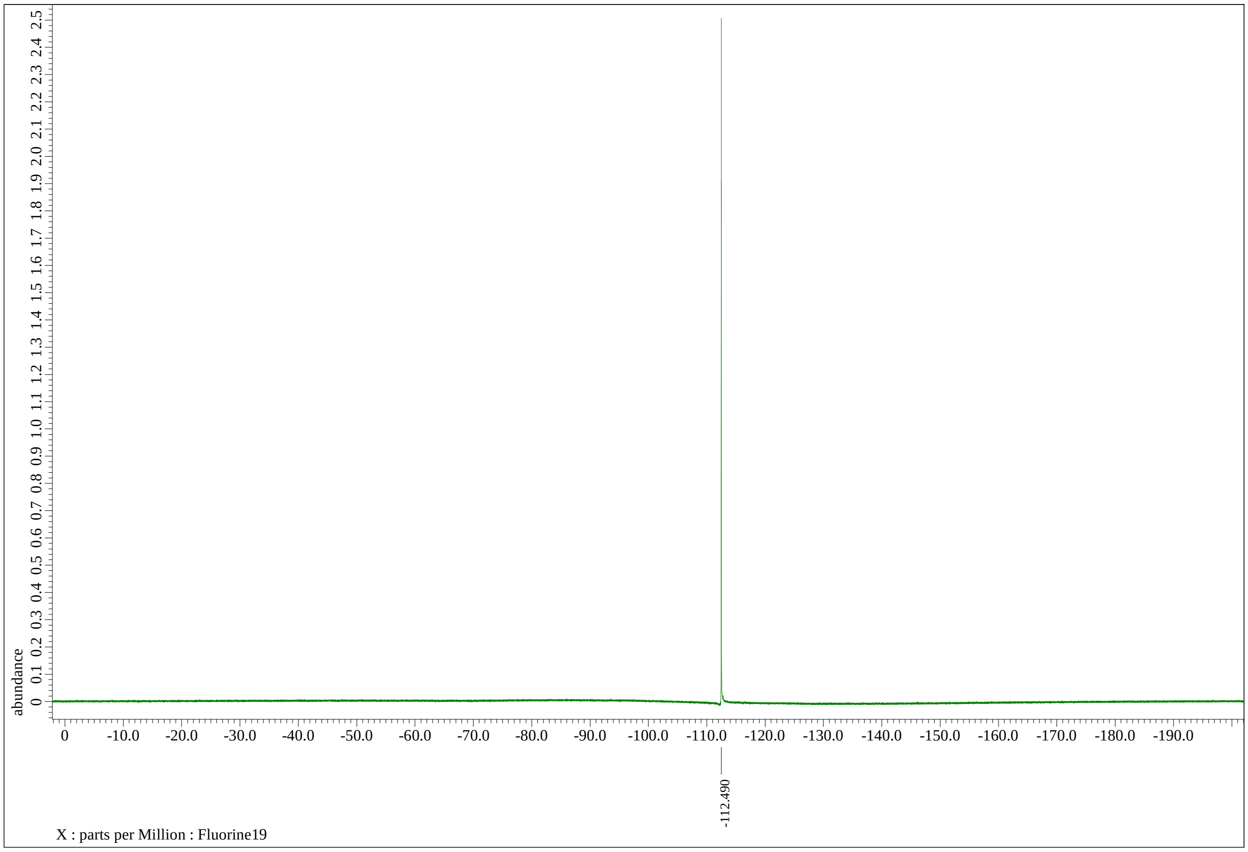
abundance
0
0.1
0.2
0.3
0.4
0.5
0.6
0.7
0.8
0.9
1.0
1.1
1.2
1.3
1.4
1.5
1.6
1.7
1.8
1.9
2.0
2.1
2.2
2.3
2.4
2.5
0
-10.0
-20.0
-30.0
-40.0
-50.0
-60.0
-70.0
-80.0
-90.0
-100.0
-110.0
-120.0
-130.0
-140.0
-150.0
-160.0
-170.0
-180.0
-190.0
-112.490
X : parts per Million : Fluorine19

- ^{1}H and ^{13}C NMR Spectra of 3f

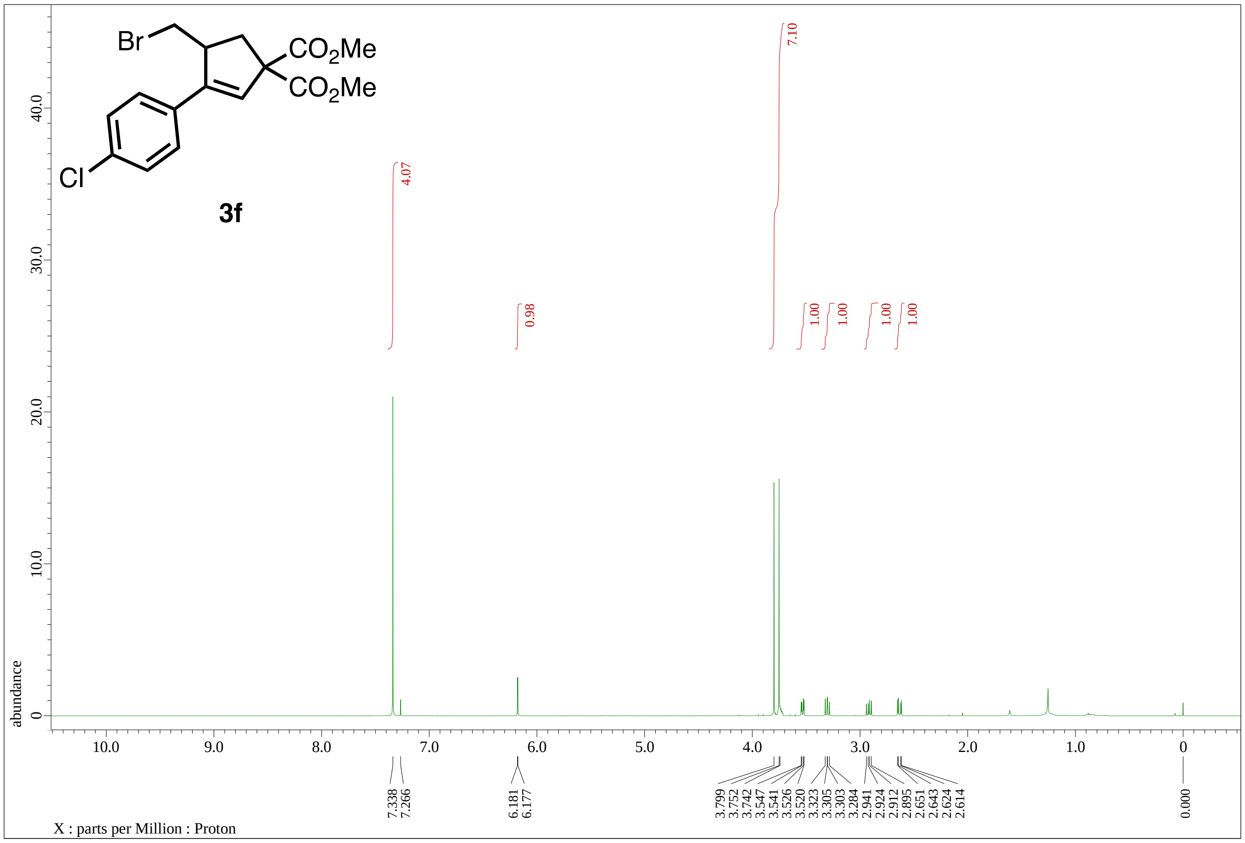

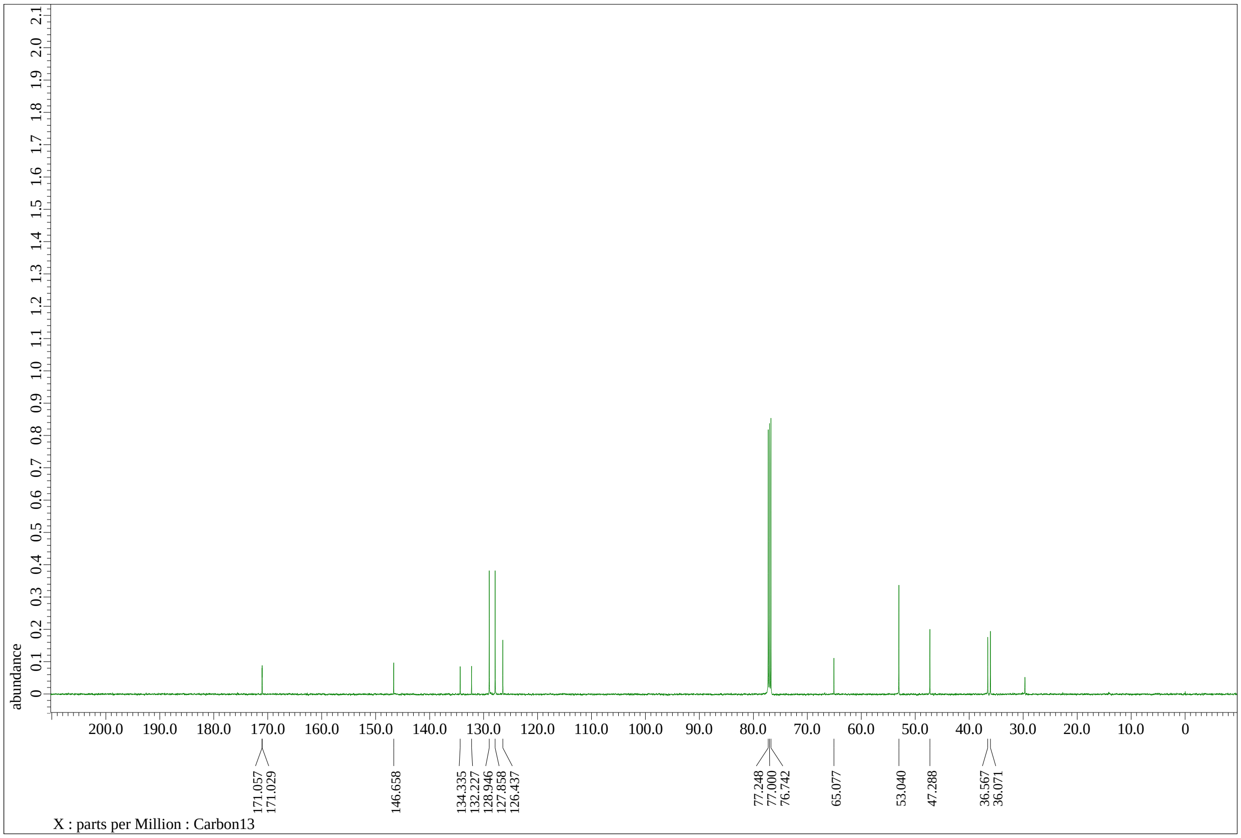

- ^{1}H and ^{13}C NMR Spectra of 3g

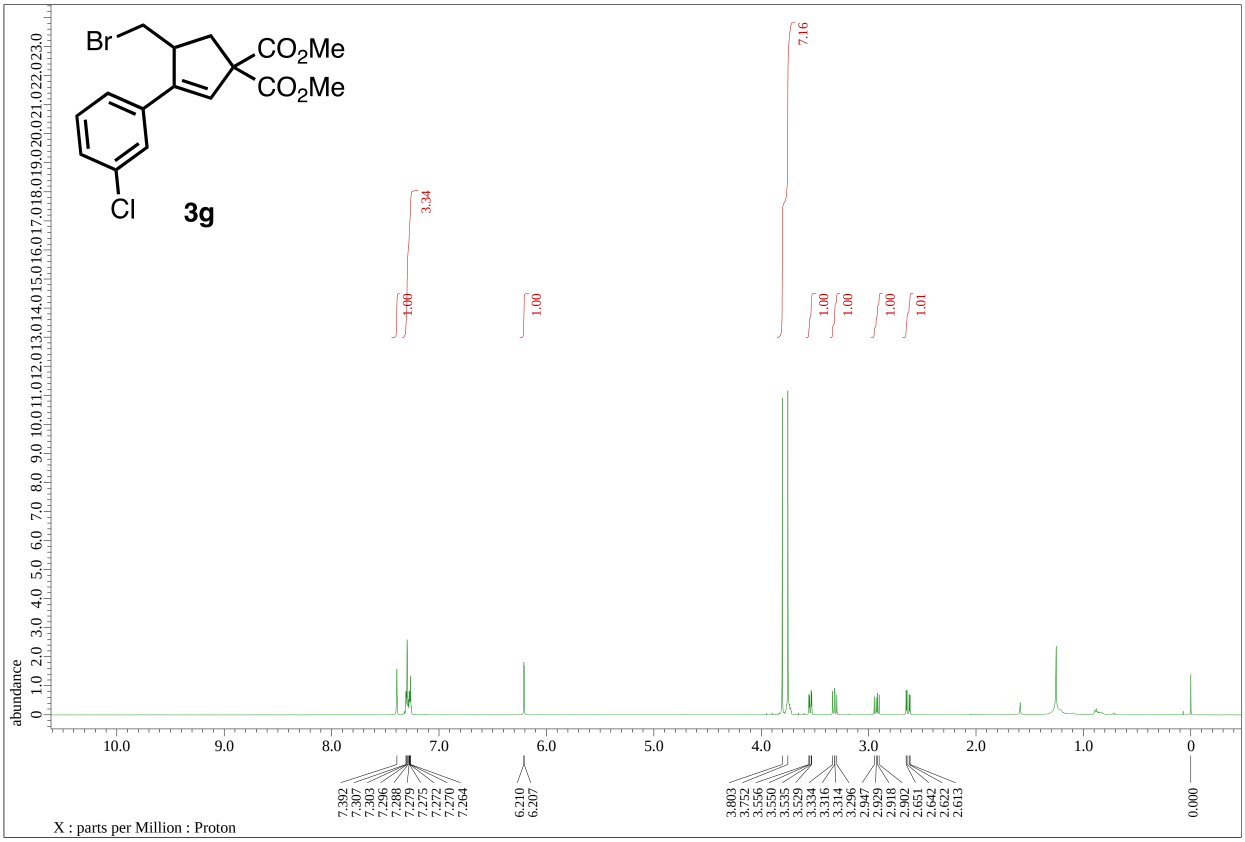

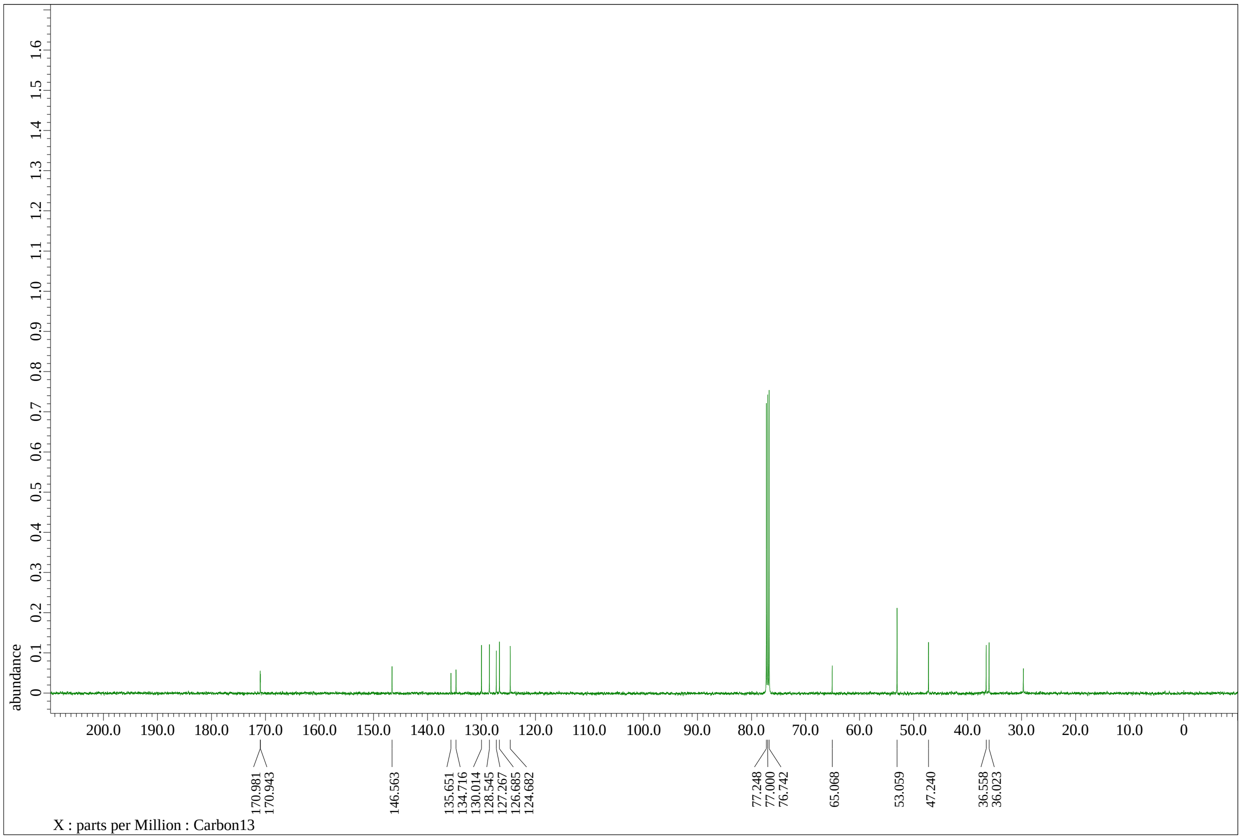

- ^{1}H and ^{13}C NMR Spectra of 3h

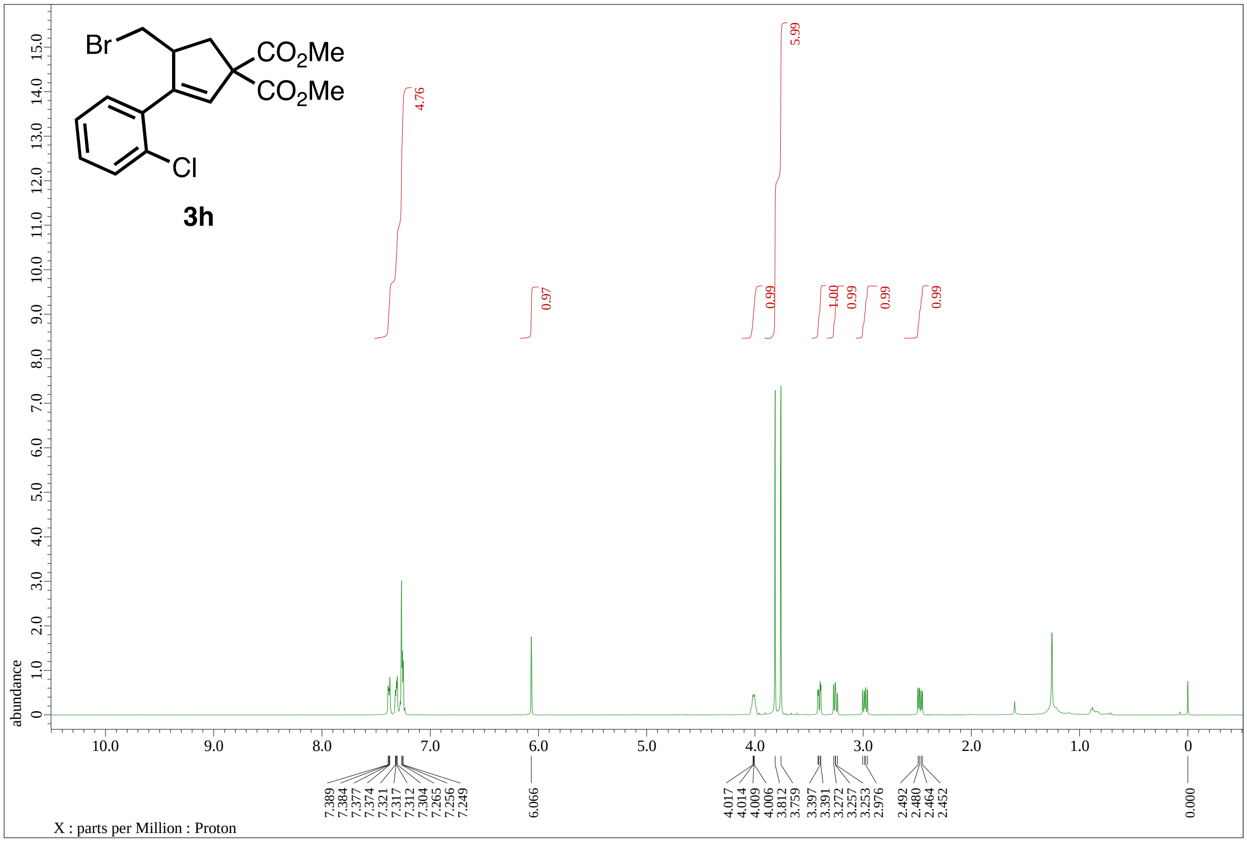

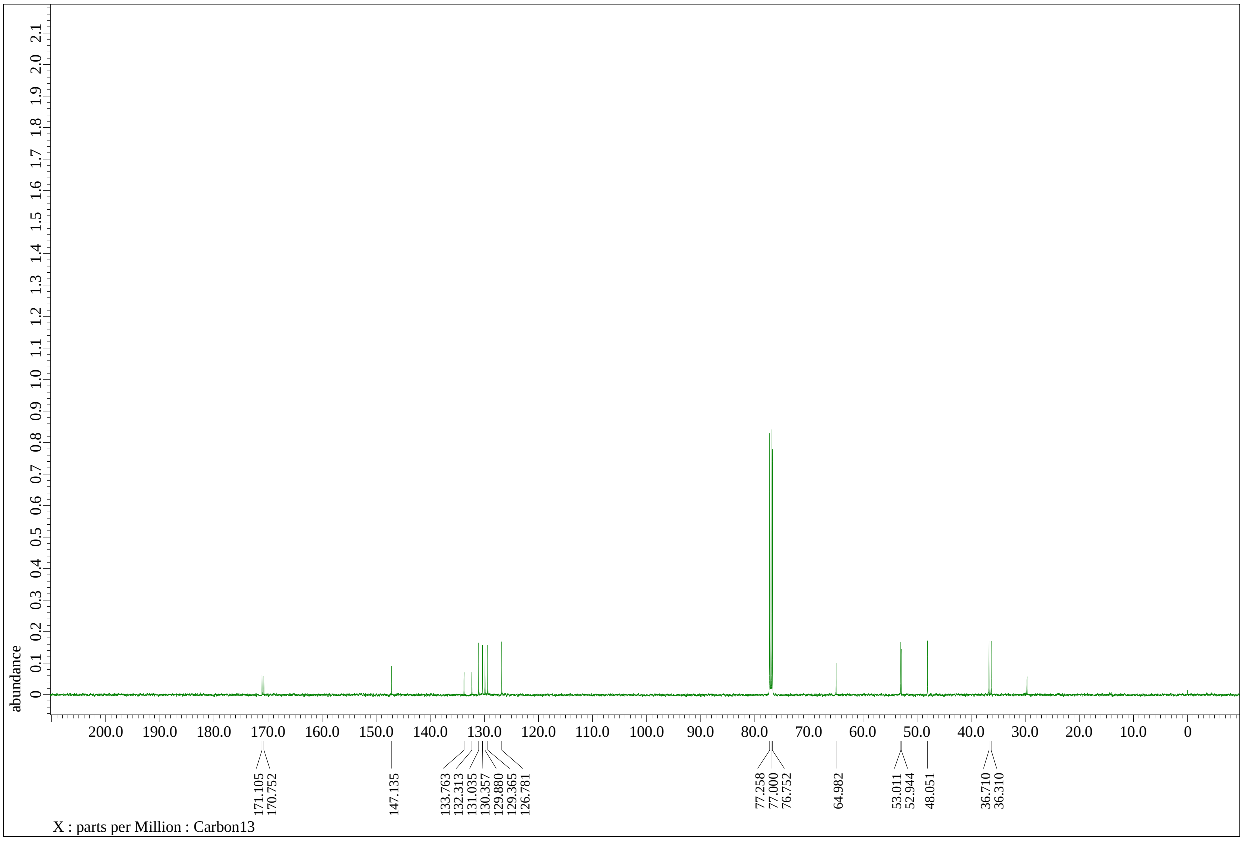

- ^{1}H and ^{13}C NMR Spectra of 3i

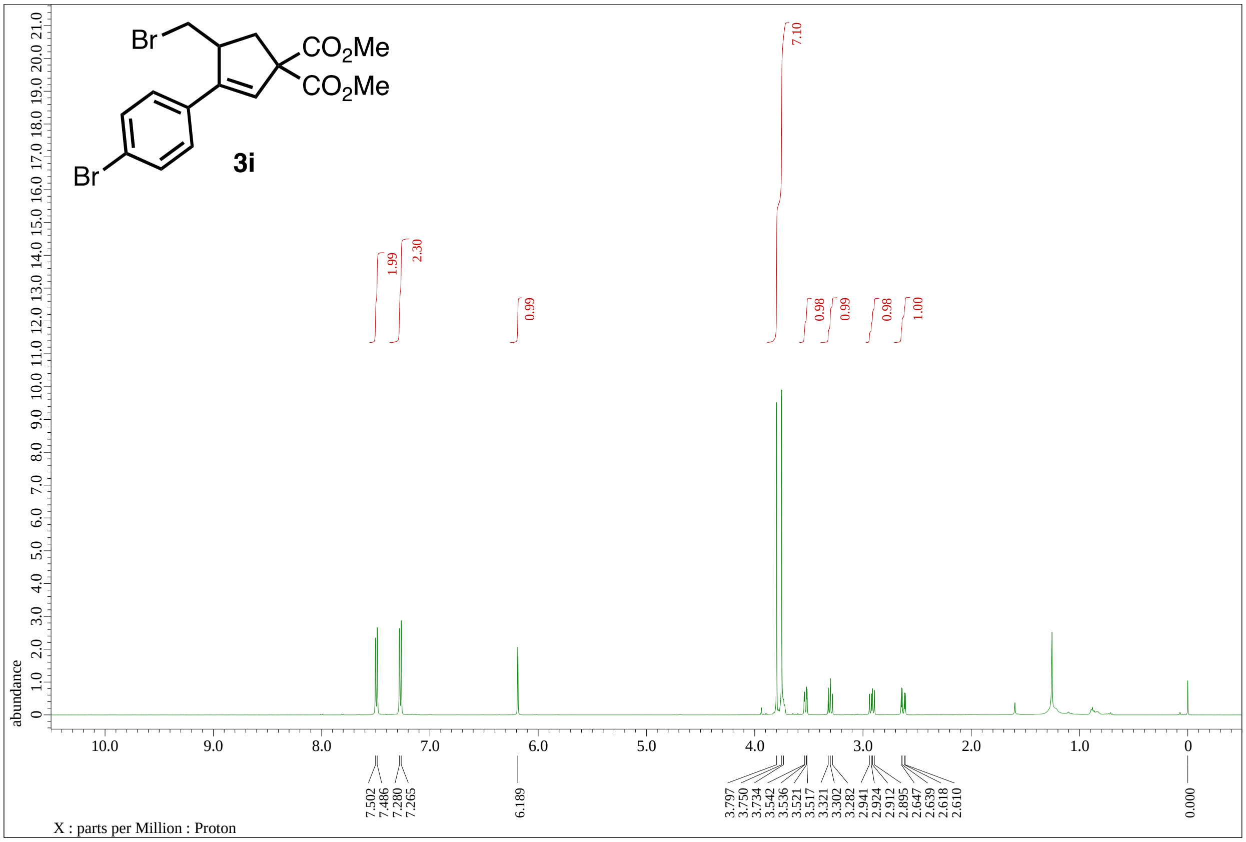

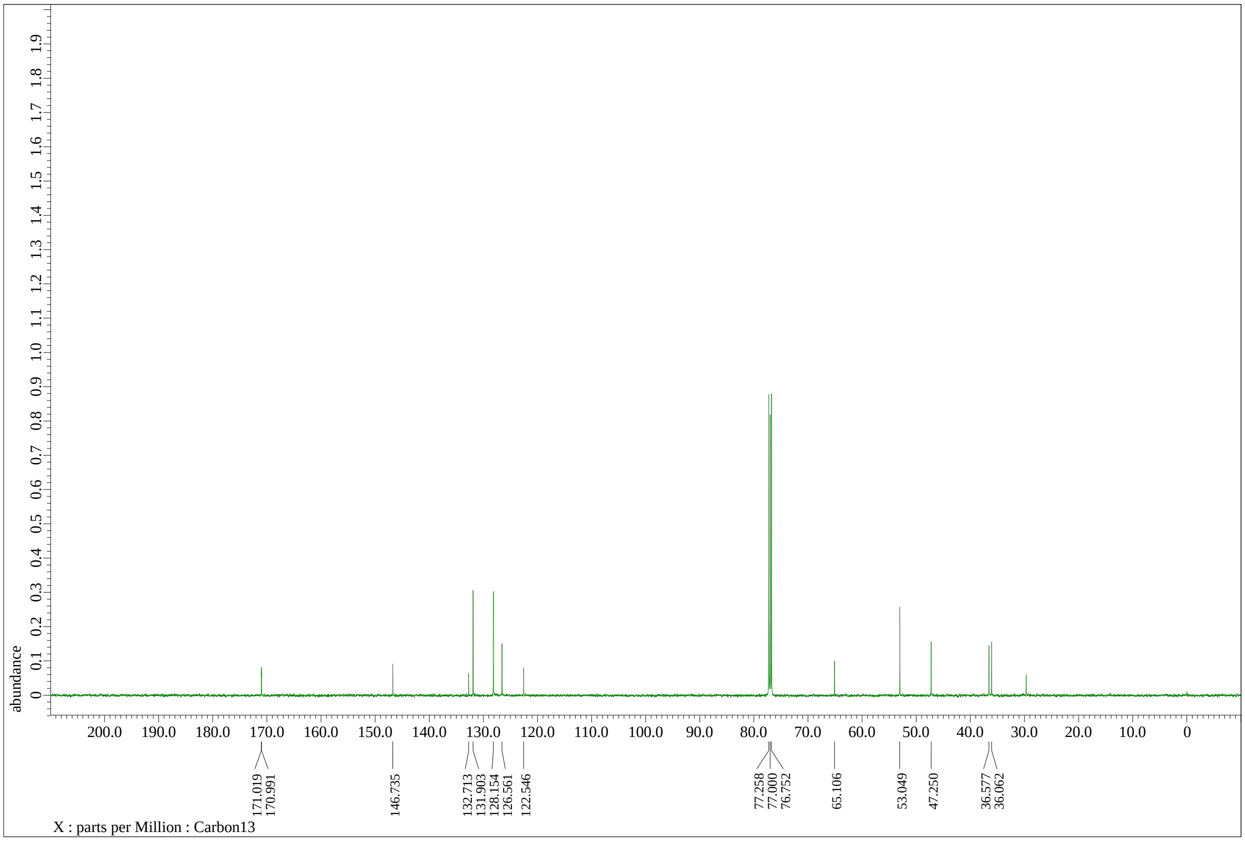

- ^{1}H, ^{13}C and ^{19}F NMR Spectra of 3j

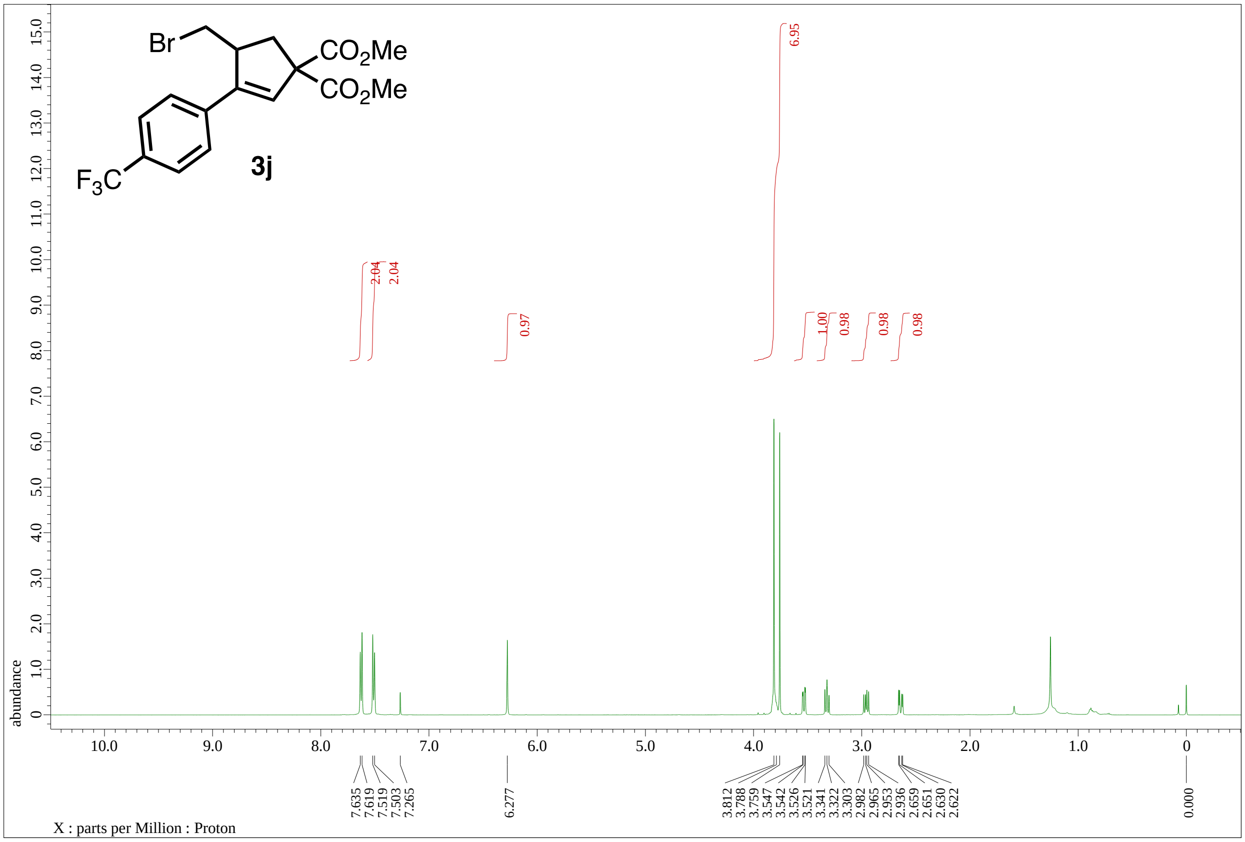

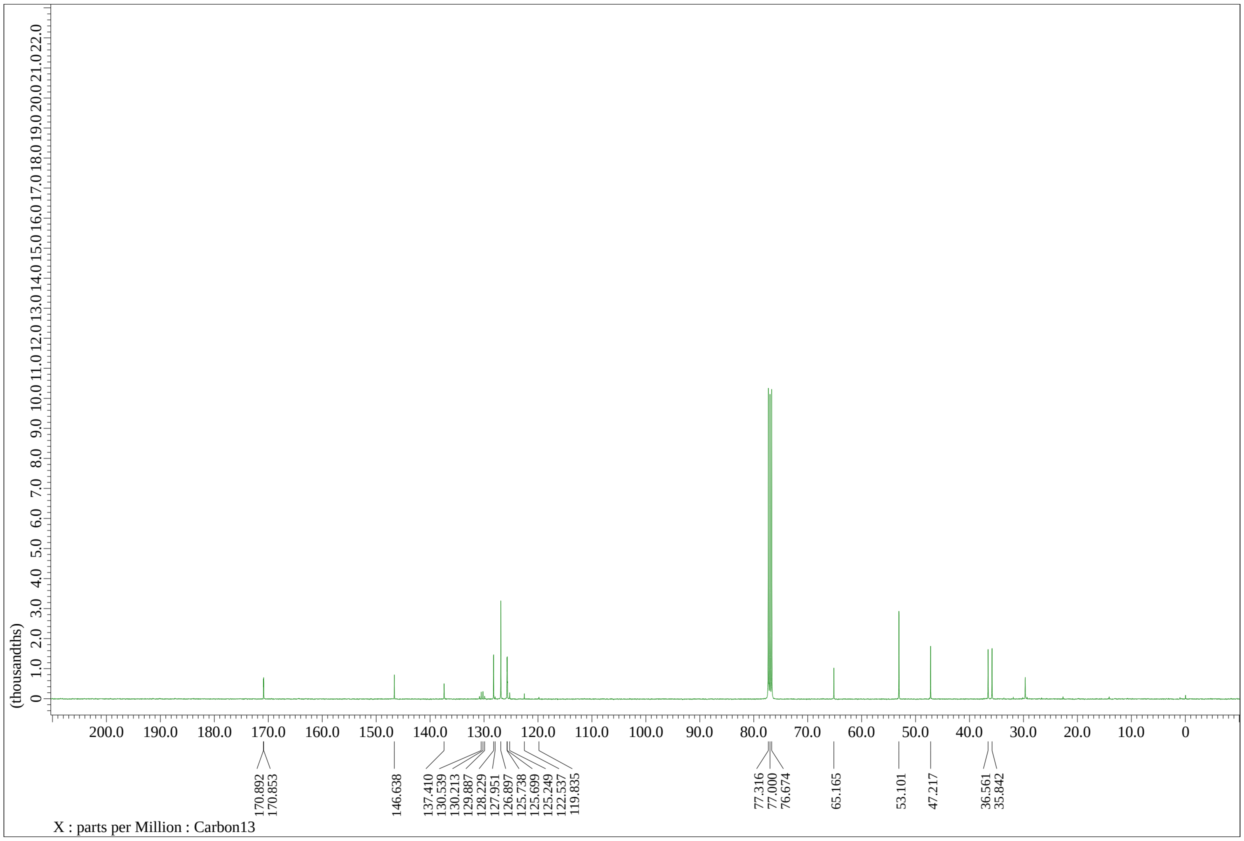

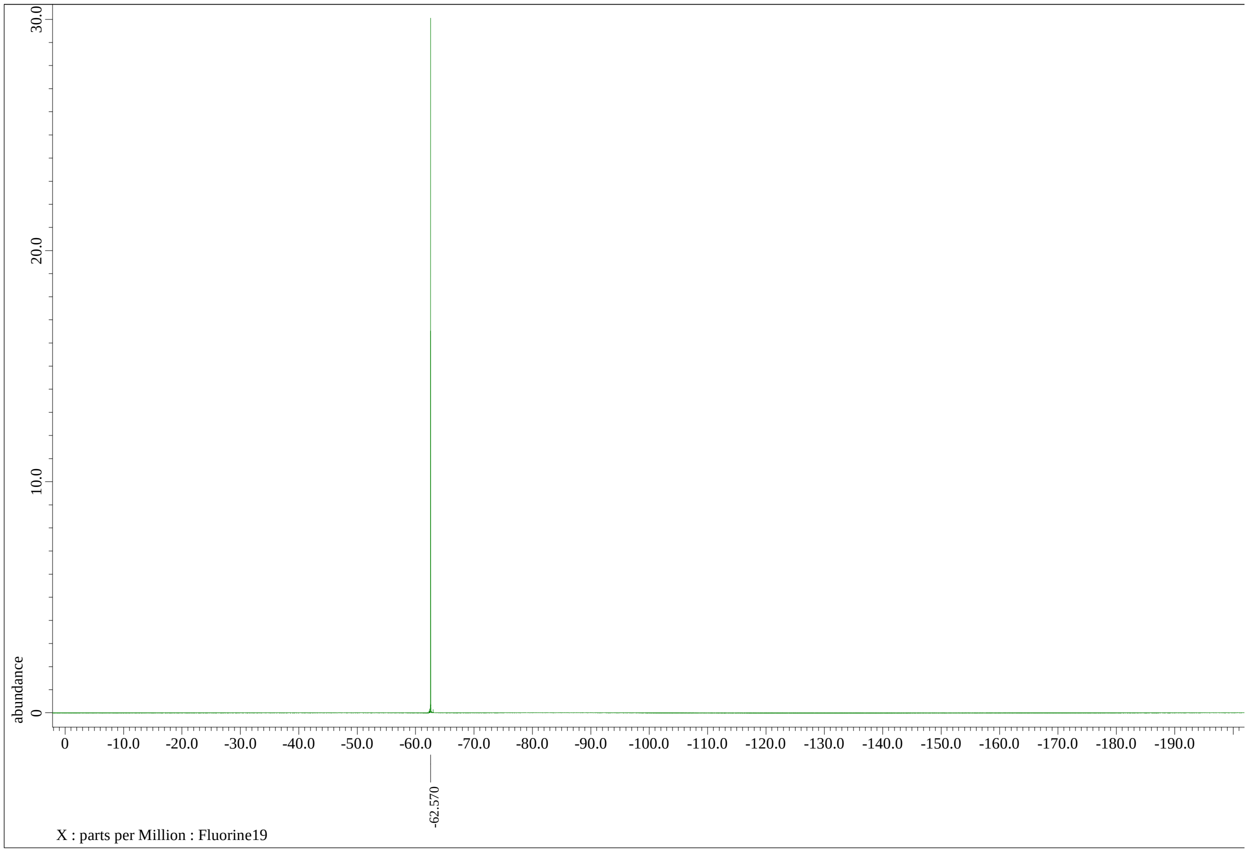

30.0
20.0
10.0
0
abundance
0 -10.0 -20.0 -30.0 -40.0 -50.0 -60.0 -70.0 -80.0 -90.0 -100.0 -110.0 -120.0 -130.0 -140.0 -150.0 -160.0 -170.0 -180.0 -190.0
-62.570
X : parts per Million : Fluorine19

- ^{1}H, ^{13}C and ^{19}F NMR Spectra of 3k

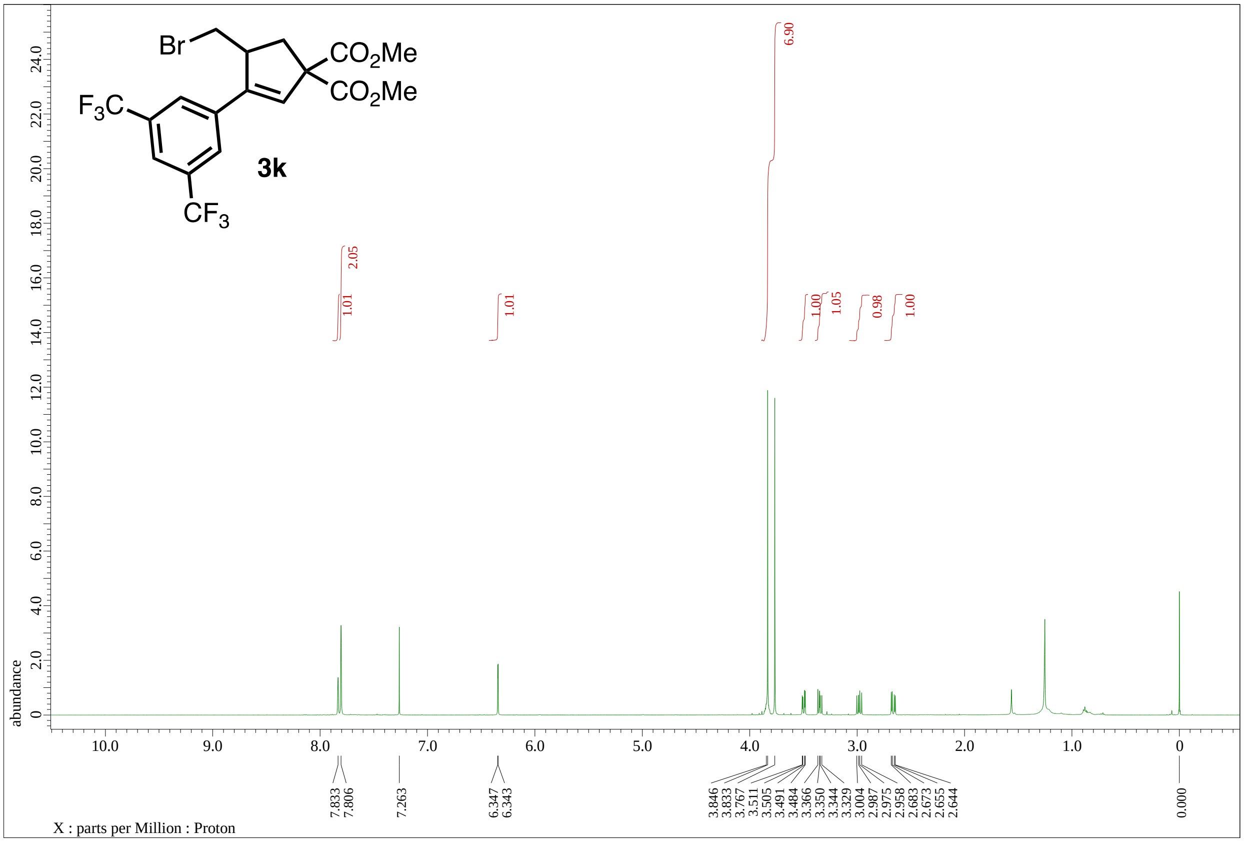

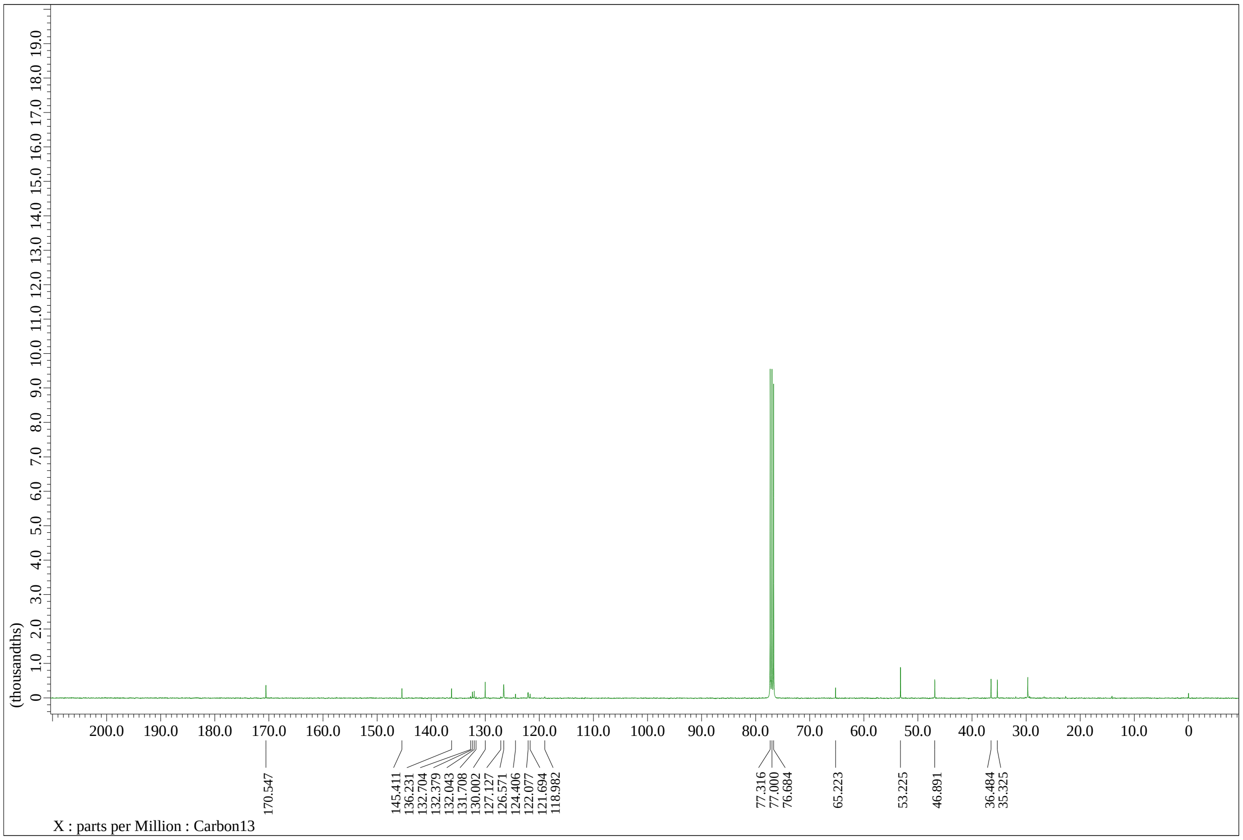

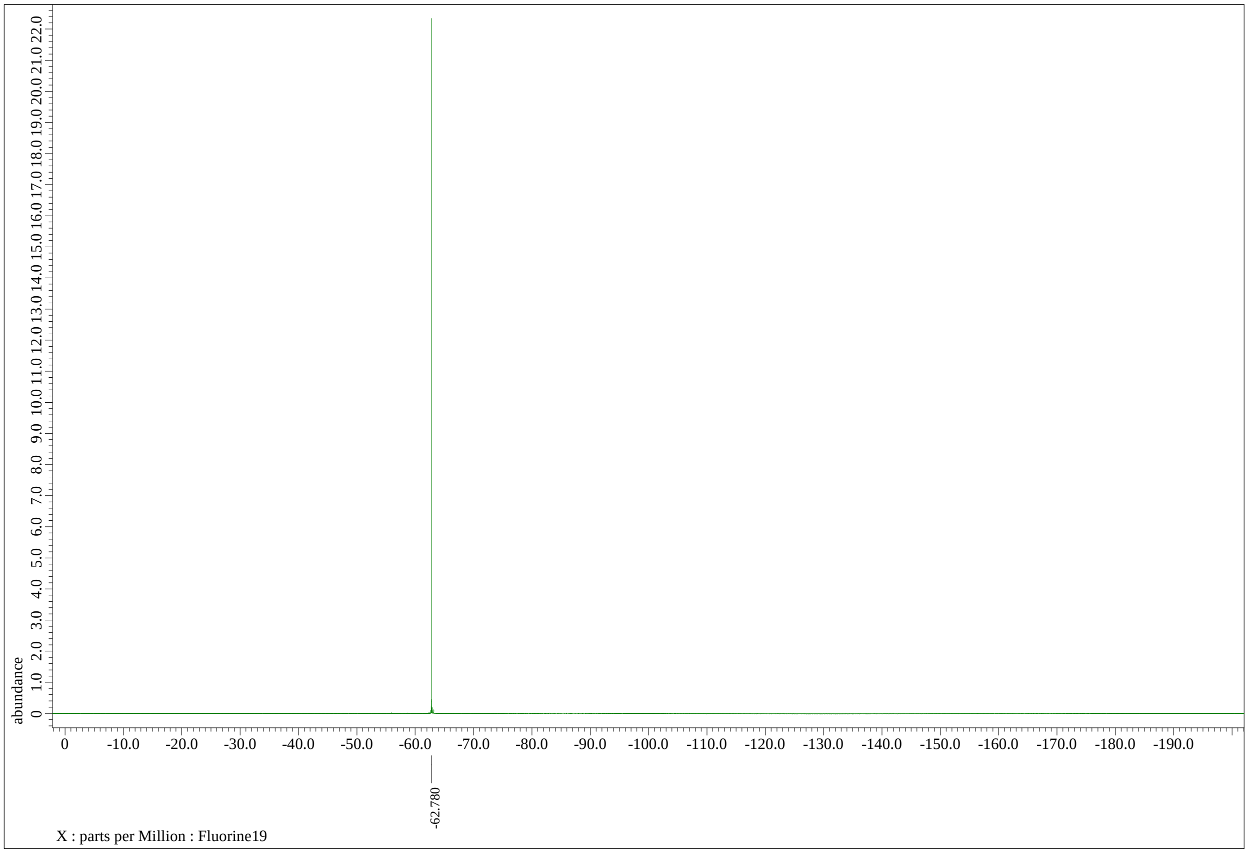
abundance
0
1.0
2.0
3.0
4.0
5.0
6.0
7.0
8.0
9.0
10.0
11.0
12.0
13.0
14.0
15.0
16.0
17.0
18.0
19.0
20.0
21.0
22.0
0
-10.0
-20.0
-30.0
-40.0
-50.0
-60.0
-70.0
-80.0
-90.0
-100.0
-110.0
-120.0
-130.0
-140.0
-150.0
-160.0
-170.0
-180.0
-190.0
-62.780
X : parts per Million : Fluorine19

- ^{1}H and ^{13}C NMR Spectra of 3l

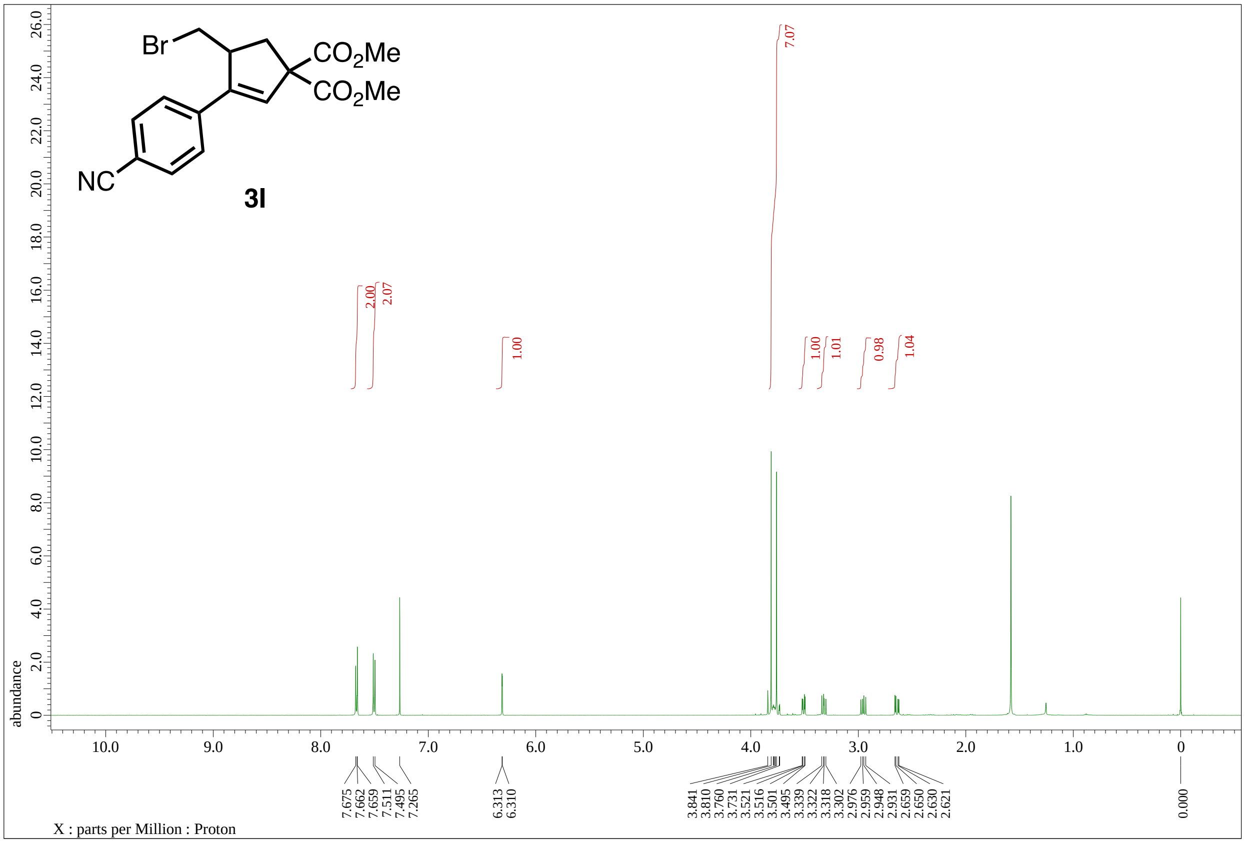

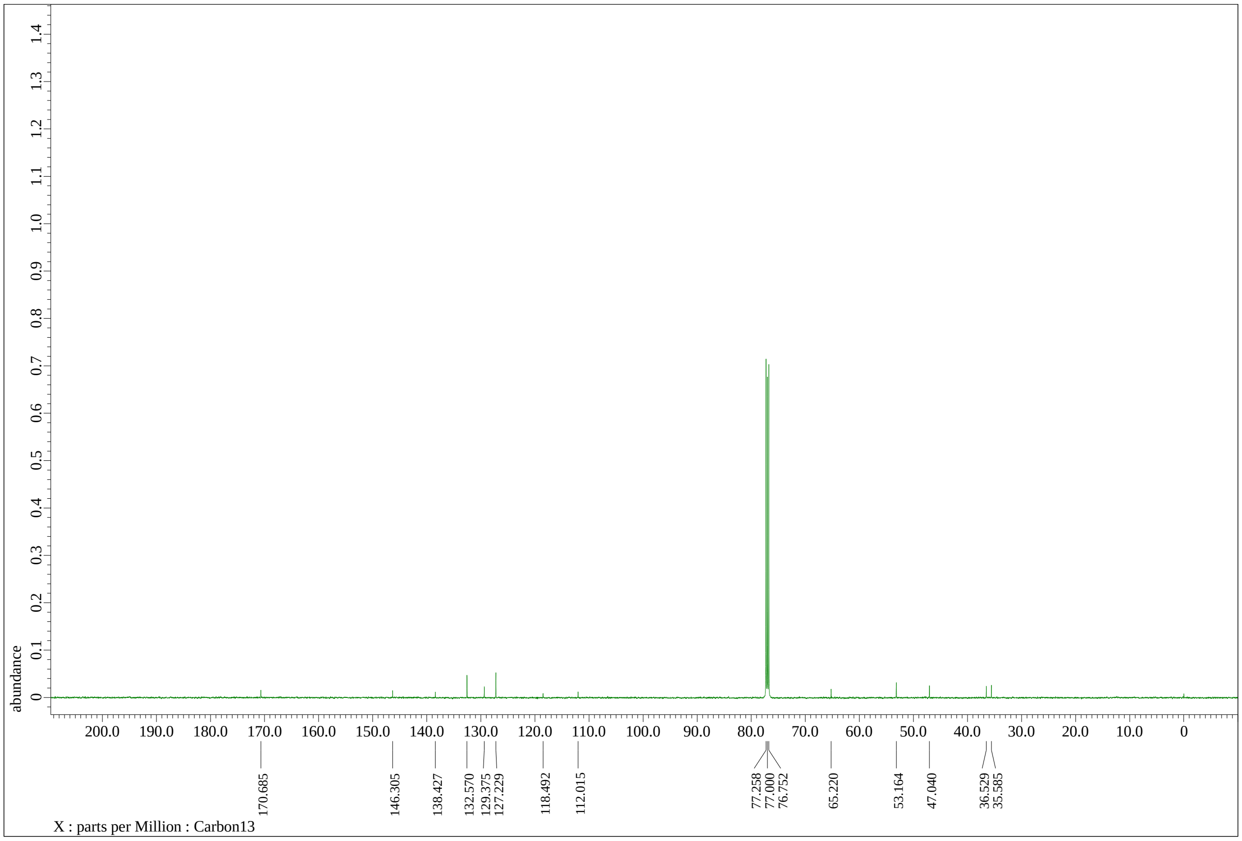

- ^1H and ^{13}C NMR Spectra of 3m

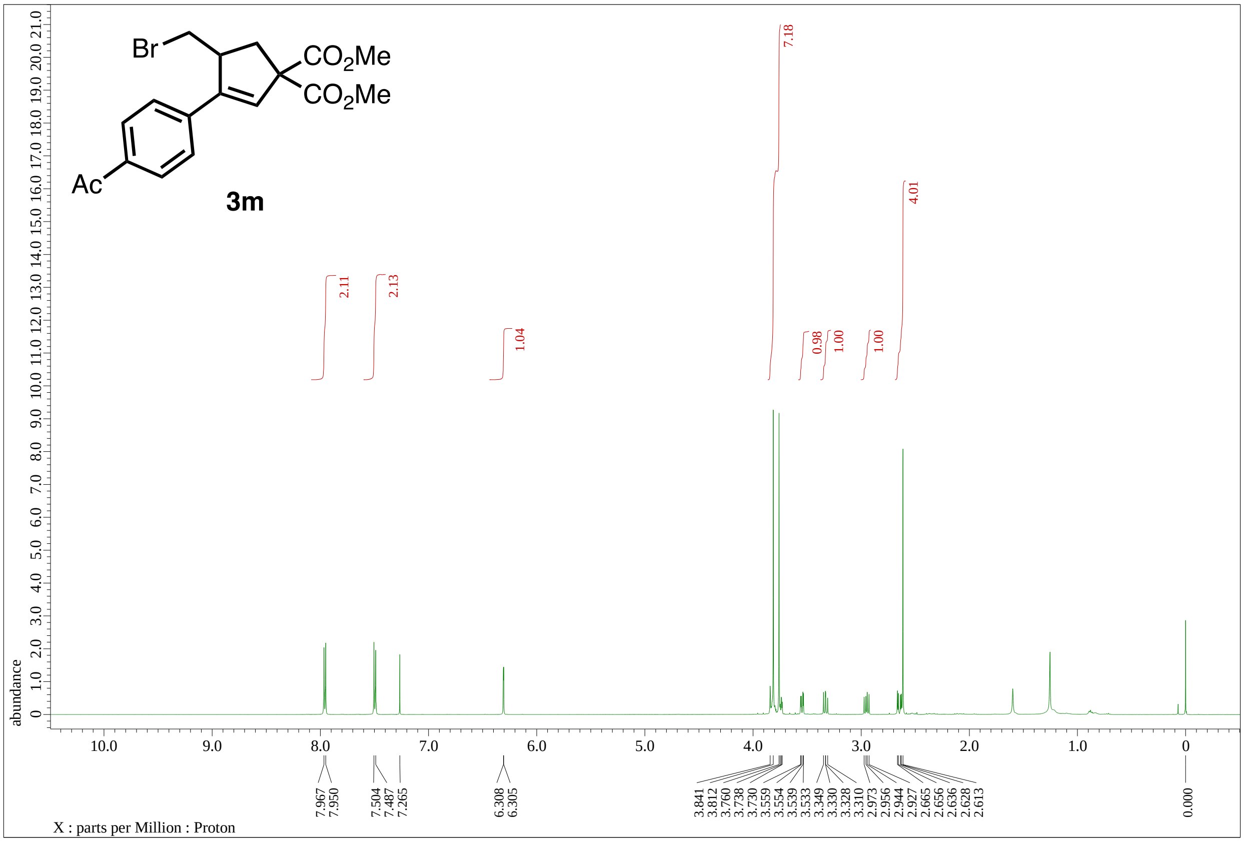

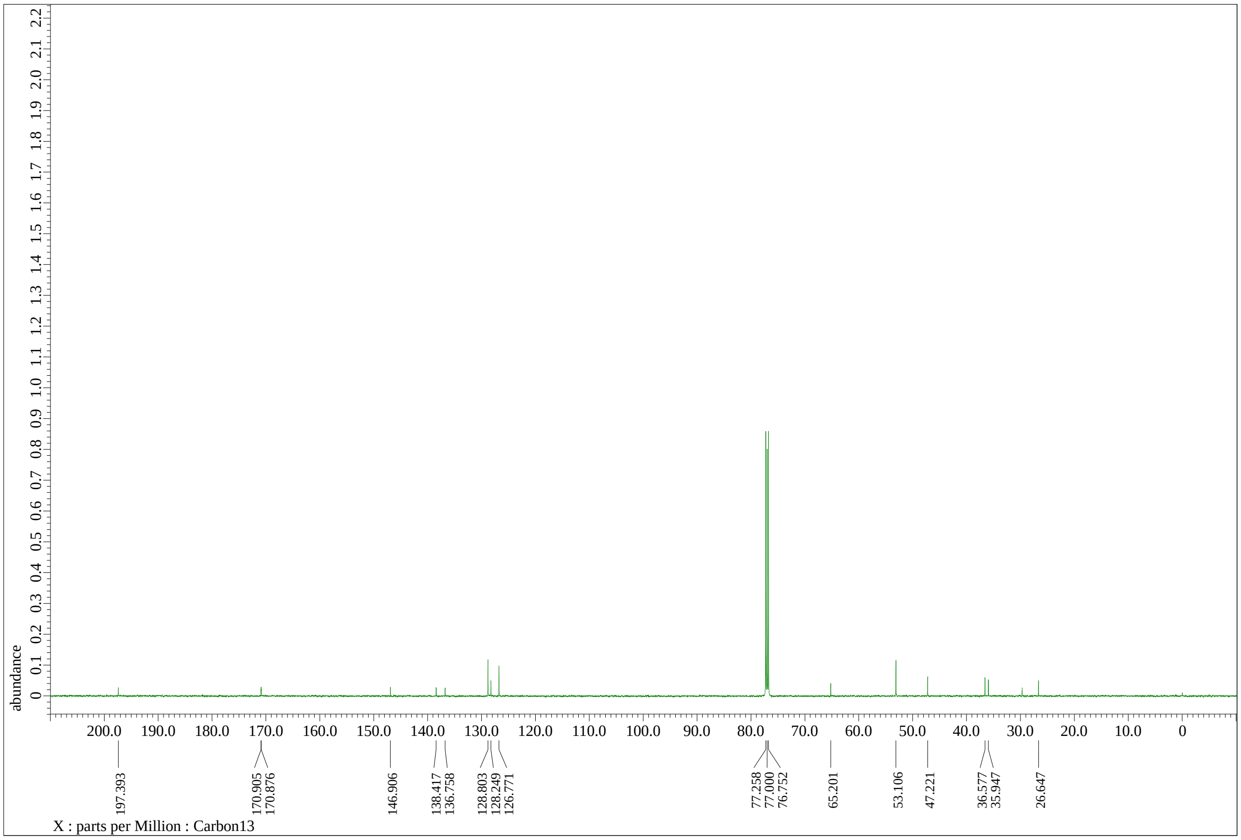

- ^{1}H and ^{13}C NMR Spectra of 3n

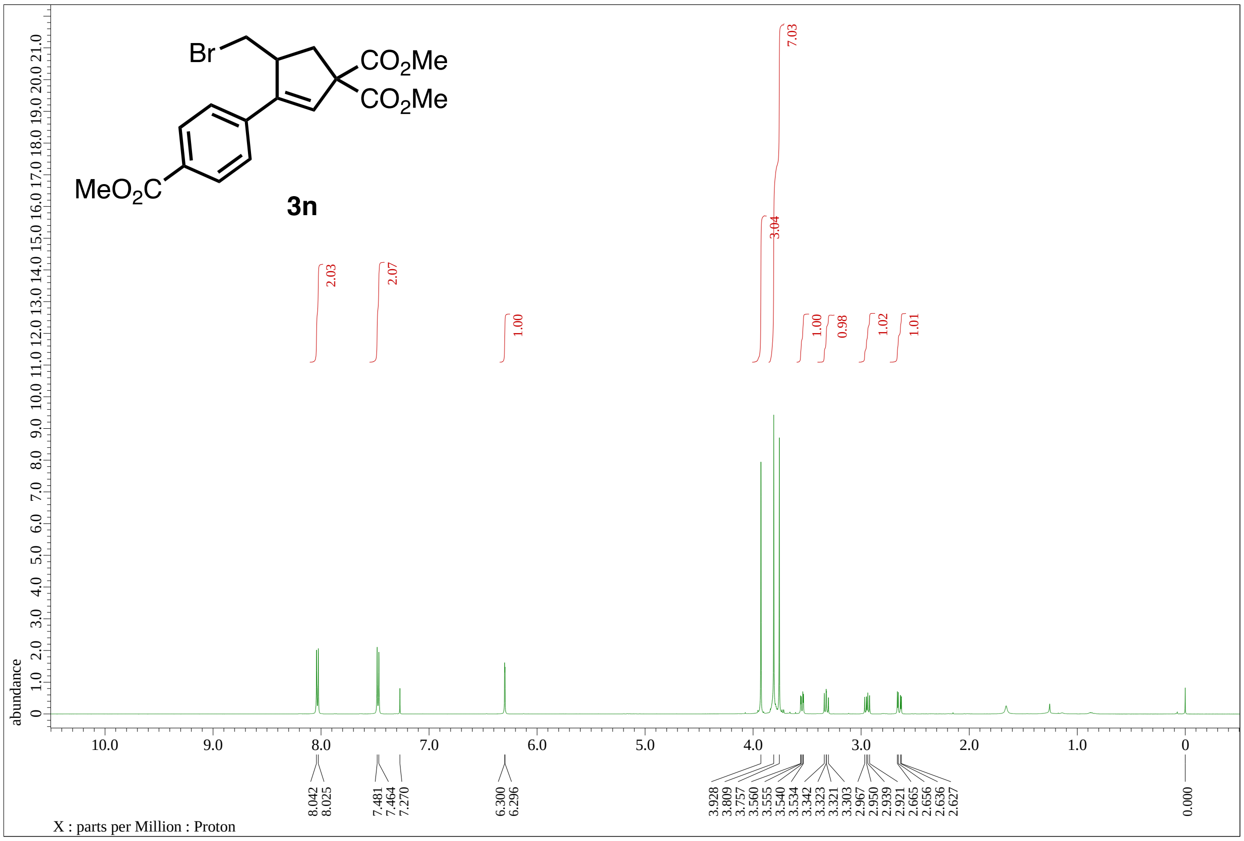

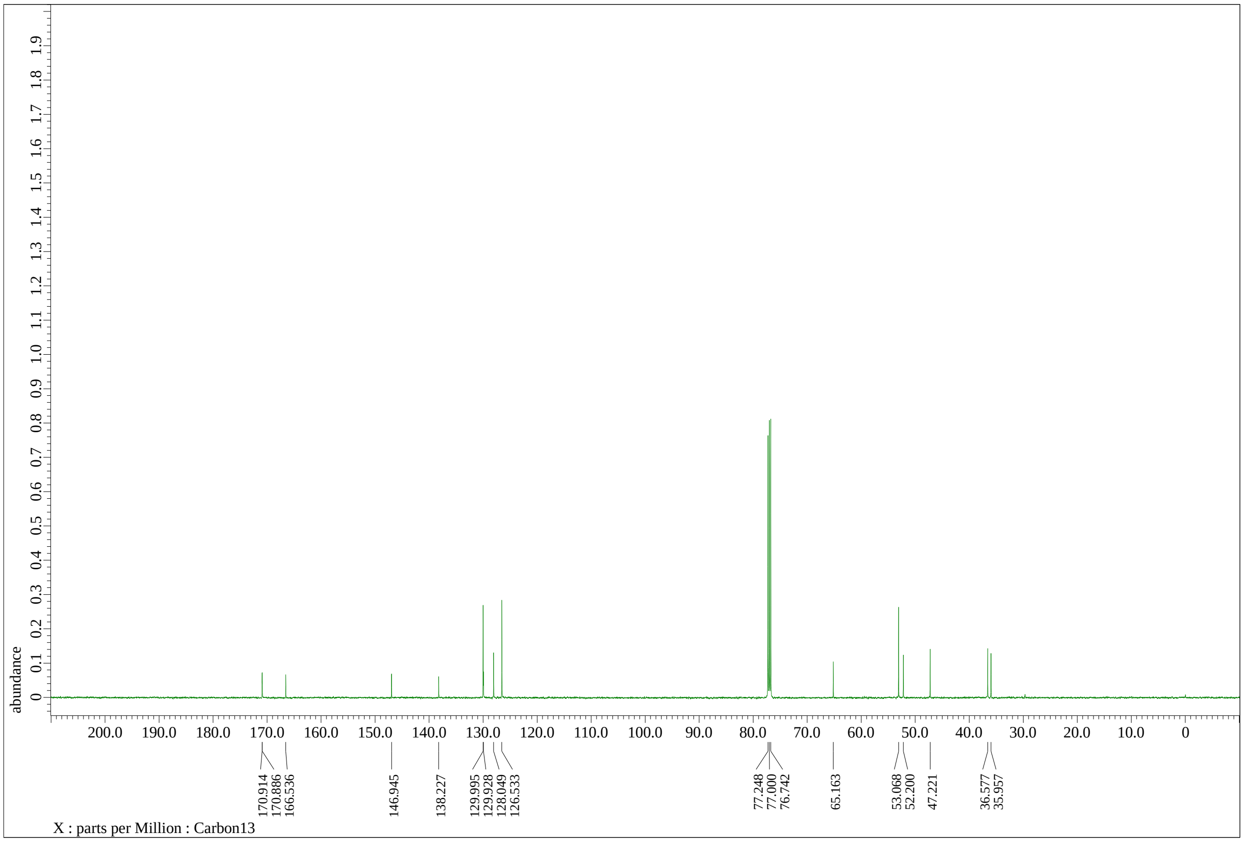

- ^{1}H and ^{13}C NMR Spectra of 3o

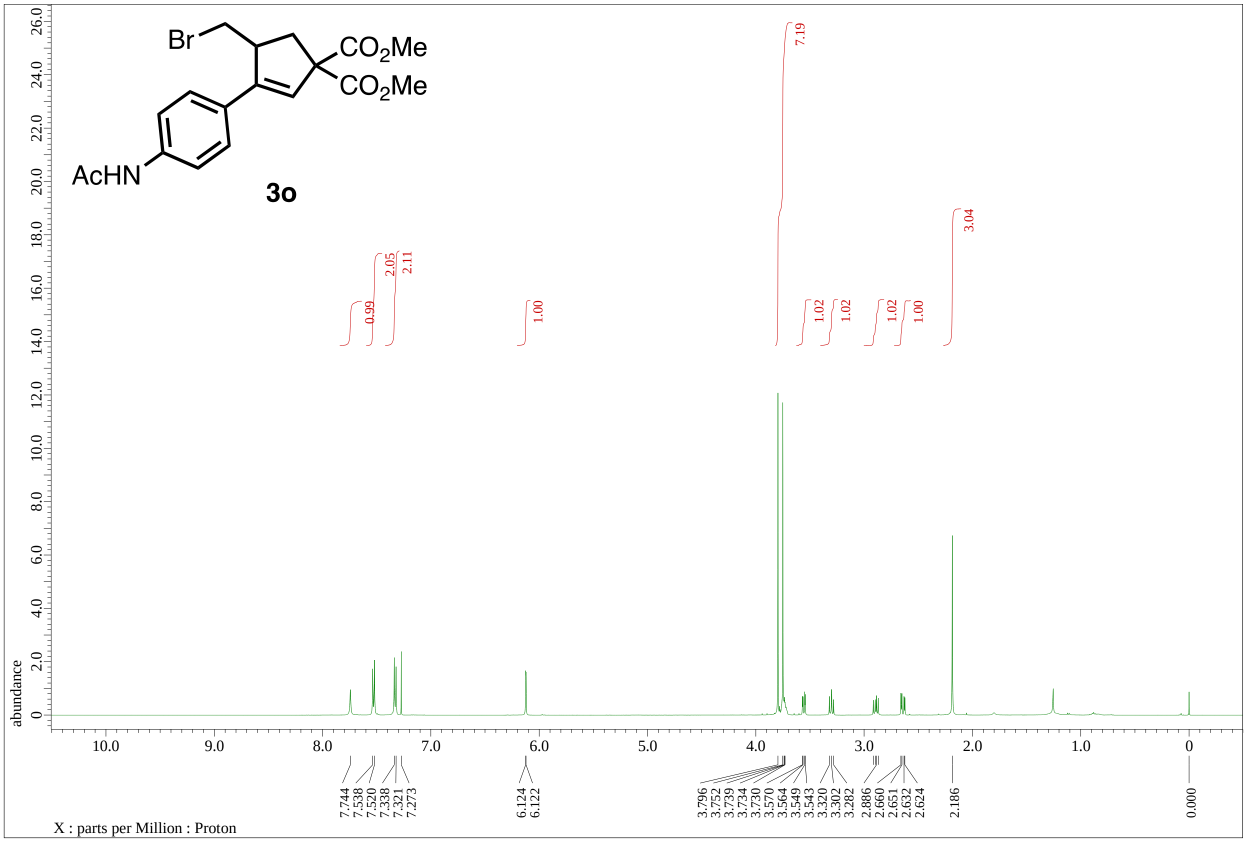

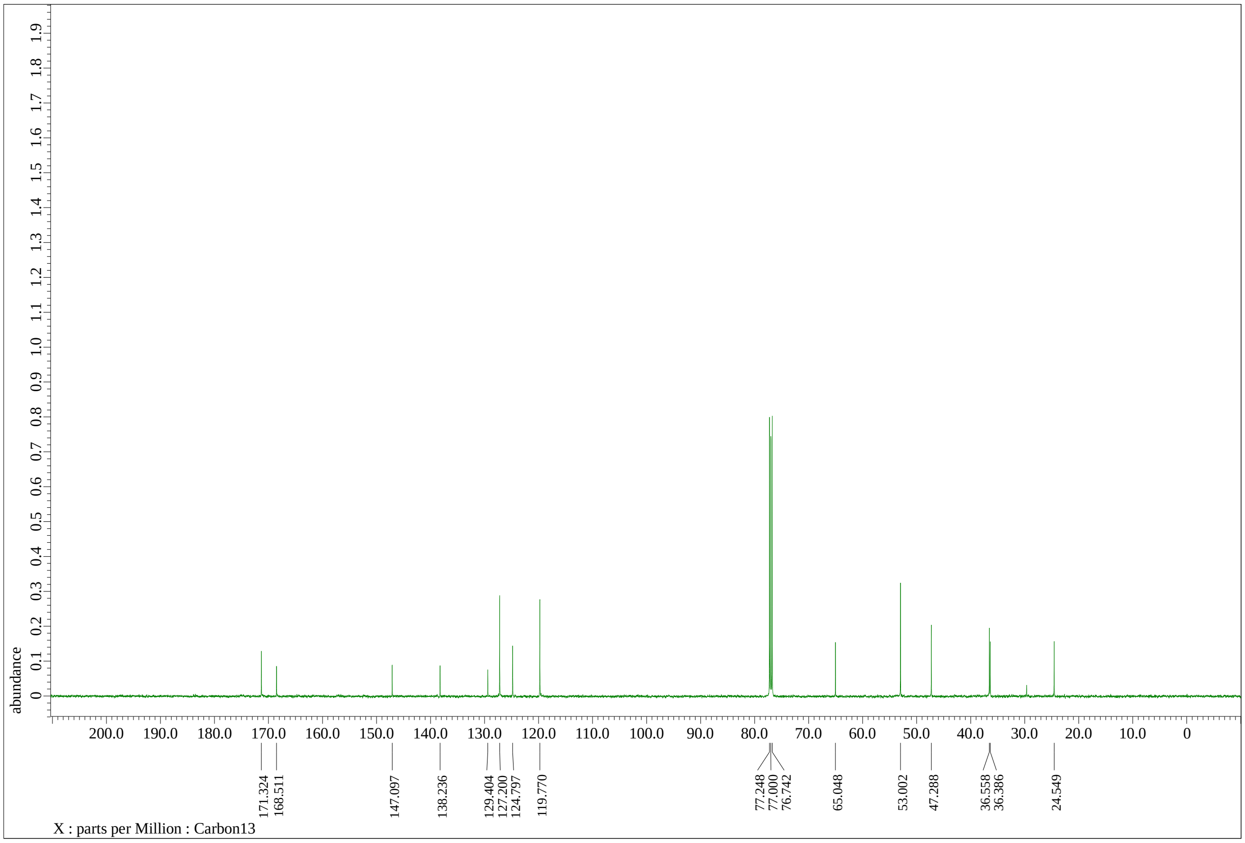

- ^{1}H, ^{13}C and DEPT-135 NMR Spectra of 3p

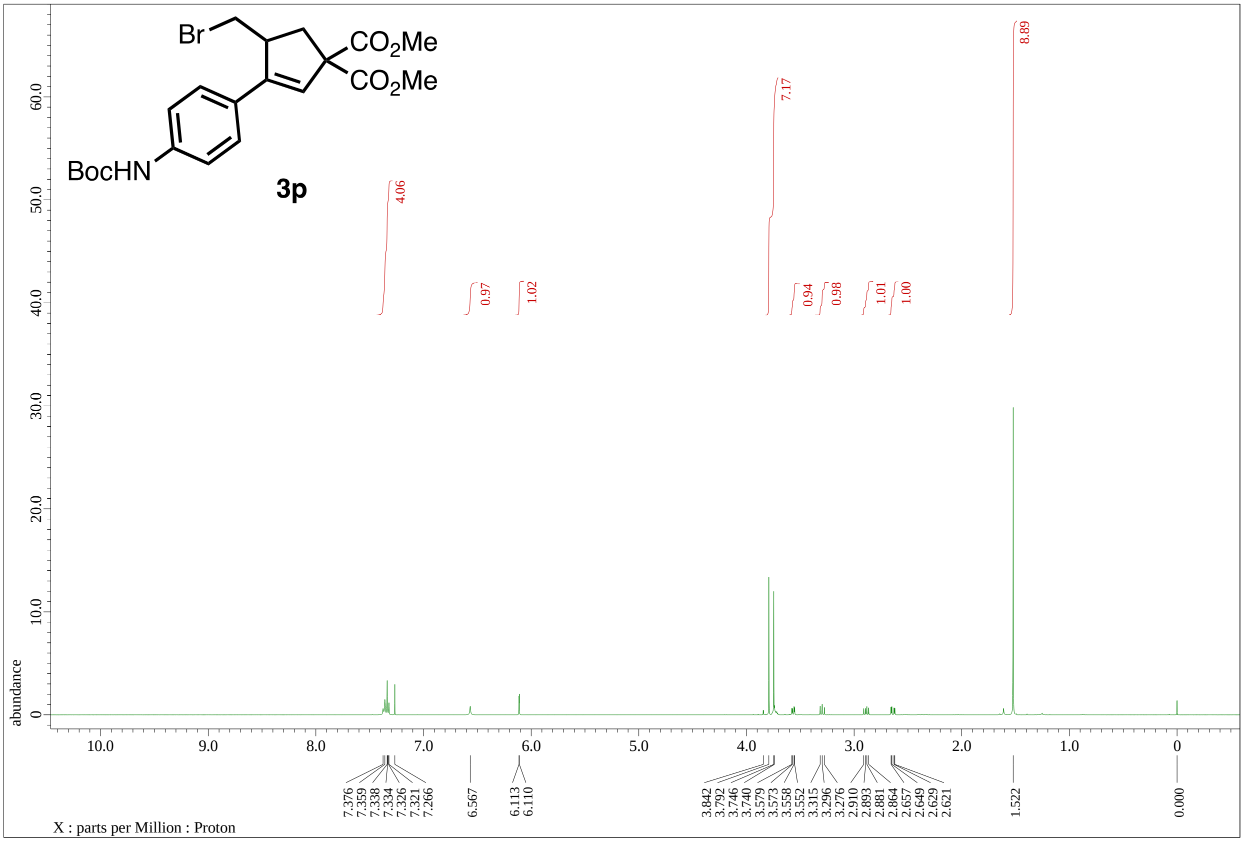

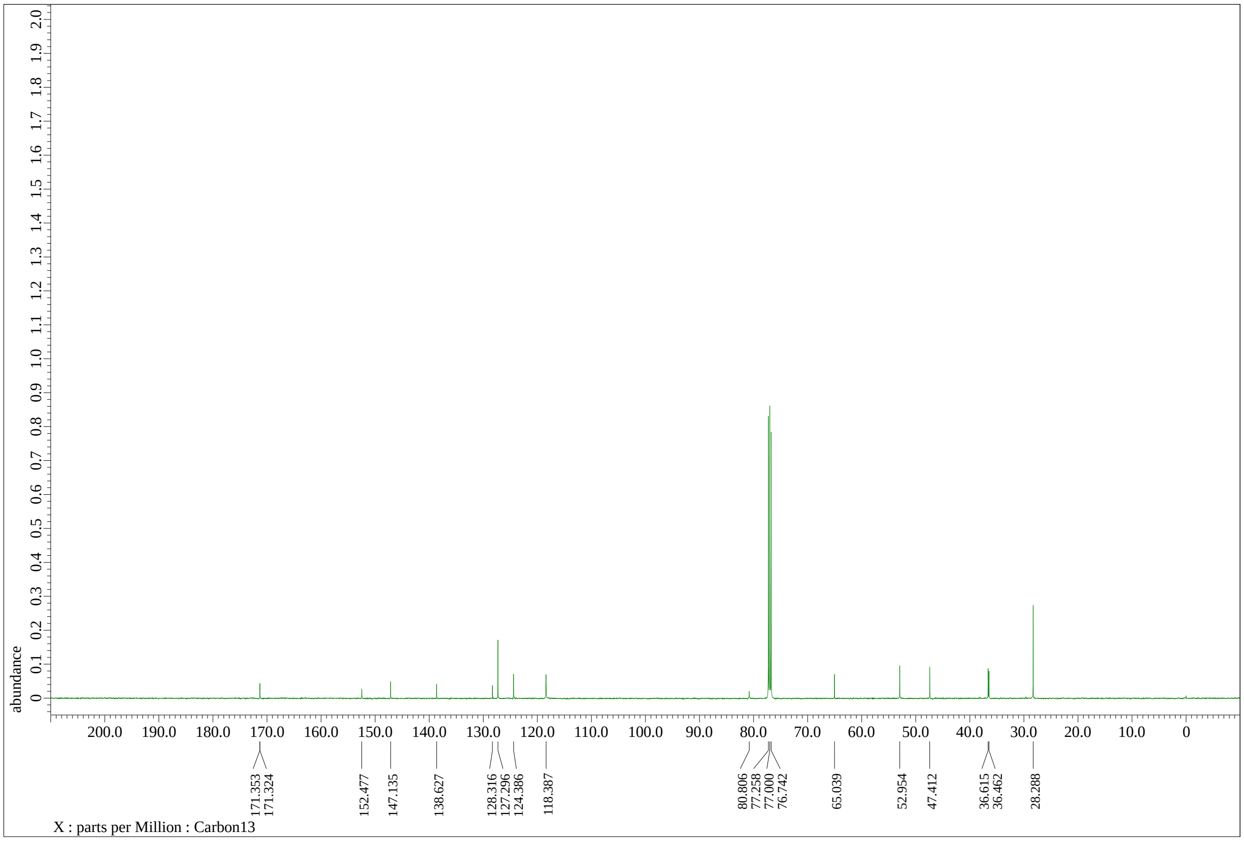

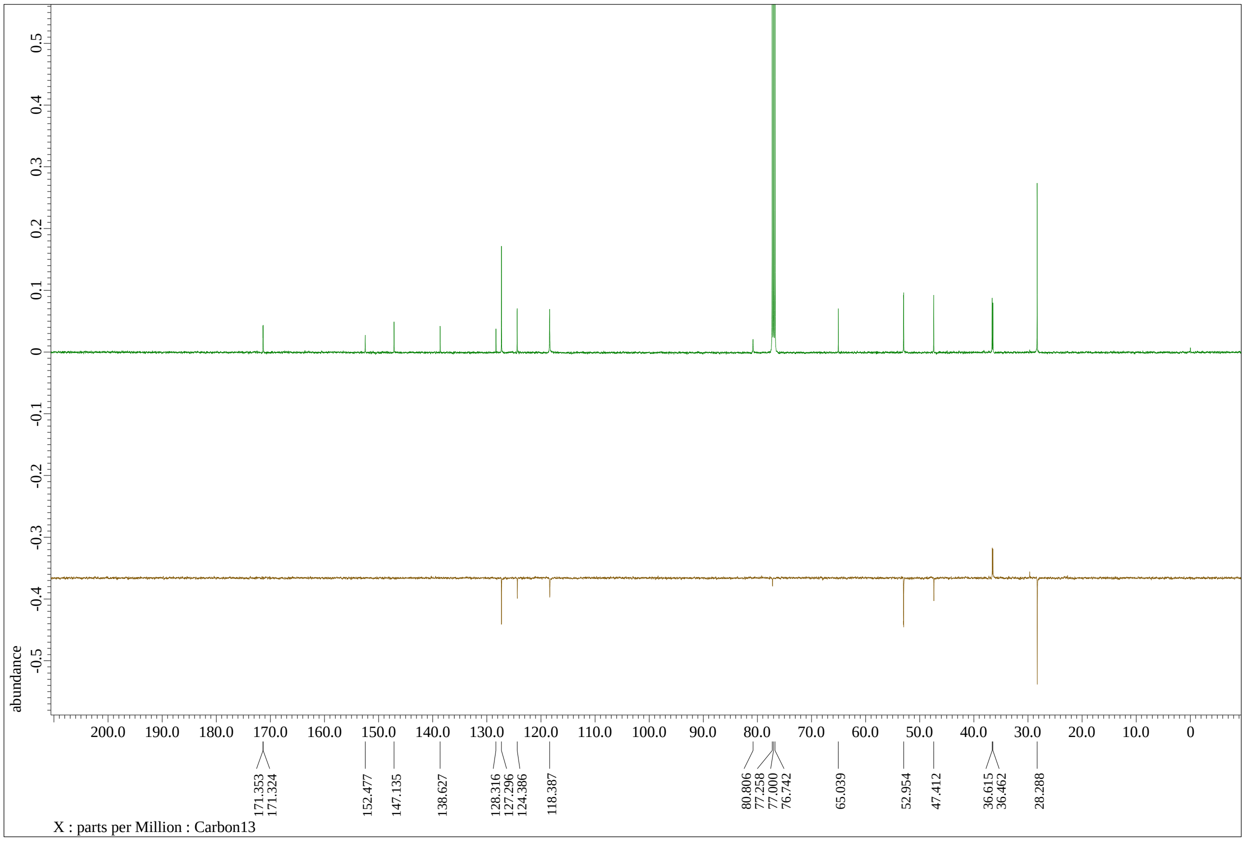

abundance
0.5
0.4
0.3
0.2
0.1
0
-0.1
-0.2
-0.3
-0.4
-0.5
200.0
190.0
180.0
170.0
160.0
150.0
140.0
130.0
120.0
110.0
100.0
90.0
80.0
70.0
60.0
50.0
40.0
30.0
20.0
10.0
0
171.353
171.324
152.477
147.135
138.627
128.316
127.296
124.386
118.387
80.806
77.258
77.000
76.742
65.039
52.954
47.412
36.615
36.462
28.288
X : parts per Million : Carbon13

- ^{1}H and ^{13}C NMR Spectra of 3q

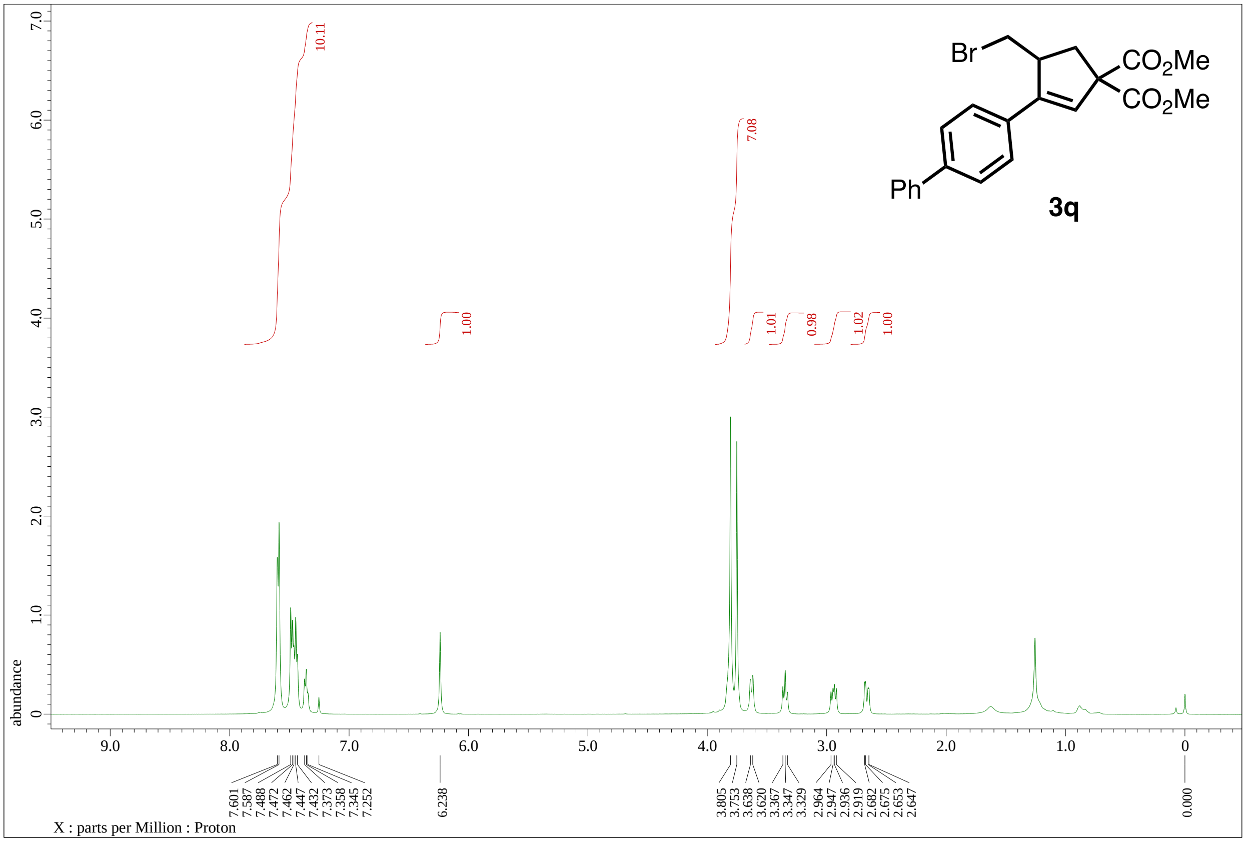

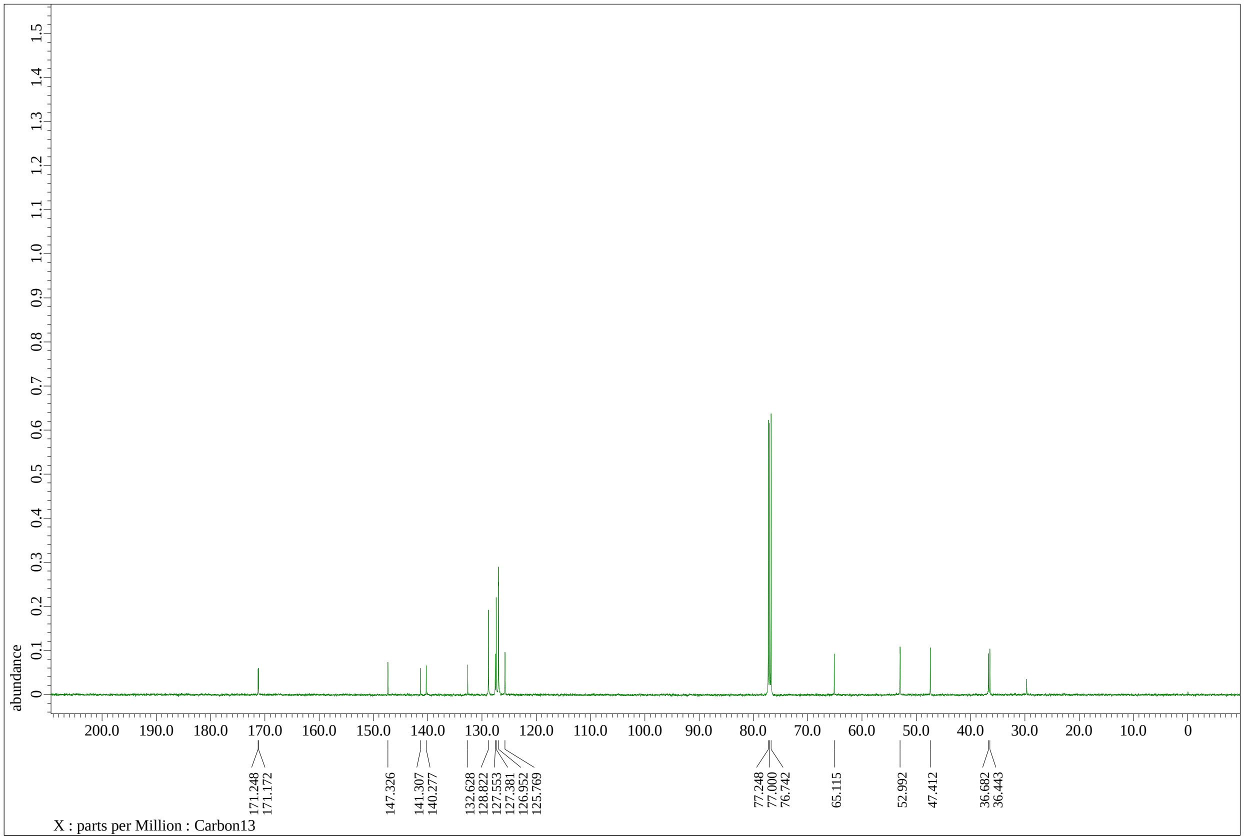

- ^{1}H and ^{13}C NMR Spectra of 3r

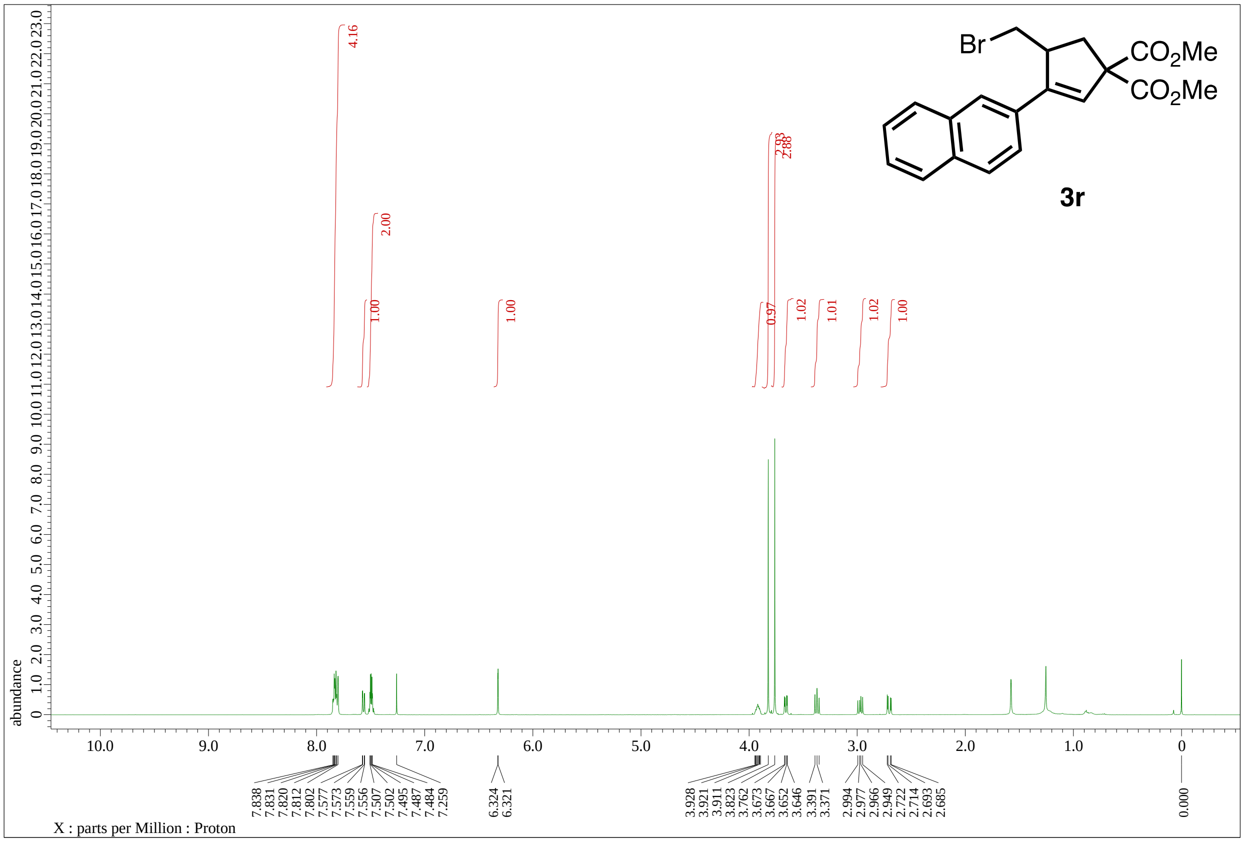

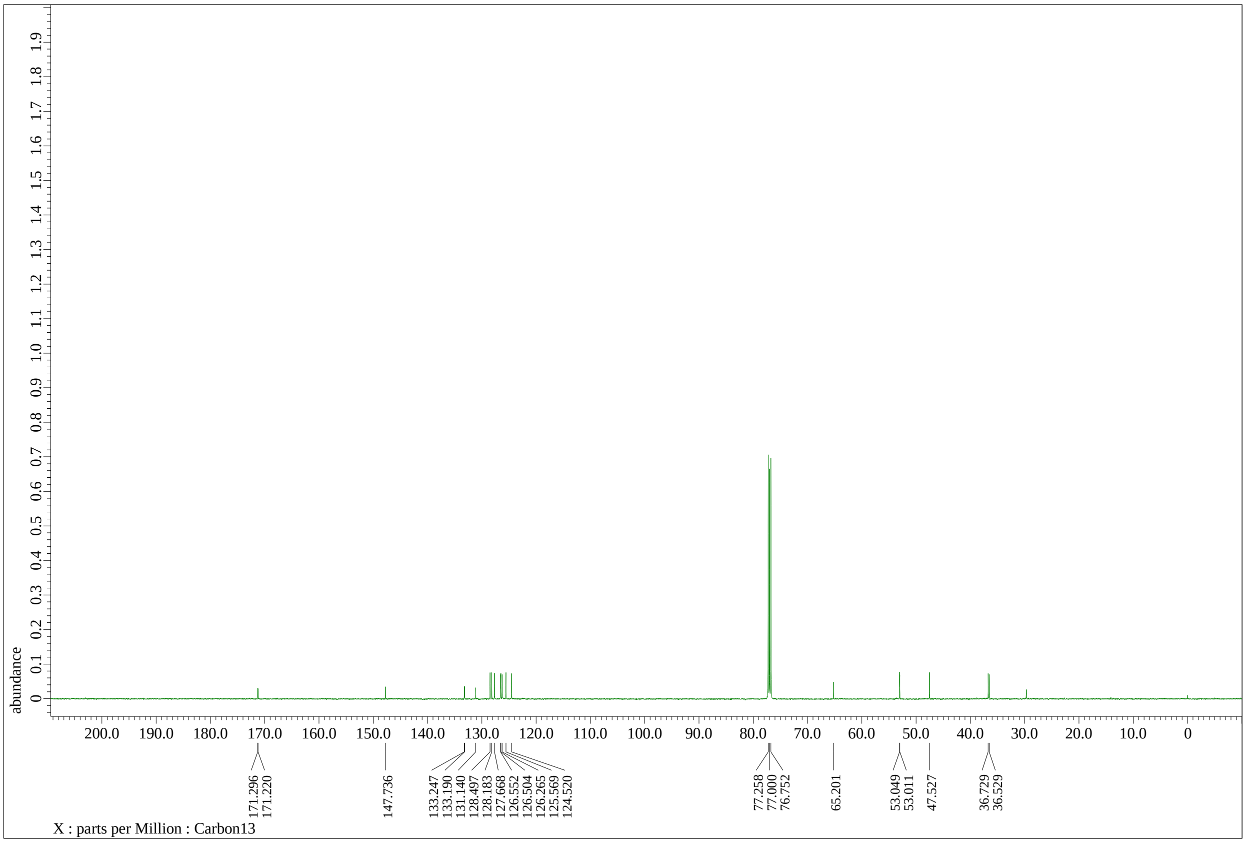

- ^{1}H, ^{13}C and DEPT-135 NMR Spectra of 3s

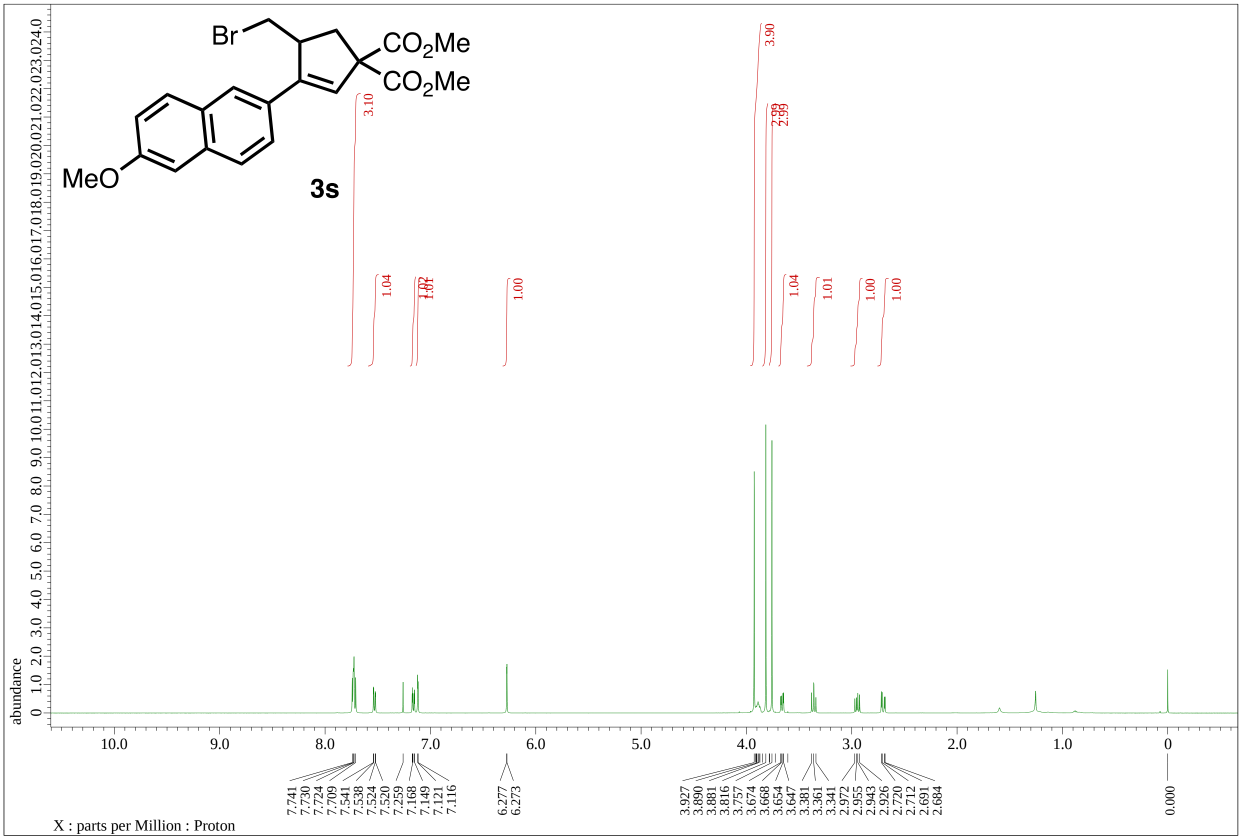

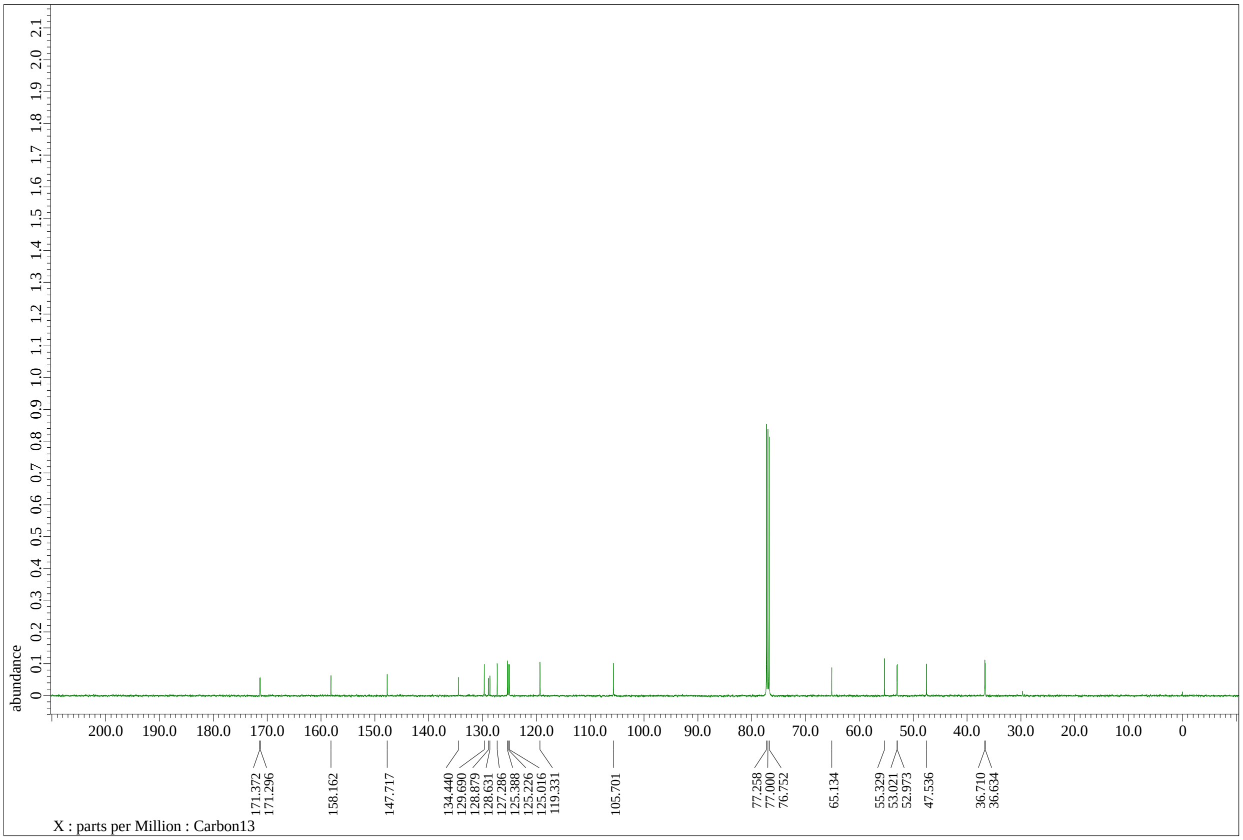

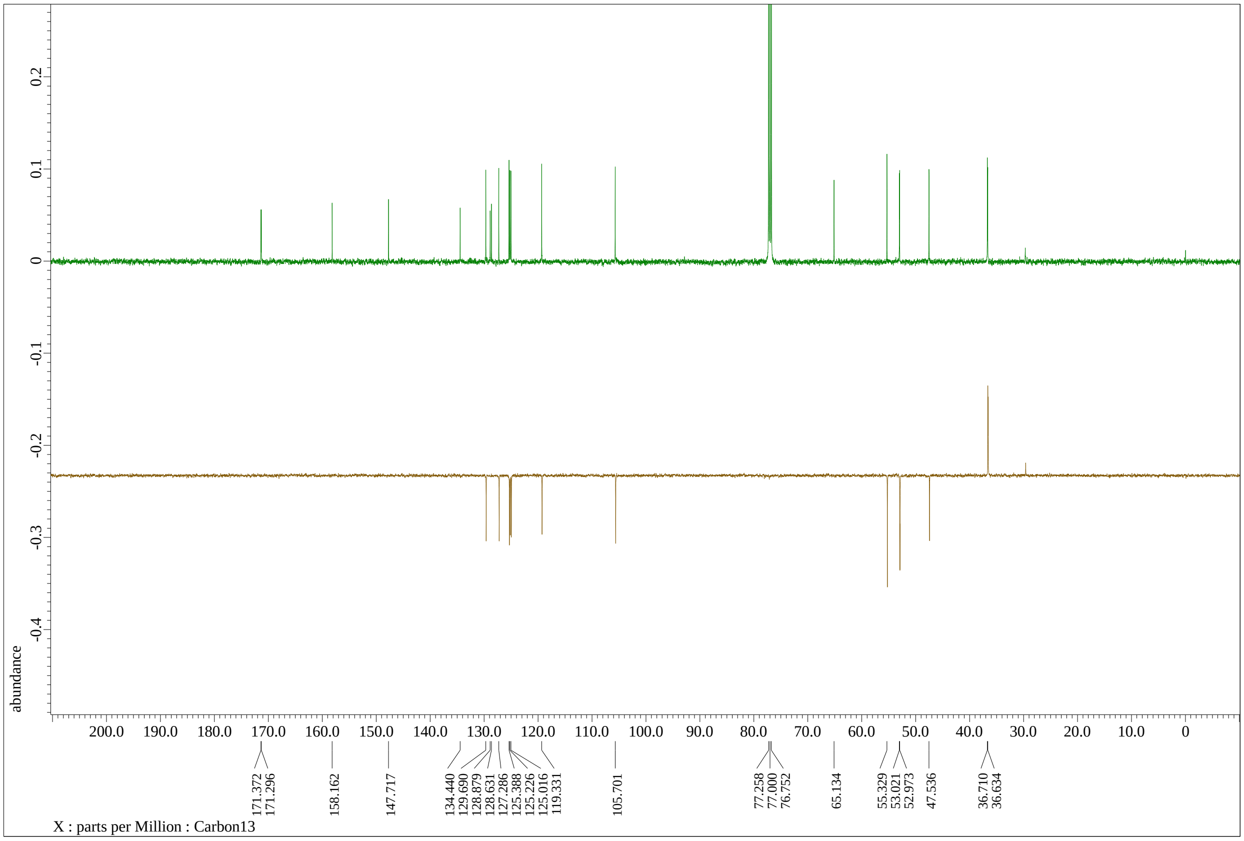

0.2
0.1
0
-0.1
-0.2
-0.3
-0.4
abundance
200.0 190.0 180.0 170.0 160.0 150.0 140.0 130.0 120.0 110.0 100.0 90.0 80.0 70.0 60.0 50.0 40.0 30.0 20.0 10.0 0
171.372
171.296
158.162
147.717
134.440
129.690
128.879
128.631
127.286
125.388
125.226
125.016
119.331
105.701
77.258
77.000
76.752
65.134
55.329
53.021
52.973
47.536
36.710
36.634
X : parts per Million : Carbon13

- ^{1}H and ^{13}C NMR Spectra of 3t

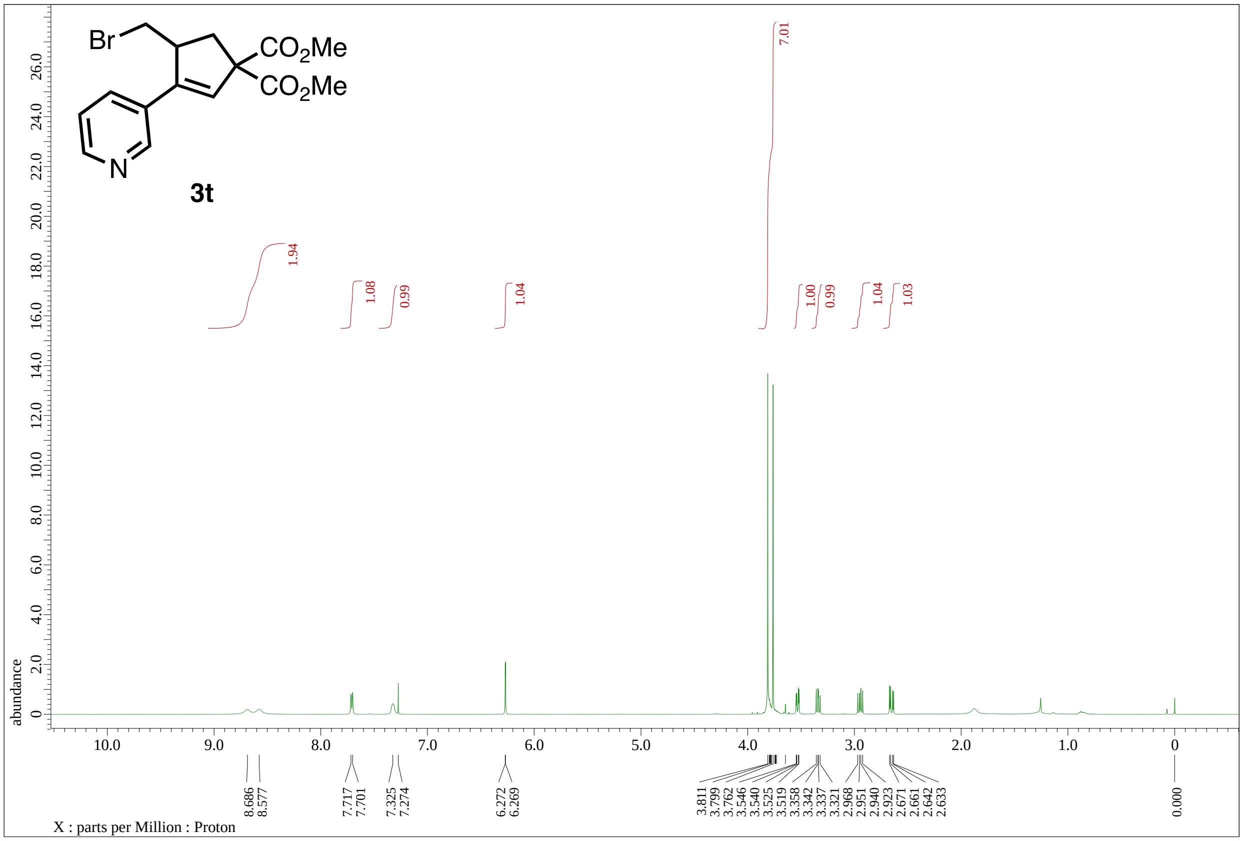

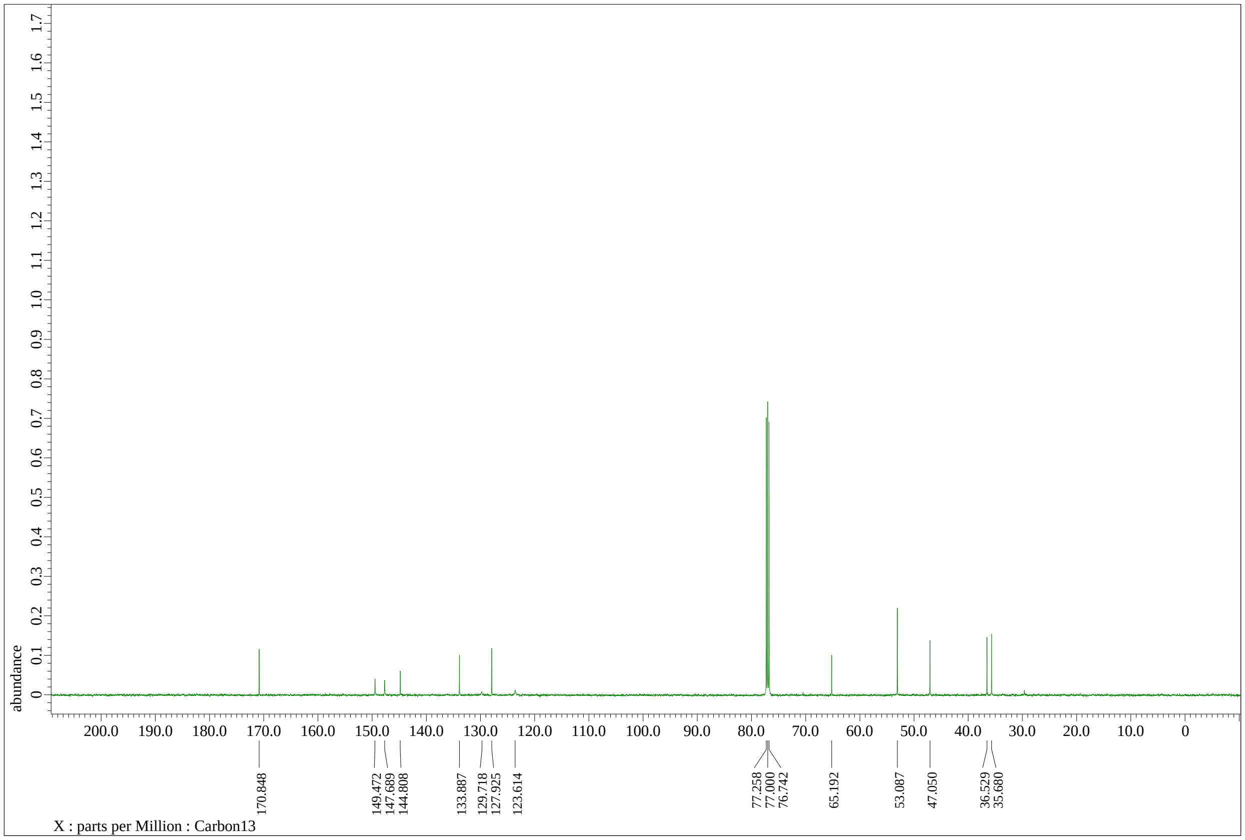

- ^{1}H and ^{13}C NMR Spectra of 3u

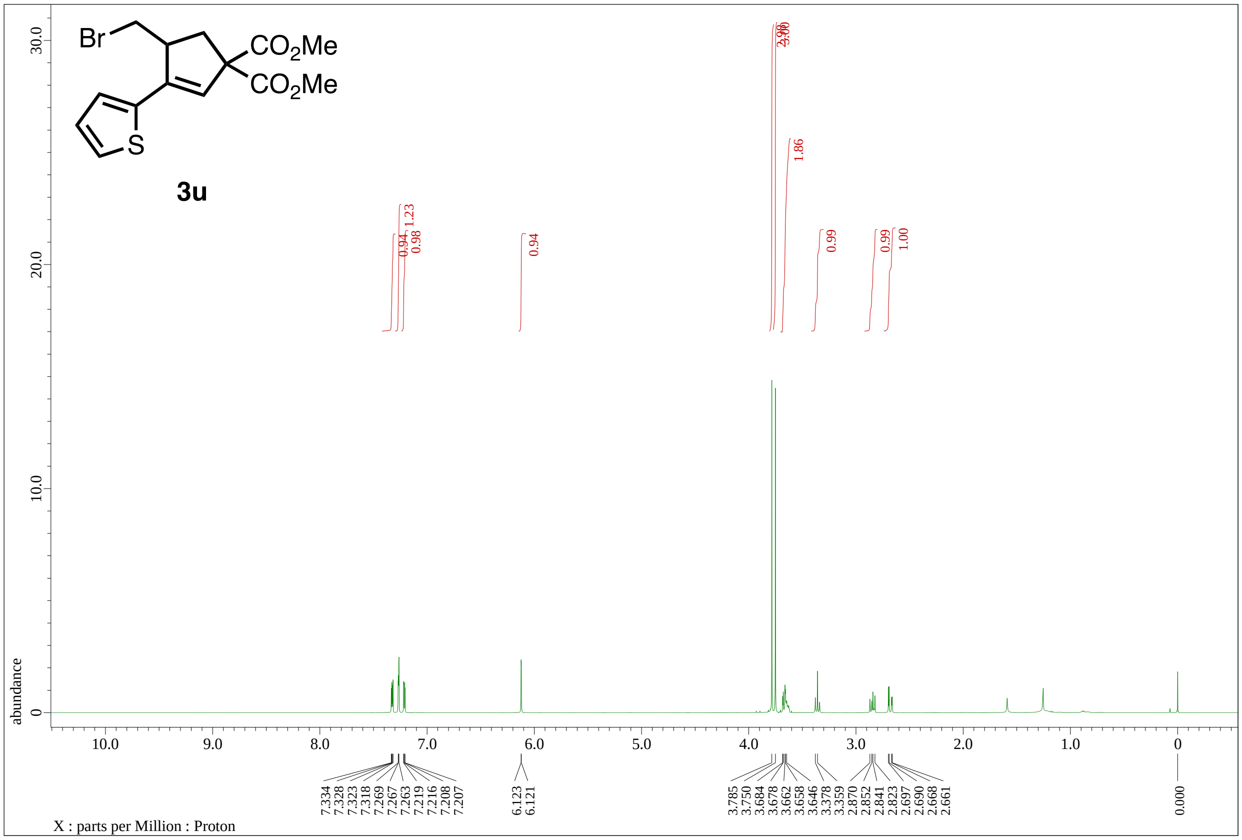

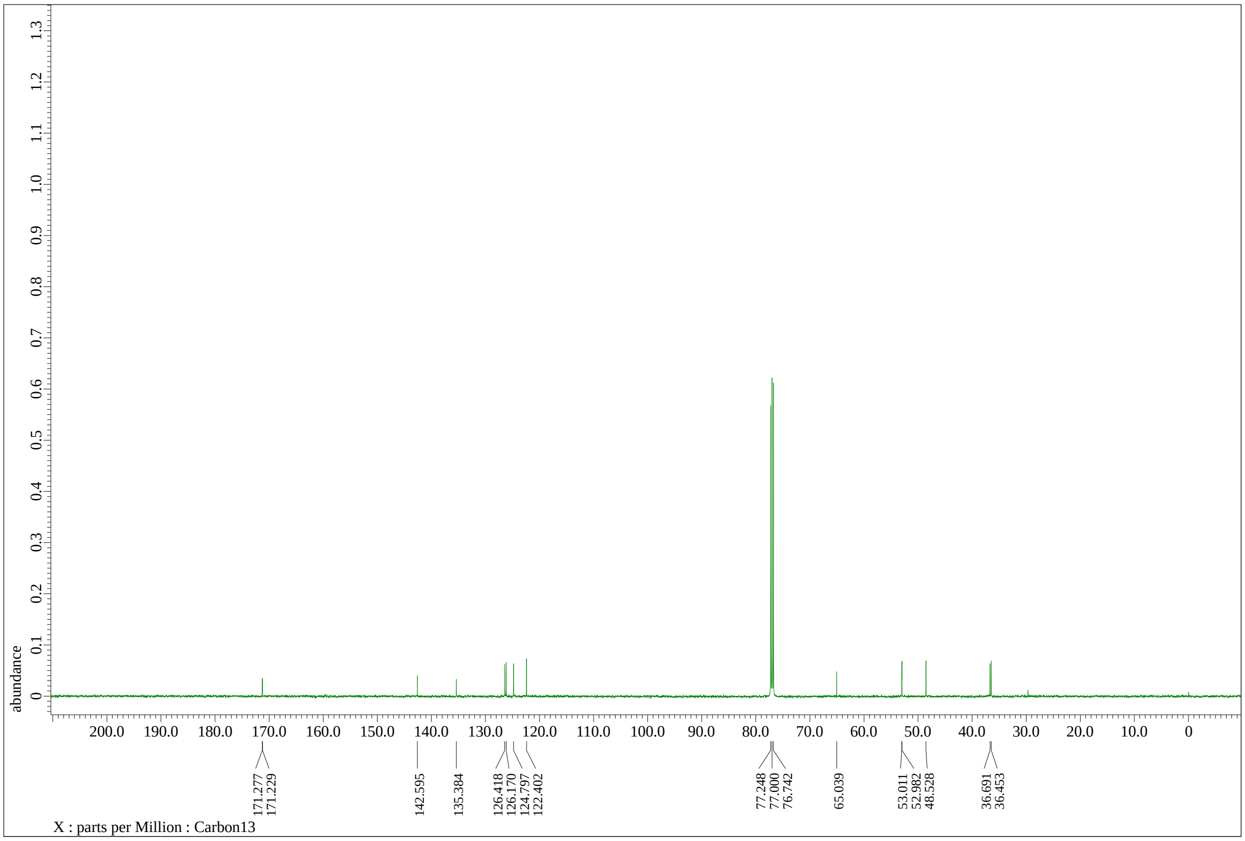

9.^{1}H NMR, ^{13}C NMR, ^{19}F and DEPT-135 NMR Spectra of 5

- ^{1}H and ^{13}C NMR Spectra of 5a

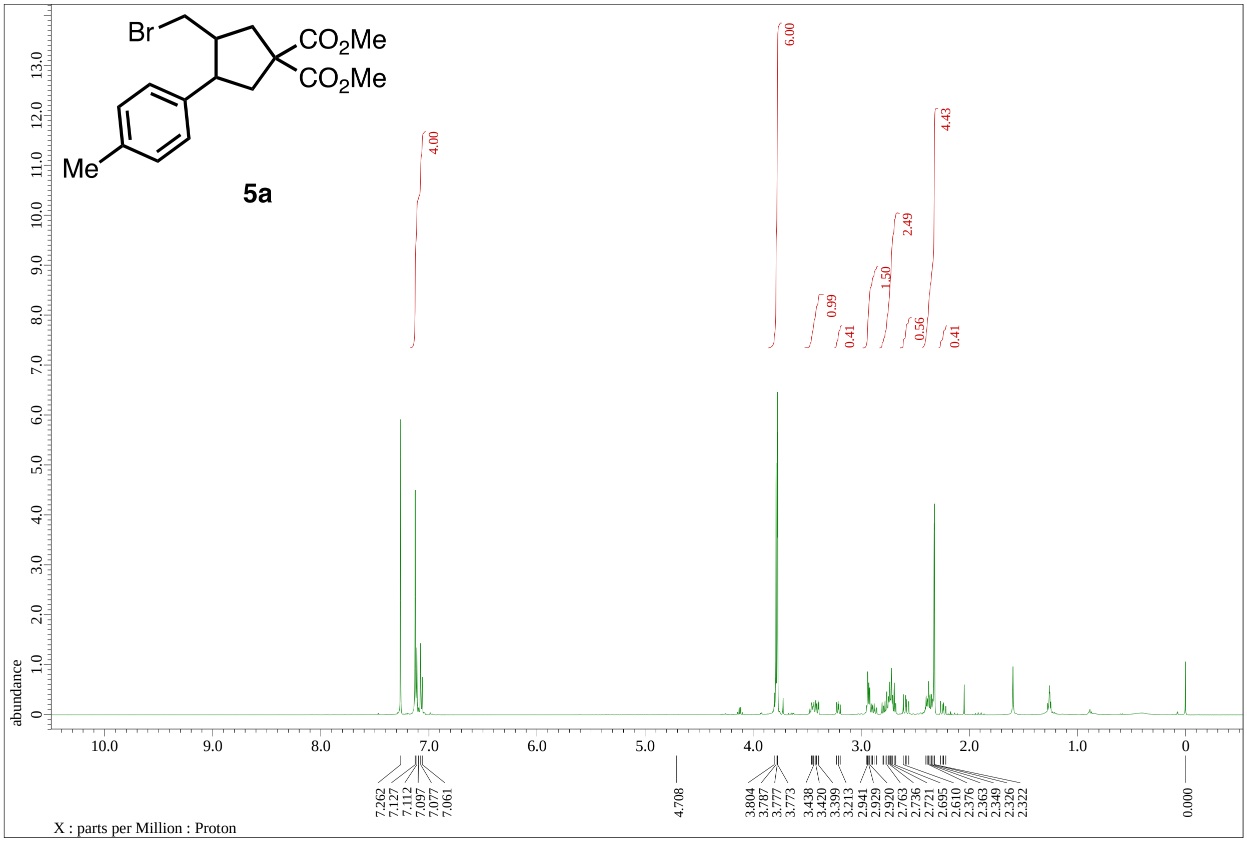

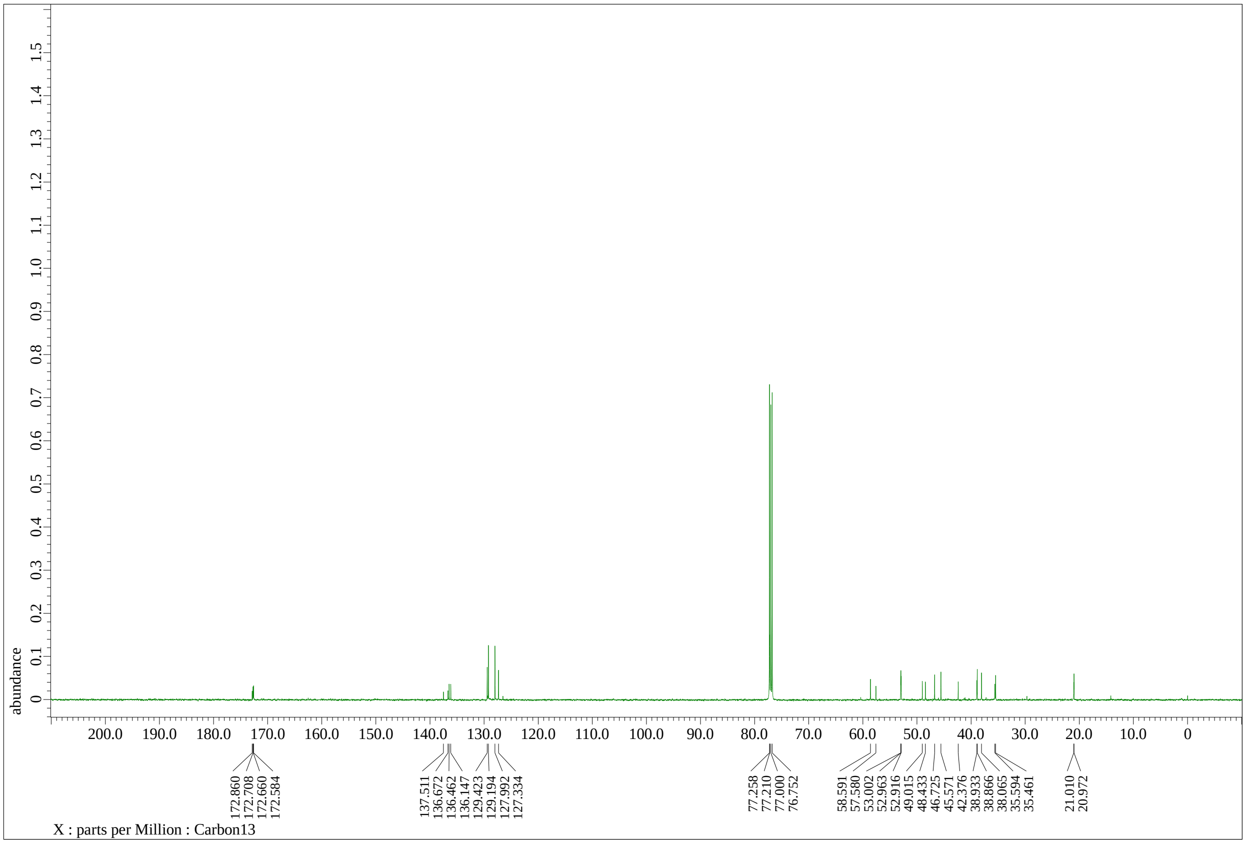

- ^{1}H and ^{13}C NMR Spectra of 5b

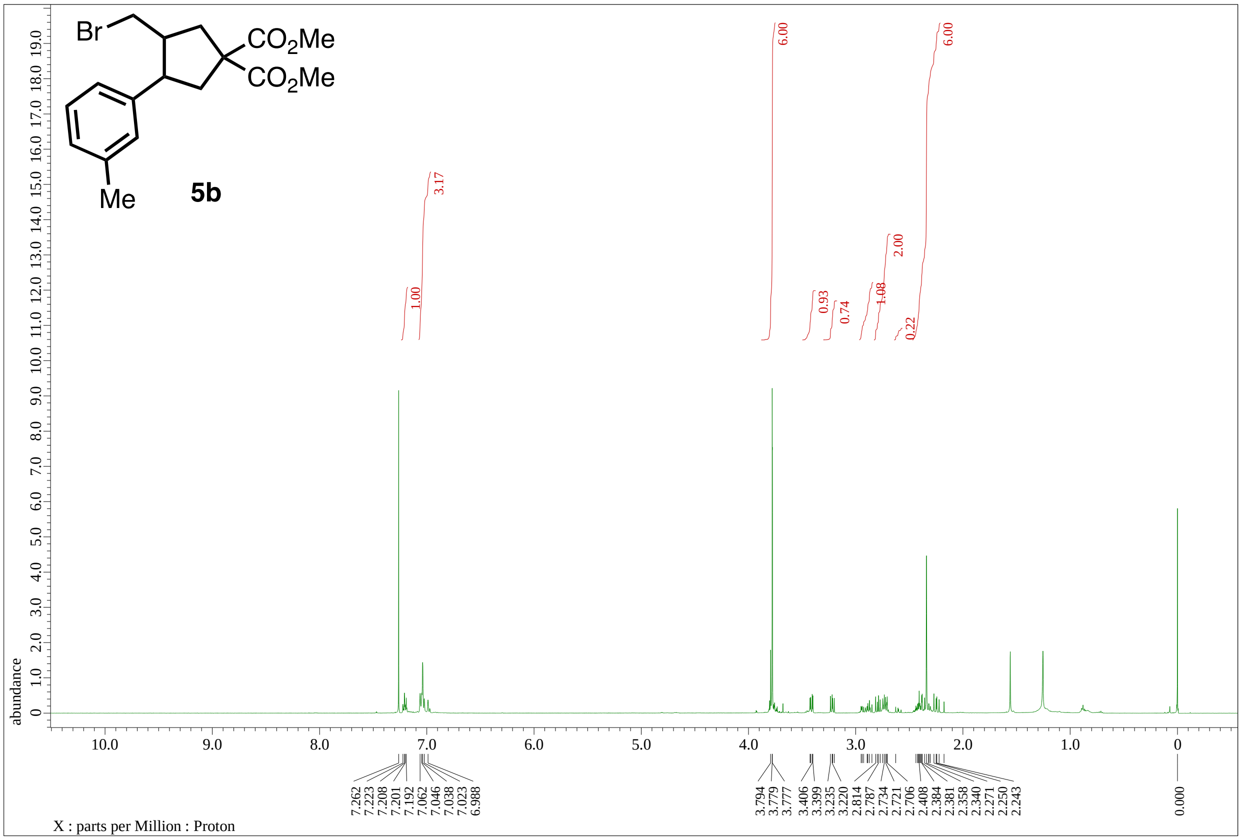

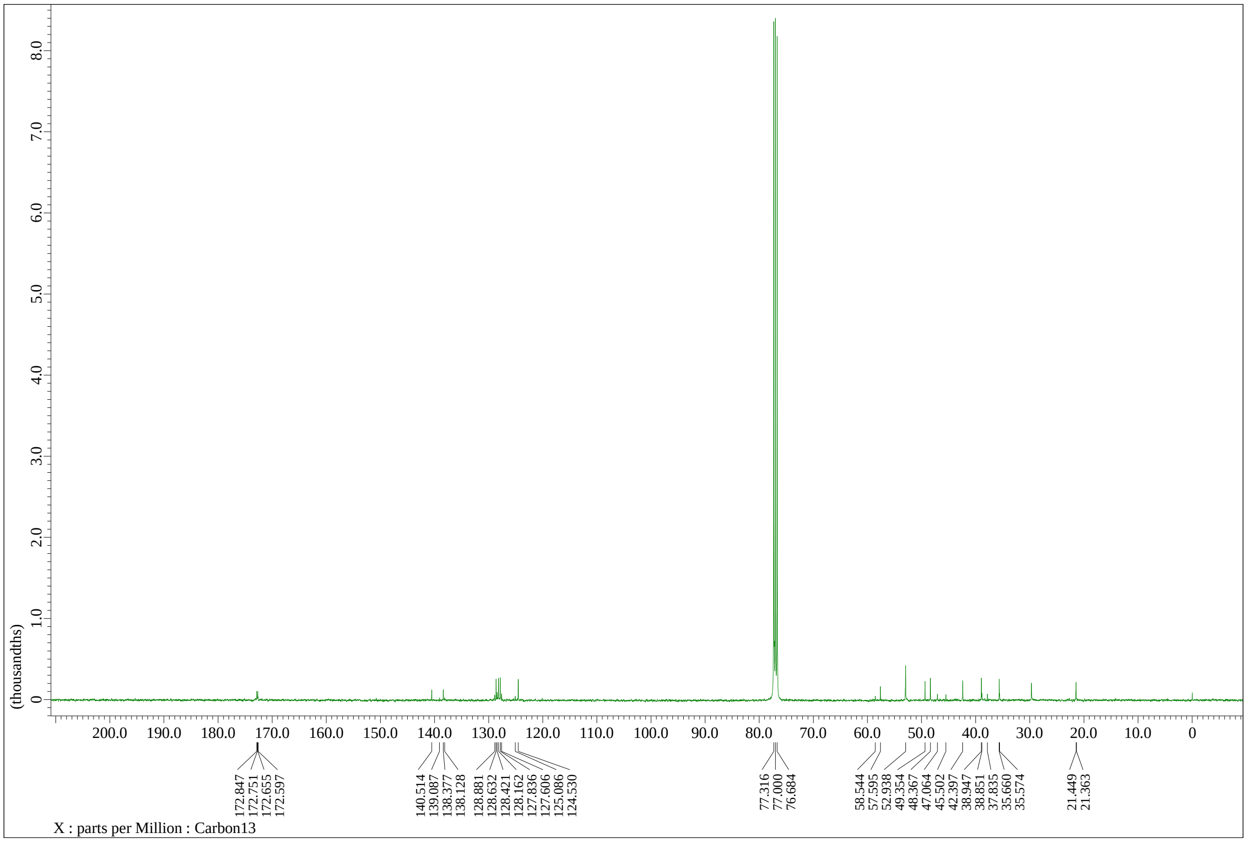

- ^{1}H and ^{13}C NMR Spectra of 5c

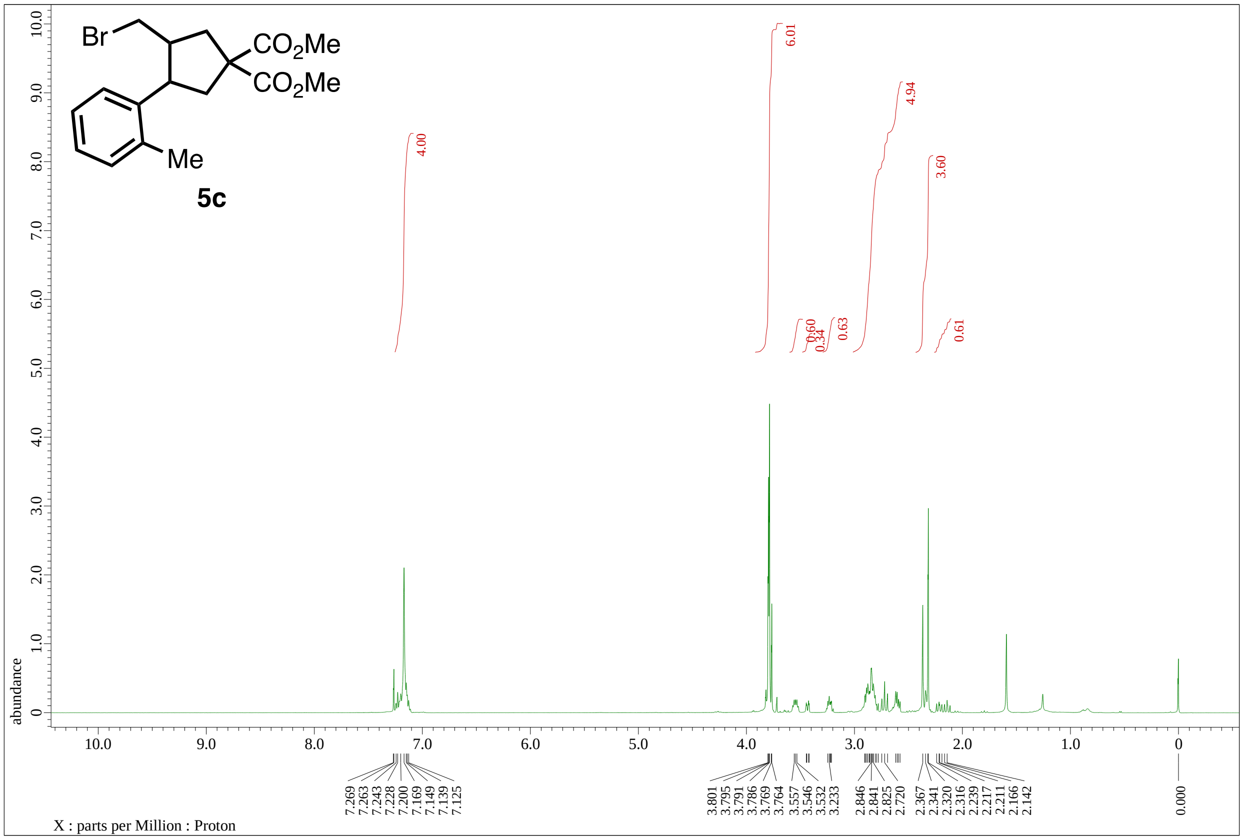

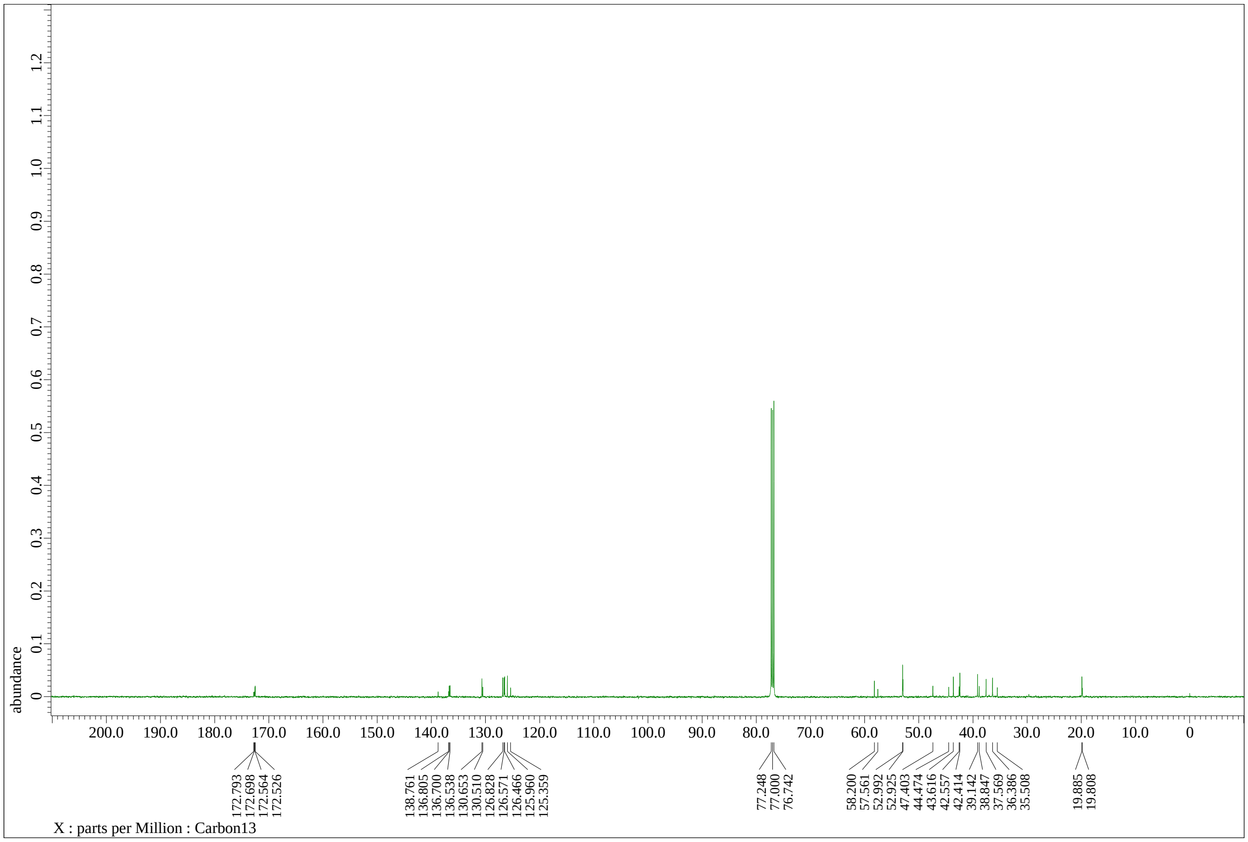

- ^{1}H and ^{13}C NMR Spectra of 5d

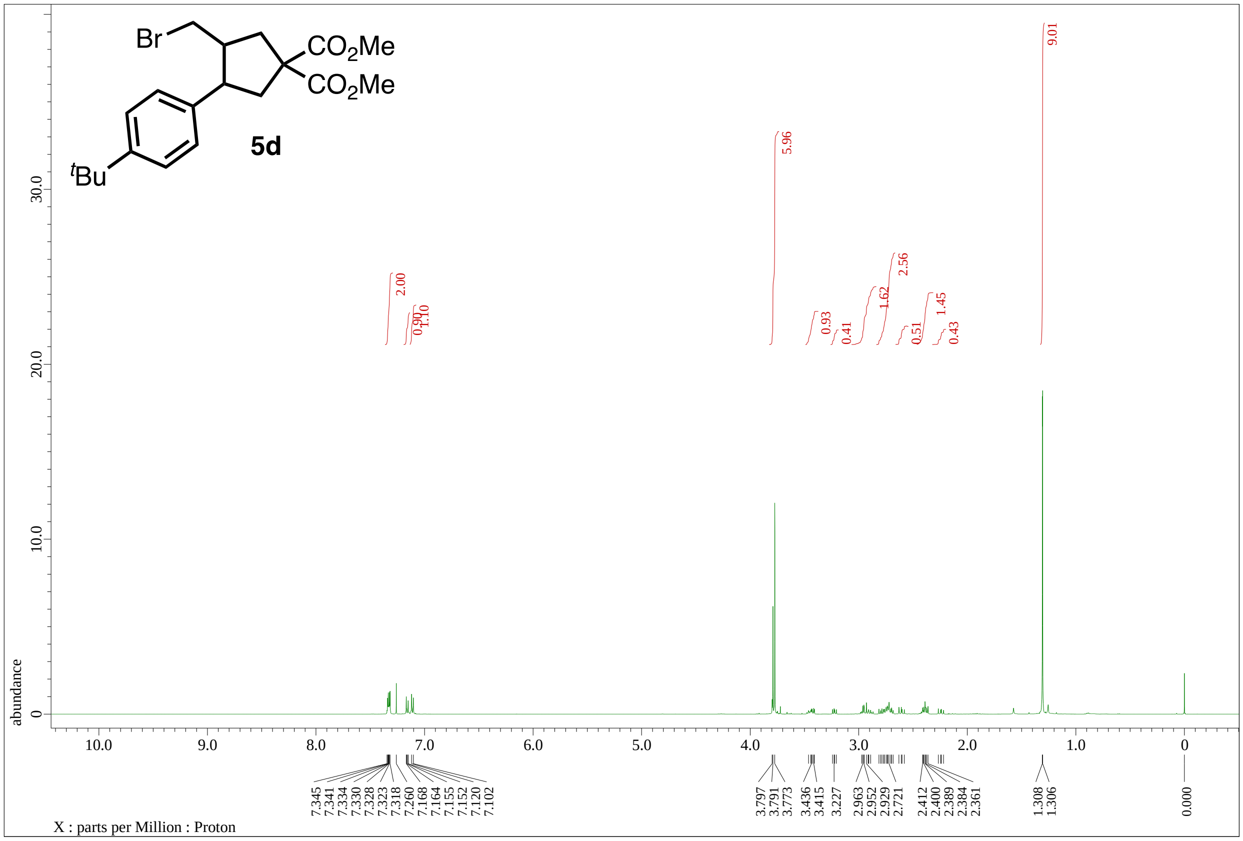

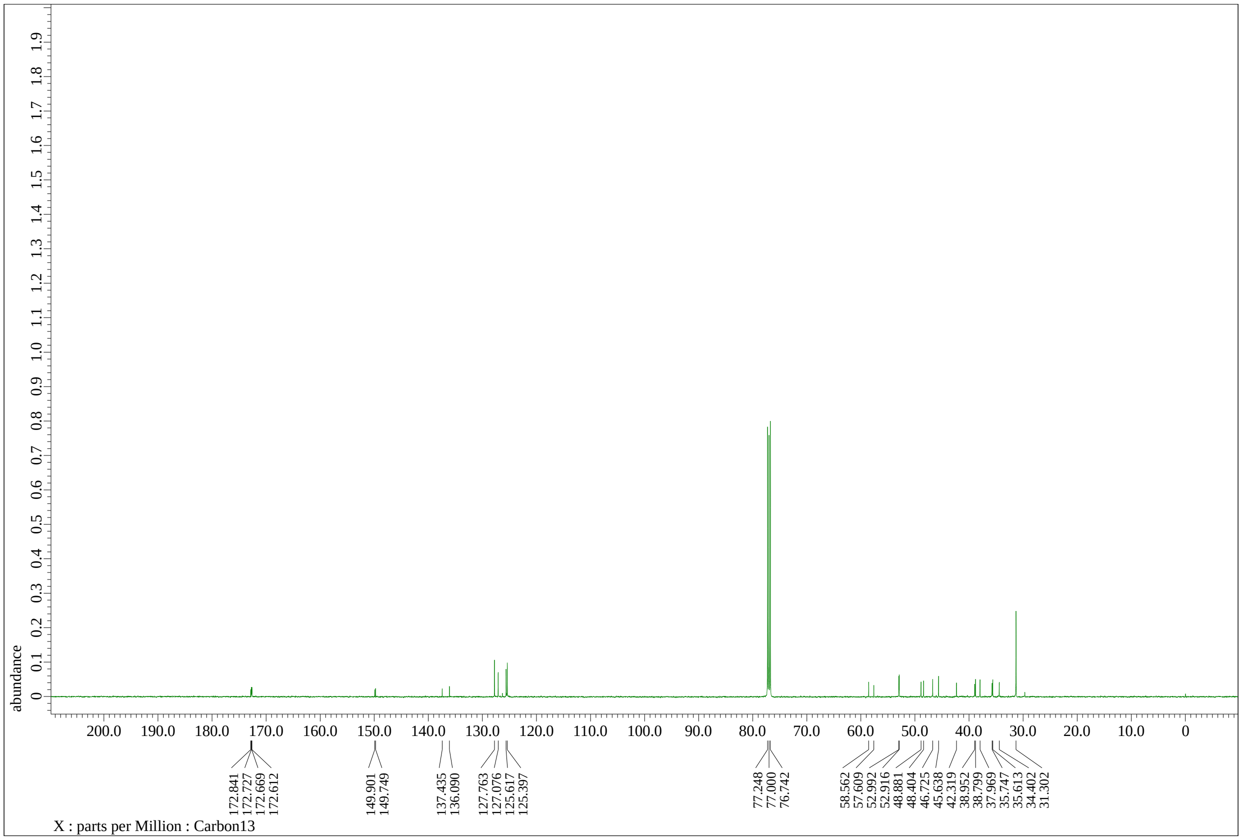

- ^{1}H and ^{13}C NMR Spectra of 5e

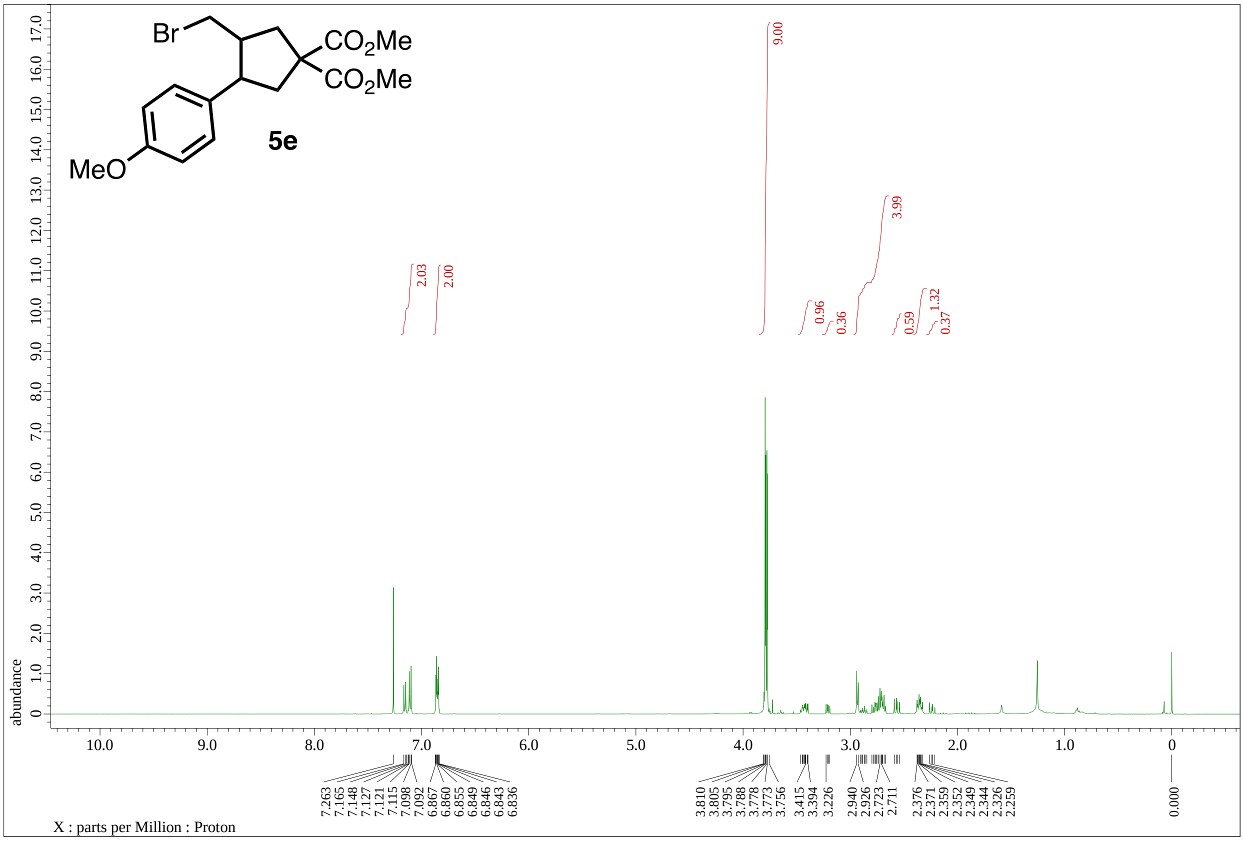

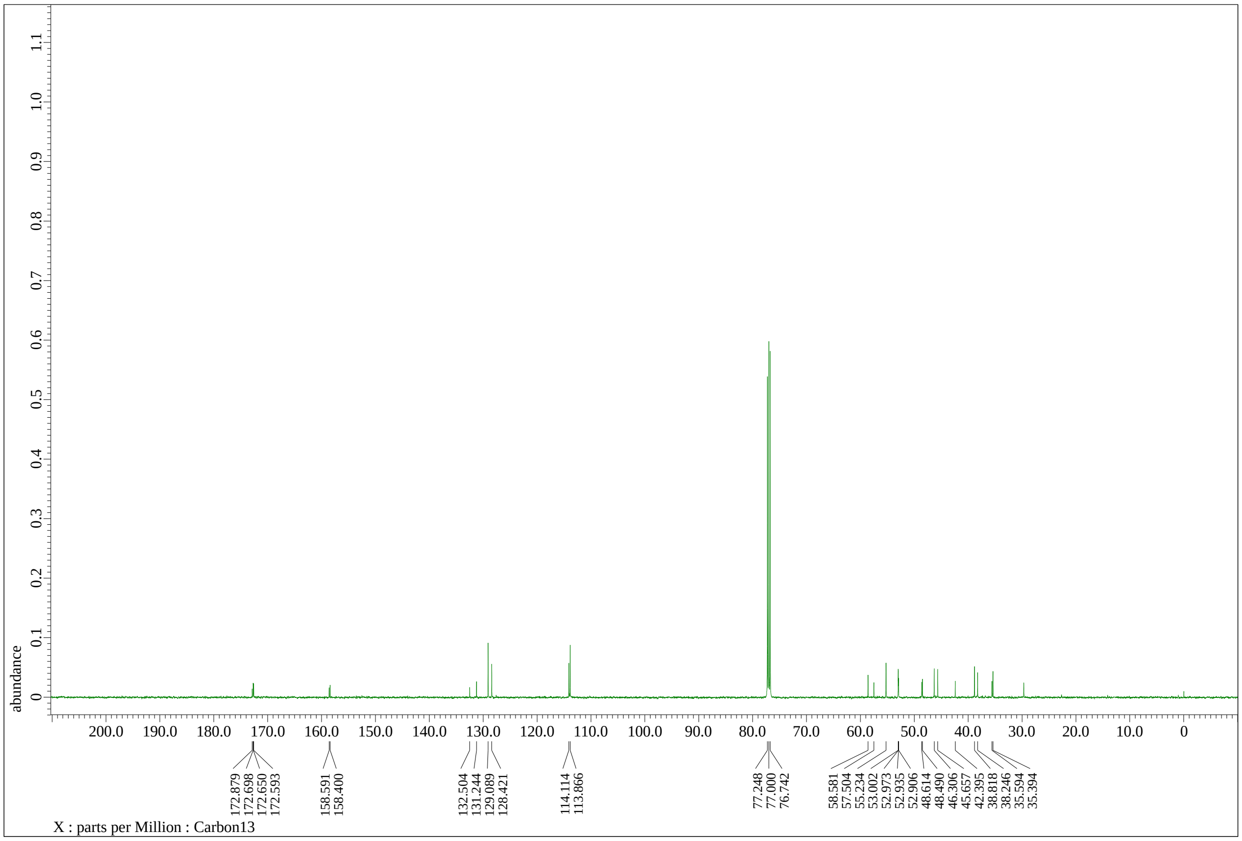

- ^{1}H, ^{13}C and ^{19}F NMR Spectra of 5f

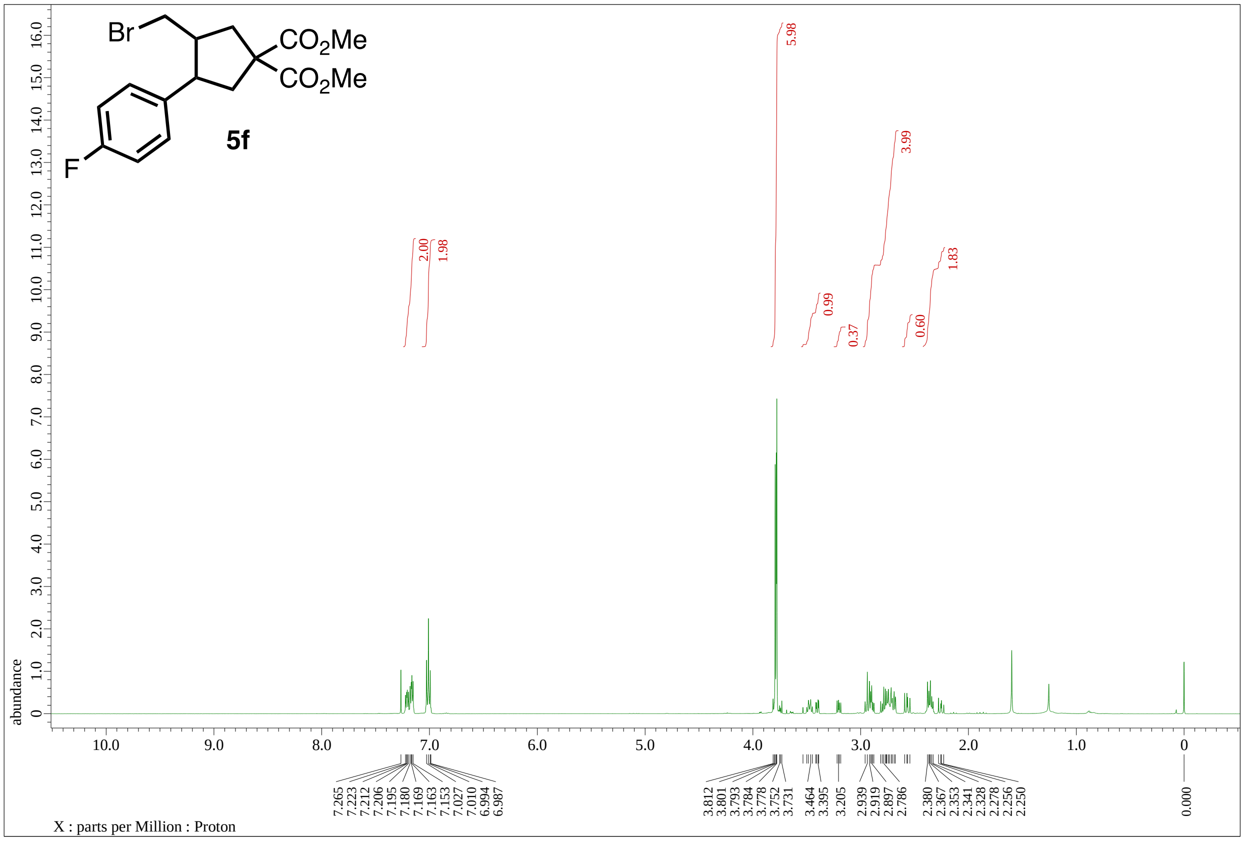

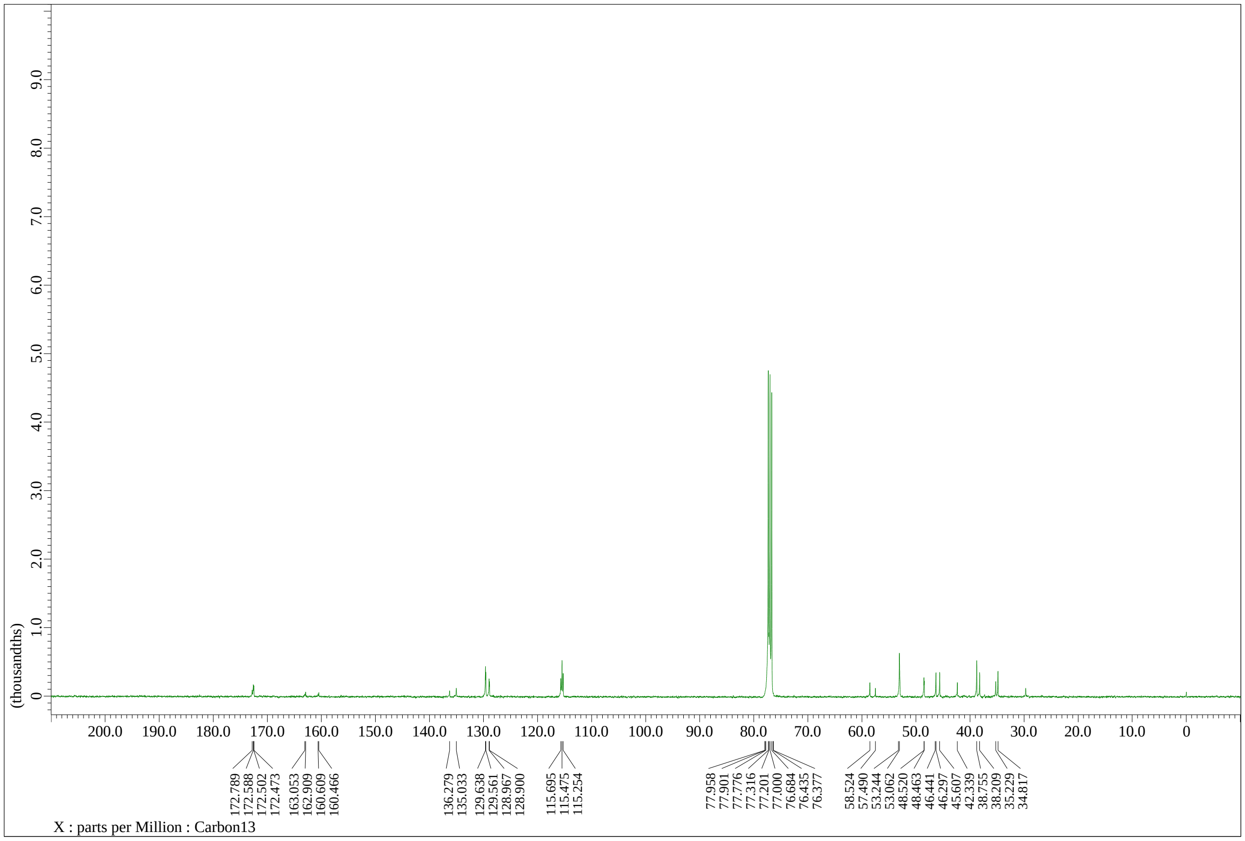

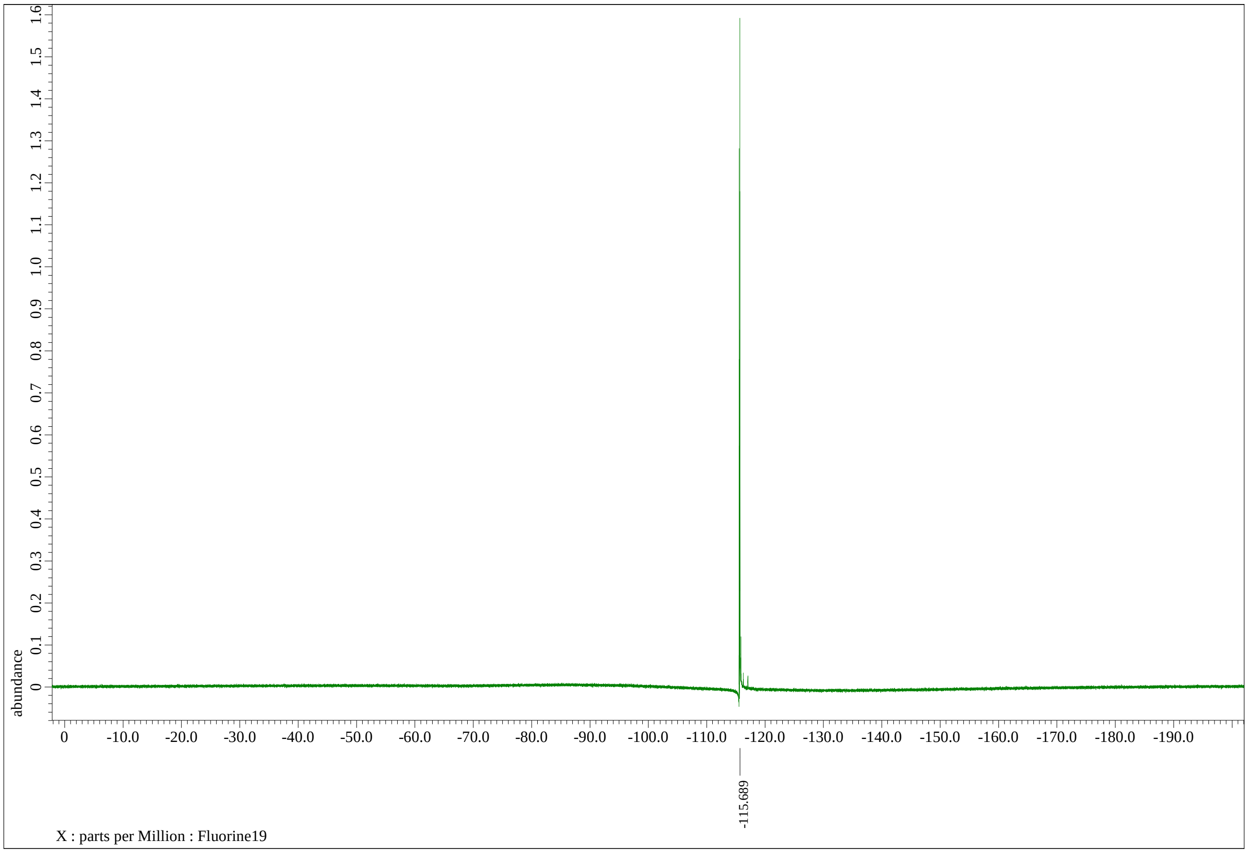
abundance
1.6
1.5
1.4
1.3
1.2
1.1
1.0
0.9
0.8
0.7
0.6
0.5
0.4
0.3
0.2
0.1
0
0
-10.0
-20.0
-30.0
-40.0
-50.0
-60.0
-70.0
-80.0
-90.0
-100.0
-110.0
-120.0
-130.0
-140.0
-150.0
-160.0
-170.0
-180.0
-190.0
-115.689
X : parts per Million : Fluorine19

- ^{1}H and ^{13}C NMR Spectra of 5g

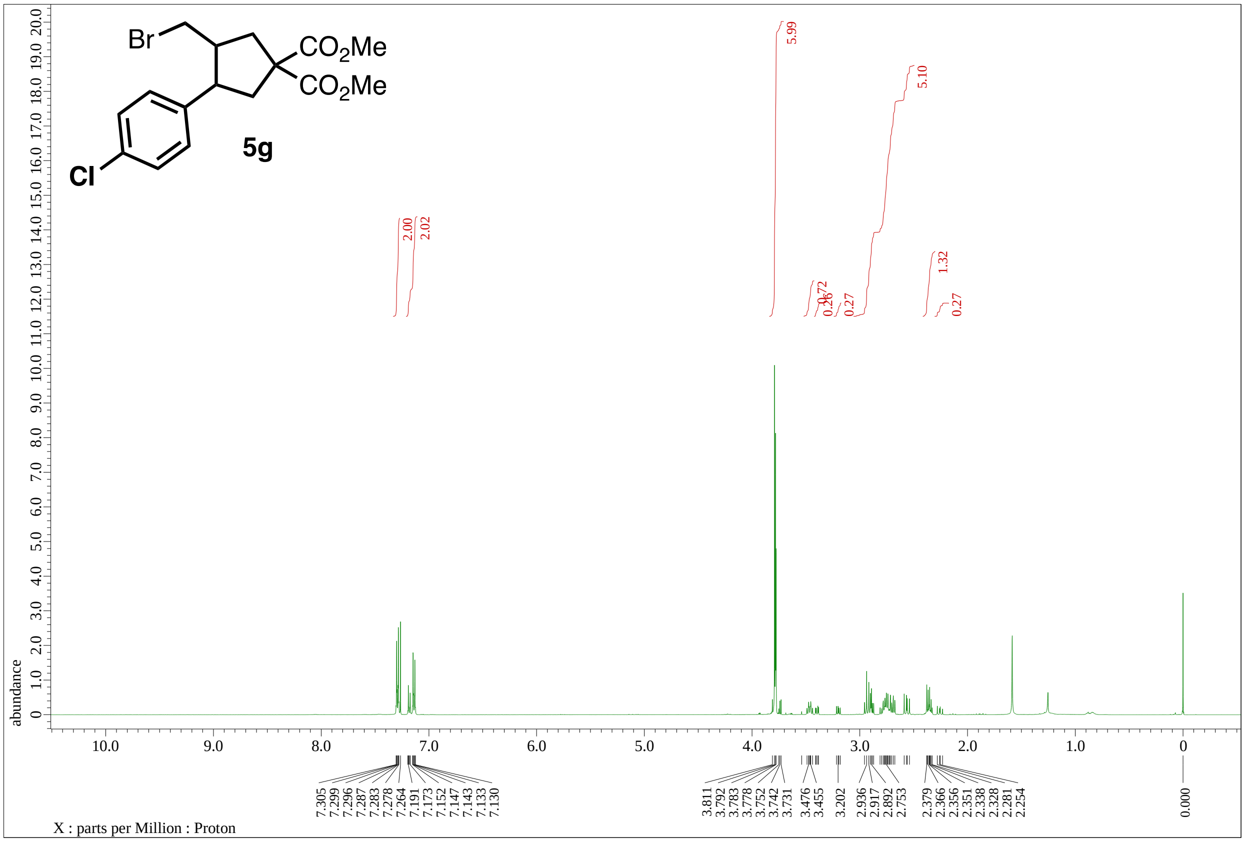

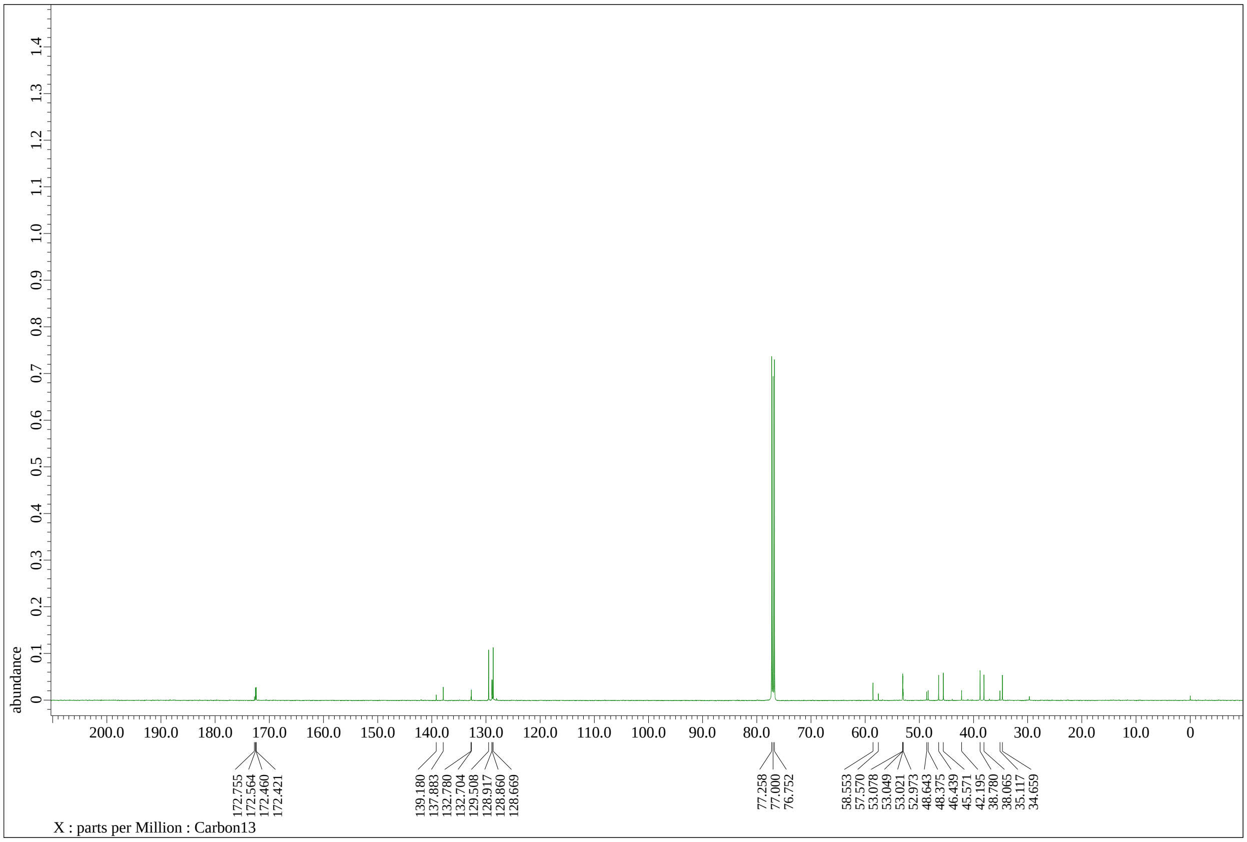

- ^{1}H and ^{13}C NMR Spectra of 5h

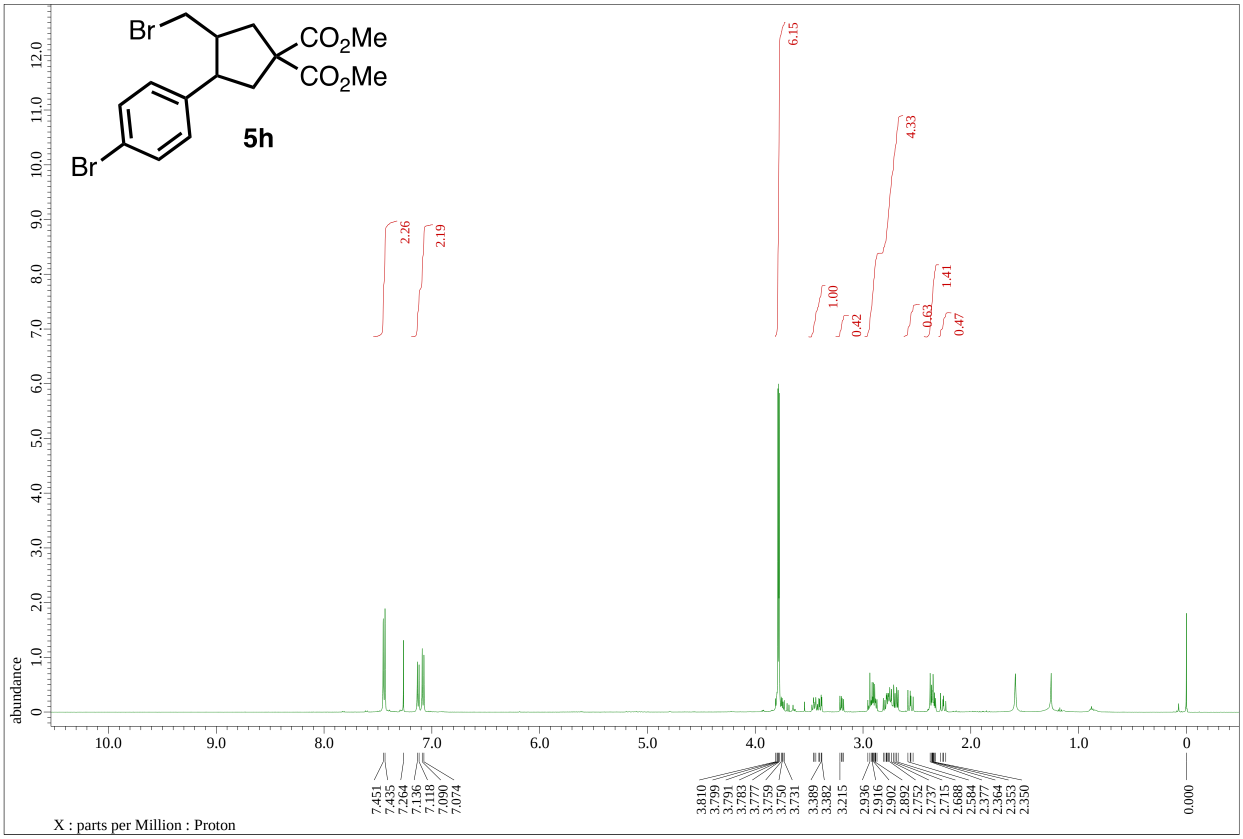

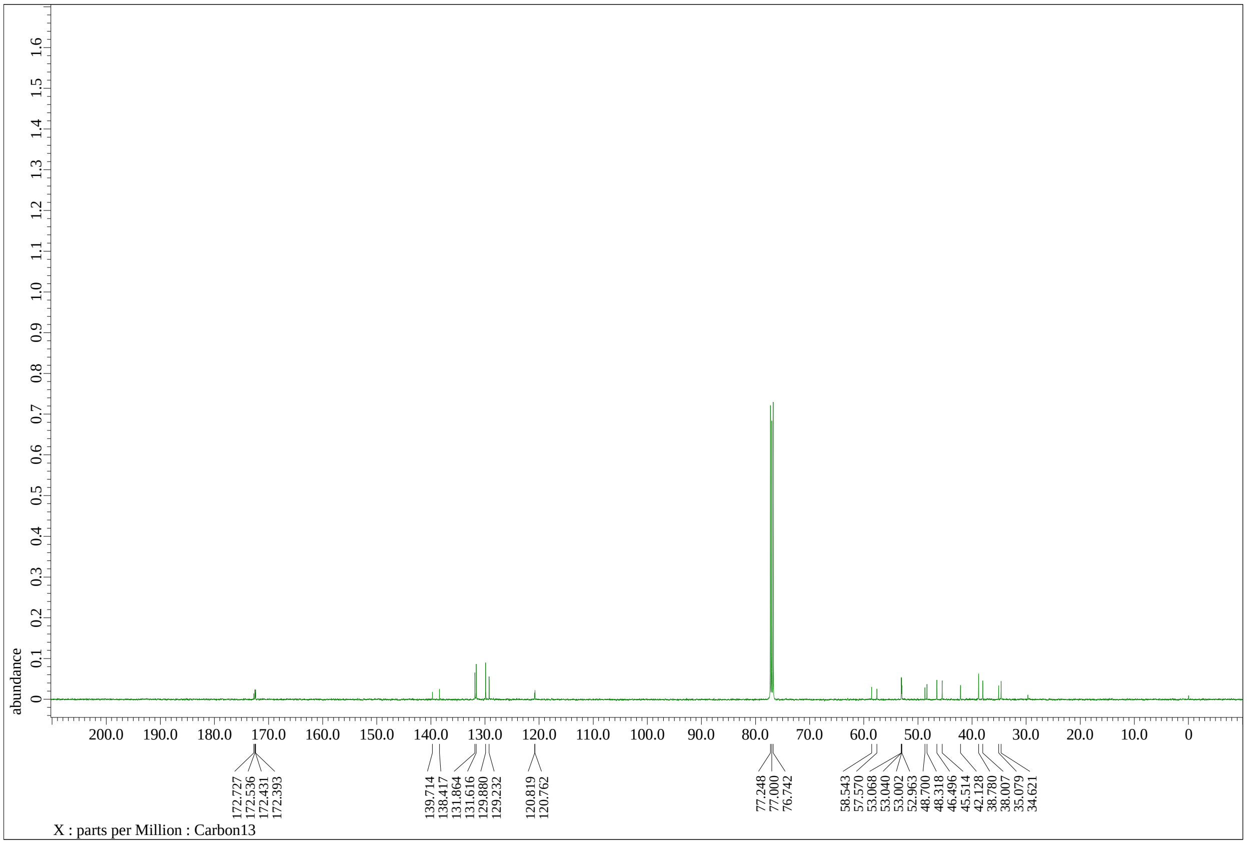

- ^{1}H and ^{13}C NMR Spectra of 5i

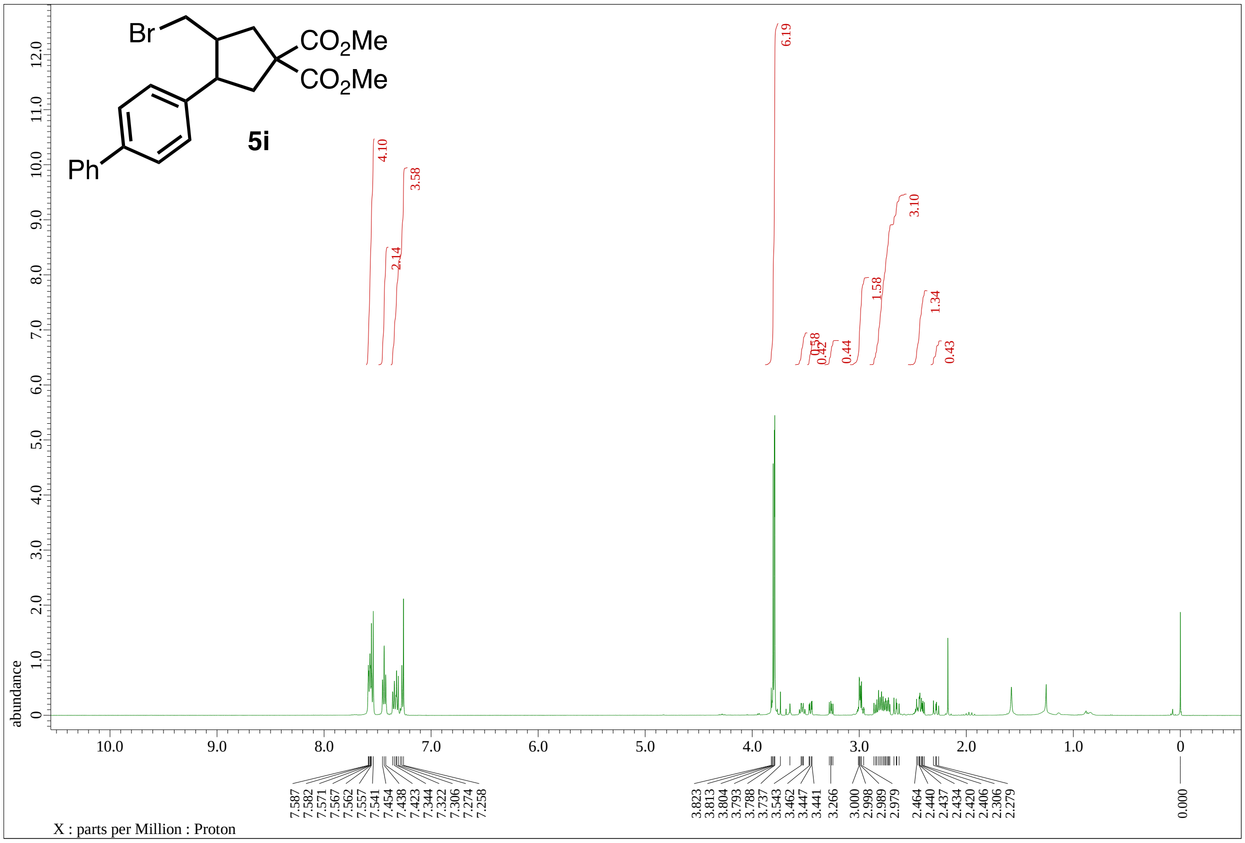

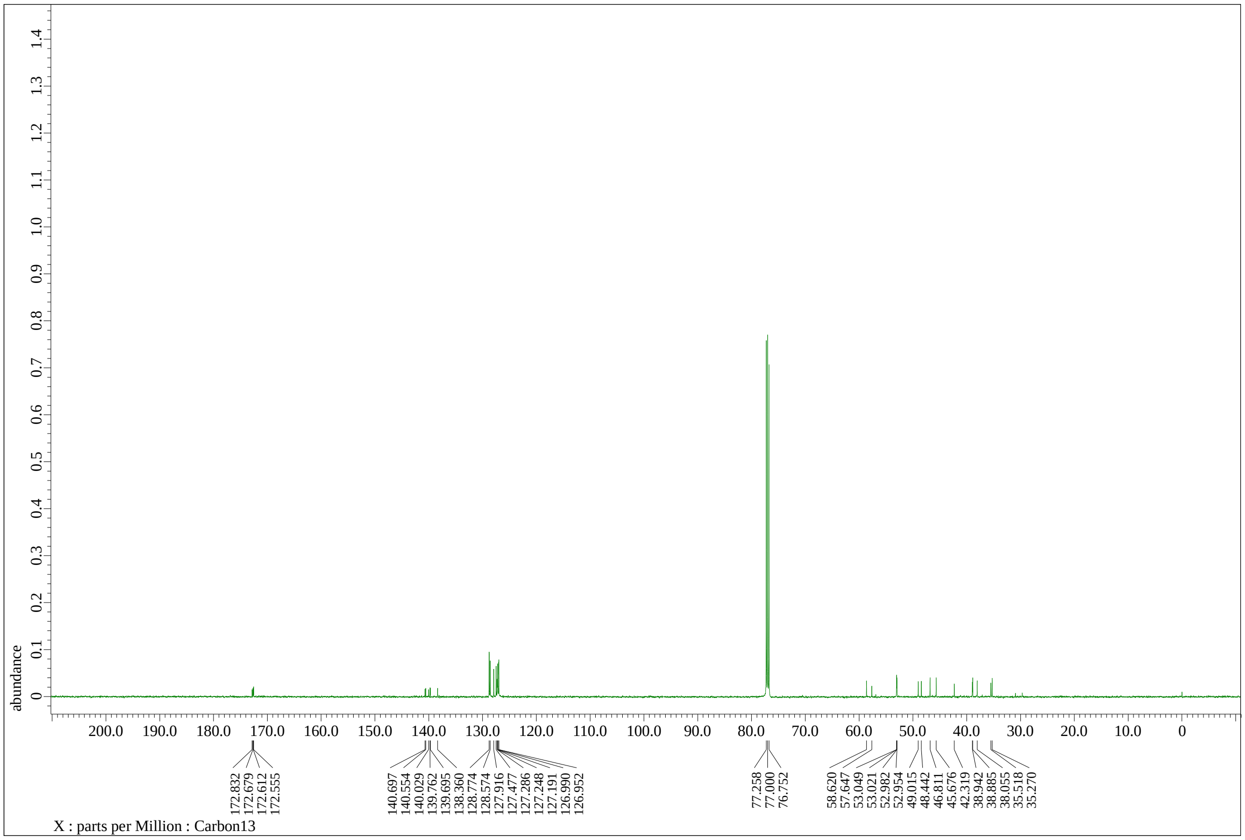

- ^{1}H and ^{13}C NMR Spectra of 5j

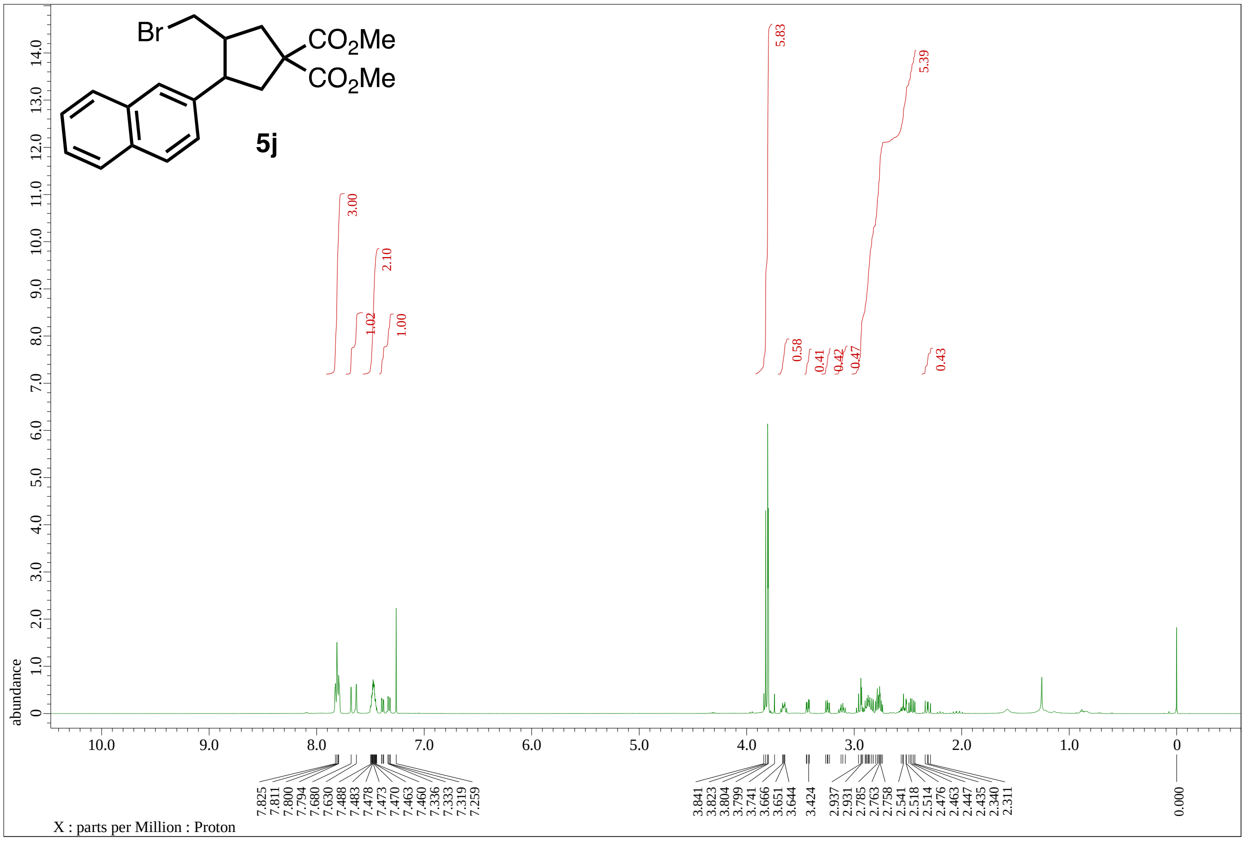

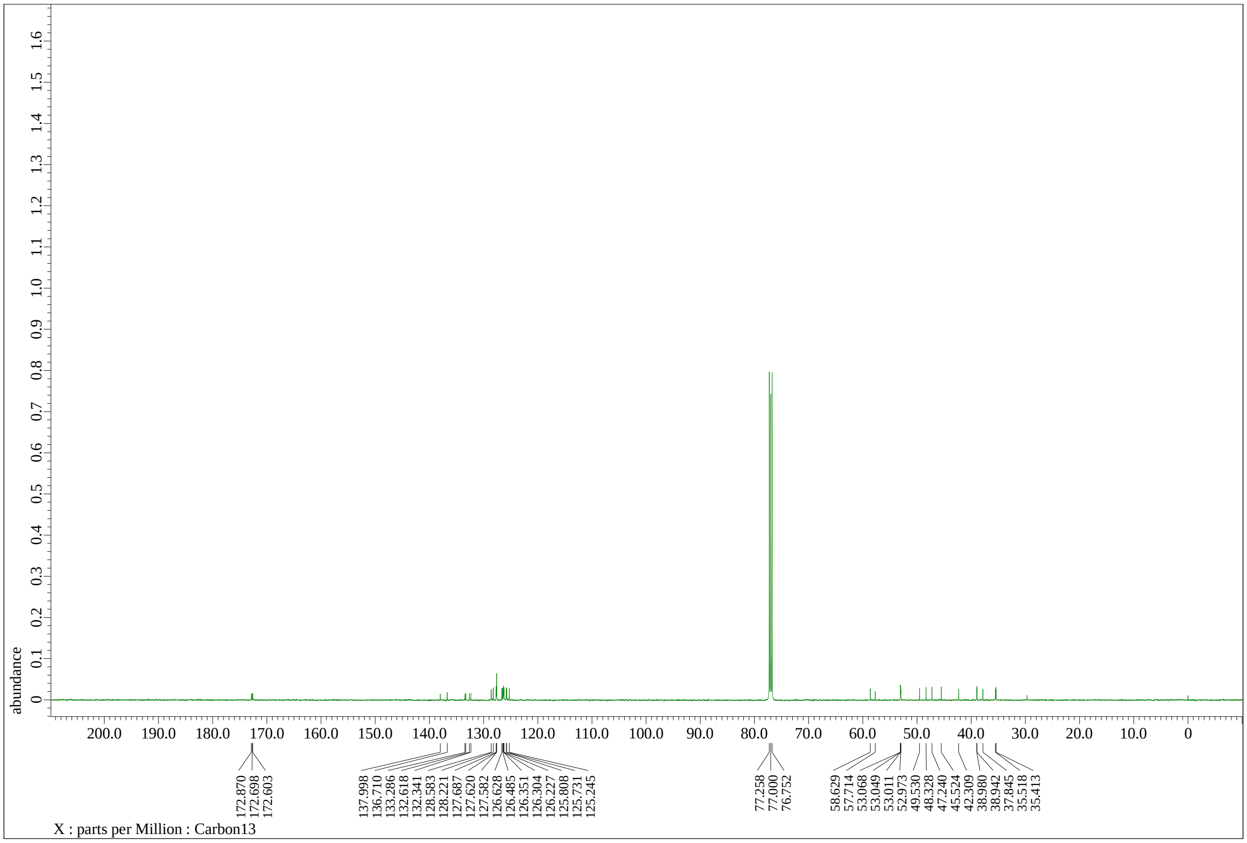

- ^{1}H and ^{13}C NMR Spectra of 5k

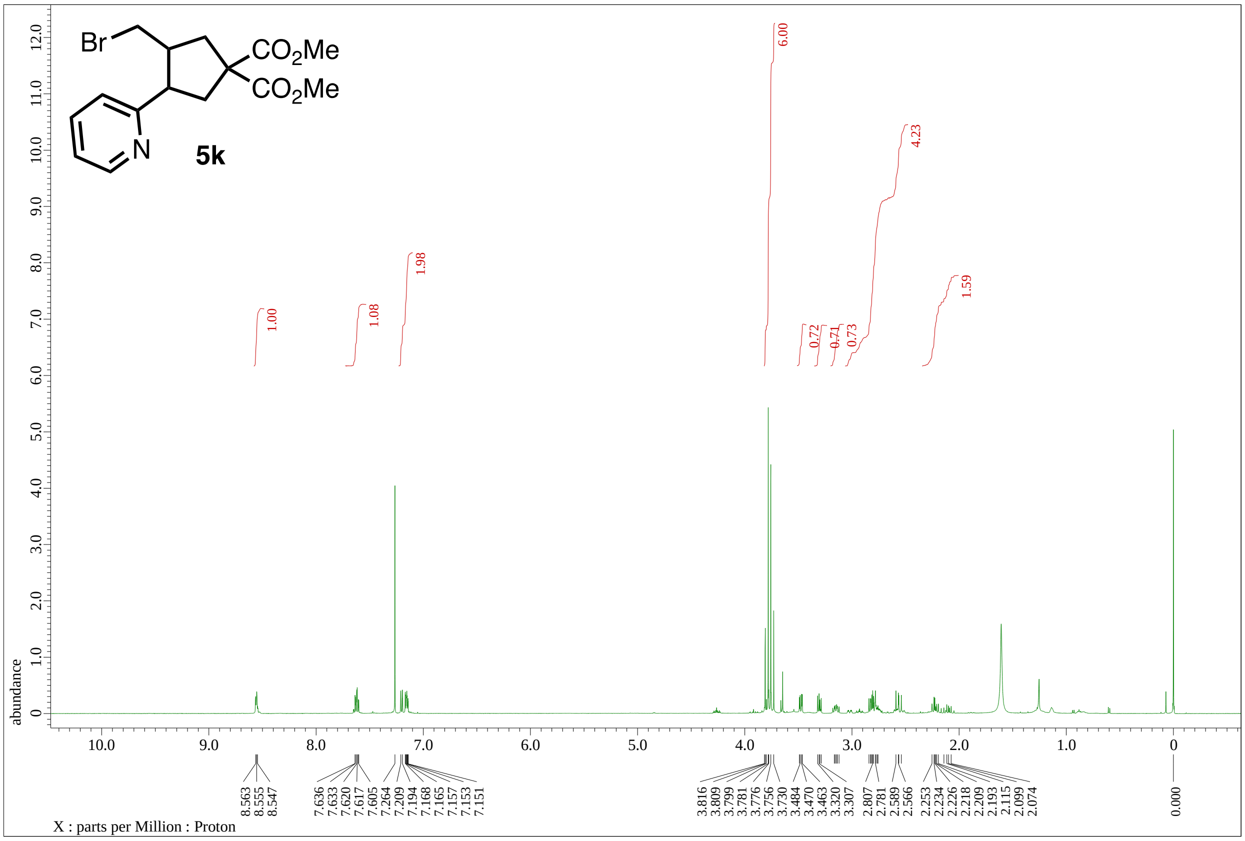

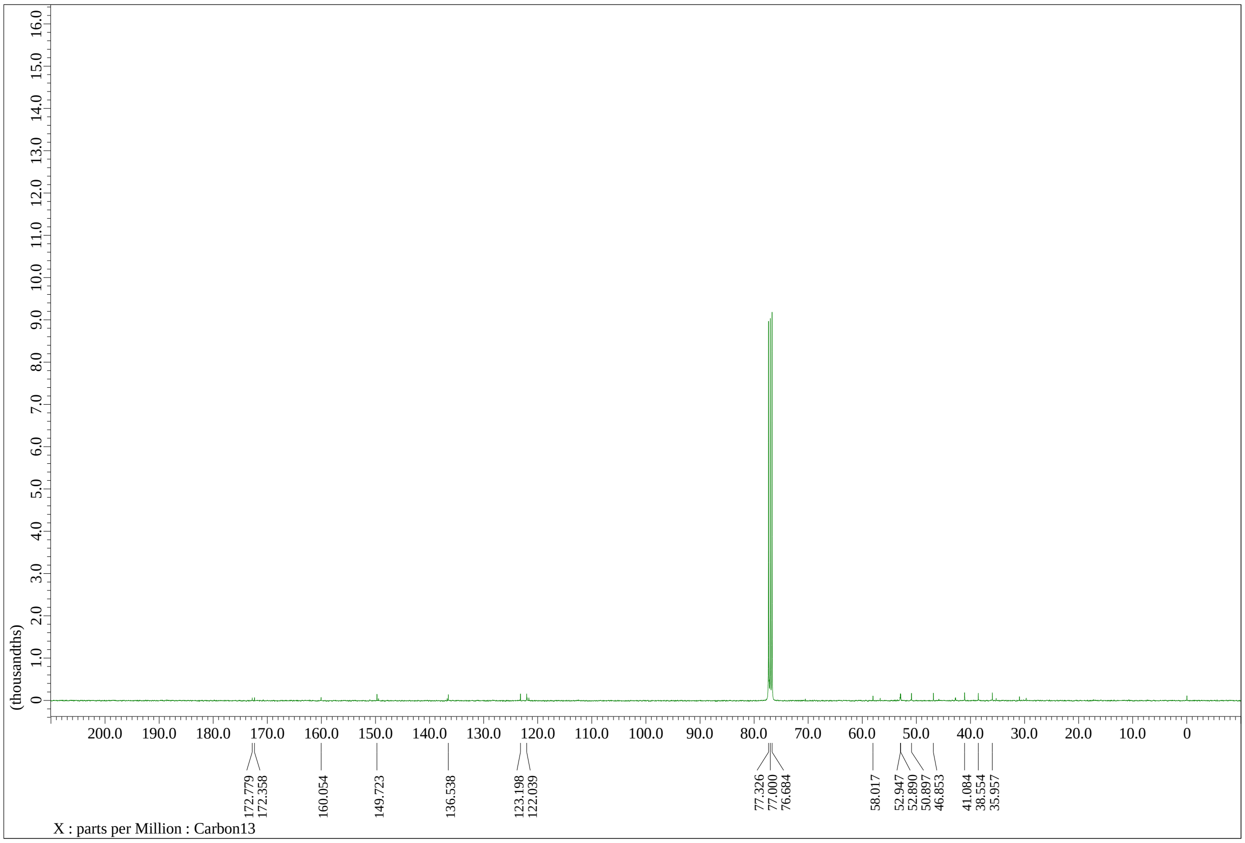

- ^{1}H, ^{13}C and DEPT-135 NMR Spectra of 5l

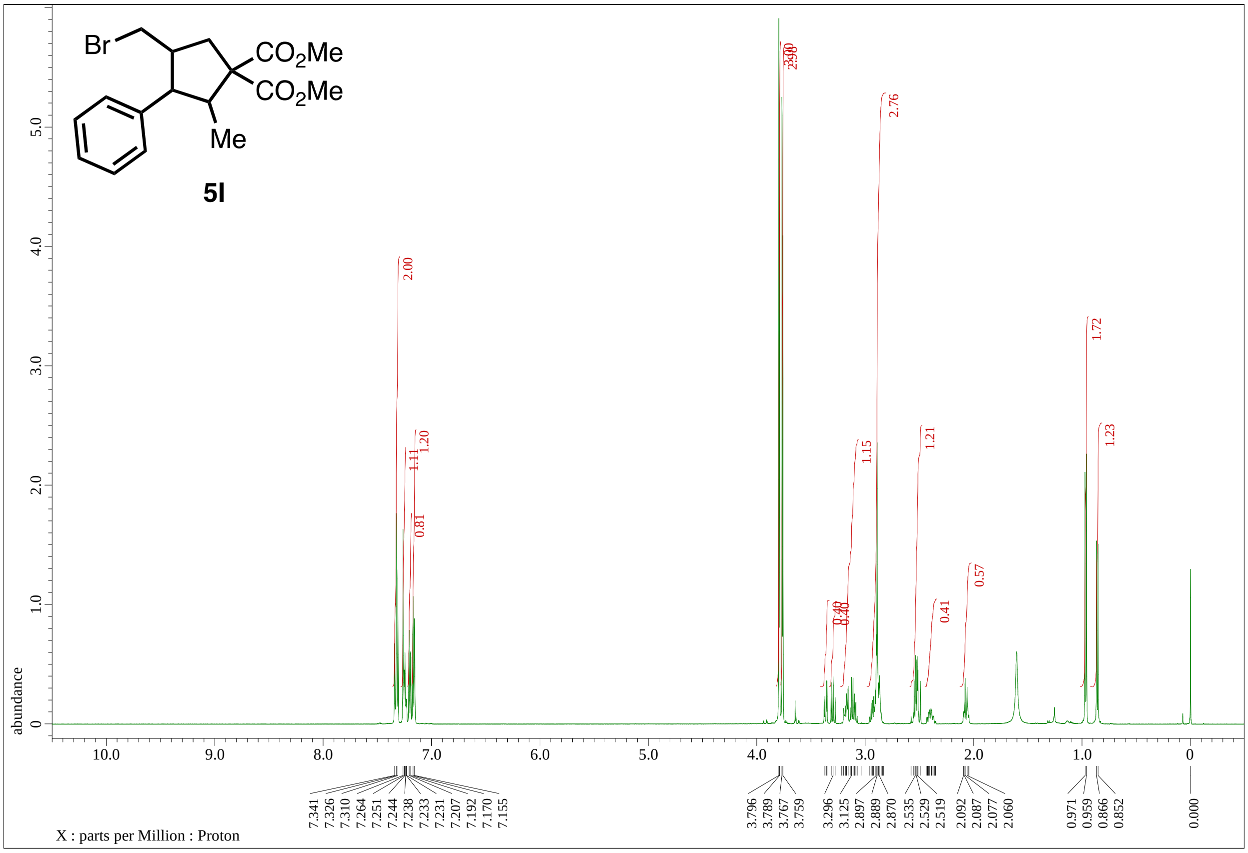

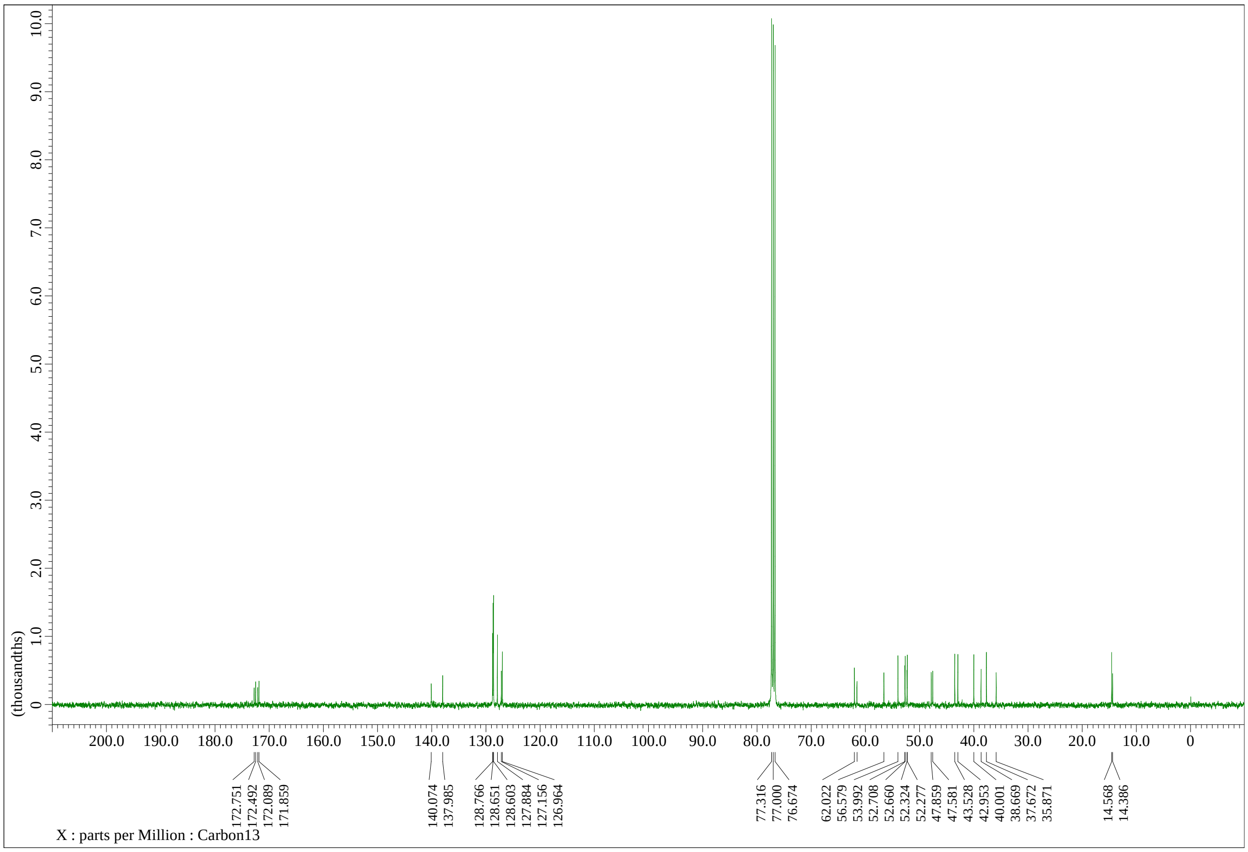

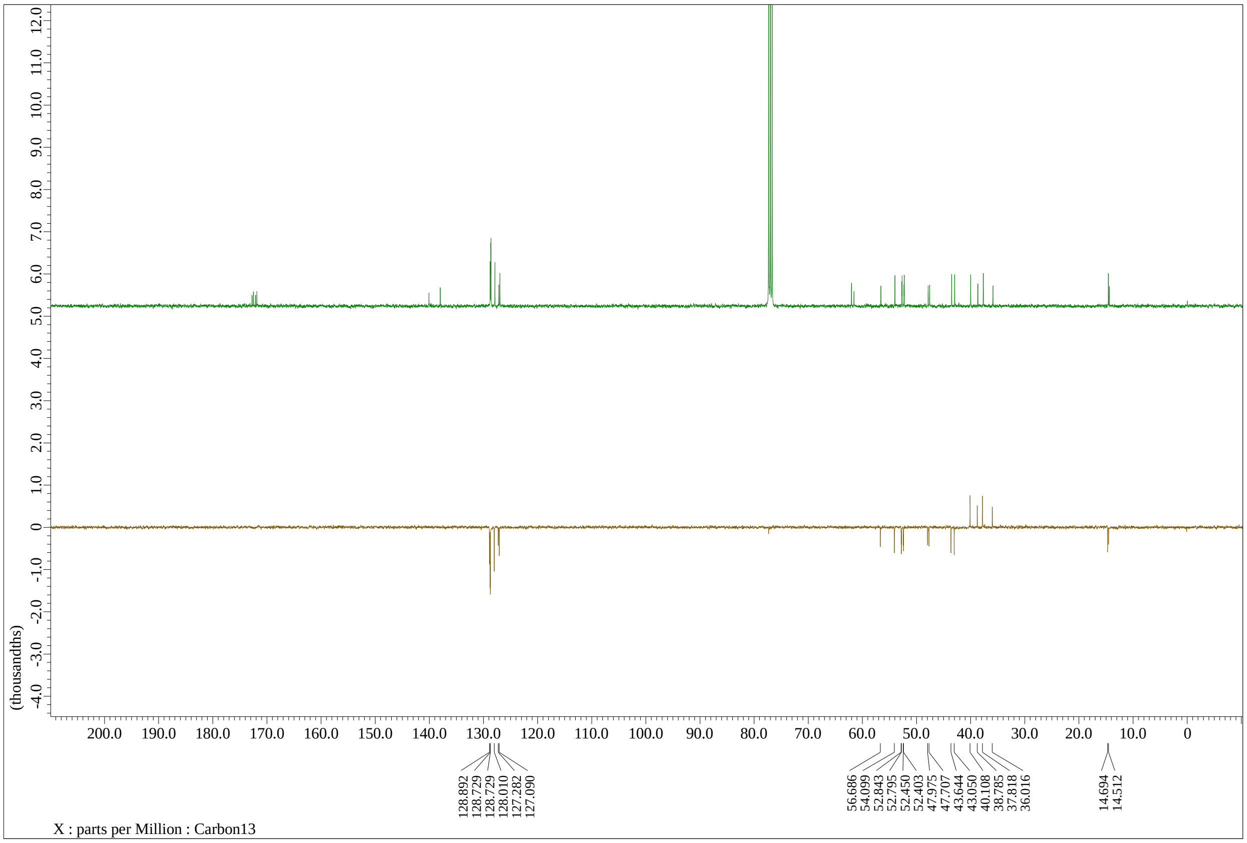

(thousandths)
X : parts per Million : Carbon13
128.892
128.729
128.729
128.010
127.282
127.090
56.686
54.099
52.843
52.795
52.450
52.403
47.975
47.707
43.644
43.050
40.108
38.785
37.818
36.016
14.694
14.512

10. ^{1}H NMR, ^{13}C NMR, ^{19}F and DEPT-135 NMR Spectra of 6

- ^{1}H and ^{13}C NMR Spectra of 6b

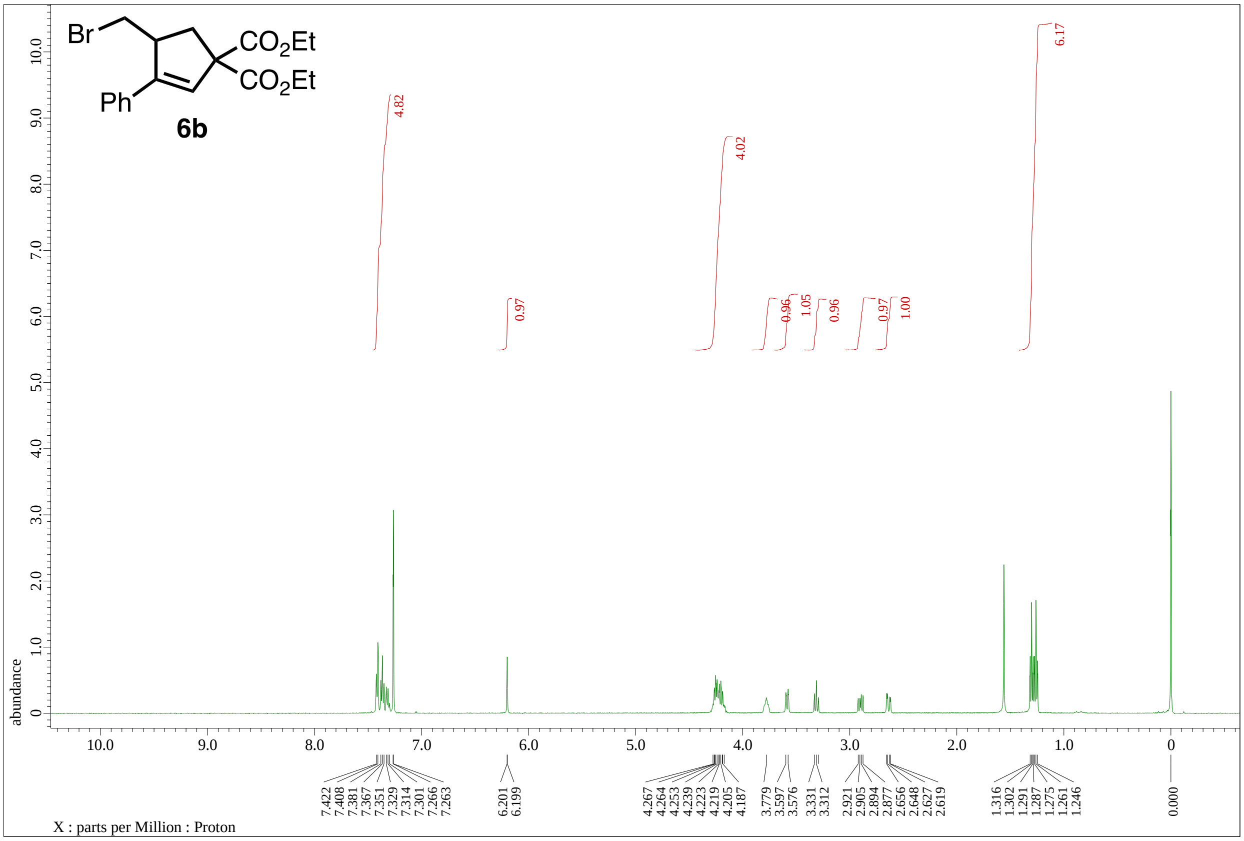

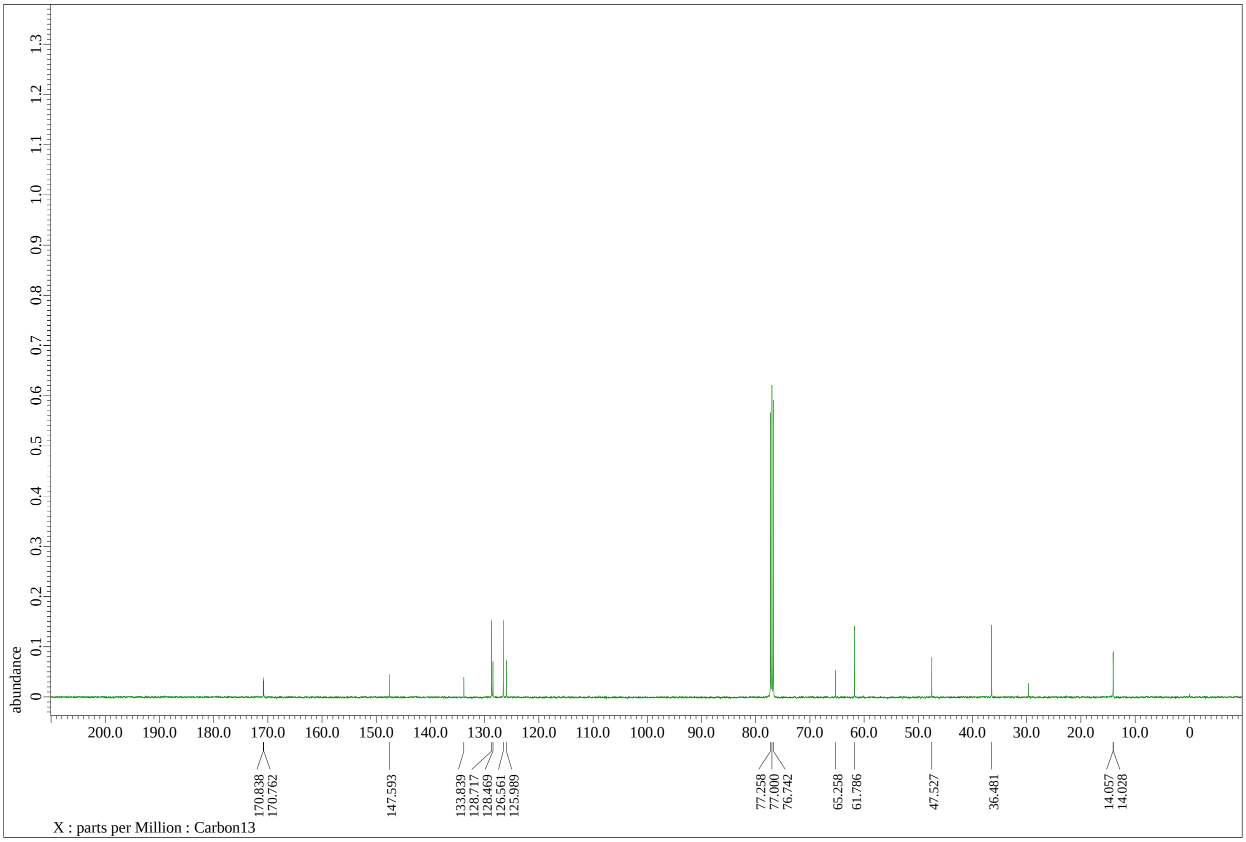

- ^{1}H, ^{13}C and DEPT-135 NMR Spectra of 6c

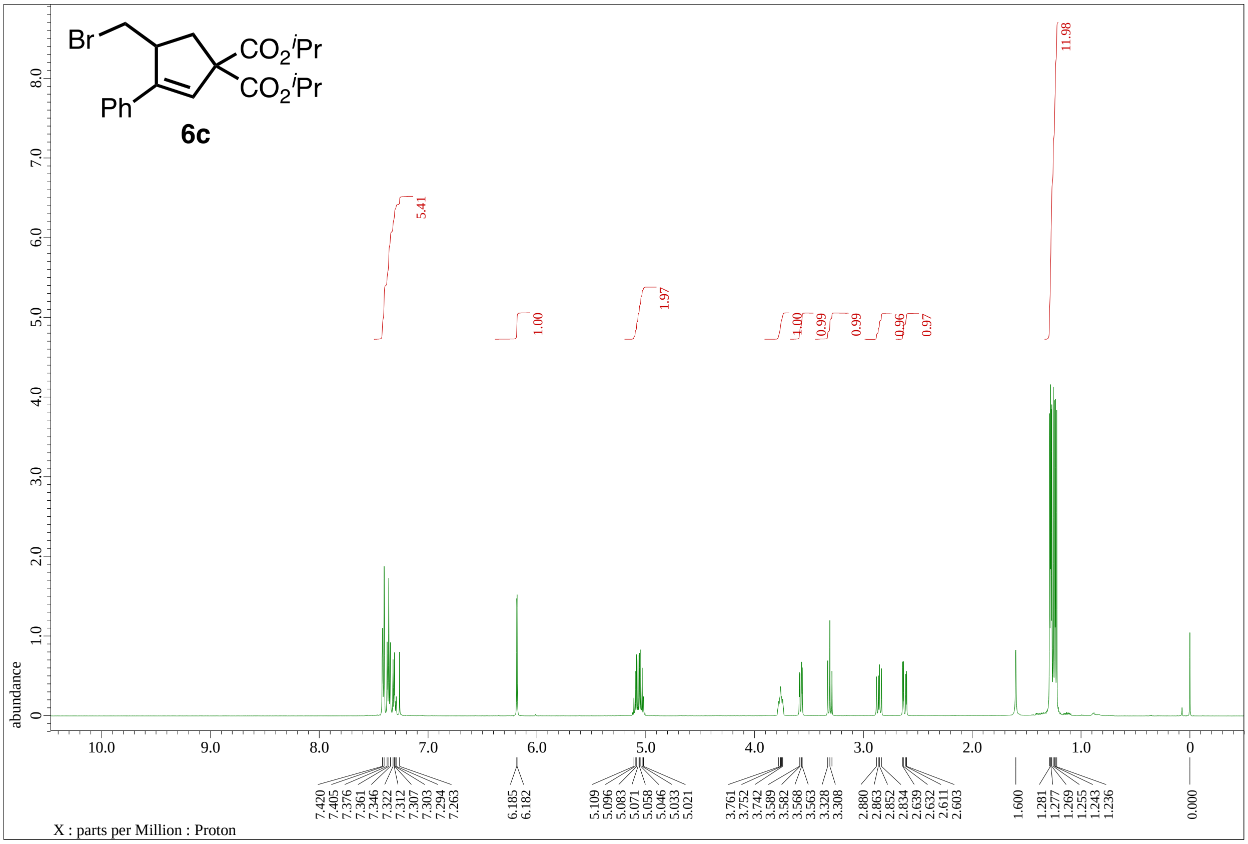

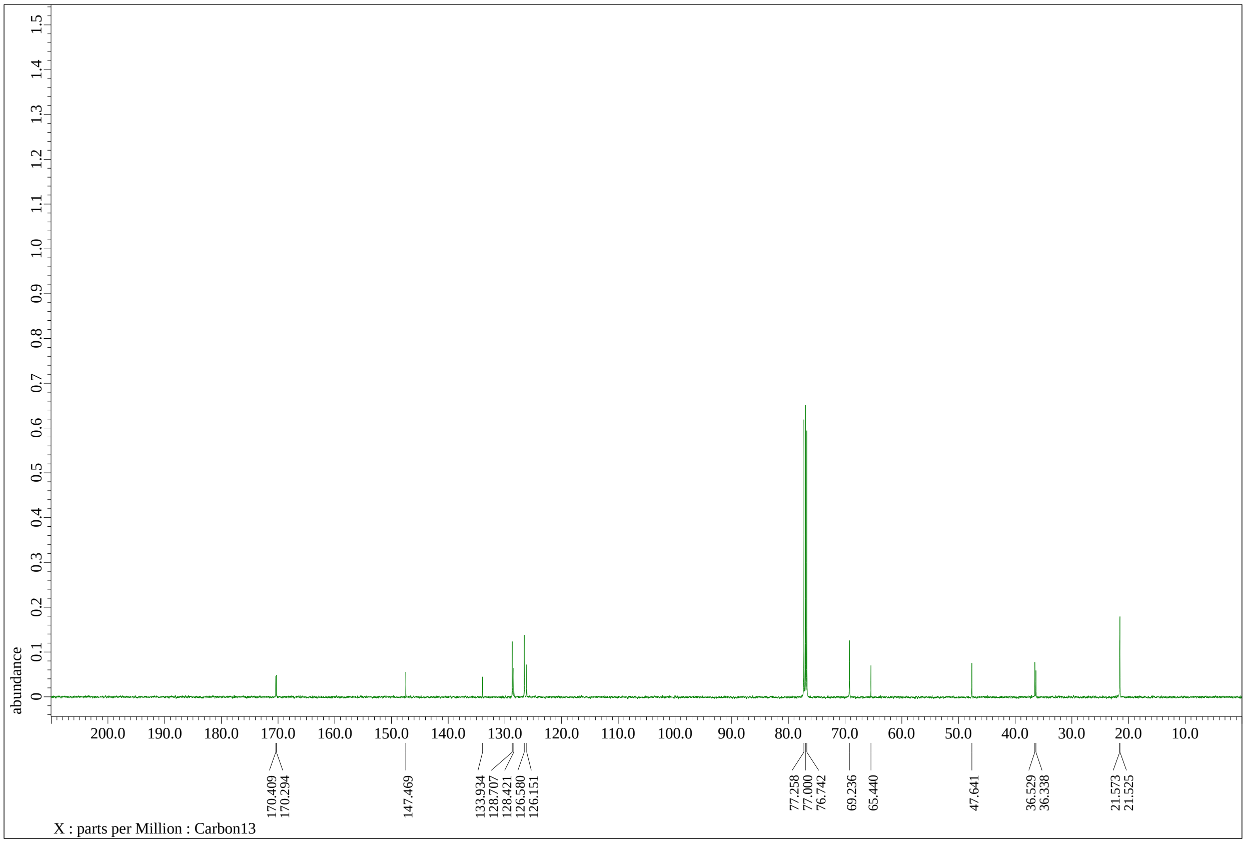

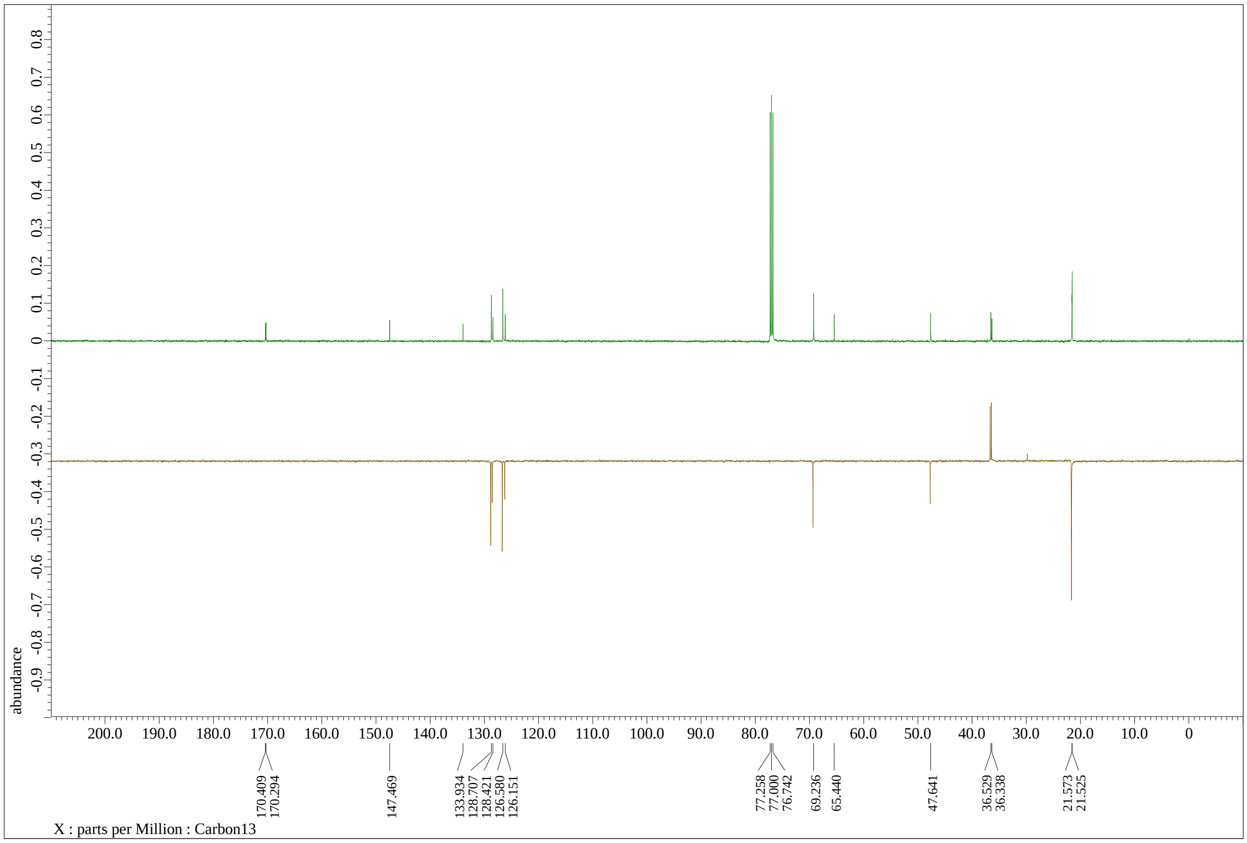

abundance
X : parts per Million : Carbon13
170.409
170.294
147.469
133.934
128.707
128.421
126.580
126.151
77.258
77.000
76.742
69.236
65.440
47.641
36.529
36.338
21.573
21.525

- ^{1}H and ^{13}C NMR Spectra of 6d

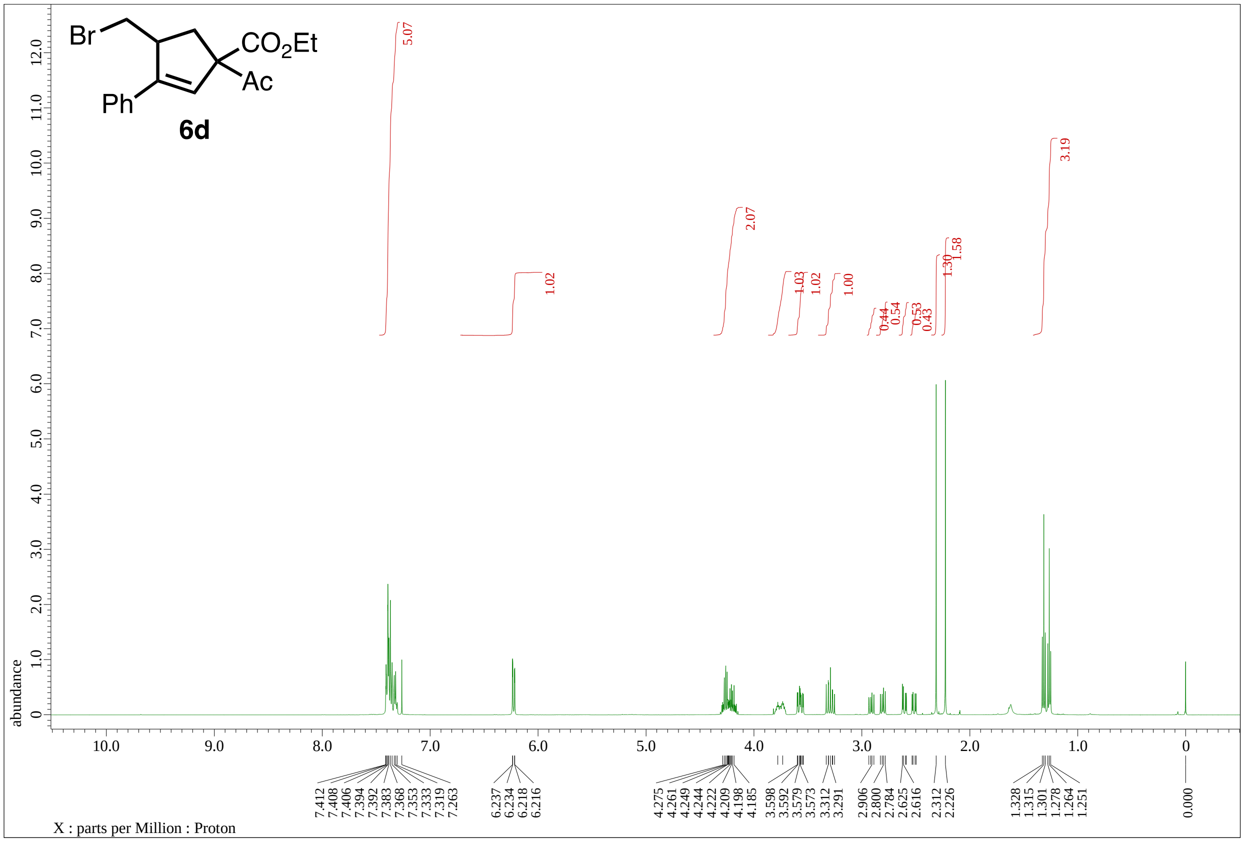

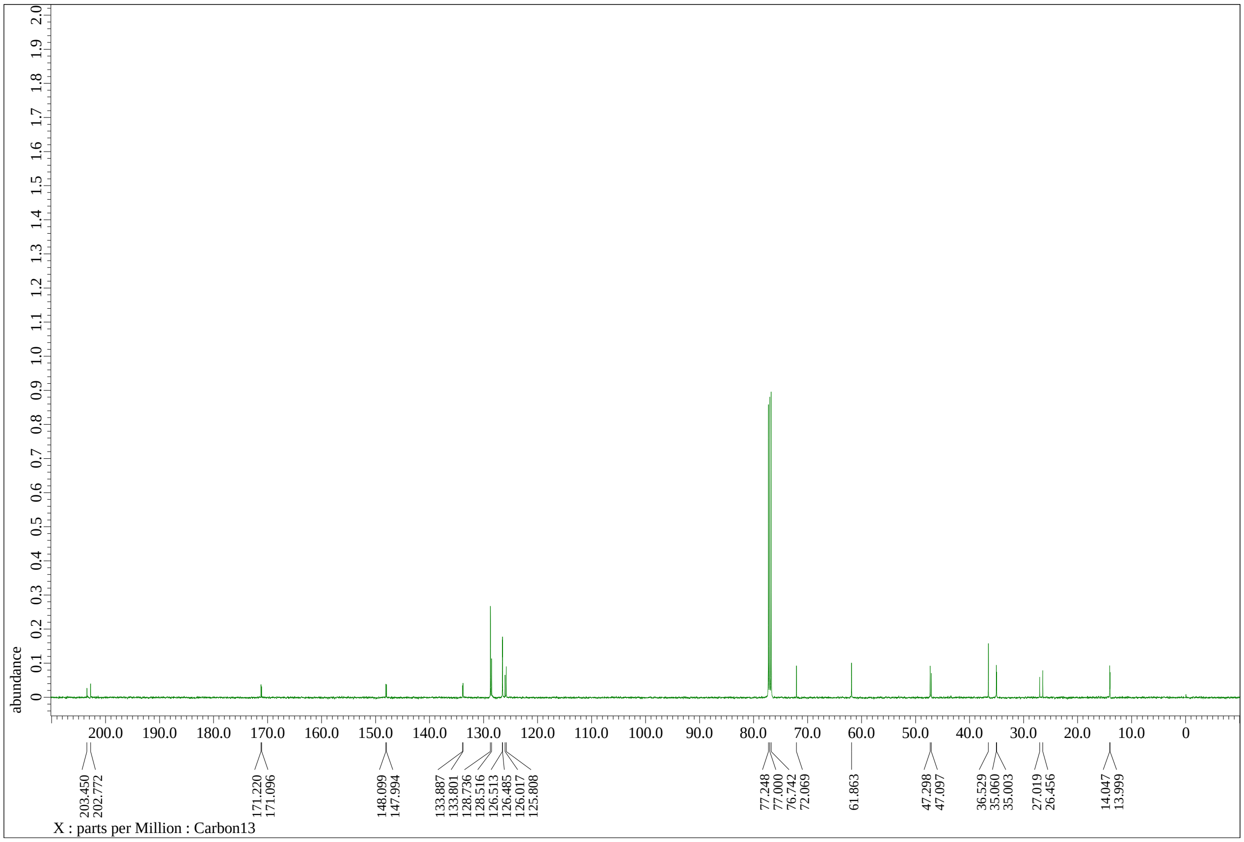

- ^{1}H and ^{13}C NMR Spectra of 6e

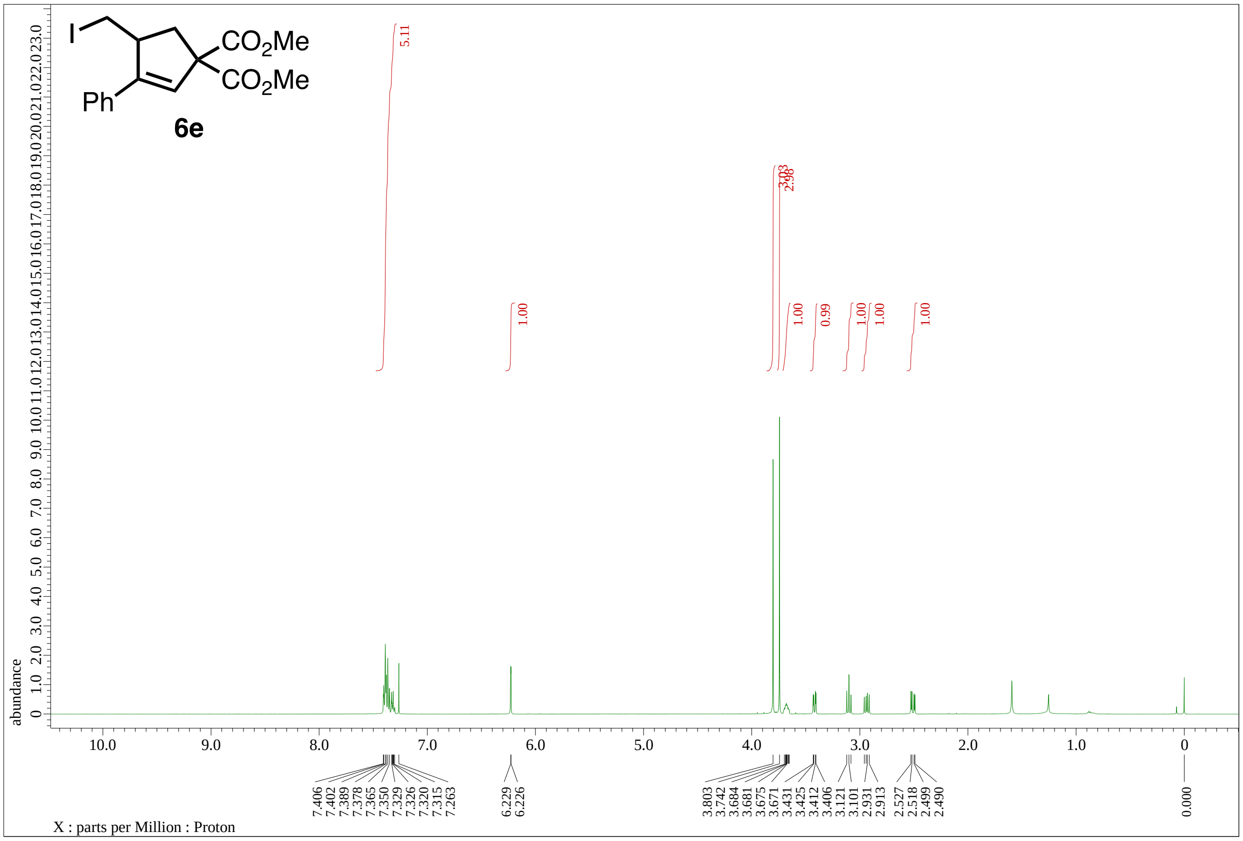

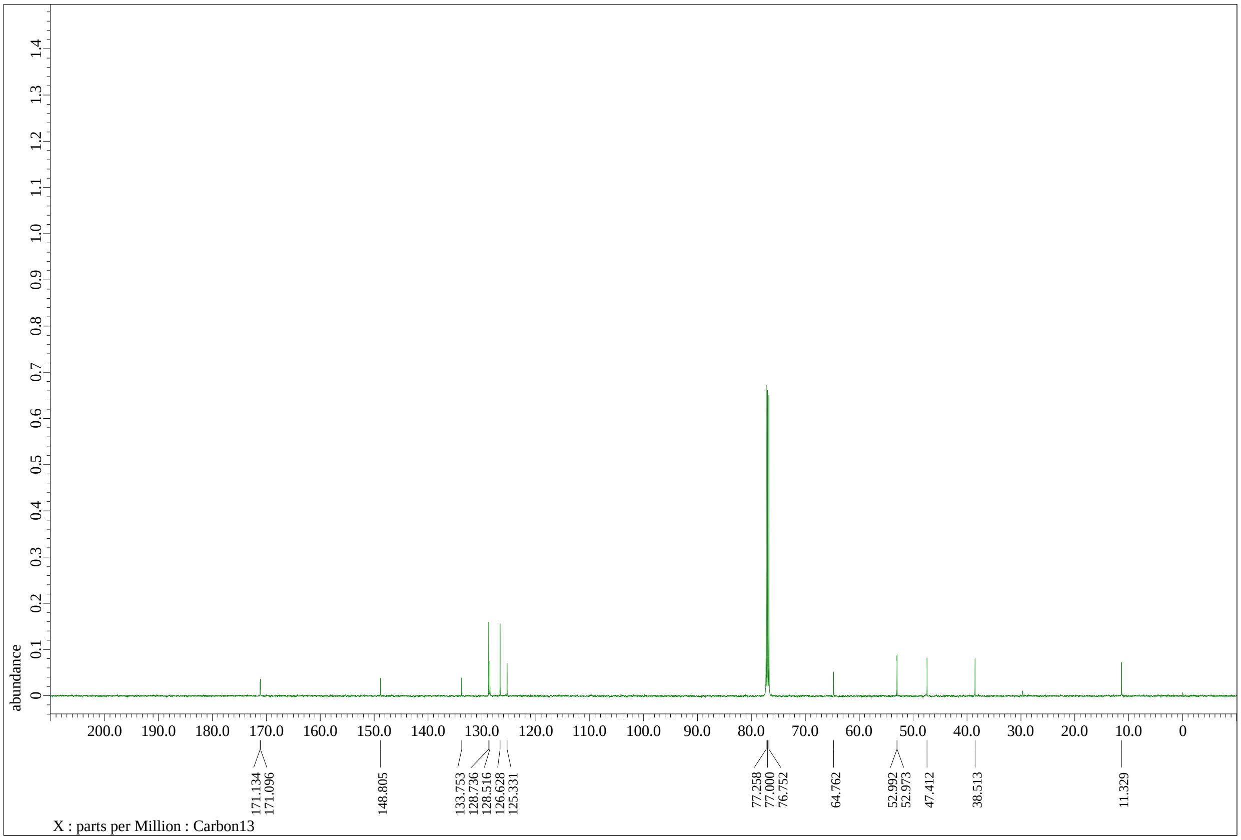

- ^{1}H, ^{13}C and DEPT-135 NMR Spectra of 6f

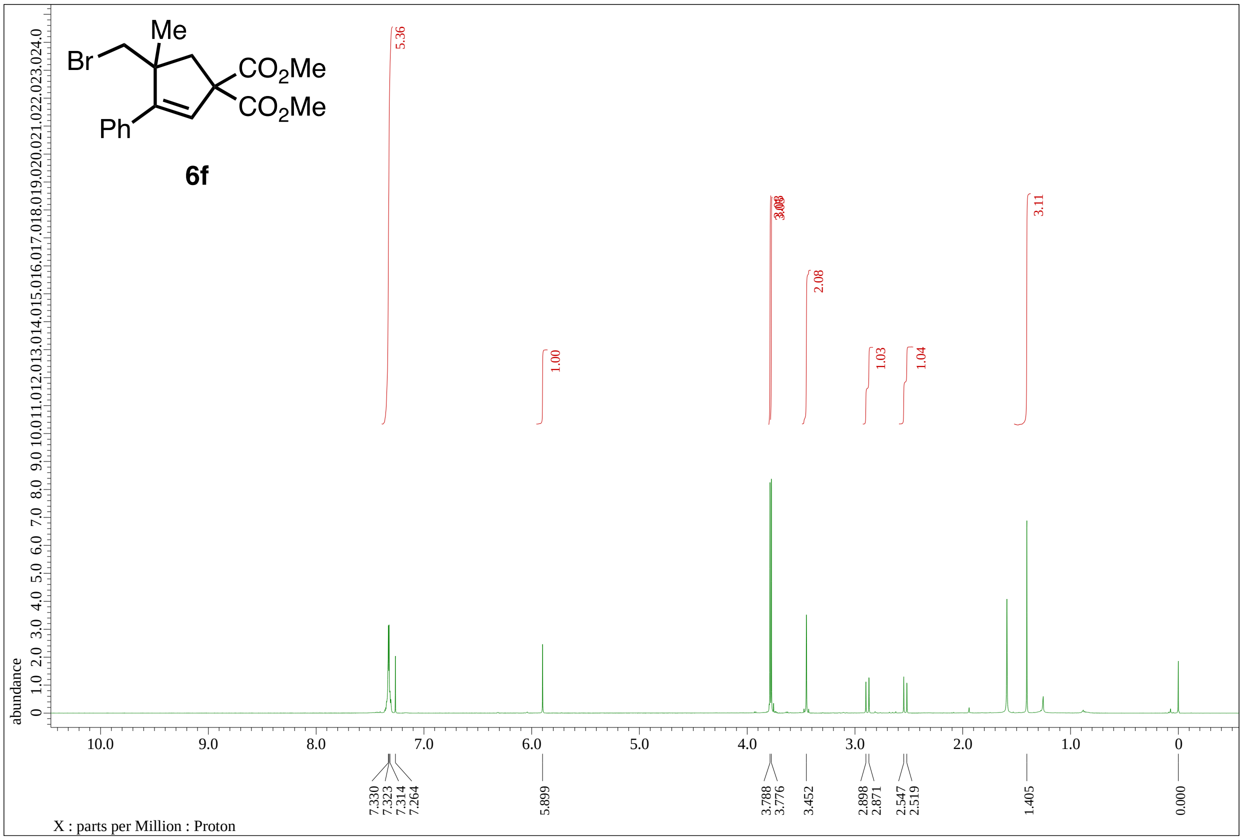

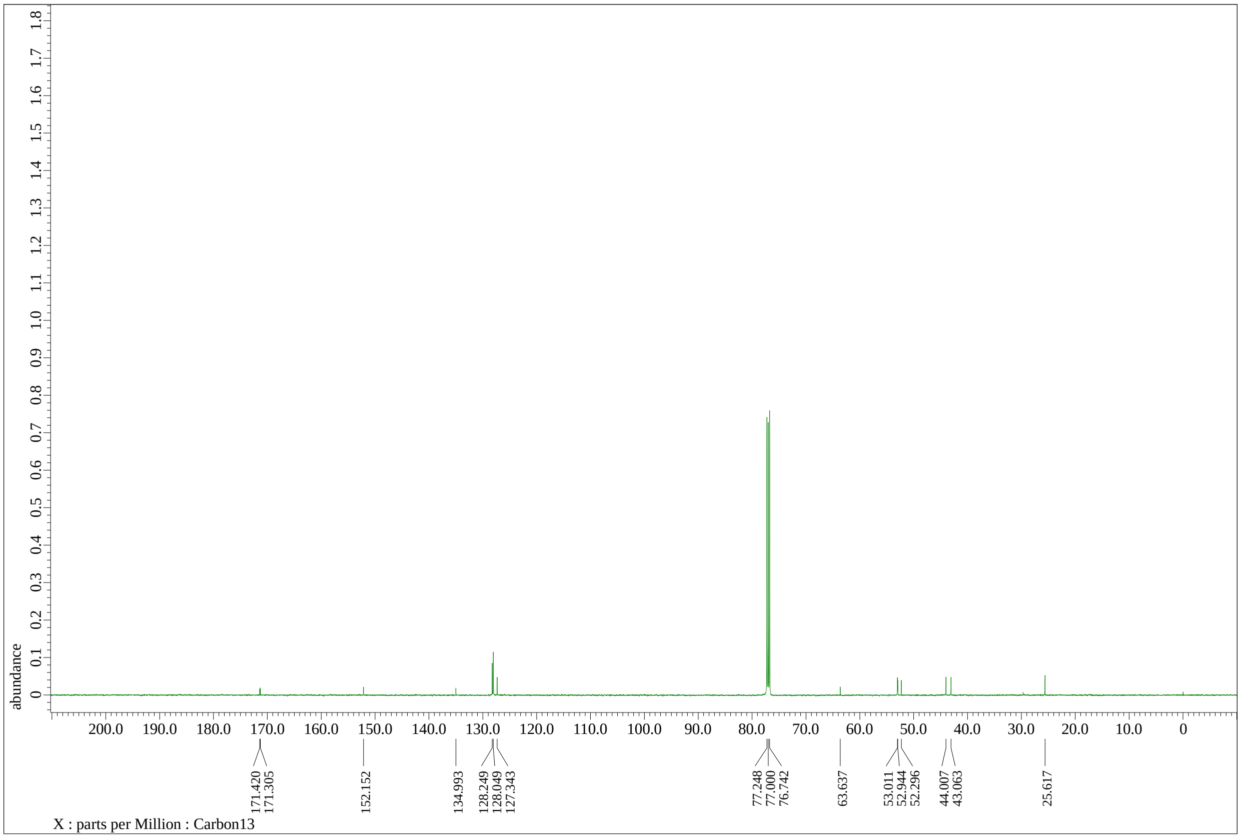

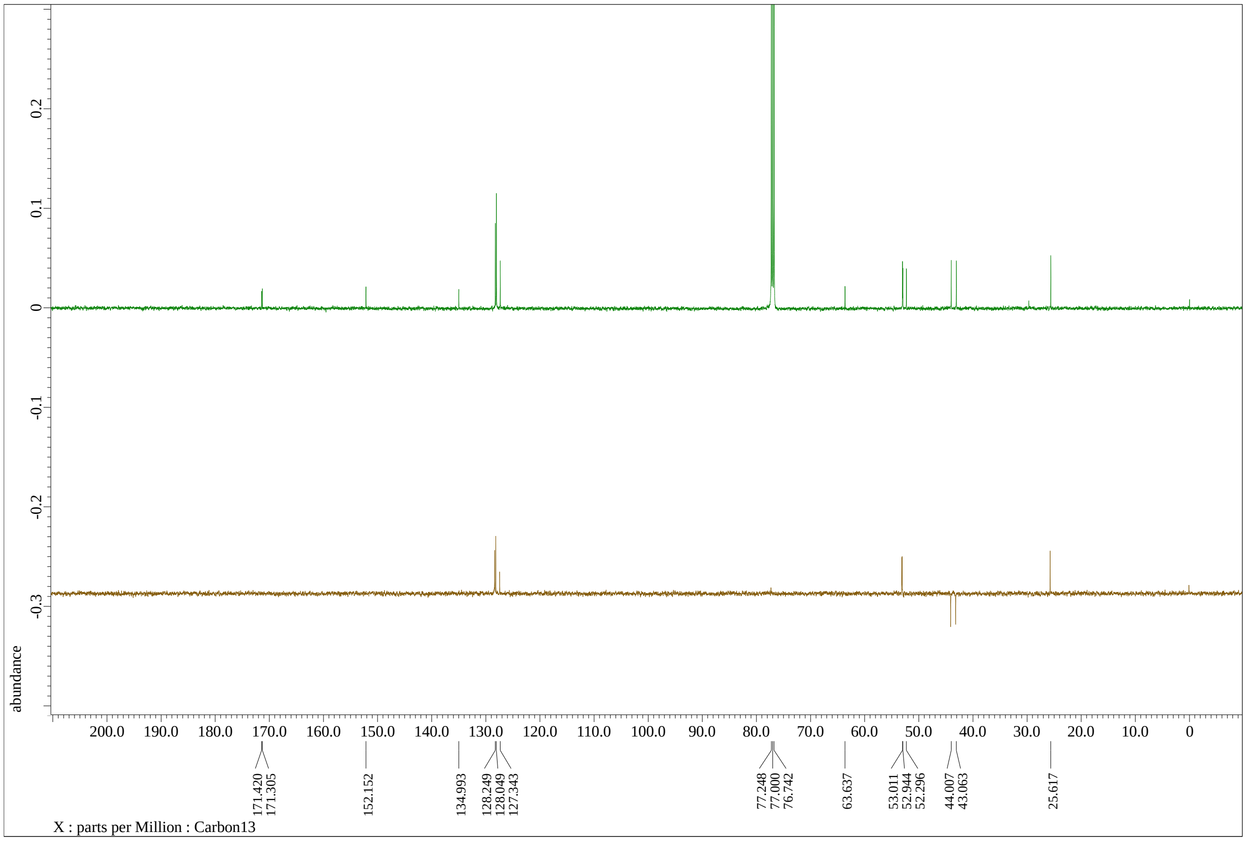

abundance
0.2
0.1
0
-0.1
-0.2
-0.3
200.0 190.0 180.0 170.0 160.0 150.0 140.0 130.0 120.0 110.0 100.0 90.0 80.0 70.0 60.0 50.0 40.0 30.0 20.0 10.0 0
171.420
171.305
152.152
134.993
128.249
128.049
127.343
77.248
77.000
76.742
63.637
53.011
52.944
52.296
44.007
43.063
25.617
X : parts per Million : Carbon13

- ^{1}H, ^{13}C and DEPT-135 NMR Spectra of 6g

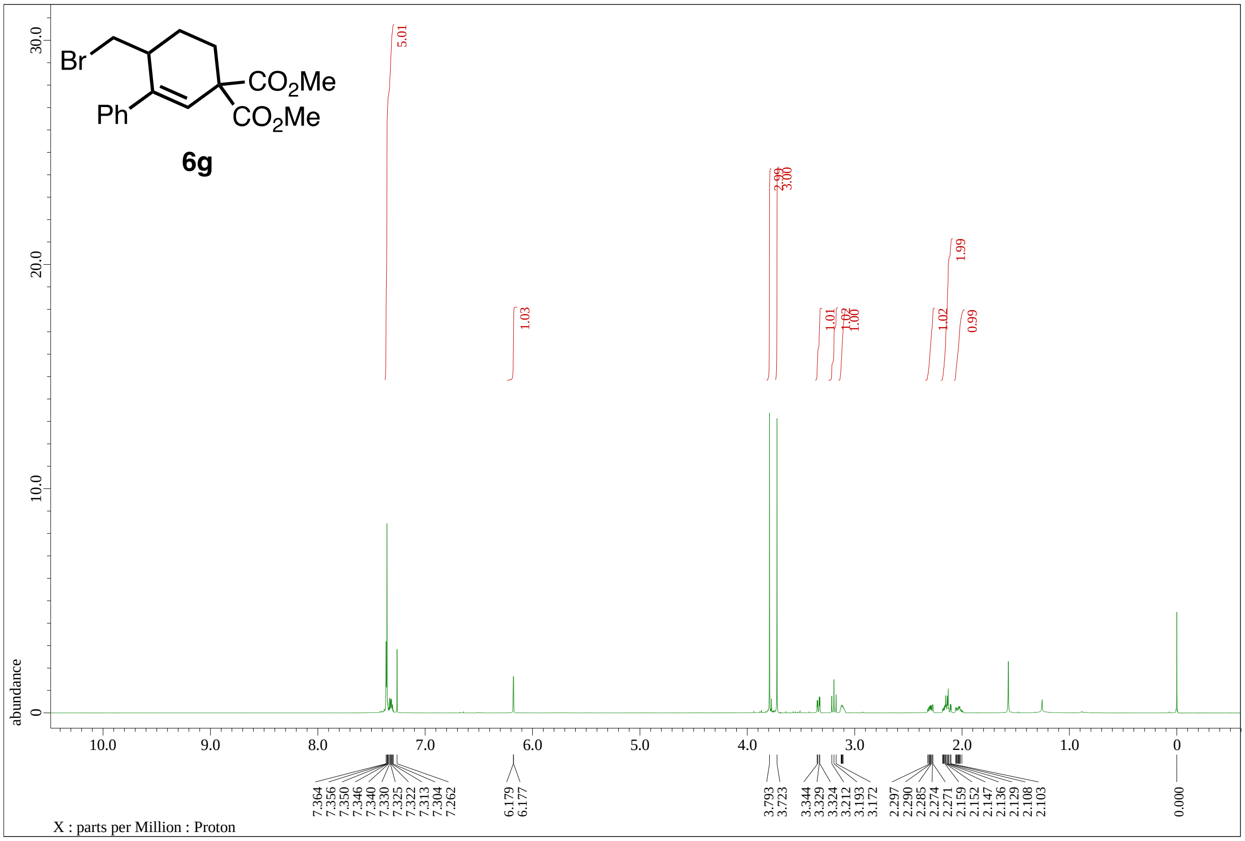

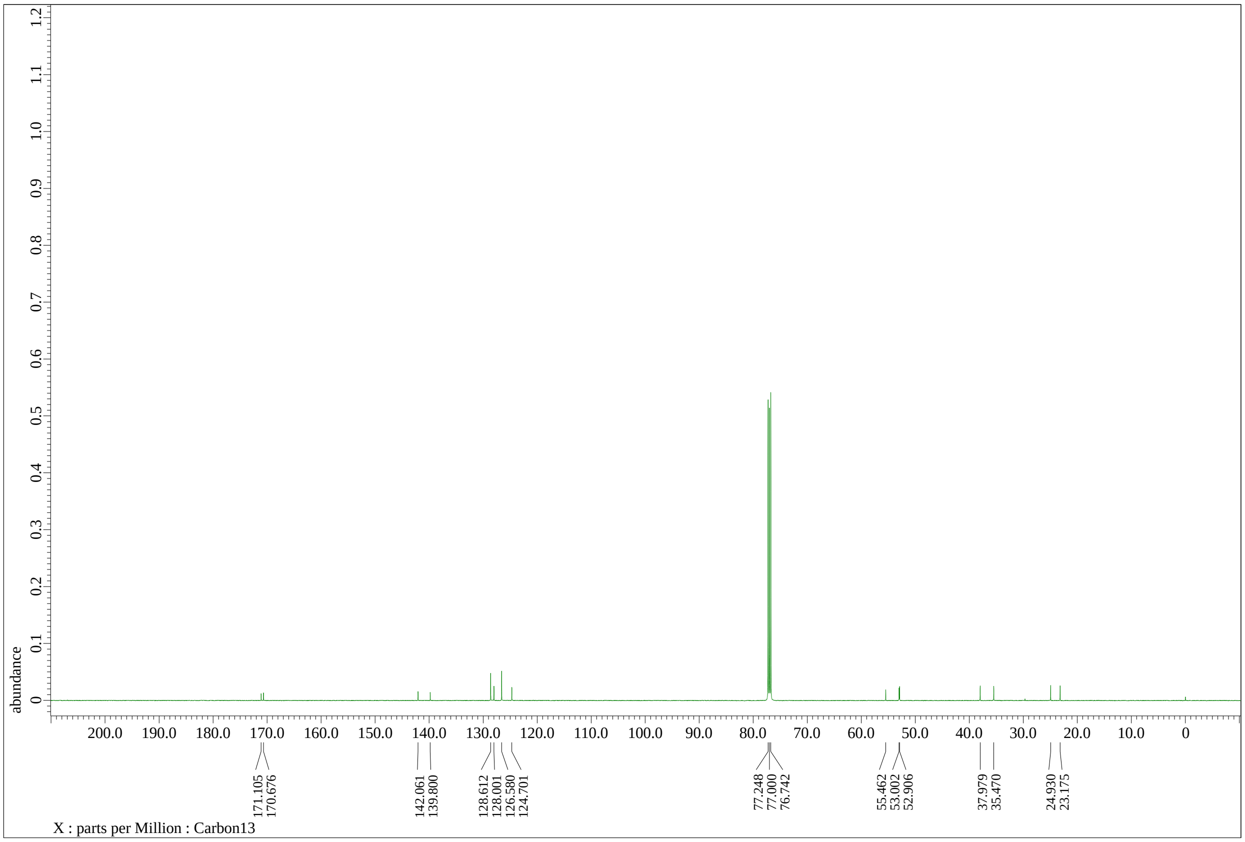

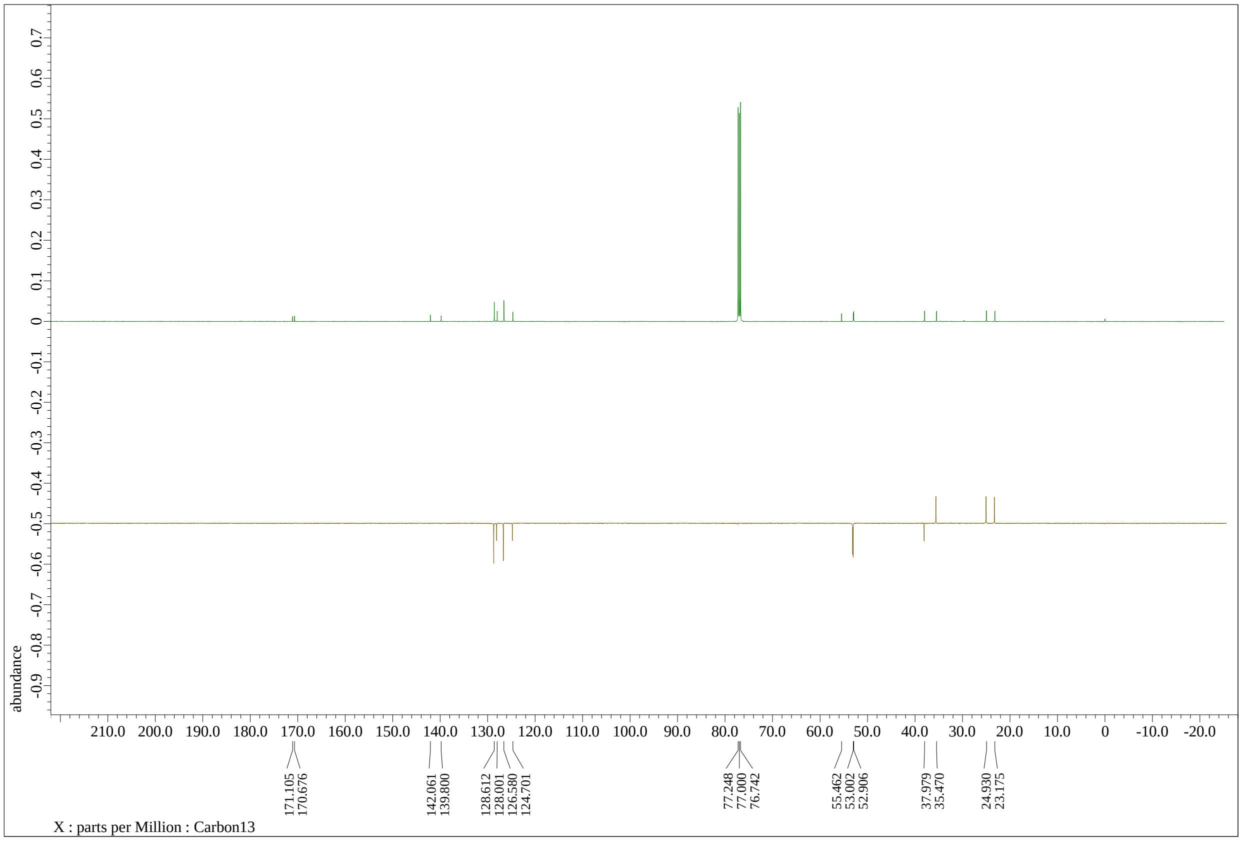
abundance
0.7
0.6
0.5
0.4
0.3
0.2
0.1
0
-0.1
-0.2
-0.3
-0.4
-0.5
-0.6
-0.7
-0.8
-0.9
210.0 200.0 190.0 180.0 170.0 160.0 150.0 140.0 130.0 120.0 110.0 100.0 90.0 80.0 70.0 60.0 50.0 40.0 30.0 20.0 10.0 0 -10.0 -20.0
171.105
170.676
142.061
139.800
128.612
128.001
126.580
124.701
77.248
77.000
76.742
55.462
53.002
52.906
37.979
35.470
24.930
23.175
X : parts per Million : Carbon13

- ^{1}H and ^{13}C NMR Spectra of 6h

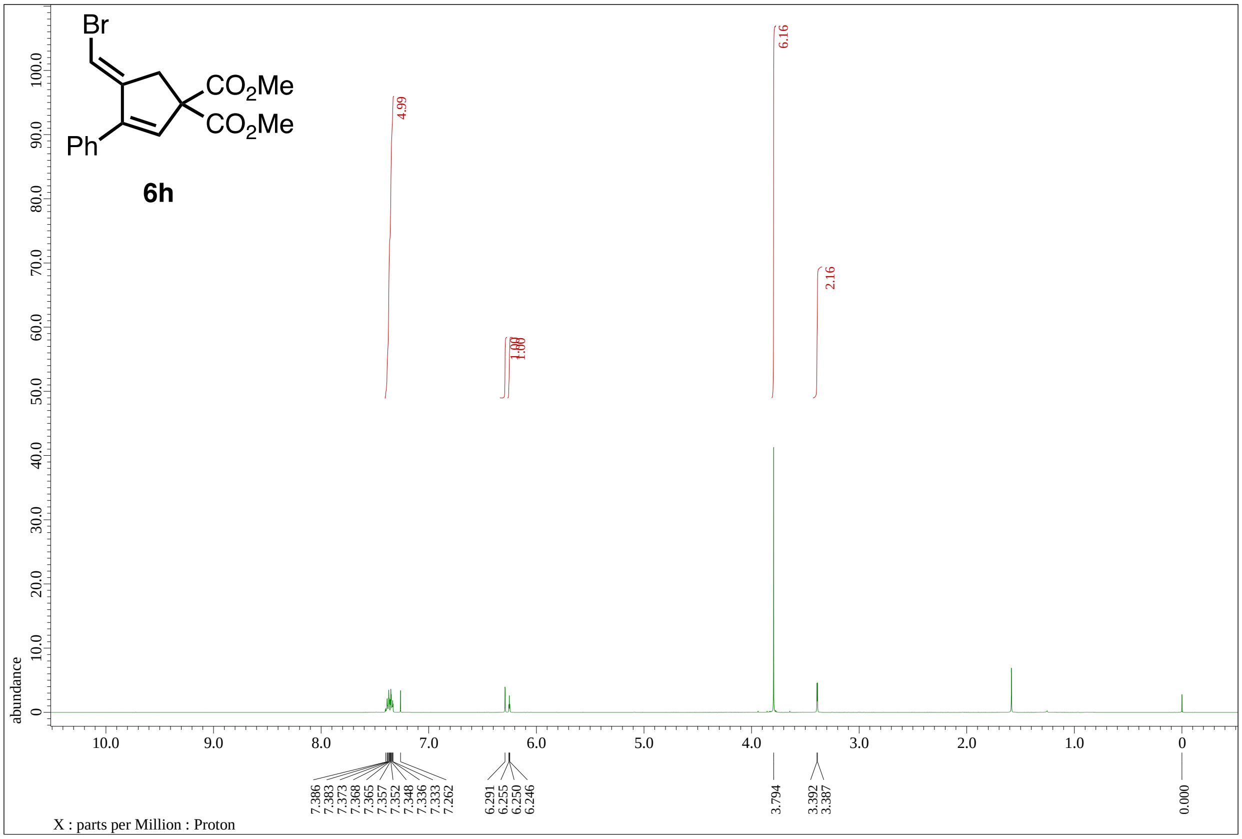

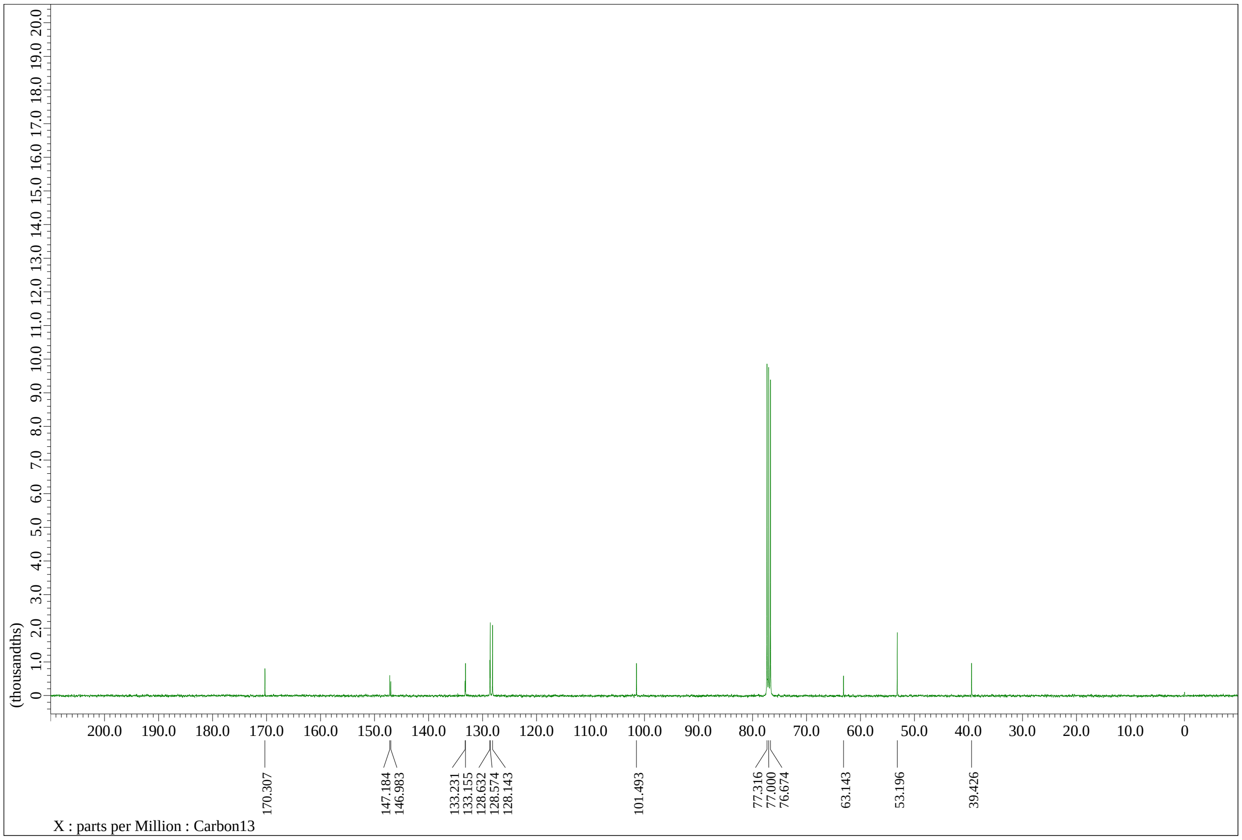

11. ^{1}H-^{1}H NOESY spectrum of 6h

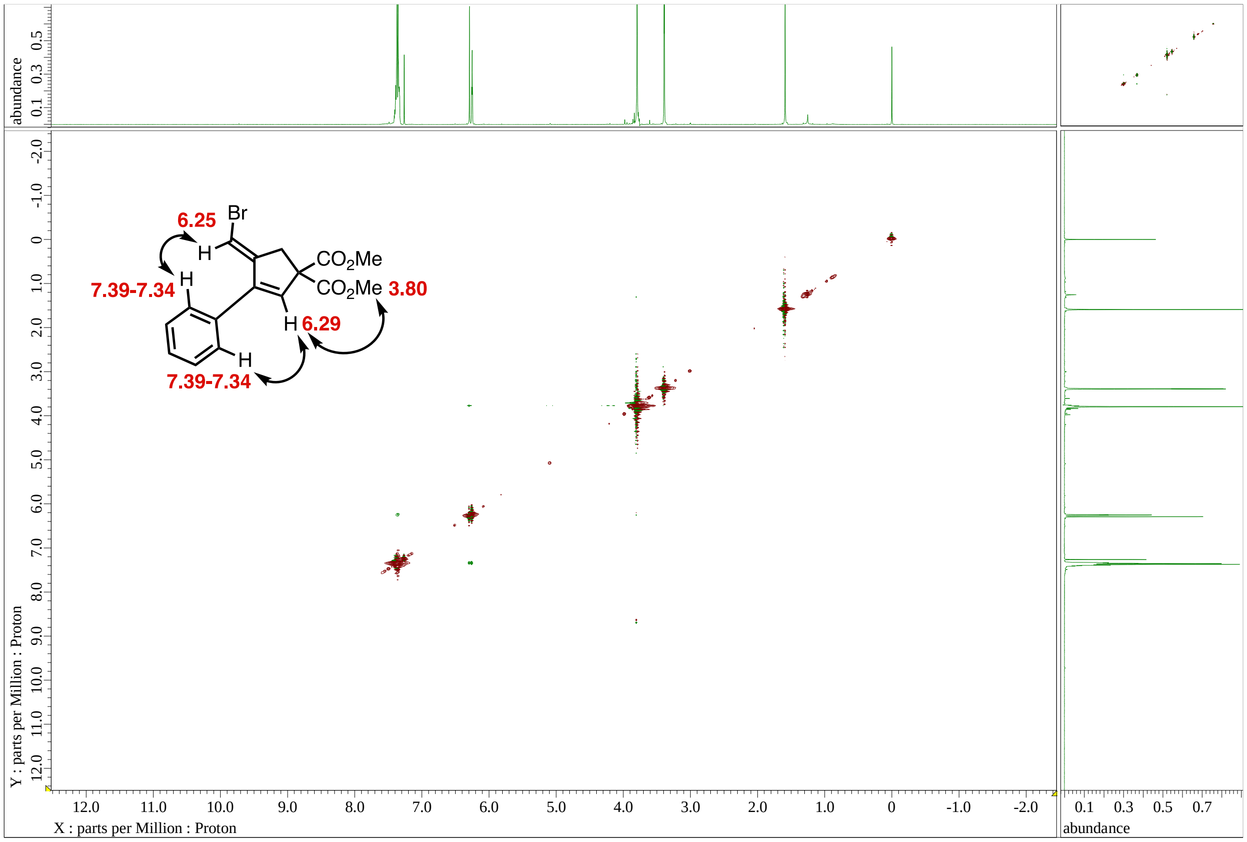

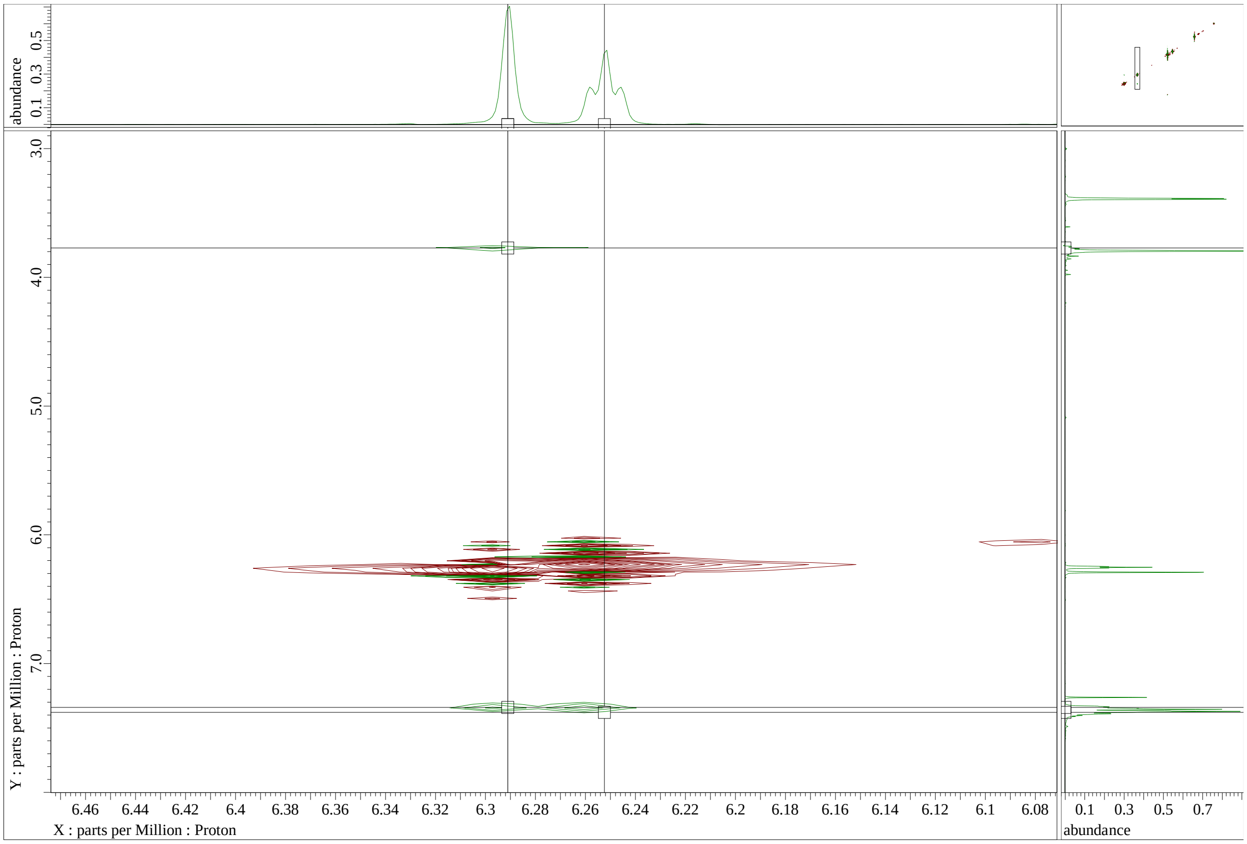

12. ^{1}H NMR, ^{13}C NMR, ^{19}F and DEPT-135 NMR Spectra of 7

- ^{1}H and ^{13}C NMR Spectra of 7a

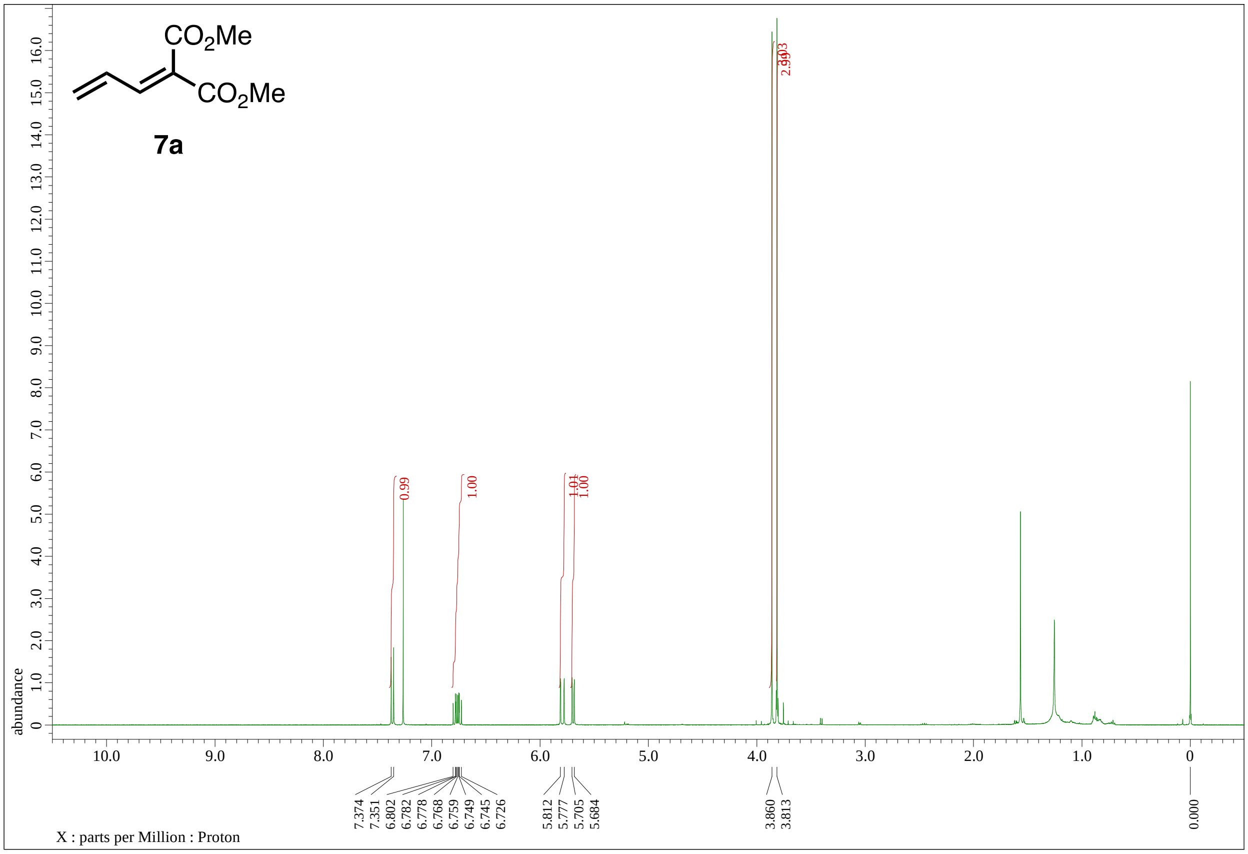

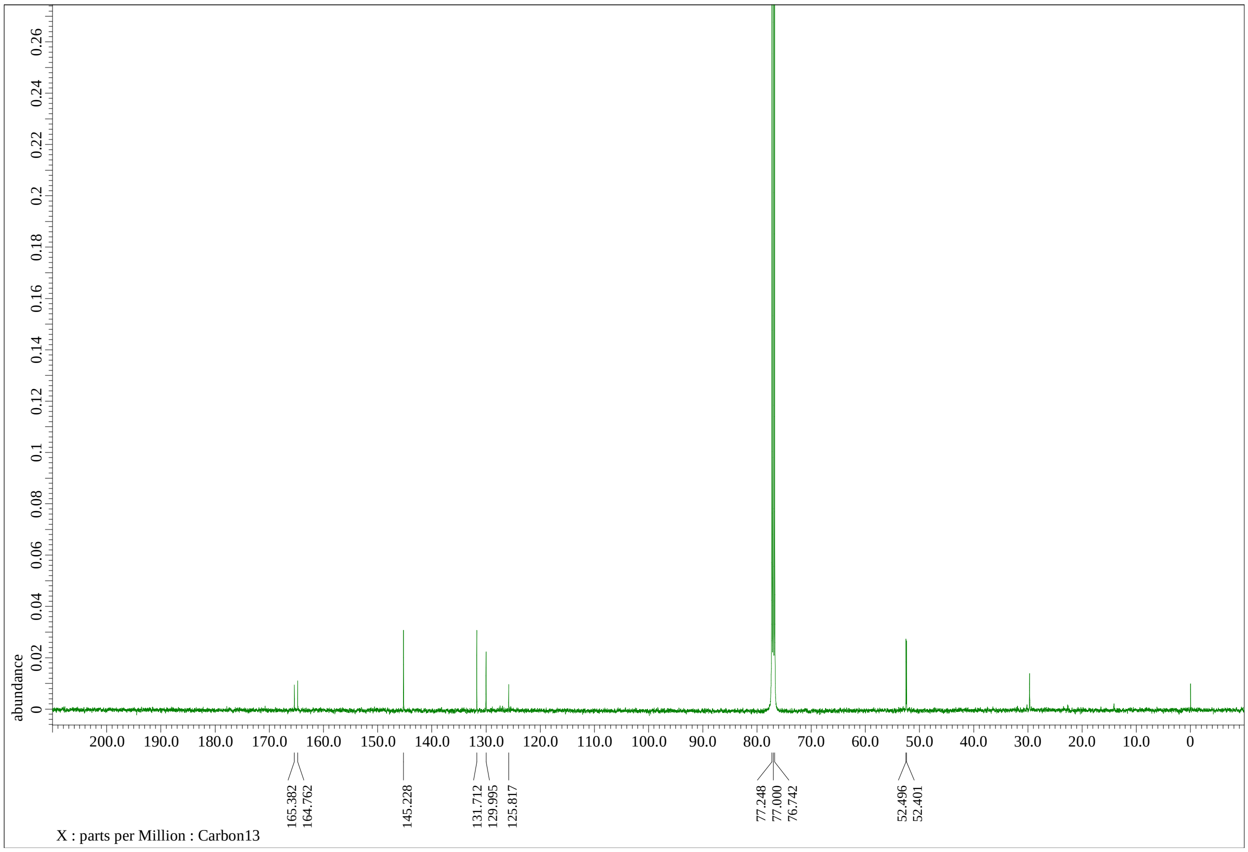

- ^{1}H and ^{13}C NMR Spectra of 7b

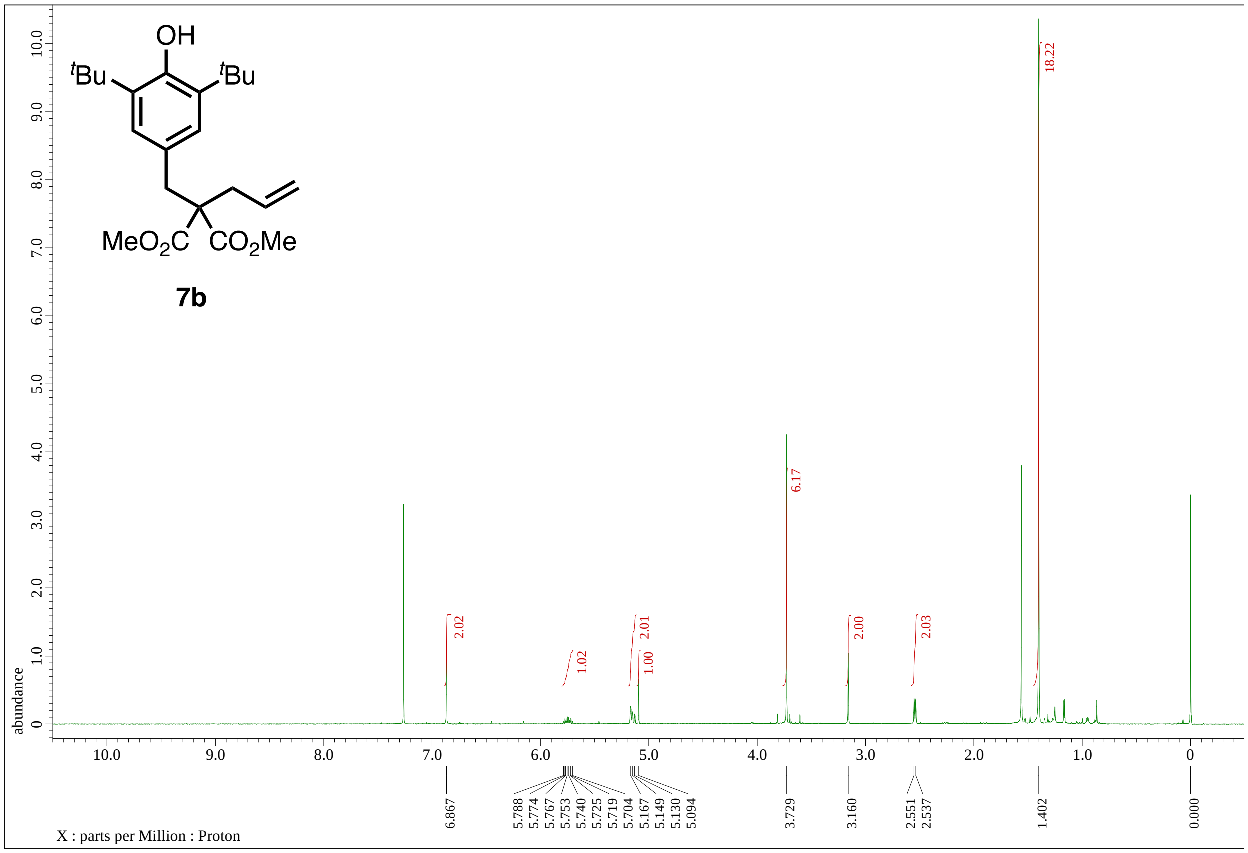

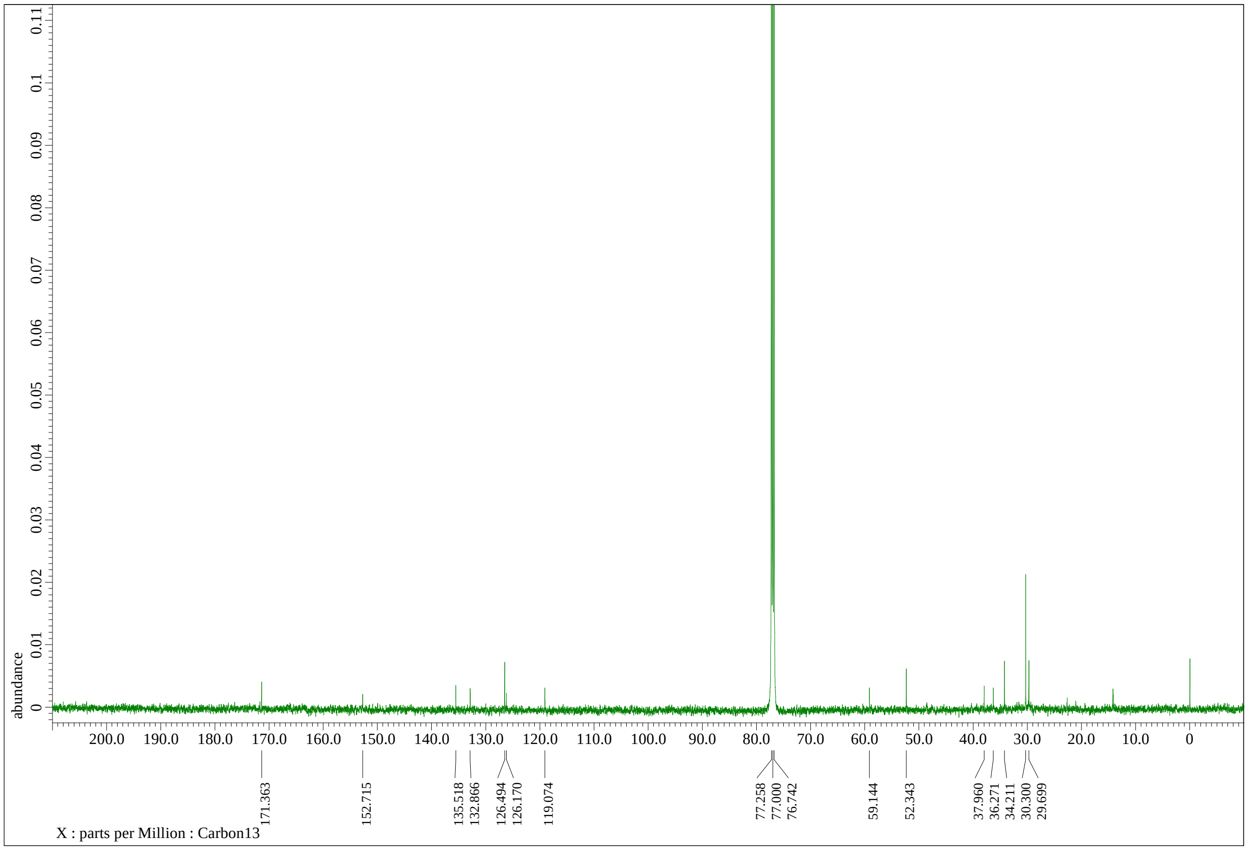

Supplement: Supplementary file 1 [file molecules-26-06781-s001.zip › molecules-1418767-supplementary.pdf]
